# Economic model of community-based falls prevention: seeking methodological solutions in evaluating the efficiency and equity of UK guideline recommendations

**DOI:** 10.1186/s12877-023-03916-z

**Published:** 2023-03-30

**Authors:** Joseph Kwon, Hazel Squires, Tracey Young

**Affiliations:** 1 Nuffield Department of Primary Care Health Sciences, University of Oxford, Radcliffe Primary Care Building, Radcliffe Observatory Quarter, Woodstock Road, Oxford, OX2 6GG England; 2 School of Health and Related Research, University of Sheffield, Regent Court (ScHARR), 30 Regent Street, S1 4DA Sheffield, England

**Keywords:** Falls prevention, Economic model, NICE falls prevention guideline, Equity

## Abstract

**Background:**

Falls significantly harm geriatric health and impose substantial costs on care systems and wider society. Decision modelling can inform the commissioning of falls prevention but face methodological challenges, including: (1) capturing non-health outcomes and societal intervention costs; (2) considering heterogeneity and dynamic complexity; (3) considering theories of human behaviour and implementation; and (4) considering issues of equity. This study seeks methodological solutions in developing a credible economic model of community-based falls prevention for older persons (aged 60 +) to inform local falls prevention commissioning as recommended by UK guidelines.

**Methods:**

A framework for conceptualising public health economic models was followed. Conceptualisation was conducted in Sheffield as a representative local health economy. Model parameterisation used publicly available data including English Longitudinal Study of Ageing and UK-based falls prevention trials. Key methodological developments in operationalising a discrete individual simulation model included: (1) incorporating societal outcomes including productivity, informal caregiving cost, and private care expenditure; (2) parameterising dynamic falls-frailty feedback loop whereby falls influence long-term outcomes via frailty progression; (3) incorporating three parallel prevention pathways with unique eligibility and implementation conditions; and (4) assessing equity impacts through distributional cost-effectiveness analysis (DCEA) and individual-level lifetime outcomes (e.g., number reaching ‘fair innings’). Guideline-recommended strategy (RC) was compared against usual care (UC). Probabilistic sensitivity, subgroup, and scenario analyses were conducted.

**Results:**

RC had 93.4% probability of being cost-effective versus UC at cost-effectiveness threshold of £20,000 per QALY gained under 40-year societal cost-utility analysis. It increased productivity and reduced private expenditure and informal caregiving cost, but productivity gain and private expenditure reduction were outstripped by increases in intervention time opportunity costs and co-payments, respectively. RC reduced inequality delineated by socioeconomic status quartile. Gains in individual-level lifetime outcomes were small. Younger geriatric age groups can cross-subsidise their older peers for whom RC is cost-ineffective. Removing the falls-frailty feedback made RC no longer efficient or equitable versus UC.

**Conclusion:**

Methodological advances addressed several key challenges associated with falls prevention modelling. RC appears cost-effective and equitable versus UC. However, further analyses should confirm whether RC is optimal versus other potential strategies and investigate feasibility issues including capacity implications.

**Supplementary Information:**

The online version contains supplementary material available at 10.1186/s12877-023-03916-z.

## Background

The global demographic trend of population ageing will increase the need for greater understanding of geriatric health challenges and related policy responses [1]. In the UK, the proportion of the population aged 65 + is projected to increase from 18.3% in 2018 to 24.2% in 2038 [2]. Falls are one of the key geriatric syndromes and are closely associated with frailty [3–5]. More than half of falls in older populations occur in community settings: i.e., excluding residential care settings such as nursing homes and hospital wards [6]. Falls impose significant morbidity and mortality burdens on older people [7], including fear of falling [8–10], depression [11], and functional decline and dependence [12–15], as well as substantial costs for health and social care systems [16–18], and wider society in terms of private care expenditures and informal caregiver burden [19–21]. Their close association with frailty and multimorbidity, and the latter’s association with socioeconomic status [22, 23], likely induce higher burden for socially deprived subgroups. Falls and falls prevention are thus closely tied with issues surrounding social inequities of health [24].

Importantly, there are many community-based falls prevention interventions shown to be efficacious in randomised controlled trial (RCT) settings [25–27], as well as established guidelines [28–30]. In the UK, the clinical guideline 161 (CG161) issued by the National Institute for Health and Care Excellence (NICE) [28] – currently being updated [31] – offers normative guidance to clinical professionals and commissioners. CG161 emphasises the *proactive* pathway initiated by older persons’ routine contact with health and social care professionals: older persons are screened for falls risk based on falls history and gait/balance impairment, and if at high risk, referred to multifactorial intervention encompassing multidisciplinary risk assessment and tailored treatments. CG161 also incorporates the *reactive* pathway, wherein older persons who experienced a fall requiring medical attention are referred to multifactorial intervention. Another potential pathway mentioned in other UK guidelines is the *self-referred* pathway, wherein older persons enrol in a falls prevention intervention (e.g., group exercise) without direct professional referral [32, 33]. The final intervention strategy should consider the eligibility and implementation conditions in all three parallel pathways.

Health economic evaluation involves comparative analyses of alternative healthcare strategies in terms of costs and consequences with the primary purpose of informing the efficient use of scarce resources under a constrained healthcare budget [34]. Community-based falls prevention has accordingly been the subject of numerous economic evaluations, including those conducted via decision modelling [35, 36]. However, CG161 is currently informed by limited economic evidence, specifically by a single Markov cohort model that evaluated a multifactorial intervention and an exercise intervention over the lifetime horizon [37]. The model contained several key methodological limitations which have been appraised previously [35, 38]. Hence, a de novo economic evaluation of CG161-recommended falls prevention strategy is strongly warranted given its guiding role for commissioning and its emphasis on the resource-intensive multifactorial interventions.

As a vehicle for economic evaluation, decision models offer key strengths relative to evaluations alongside individual clinical studies, including [34, 39, 40]: comparing all potential intervention strategies and scenarios; considering all relevant costs and outcomes of interventions over long time horizons; and making evaluation results applicable to the population-level decision context. Nevertheless, decision modelling also brings key conceptual and methodological challenges. First, the model structure should be informed a priori by a conceptual model that elaborates key features of falls epidemiology, falls prevention, and priority setting challenges without being constrained by data and technical considerations [41–43]. Second, specific challenges arise when modelling is applied to public health decision problems concerning broad heterogeneous target populations rather than clinical ones concerning narrowly defined patient groups [44].

Community-based falls prevention can be classified as a geriatric public health intervention, and its modelling should therefore address these public health modelling challenges, which have previously been divided into four categories [38]: (1) capturing non-health outcomes and societal intervention costs; (2) considering heterogeneity and dynamic complexity; (3) considering theories of human behaviour and implementation; and (4) considering issues of equity. A modelling study focused on seeking methodological solutions to these challenges will contribute towards improving the credibility of models in falls prevention and in further geriatric public health areas.

Therefore, this study aims to seek methodological solutions in developing a credible economic model of community-based falls prevention interventions for older persons (aged 60 +) which will assess the health economic performance of the UK guideline-recommended falls prevention strategy relative to current local practice (represented by that in Sheffield, UK). The model structure will be informed by a conceptual model that incorporates local stakeholders’ views and literature findings. Model parameterisation will utilise publicly available data and implement best practices found in previous models. Finally, the model results will provide timely economic evidence for the ongoing update to CG161 [31], while also exploring the current UK guidelines’ performance in terms of broader decisional criteria (e.g., reducing social inequities of health).

## Methods

### Model conceptualisation

A framework for conceptualising public health economic models was followed [41]. The conceptualisation phases were: (A) aligning the framework with the decision-making process; (B) identifying relevant stakeholders; (C) understanding the problem; and (D) developing and justifying the model structure. Additional file 1: Appendix A describes the detailed steps and results of phases (A)-(C), particularly Fig. A1 illustrating the conceptual model.

**Fig. 1:**
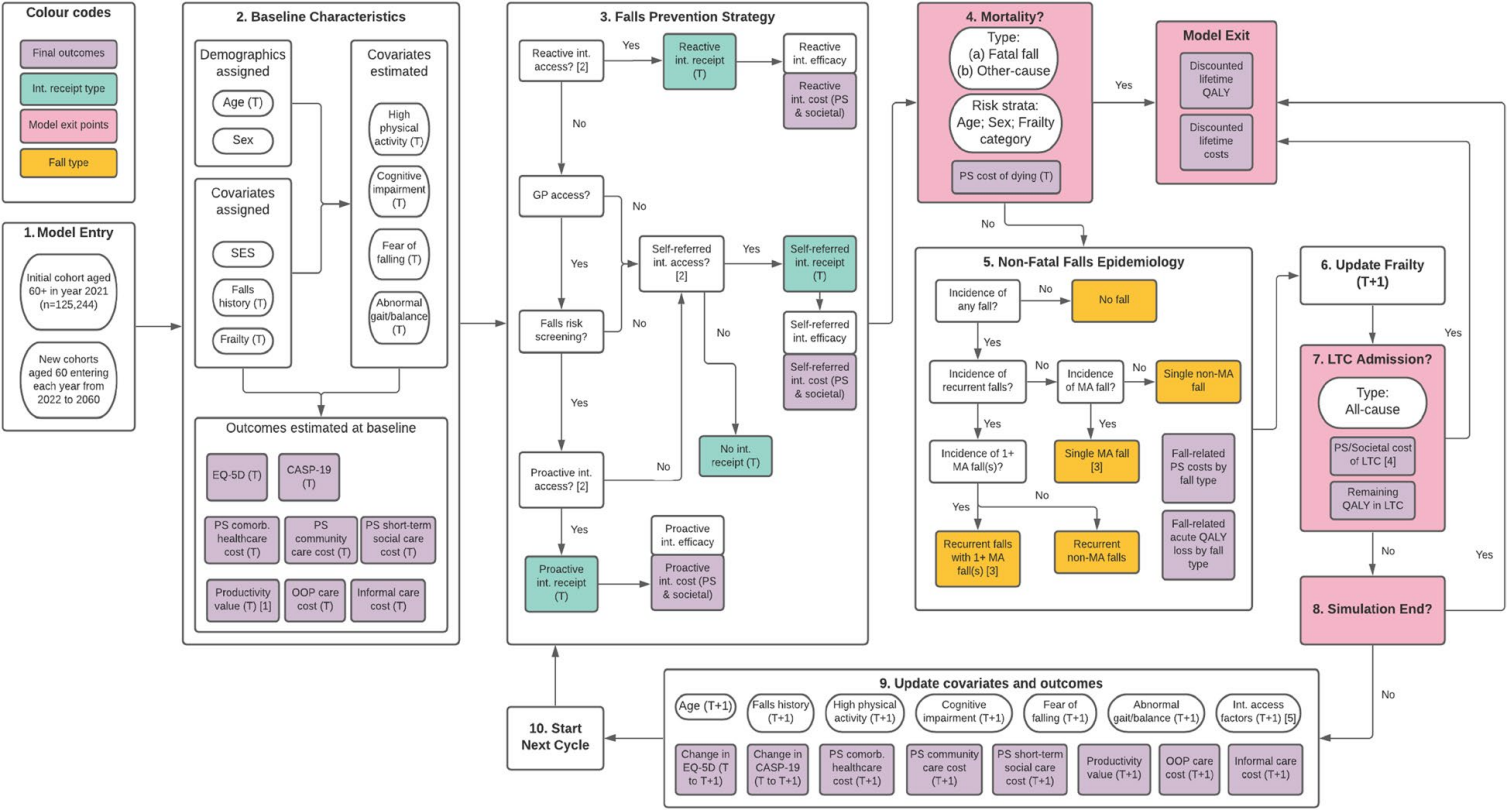
Model representation diagram. *Abbreviation*: CASP-19: control, autonomy, self-realisation and pleasure, 19 items; Comorb.: comorbidity; Int.: intervention; LTC: long-term care; MA fall: fall requiring medication attention; OOP: out-of-pocket; PS: public sector; QALY: quality-adjusted life year; SES: socioeconomic status. *Notes*: [1] Includes paid employment and unpaid work. [2] Intervention access rates are functions of eligibility (determined by covariates such as falls history) and implementation factors (demand and supply capacity); these can be altered by intervention scenarios. [3] For those experiencing recurrent falls with 1 + MA fall(s), the probability for experiencing a second MA fall is applied; MA falls are subdivided into hospitalised and non-hospitalised MA falls. [4] The share of LTC cost incurred by public sector depends on individual’s SES quartile. [5] Probability of GP contact and demand for self-referred intervention are updated longitudinally

Within phase (C), attention was given to four key conceptual themes that present challenges for public health economic modelling [44]: (1) non-health and societal outcomes of falls and falls prevention; (2) heterogeneity and dynamic complexity; (3) behavioural factors and implementation challenges; and (4) issues of equity. In addition, key variables influencing falls epidemiology and key features of current and recommended falls prevention strategies were identified and described. Conceptualisation was conducted in Sheffield, seen here as representative of urban UK local health economies. It involved consultations of local commissioners and clinical professionals, qualitative research with local older persons [42], literature reviews, consultation of modelling experts, and primary data analyses. Phase (D) is reported below.

### Model overview

**Fig. 2:**
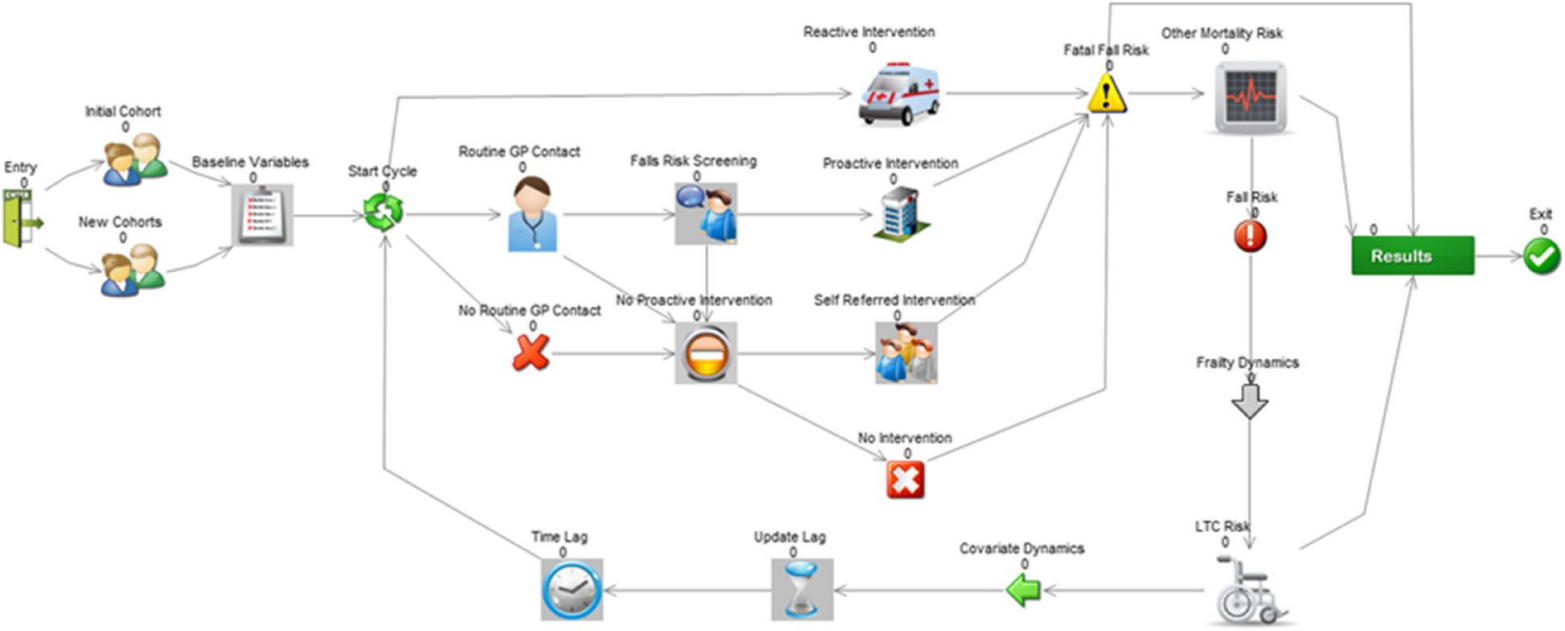
Simul8 model schematic. *Abbreviation*: Int: intervention; LTC: long-term care; MF Int: multifactorial intervention

Detailed information about model parameterisation is described in Additional file 2: Appendix B. As noted, the model was conceptualised in Sheffield to be representative of urban UK local health economies. The model type is discrete individual simulation according to a published model structure taxonomy [45] with annual cycles. The target population was community-dwelling adults aged 60 + . This covers a broader age range than NICE CG161 targeting those aged 65 + and was motivated by local commissioners’ emphasis on early/primary prevention and local older persons’ awareness of falls risk before the age of 65 [42]. Commissioners oversee a geographical jurisdiction rather than a specific cohort; hence, the target population included cohorts who newly turn age 60 during simulation. Figure 1 graphically represents the model including its covariates, falls prevention pathways, fall types, exit points, and final outcomes. Figure 2 shows the model schematic implemented in Simul8.

At entry, individuals are assigned age, sex, socioeconomic status (SES) quartile, falls history in past year, and frailty index score (range 0–100). The latter was used to group individuals into four frailty categories, applying the same cut-off levels available in literature [46]: fit (up to 50^th^ centile of frailty score, equivalent to score range 0–10); mild (> 50^th^ to 85^th^ centile, range > 10 to 23); moderate (> 85^th^ to 97^th^ centile, range > 23 to 37); and severe (> 97^th^ centile, range > 37) (see Section B3.4 in Additional file 2: Appendix B).

Multivariate logistic regressions are then used to estimate individuals’ baseline risks/probabilities of the following covariates: engaging in high physical activity; cognitive impairment; fear of falling; and abnormal gait/balance. Subsequently, the following baseline outcomes are estimated using logistic/linear regression: EQ-5D-3L; control, autonomy, self-realisation and pleasure, 19 items (CASP-19), a social wellbeing measure, rescaled to 0–1 scale; primary and secondary healthcare cost (comprising GP consultations and emergency and elective hospital admissions); community care cost; short-term social care cost; productivity (paid and unpaid work) value; out-of-pocket (OOP) care expenditure; and informal care cost.

Depending on eligibility and implementation factors (supply and demand), individuals then enter one of three falls prevention pathways, if any: reactive; proactive; and self-referred. Eligibility and implementation vary between two scenarios: usual care (UC), representing current practice; and recommended care (RC), representing UK guideline recommendations. Table 1 summarises the eligibility conditions; details on the access conditions are given in Section B5 of Additional file 2: Appendix B. Intervention access gains efficacy and incurs intervention costs (public sector and societal).

**Table 1:** Intervention eligibility conditions by pathway and scenario

Pathway	Usual care (UC)	Recommended care (RC)
Reactive	HAM for hospitalised fallers only (around 28% of MA fallers)	Multifactorial intervention for all MA fallers
Proactive	Multifactorial intervention for high falls risk individuals screened at routine GP contact, ^a^ who are: (i) cognitively intact; (ii) not receiving the reactive intervention that year; (iii) have not previously received the proactive intervention	Multifactorial intervention for high falls risk^b^ individuals screened at routine GP contact: (i) regardless of cognitive status; (ii) not receiving the reactive intervention that year; and (iii) regardless of proactive intervention history^c^
Self-referred	Self-financed exercise intervention for 0.1% of persons in the most privileged SES quartile not receiving reactive/proactive intervention that year	Publicly funded exercise intervention for persons who are not receiving the reactive/proactive intervention that year

*Abbreviation*: *ELSA* English Longitudinal Study of Ageing, *HAM* Home assessment and modification, *MA faller* Faller requiring medical attention, *SES* Socioeconomic status

^a^According to ELSA: around 81% of older persons aged 60 + access routine GP contact each year; under usual care, only around 31% of persons receive falls risk screening at GP contact; around 34% of screened individuals receive the intervention. See Table B26 in Additional file 2: Appendix B for greater detail on access conditions

^b^Assessed using NICE CG161 criteria: high falls risk if had recurrent falls in past year and/or abnormal gait/balance [28]

^c^To keep client flow to that compatible with 7 falls clinics, those with 3 previous re-receipts are excluded

After intervention, individuals face risks of fatal fall or other-cause mortality stratified by age, sex, and frailty category. Those who experience mortality exit and incur cost of dying. At exit, discounted lifetime outcomes are calculated. Others face risks of non-fatal falls. Logistic regressions are used to estimate the risks of: (1) any fall; (2) recurrent falls given any fall; (3a) fall requiring medical attention (MA fall) given single fall; and (3b) MA fall(s) given recurrent falls. These produce five faller types: (i) no fall; (ii) single non-MA fall; (iii) single MA fall; (iv) recurrent non-MA falls; and (v) recurrent falls with 1 + MA fall(s). Individuals in (v) face the risk of experiencing two MA falls. MA fallers face further risk of experiencing a hospitalised, as opposed to non-hospitalised, MA fall(s). Healthcare costs (see Table B43 in Additional file 2: Appendix B for breakdown) and acute quality-adjusted life year (QALY) loss directly attributable to falls are assigned by faller type.

The post-fall frailty progression is then estimated by linear regression. The positive association between fall incidence and frailty progression propagates the secondary or indirect effects of falls, whereby the fall-induced increase in frailty raises comorbidity care costs and worsens outcomes (e.g., EQ-5D-3L) and falls risk in the next cycle. The frailty progression and other covariates are used as independent variables to predict long-term care (LTC) admission. Those admitted to LTC exit after incurring the admission cost (comprised of publicly funded NHS and residential costs and self-funded residential costs; see Table B46 in Additional file 2: Appendix B for breakdown) and being assigned the average remaining QALY in LTC. The model then concludes if the cycle is the final one. If so, all individuals exit, and final outcomes are computed. Otherwise, their covariates and outcomes are updated for the next cycle. This repeats until the final cycle.

### Model parameterisation and validation

The model parameterisation (detailed in Additional file 2: Appendix B) sought to address the methodological challenges arising from the key conceptual themes and falls epidemiology and prevention features included in the conceptual model. Best practices and data sources from previous falls prevention models were also appraised and utilised where appropriate [35, 38].

There were two main data sources for parameterisation, both of them publicly available: (i) the English Longitudinal Study of Ageing (ELSA) Waves 4 and 5 [47]; and (ii) UK-based RCTs of community-based falls prevention obtained via a systematic review and reference searching of previous reviews. Section B2 in Additional file 2: Appendix B provides more detail on the two sources, including the rationale for using ELSA Waves 4–5 rather than the later waves. Both ELSA and the RCTs were assumed generalisable to Sheffield (affirmed by local commissioners) and other UK local health economies. The baseline year was set to 2021 but the model results should be generalisable to later baseline years near 2021.

The parameterisation involved several cross-sectional and longitudinal multivariate regressions, linear and logistic. These regressions sought associative patterns for parameterisation rather than make causal inferences. All statistical analyses were conducted using STATA [48]. All model simulations were run on Simul8 Professional® [49]. The Simul8 Visual Logic codes are available upon request. The parameterised model was subsequently assessed for face, internal, and external validity, and the results are reported in Section B8 of Additional file 2: Appendix B.

### Key methodological solutions

This section summarises the key methodological solutions that were achieved to meet the study aim. They are discussed below under the four key conceptual themes.

#### (1) Capturing non-health outcomes and societal intervention costs

The current model incorporated a wider range of non-health outcomes and societal intervention costs than any previously reviewed model [38]. For example, the model is unique in identifying and valuing unpaid work (e.g., childcare and informal caregiving) in addition to paid employment of older persons to reflect pattern of contribution in this population: 28.0% of older persons aged 60 + engaged in weekly unpaid work according to ELSA versus 17.4% in paid employment (see Section B4.2 in Additional file 2: Appendix B). Importantly, the wide range of outcome/cost incorporation was achieved using publicly available data. The model also balanced the outcomes with their respective intervention costs (e.g., OOP care expenditure with intervention private co-payment), unlike most of the previous models [38]. As shown in Results below, productivity gain and OOP expenditure reduction were outstripped by time opportunity cost and co-payment, respectively, illustrating the importance of balanced incorporation.

#### (2) Considering heterogeneity and dynamic complexity

The model incorporated several variables that captured the heterogeneity and dynamic complexity in geriatric health: e.g., SES quartiles, multivariate frailty index, physical activity level, fear of falling, and cognitive status. A new 52-item frailty index was developed using the ELSA data which covered all major deficit categories included in previous frailty indices in literature [5, 46, 50–53] (see Section B3.4 in Additional file 2: Appendix B). The index captured the continuous and dynamic nature of geriatric health and improved upon the discrete/binary depiction (if at all) in previous models [38]. The feedback loop between falls and frailty propagated the secondary effects of falls which had substantial impact on efficiency and equity. Moreover, the intervention features (i.e., type, cost, efficacy, and implementation level) varied by cognitive status, frailty, falls risk, and intervention history to account for heterogeneity.

#### (3) Considering theories of human behaviour and implementation

Data limitations were substantial for parameterising the behavioural determinants and patterns in geriatric health. Nevertheless, the model parameterised the demand for self-referred exercise at individual-level granularity; those with exercise history were significantly likelier to self-refer in the next cycle, establishing a feedback loop. The probability of accessing the GP was likewise parameterised at the individual-level. The model accounted for three parallel pathways, each with unique eligibility and implementation conditions. It is also capable of incorporating capacity constraints and scenarios with such constraints will be evaluated and reported in a subsequent manuscript.

#### (4) Considering issues of equity

The model incorporated the SES variable as the characteristic of equity relevance. This approach improved upon previous models, few of which incorporated social characteristics of equity relevance [38]. Distributional cost-effectiveness analysis (DCEA) was subsequently conducted (methods are described below) to understand the joint efficiency-equity impact of RC versus UC. In addition, several individual-level events of equity relevance (described below) were tracked which would otherwise be masked when aggregating the individuals’ outcomes [54].

## Model analysis methods

### Societal cost-utility analysis

The primary analysis for the study involved cost-utility analysis (CUA), using the QALY as the health outcome, from the societal perspective and over a 40-year horizon. The ‘quality’ within QALY was measured by EQ-5D-3L estimated from ELSA (see Section B4.1 in Additional file 2: Appendix B for the estimation method). The societal perspective accounted for non-healthcare costs and non-health outcomes. Costs were reported in pounds (£) at year 2021/22 prices. Both costs and health outcomes were discounted at 3.5% annually [55, 56]. For public sector costs, the distinction was made between all-cause costs and directly fall-related costs as recommended by a guideline to falls prevention economic evaluation [57]. As noted, a fall can affect costs not directly related to it by influencing frailty progression.

Productive efficiencies, expressed as cost-effectiveness thresholds, were assumed to be different inside and outside the public sector. A commonly used cost-effectiveness threshold of £30,000 per QALY gained was used to express the health opportunity cost of public sector costs [56], and £60,000 per QALY gained for societal costs as recommended by the Department of Health [58]. Incremental societal costs were converted to their QALY equivalent and added to predicted QALY gains, thus obtaining total *societal* QALY gains. An incremental cost-effectiveness ratio (ICER) was calculated as incremental public sector cost (all-cause or fall-related) per societal QALY gained; an ICER of less than £30,000 per QALY gained was interpreted as being cost-effective.

Efficiency was also reported as net benefits: (i) incremental net monetary benefit (INMB), calculated by translating the societal QALY gained into equivalent monetary amount and subtracting the incremental public sector cost; and (ii) incremental net health benefit (INHB) when conducting DCEA, calculated by translating the incremental public sector cost into the QALY equivalent and adding to the societal QALY gained. INMB/INHB level above zero indicated the net efficiency gain. The person-years of intervention use and user characteristics are presented by pathway.

### Handling uncertainty

Probabilistic sensitivity analysis (PSA) was undertaken to account for second-order uncertainty which arises from uncertainty in parameter point estimates [34, 59]. Parameter distributions used for PSA are described in Section B9.1 of Additional file 2: Appendix B. After verifying that the societal ICER stabilised after around 600 model simulations, 1,000 simulations were run. The PSA outcomes are presented for the primary analysis of 40-year societal CUA, including the jack-knife mean to account for bias in averaging ICERs across simulations [60], the probability of RC being cost-effective versus UC, and the cost-effectiveness acceptability curve (CEAC) depicting the probability of each strategy being cost-effective at different cross-effectiveness thresholds.

The study also reports deterministic outcomes that account for first-order uncertainty which arises from variability in simulated experiences across individuals [59]. This was done by re-running analysis with 20 random number seeds and computing the average outcomes. Due to the computational burden of PSA, only the deterministic outcomes are presented for secondary analyses.

### Individual-level lifetime outcomes

The number of individuals within a single cohort aged 65 at model baseline (*n* = 5,399) (all deceased or admitted to LTC by the final model cycle) who experienced the following equity-relevant events over their lifetime were reported for UC and RC:

- ‘fair health-related innings’ [61], defined as 60% of the median lifetime QALY for this group under UC;
- ‘fair wellbeing-related innings’, which is same as the health-related innings except for quality being measured by CASP-19 rather than EQ-5D-3L
- ‘productive ageing’, meaning participating in paid or unpaid work for at least ten years from age 65;
- ‘catastrophic private expenditure’, which is individuals in the 3^rd^ and 4^th^ SES quartiles accumulating expenditure (OOP care expenditure and intervention co-payment) exceeding 40% of individuals’ capacity to pay [62] (see Section B7.2 in Additional file 2: Appendix B where conjectures are made on the average capacity by quartile); and
- ‘excessive informal caregiver burden’, defined as the accumulated value of informal caregiving exceeding £85,025 which is five times the annual income earned at the national living wage [63].

### Subgroup analysis

Results were presented by the following subgroups: initial cohort aged 60 + at baseline vs. cohorts aged 60 newly entering at non-baseline years; five-year age group at entry; sex; initial frailty category at entry; initial physical activity status; initial cognitive status; initial fear of falling status; initial gait/balance status; and initial falls history type. Results by SES quartile are reported under DCEA.

### Distributional cost-effectiveness analysis

Outcome differences across SES quartiles were deemed ‘unfair’. DCEA was used to jointly consider the efficiency and equity impacts of RC versus UC [64], where equity is defined as reducing the relative or absolute outcome gap across SES quartiles. The outcome of interest is the per-capita societal net health benefit (NHB) that accrues to each subgroup. The equally distributed equivalent (EDE) level of societal NHB is calculated for each intervention strategy using formulae [1] and [2] for relative and absolute inequality aversion, respectively [64]:

1 $${NHB}_{ede}^{Rel}={\left[\frac{1}{n}\sum_{i=1}^{n}{\left[{NHB}_{i}\right]}^{1-\varepsilon }\right]}^{\frac{1}{1-\varepsilon }}$$

2 $${NHB}_{ede}^{Abs}=-\left(\frac{1}{\alpha }\right)\mathrm{log}\left(\frac{1}{n}\sum_{i=1}^{n}{e}^{-\alpha {NHB}_{i}}\right)$$

*NHB*_*i*_ is the per-capita societal NHB for subgroup *i* amongst *n* = 4 SES quartiles. Atkinson index *ε* and Kolm index *α* depict the strength of relative and absolute inequality aversions, respectively, where higher values denote greater aversion. The key metric is the *incremental* EDE NHB (EDE INHB) of RC versus UC: EDE INHB above zero implies RC being preferred over UC based on efficiency *and* equity. Equally important is the ratio or proportion of EDE INHB relative to the incremental NHB when inequality aversion is *not* considered, referred to as incremental no-aversion benefit (INAB). If both EDE INHB and INAB are above zero and EDE INHB is less than INAB, there is an equity-efficiency trade-off in implementing RC over UC, even though RC would still be preferred. If both are above zero and EDE INHB is greater than INAB, then RC improves both efficiency and equity versus UC.

### Scenario analysis

The following scenarios were explored as suggested by the conceptual model:

- Time horizons of 5, 10, 15, 20 and 30 years
- Discount rates of 0% and 6%
- Removing the falls-frailty feedback loop: the categorical falls incidence variable was removed as an explanatory variable in estimating the longitudinal frailty progression rate. This removed the secondary effects of falls on outcomes via frailty progression.
- Frailty reduction: (i) frailty levels at model entry reduced by 20%; (ii) frailty progression rate between cycles reduced by 20%.
- Higher life expectancy: other-cause mortality risks across all subgroups (age, sex, and frailty category) were reduced by 20%.
- Reduction in other-cause mortality risk gap across frailty categories: the mortality hazard ratios delineated by frailty category (relative to the fit category) were reduced by 20%; this did not alter the average mortality risk but its gradient across frailty.

Note that scenarios (d), (e), and (f) explore the complementarity of RC with broader public health strategies that successfully improve geriatric health.

## Results

### Societal cost-utility analysis

Table 2 shows the PSA outcomes of 40-year societal CUA. Relative to UC, RC reduced all-cause costs by £123.5 m and fall-related costs by £102.4 m, gained 18,946 QALYs, and incurred £396.7 m additional intervention cost. RC generated £39.1 m in productivity gain (£17.0 m via paid employment increase and £22.1 m via unpaid work increase) but this was outstripped by £41.2 m increase in intervention time opportunity cost (TOC). There was hence a net productivity loss of £2.1 m. Likewise, the reduction in OOP care expenditure of £44.8 m was outstripped by a private co-payment increase of £66.6 m. By contrast, the reduction in informal caregiving cost of £139.2 m was greater than the increase in caregiver intervention TOC. The overall societal gain was positive, equivalent to 1,068 QALYs. The jack-knife mean of societal ICER was £14,067 (95% uncertainty interval (UI): £12,011—£15,923) and £15,149 (95% UI: £13,193—£17,006) per QALY gained when considering all-cause costs and only fall-related costs, respectively. These amounted to INMBs of £327.3 m and £261.7 m, respectively, at the cost-effectiveness threshold of £30,000 per QALY gained.

**Table 2:** Probabilistic outcomes of 40-year societal cost-utility analysis

*N* = 385,192	**Usual care**	**Recommended care**	**Incremental**
**Public sector costs**^a^	Mean (SE)	Mean (SE)	Mean (SE)
* (1) All-cause public sector costs*^b^	£10,060,099,947 (£99,415,713)	£9,936,609,337 (£98,628,205)	-£123,490,610 (£5,909,603)
* (2) Fall-related healthcare costs*	£663,114,733 (£28,095,762)	£560,737,568 (£23,700,572)	-£102,377,165 (£5,504,016)
*Public sector intervention costs*	£33,992,444 (£2,186,958)	£430,663,194 (£8,281,037)	£396,670,750 (£7,888,734)
QALY	2,091,707 (10,224)	2,110,653 (10,238)	18,946 (499)
**Societal outcomes**			
Productivity			
*Productivity value*^c^	£12,828,548,949 (£738,143,648)	£12,867,696,042 (£740,545,423)	£39,147,094 (£4,280,003)
*Intervention TOC*	£1,198,057 (£143,984)	£42,417,182 (£2,592,854)	£41,219,125 (£2,594,679)
Net productivity gain			-£2,072,031 (£1,327,648)
Personal finance			
*OOP care expenditure*^d^	£2,523,459,511 (£111,570,114)	£2,478,690,212 (£109,989,355)	-£44,769,299 (£2,914,804)
*Intervention private co-payment*	£8,855,861 (£606,367)	£75,446,429 (£4,512,590)	£66,590,569 (£4,554,691)
Net personal finance cost			£21,821,270 (£1,543,638)
Informal caregiver burden			
*Informal caregiving cost*^e^	£14,884,116,295 (£902,879,471)	£14,744,953,134 (£894,187,914)	-£139,163,161 (£10,486,904)
*Intervention caregiver TOC*	£356,678 (£22,893)	£51,535,375 (£3,439,769)	£51,178,696 (£3,439,868)
Net informal caregiver cost			-£87,984,465 (£8,660,865)
**Societal gain, QALY equivalent**			1,068 QALYs
			**Jack-knife mean (95% UI)**^e^
**Societal ICER using (1)**^f^			£14,067 per QALY gained (£12,011—£15,923)
**Societal ICER using (2)**			£15,149 per QALY gained (£13,193—£17,006)
			**Mean (95% UI)**^g^
**Societal INMB using (1)**			£327,260,886 (£286,031,713—£368,490,059)
**Societal INMB using (2)**			£295,215,304 (£261,690,664—£328,739,943)

*Abbreviation*: *ICER* Incremental cost-effectiveness ratio, *INMB* Incremental net monetary benefit, *OOP* Out-of-pocket, *QALY* Quality-adjusted life year, *SE* Standard error, *TOC* Time opportunity cost, *UI* Uncertainty interval

^a^All outcomes were averaged across 20 model trial runs with different random number seeds

^b^Includes costs of fall-related primary and secondary healthcare, comorbidity primary and secondary healthcare, cost of dying, community healthcare, short-term social care, all-cause long-term care

^c^Includes values of paid and unpaid employment

^d^Includes OOP care expenditure and privately incurred long-term care cost

^e^ICERs are computed using the jack-knife method to avoid bias associated with ratios [60]

^f^The jack-knife means for public sector ICERs are £15,367 per QALY gained using [1] and £15,829 using [2]

^g^The incremental net monetary benefits were estimated from the incremental outcomes rather than from jack-knife method using the cost-effectiveness threshold of £30,000 per QALY gained

**Fig. 3:**
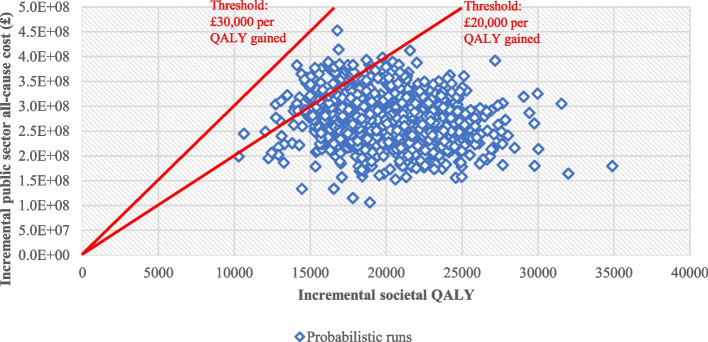
Scatter plot of probabilistic sensitivity analysis result for societal cost-utility analysis over 40-year horizon. *Abbreviation*: QALY: quality-adjusted life year

Figure 3 shows the scatter plot of the incremental societal QALY and incremental public sector all-cause cost of RC versus UC based on 1,000 probabilistic runs. The scatter points were placed in the north-east quadrant of the incremental QALY-cost graph and lay below the line for the £30,000 per QALY gained cost-effectiveness threshold, while 93.4% of runs lay below that for the £20,000 per QALY gained threshold. Figure C1 in Additional file 3: Appendix C shows the CEAC for RC versus UC. The probability of RC being cost-effective versus UC crossed 50% at the threshold of £13,700 per QALY gained.

The results were similar for deterministic outcomes that accounted only for first-order uncertainty. The societal ICER considering all-cause costs was £12,877 per QALY gained, which was lower than the jack-knife mean but lay within the uncertainty interval. The corresponding INMB was £358.7 m at the £30,000 per QALY gained threshold. The person-years of any fall declined by 6.4% in RC versus UC.

Table C1 in Additional file 3: Appendix C shows the characteristics of intervention users by pathway. The total person-years of intervention use across all pathways increased by over tenfold from 159,169 under UC to 1,710,424 under RC; the annual average usage increased from 3,979 to 42,761. The self-referred pathway experienced the largest proportional expansion, increasing 272-fold from 47 users per year to 12,793. The proactive pathway use increased around 18-fold from 1,517 users per year to 26,928. Under RC, there were 21,131 annual multidisciplinary falls clinic clients.

### Individual-level lifetime outcomes

Table 3 compares the individual-level lifetime outcomes of UC and RC for the cohort aged 65 at baseline. The numbers of persons achieving health- and wellbeing-related ‘fair innings’ in RC versus UC increased by 0.8% and 0.5%, respectively. The number achieving productive ageing saw the largest change, increasing by 2.7%. The number in the 3^rd^ and 4^th^ SES quartiles experiencing catastrophic private expenditure *increased* by 1.8% but declined by 0.9% when intervention private co-payments were excluded; the net increase in the catastrophic expenditure incidence can hence be attributed to co-payments. The incidence of excessive informal caregiver burden declined slightly by 0.8%.

**Table 3:** Comparison of individual-level lifetime outcomes for the cohort aged 65 at baseline (*n* = 5,399) under 40-year societal cost-utility analysis

**Outcomes**^a,b^	**UC**	**RC**	**Incremental (% change)**
Persons achieving ‘fair health-related innings’	4,415	4,450	35 (0.8)
Persons achieving ‘fair wellbeing-related innings’	4,482	4,504	22 (0.5)
Persons achieving ‘productive ageing’	622	639	17 (2.7)
Catastrophic private expenditure (CPE)			
* (1) Persons experiencing CPE*	553	563	10 (1.8)
* (2) Excluding intervention private co-payment from (1)*	550	545	-5 (-0.9)
Excessive informal care burden			
* (3) Persons experiencing excessive informal care burden*	1,753	1,738	-14 (-0.8)
* (4) Excluding intervention TOC from (3)*	1,753	1,729	-24 (-1.4)

*Abbreviation*: *LTC* Long-term care, *MA fall* Fall requiring medical attention, *OOP* Out-of-pocket, *QALY* Quality-adjusted life year, *RC* Recommended care, *SES* Socioeconomic status, *TOC* Time opportunity cost, *UC* Usual care

^a^All outcomes were averaged across 20 model trial runs with different random number seeds

^b^See ‘Individual-level lifetime outcomes’ under Methods for definitions of the outcomes

### Subgroup analysis

Table C2 in Additional file 3: Appendix C shows the subgroup outcomes for the initial cohort aged 60 + at baseline and for the new cohorts entering as 60-year-olds at subsequent cycles. The societal ICERs considering all-cause costs were higher for the new cohorts (£13,918 versus £11,619 per QALY gained), but these remained well below the £30,000 per QALY gained threshold. The per-capita societal gain was higher for the new cohorts (1,199 versus 181 QALYs), particularly due to the marked reduction in informal caregiving costs. Overall, accounting for the needs of newly eligible older persons over the intervention horizon affects intervention use but has no major impact on the cost-effectiveness of RC versus UC.

Table C3 shows the subgroup outcomes by five-year age group at model entry. For the subgroup aged 60–64 at entry, results for only the initial cohort members were evaluated since later cohorts (aged 60 at entry) spent varying durations in the model. The societal ICERs for RC versus UC were below the £30,000 per QALY gained threshold except for those aged 90 + when considering fall-related costs only (£31,681 per QALY gained). The cost-effectiveness improved with younger age at entry. The net societal gains were concentrated among those aged 60–64. Those aged 70 + incurred net societal loss, while those aged 85 + experienced net productivity loss, net private expenditure increase, and net informal caregiving cost increase.

Table C4 shows the subgroup outcomes by sex. There was a marked difference in the cost-effectiveness outcomes across sex, with the societal ICER of RC versus UC (including all-cause costs) for men nearly double those for women (£18,641 vs. £9,659 per QALY gained). RC was hence particularly cost-effective for women and raises female societal contributions. Table C5 shows the subgroup outcomes by initial frailty category. RC was cost-effective versus UC for all categories. The societal ICERs were lowest for the moderate category and highest for the severe. The societal gains were concentrated among the fit and net negative for the severe. The latter experienced a net increase in informal caregiving cost due to the high prevalence of cognitively impaired persons whose informal caregivers incurred time opportunity costs in attending interventions.

**Table 4:** Equally distributed equivalent net health benefits by SES quartile for 40-year societal cost-utility analysis

Inequality aversion type/strength	Usual care (UC)	Recommended care (RC)	Incremental
No inequality aversion	Per-capita societal mean NHB:^a^^,b^		INAB:
	4.1774	4.2081	0.0307
(1) Relative inequality	Per-capita societal mean EDE NHB:		EDE INHB:
Atkinson ε = 0	4.1774	4.2081	0.0307
Atkinson ε = 3	3.9184	3.9521	0.0337
Atkinson ε = 5	3.7365	3.7722	0.0357
Atkinson ε = 11	3.3627	3.3994	0.0367
Atkinson ε = 15	3.2445	3.2804	0.0359
Atkinson ε = 20	3.1630	3.1981	0.0351
Atkinson ε = 25	3.1155	3.1501	0.0346
Atkinson ε = 30	3.0846	3.1189	0.0343
(2) Absolute inequality	Per-capita societal mean EDE NHB:		EDE INHB:
Kolm α = 0.025	4.1698	4.2005	0.0307
Kolm α = 0.050	4.1622	4.1929	0.0307
Kolm α = 0.150	4.1311	4.1618	0.0308
Kolm α = 0.250	4.0991	4.1300	0.0309
Kolm α = 0.400	4.0501	4.0810	0.0310
Kolm α = 0.500	4.0167	4.0478	0.0311

*Abbreviation*: *EDE* Equally distributed equivalent, *INAB* Incremental no-aversion benefit, *INHB* Incremental net health benefit, *NHB* Net health benefit, *QALY* Quality-adjusted life year, *SES* Socioeconomic status

^a^All outcomes were averaged across 20 model trial runs with different random number seeds

^b^The societal NHB incorporates QALY gain and QALY-equivalent net societal gain minus public sector opportunity costs (translated to QALY-equivalent using cost-effectiveness threshold of £30,000 per QALY gained) for each SES quartile. To account for the differing sizes of SES subgroups, *per-capita* societal NHBs were computed for each subgroup. The unweighted *mean* of the per-capita NHBs was then computed, effectively treating the subgroups as of equal size

**Table 5:** SES-delineated per-capita EDE INHBs for RC versus UC under frailty reduction scenarios

	**RC versus UC**		
**Per-capita societal INHB**^a^	**(i) Initial frailty reduction**	**(ii) Frailty progression rate reduction**	**Base case**
(1) Incremental no-aversion mean (INAB)	0.0276	0.0225	0.0307
(2) EDE INHB for Atkinson ε = 30	0.0312	0.0241	0.0343
*Proportion of (2) relative to (1)*	*1.1304*	*1.0684*	*1.1169*
(3) EDE INHB for Kolm α = 0.5	0.0281	0.0225	0.0311
*Proportion of (3) relative to (1)*	*1.0181*	*1.0004*	*1.0121*

*Abbreviation*: *EDE* Equally distributed equivalent, *INHB* Incremental net health benefit, *QALY* Quality-adjusted life year, *RC* Recommended care, *SES* Socioeconomic status, *UC* Usual care

^a^All outcomes were averaged across 20 model trial runs with different random number seeds

The results of further subgroup analyses are presented in Additional file 3: Appendix C: Table C6 for outcomes by initial physical activity status; Table C7 initial cognitive status; Table C8 initial fear of falling status; Table C9 initial gait/balance status; and Table C10 initial falls history type. The societal ICERs consistently remained below the £30,000 per QALY gained threshold. The ICERs were lower for the subgroup with initial gait/balance impairment and the subgroup with recurrent falls history compared to their counterparts. Hence, including these subgroups in the target population would improve cost-effectiveness. The cognitively impaired subgroup enjoyed comparable per-capita QALY gain compared to the cognitively intact (0.051 versus 0.047), but the societal gains were concentrated among the intact.

### Distributional cost-effectiveness analysis

**Fig. 4:**
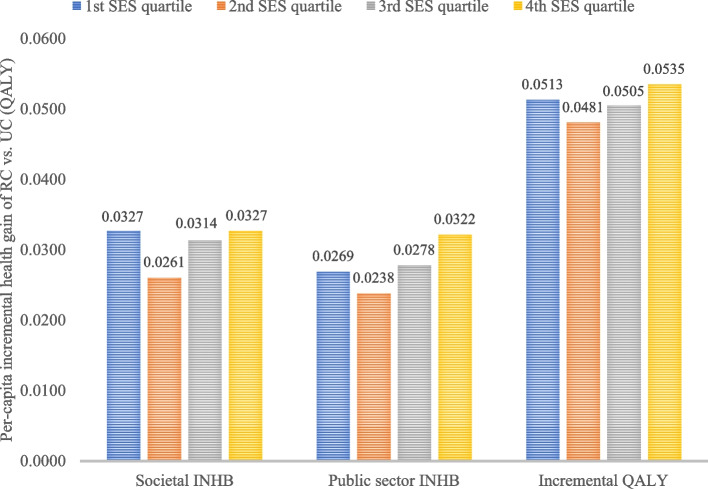
Per-capita incremental health gain by SES quartile of RC versus UC considering all-cause costs and cost-effectiveness threshold of £30,000 per QALY gained. *Abbreviation*: INHB: incremental net health benefit; QALY: quality-adjusted life year; RC: recommended care; SES: socioeconomic status; UC: usual care. *Note*: Outcomes were averaged across 20 model runs with different random number seeds

Table C11 presents the CUA outcomes of RC versus UC by SES quartile. The trends in the societal ICERs across the quartiles were non-linear, with the lowest ICER (considering all-cause costs) for the most deprived 4^th^ quartile (£11,844 per QALY gained) and highest for the 2^nd^ quartile (£14,450 per QALY gained). Figure 4 shows the per-capita incremental health gain metrics delineated by SES quartile. The 4^th^ quartile enjoyed the most favourable outcome for public sector INHB and incremental QALY but had near identical societal INHB as the 1^st^ quartile.

Table 4 reports the results of DCEA delineated by SES quartile. It shows the EDE levels of NHB for UC and RC under various relative and absolute inequality aversion indices (Atkinson ε and Kolm α, respectively) and where health differences across SES quartiles are deemed unfair. The EDE NHBs for both UC and RC declined as the aversion parameters increased. The positive INAB of 0.0307 shows that RC is cost-effective versus UC at the £30,000 per QALY gained threshold. Moreover, the EDE INHBs of RC versus UC remain higher than the INAB across the ranges of relative and absolute inequality aversion parameters, showing that RC also improved equity versus UC. Figures C2(a) and (b) show the EDE INHBs across the ranges of relative and absolute inequality aversion parameters.

### Scenario analysis

#### Changes to time horizon

**Fig. 5:**
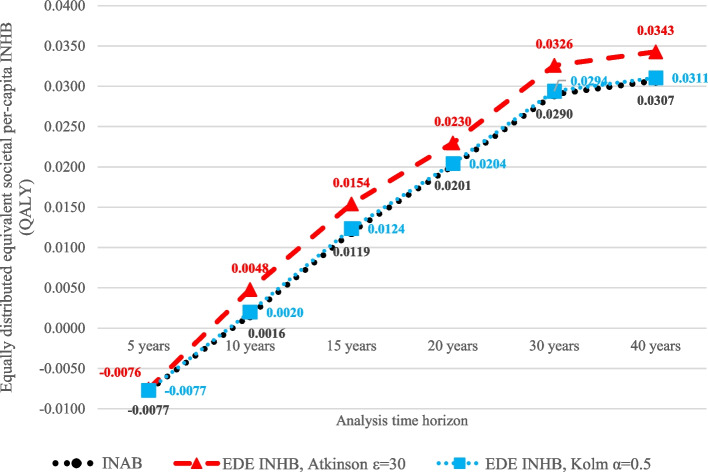
SES-delineated equity analysis: per-capita societal EDE INHBs by inequality aversion level and time horizon. *Abbreviation*: EDE: equally distributed equivalent; INAB: incremental no-aversion benefit; INHB: incremental net health benefit; QALY: quality-adjusted life year; SES: socioeconomic status. *Note*: Outcomes were averaged across 20 model runs with different random number seeds

Table C12 in Additional file 3: Appendix C presents the CUA outcomes of RC versus UC under 5-, 10-, 15-, 20- and 30-year horizons. There is a non-linear decline in the ICERs under both societal and public sector perspectives as the horizon increases. There were net societal losses under 5- and 10-year horizons such that the societal ICERs were higher than the public sector ICERs. The threshold of £30,000 per QALY gained is crossed between 5- and 10-year horizons (considering all-cause costs). Figure 5 shows the DCEA outcomes across time horizons: societal EDE INHBs per capita under (i) no inequality aversion (i.e., INAB), (ii) high relative inequality aversion (Atkinson ε = 30), and (iii) high absolute inequality aversion (Kolm α = 0.5). The EDE INHBs under (ii) and (iii) were consistently above the INAB of (i), meaning that RC improved SES-delineated equity versus UC under all time horizons.

#### Changes to discount rates

Table C13 presents the 40-year CUA outcomes under the discount rates of 0% and 6% for health and cost outcomes. The rate variations had large impacts on the present values of costs and outcomes but modest impacts on the societal ICERs. For 0% rates, the EDE INHBs of RC versus UC were 0.0799 and 0.0730 under Atkinson ε = 30 and Kolm α = 0.5, respectively. These were higher than the INAB of 0.0710, implying joint equity-efficiency improvements. The corresponding figures for 6% rates were 0.0189, 0.0172 and 0.0170, again implying joint improvements.

#### Removing the falls-frailty feedback loop

**Fig. 6:**
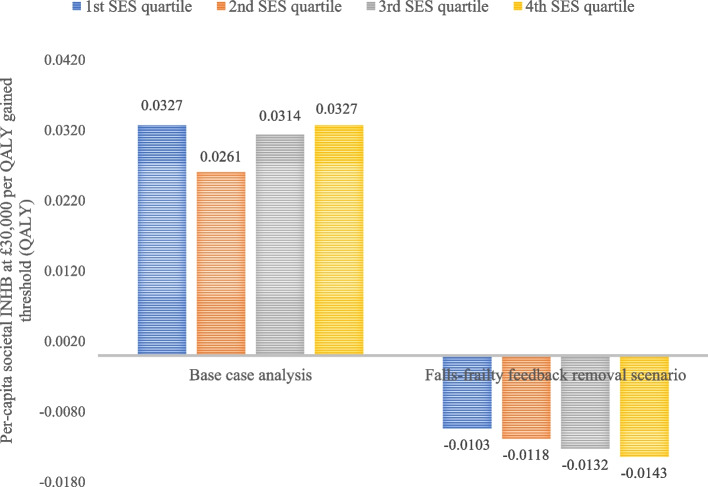
Per-capita societal INHBs for all-cause care costs by SES quartile in base case and in falls-frailty feedback removal scenario. *Abbreviation*: INHB: incremental net health benefit; QALY: quality-adjusted life year; SES: socioeconomic status. *Note*: Outcomes were averaged across 20 model runs with different random number seeds

Table C14 shows the 40-year CUA outcomes after removing the falls-frailty feedback loop. The impact was substantial, with societal and public sector ICERs increasing above the £30,000 per QALY gained threshold. The QALY gain saw the most significant decline from 19,570 gain under the base case to 6,895. The societal outcomes saw a shift from net gain of 1,380 QALYs to net loss of 1,587. Figure 6 compares the per-capita societal INHBs delineated by SES quartile in the base case and this scenario. The U-shaped SES gradient disappears under the latter and the 4^th^ quartile now derives the lowest INHB. The societal EDE INHBs were -0.015 and -0.013 under Atkinson ε = 30 and Kolm α = 0.5, respectively, which were lower than the INAB of -0.012. RC worsened SES-delineated health inequity versus UC.

#### Frailty reduction

Table C15 shows the 40-year CUA outcomes under scenarios of 20% reduction in: (i) initial frailty levels; and (ii) the annual rate of frailty progression. Under both, the societal and public sector ICERs increased relative to the base case but remained below the £30,000 per QALY gained threshold. Table 5 shows the SES-delineated per-capita societal EDE INHBs of RC versus UC under the two scenarios. Comparing the proportions of EDE INHB relative to INAB, Table 5 shows that scenario (i) magnified the extent of equity improvement of RC versus UC since the proportion rose from 1.1169 in the base case to 1.1304 in terms of relative inequality aversion and from 1.0121 to 1.0181 in terms of absolute aversion. The opposite was true for scenario (ii), even though the joint equity-efficiency improvements of RC versus UC were maintained.

#### Higher life expectancy

Table C16 shows the 40-year CUA outcomes when other-cause mortality risks were reduced by 20%. Compared to the base case, the societal and public sector ICERs were all lower due to the higher total QALY gains and public sector cost savings, though the net societal gain was lower. The per-capita societal EDE INHBs for the scenario were 0.0376 and 0.0336 under Atkinson ε = 30 and Kolm α = 0.5, respectively, which were higher than the INAB of 0.0328. The proportions of EDE INHBs relative to INAB were 1.1483 and 1.0249 respectively, which were higher than those under the base case (1.1169 and 1.0121). Hence, the higher life expectancy magnified the equity improvement of RC versus UC.

#### Reduction in other-cause mortality risk gap across frailty categories

Table C17 shows the 40-year CUA outcomes from the scenario with 20% reduction in the mortality hazard ratios across frailty categories. There were modest increases in the societal ICERs compared to the base case, but RC remained cost-effective versus UC. The per-capita societal EDE INHBs for the scenario were 0.0327 and 0.0300 under Atkinson ε = 30 and Kolm α = 0.5, respectively, while the INAB was 0.0294. The proportions of EDE INHBs relative to the INAB were 1.1115 and 1.0192, respectively, compared to 1.1169 and 1.0121, respectively, under the base case. Hence, the scenario magnified the equity improvement in terms of absolute, but not relative, SES-delineated inequality.

## Discussion

This study explored the efficiency and equity of RC versus UC, representing UK guideline recommendations and current local practice, respectively. Under the 40-year societal CUA, RC had a 100% probability of being cost-effective versus UC at the cost-effectiveness threshold of £30,000 per QALY gained and 93.4% at £20,000 per QALY gained. It increased productivity (particularly in the form of unpaid work including childcare and caregiving) and reduced private care expenditure and informal caregiving cost, but the productivity gain and the private expenditure reduction were outstripped by increases in intervention time opportunity costs and co-payments, respectively. There was no equity-efficiency trade-off in terms of relative and absolute inequality delineated by SES quartile. However, gains in terms of individual-level lifetime outcomes were small. Alternative scenarios showed that falls prevention is highly integrative with other geriatric public health interventions that reduce baseline and contemporaneous frailty and improve life expectancy.

Comparison of results to those of previous models for cross-validation is difficult since only one previous model evaluated a falls prevention programme encompassing the reactive, proactive, and self-referred pathways [65]. Eldridge and colleagues estimated that the number of fallers declined by 6.5% under the programme versus usual care and by 11.3% if the falls risk screening rate reached 100% [65]. In comparison, the current model (with 100% screening rate under RC) estimated 6.4% decline in person-years of any fall. The difference could be attributed to the higher efficacy estimate in the Eldridge model [65]. A model developed by Johansson and colleagues to evaluate a combination of multifactorial and environmental interventions shared a similar analytic approach to the current study in incorporating comorbidity care costs and productivity value [66]. The Johansson model found that the combination dominated usual care in the base case but produced an ICER of around £17,000 per QALY gained when costs of added life-years in the form of net productivity loss were included in scenario analysis. By contrast, the current model did not find such large-scale differences in ICERs between public sector and societal outcomes. The difference could be attributed to how the societal cost of added life-years was calculated in the Johansson model, namely as productivity net consumption [66], as opposed to productivity net of intervention time opportunity cost in this model.

The study methods and results contribute to the growing awareness and practice of considering equity and priority setting objectives alongside efficiency [43, 67]. The DCEA jointly assessed efficiency and equity defined in terms of the unequal distribution of the per-capita societal INHB across SES quartiles and the degree of aversion towards the inequality level [64]. Several caveats concerning the current DCEA approach can be noted. First, the DCEA was conducted only on deterministic outcomes that accounted for first-order uncertainty, rather than on the probabilistic outcomes as recommended [64]. But the computational burden of generating probabilistic outcomes for each inequality aversion type and level was deemed excessive. Second, the approach did not explore alternative distributions of intervention opportunity costs as done previously [64]. Third, the outcomes were not adjusted for the impacts of variables *without* equity relevance; if, for example, the INHB differential across sex is deemed fair, then the analysis should adjust for the impact of sex on INHB estimated from a multivariate Eq. [64]. The conceptual model accounted for variables of *key* equity relevance [42], and not those of *no* relevance. In this scenario, the use of unadjusted INHBs is likely justified.

The model tracked further metrics of equity and priority setting relevance. The first set of such metrics were the individual-level lifetime outcomes, including the numbers of individuals experiencing health- and wellbeing-related ‘fair innings’ [61]. This granularity avoided aggregating individual-level outcomes to subgroup- and population-level ones (e.g., total QALY gain) which violate the principle of prioritising individuals’ capabilities [54, 68, 69]. It is thus significant that the changes in individual-level outcomes were muted relative to the aggregated outcomes (e.g., 100% of being cost-effective at the £30,000 per QALY gained threshold). Further work is warranted on how the individual-level metrics, particularly the threshold definitions (e.g., 10 years of paid/unpaid work for ‘productive ageing’), are chosen for each evaluation context. Another outcome with a potential ethical implication was the reduced cost-effectiveness of falls prevention for older subgroups. Rather than curtail provision for the oldest, the inclusion of the younger old (i.e., 60–64 years, currently excluded from CG161 [28]) in the target population should be encouraged to improve the overall cost-effectiveness.

The key study aim was to achieve solutions to a broad range of methodological challenges inherent in geriatric public health economic modelling, and the main solutions were highlighted above in Methods under the four conceptual themes. It is also worth discussing, based on the model results, how these themes intersect, particularly between the first three themes and the issues of equity addressed in the last theme [38]. First, the inclusion of societal outcomes worsened the unequal distribution of intervention benefits across SES quartiles: the per-capita net societal gain was lowest for the 4^th^ (0.0005 QALYs) and highest for the 1^st^ (0.0058 QALYs). The assessment of non-health outcomes, highlighted as a key priority setting challenge [43, 70], must therefore be complemented by explicit consideration of social inequities of health. Second, there was a close relationship between dynamic complexity and equity: the scenario that removed the falls-frailty feedback loop eliminated the SES-delineated equity gain (as well as efficiency) of RC versus UC. Third, the increase in the number of individuals experiencing catastrophic private expenditures could be attributed to the private co-payments incurred at intervention access. This suggests RC should be supplemented by policies reducing the co-payments of socially vulnerable groups. It also raises a methodological caveat in that the model likely overestimated the intervention demand pattern of these groups when facing high co-payments. This highlights the need for greater understanding and data on geriatric health behaviours.

A key strength of decision modelling is its capacity to evaluate all scenarios and strategies of interest. Further falls prevention strategies that are potential alternatives to RC will be evaluated in future work. This manuscript reported on the outcomes of several scenarios that potentially affect the performance of RC versus UC. Specifically, the scenarios of frailty reduction, life expectancy extension, and mortality risk gap reduction approximate the impacts of broader geriatric and earlier life-course public health strategies that alter the epidemiological characteristics of the target population for falls prevention. The results generally showed that the performance of RC versus UC is not substantially affected by these epidemiological changes. This finding is important given the current policy interest in integrated care [71–73]: falls prevention makes an independent contribution to geriatric health promotion even when contextualised by other highly successful health policies.

This study has several limitations/caveats beyond those already discussed. First, the model does not incorporate the long-term impact of the COVID-19 pandemic on the intervention context; the capacity to organise group exercise sessions, for example, may have been permanently impacted, in which case the modelled intervention features and implementation levels would be inaccurate. That said, there is no indication in the CG161 update scope that the guideline will account for the pandemic impact [31]. Second, several assumptions were involved in modelling the intervention features of UC (conceptualised with Sheffield stakeholders and parameterised using ELSA). However, deterministic sensitivity analyses (see Sect. 6.2.3 in JK’s thesis [74]) showed that none of the parameters governing UC intervention features was in the list of top 20 variables with the largest impact on ICER. This suggests that the current results are robust to local variations in usual care. Third, the model parameterisation relied on ELSA Waves 4–5 rather than the most recent waves, although there were clear reasons for choosing these waves (see Section B2 in Additional file 2: Appendix B). Moreover, there were methodological issues in using the falls data from ELSA, including recall bias. Data from a falls-specific study using prospective, high-frequency falls recording would have been preferable [75]; but it is unlikely that such study would have included the wide range of variables in ELSA (e.g., paid/unpaid work status). Fourth, the model assumed that SES remained static from baseline, rather than dynamic due to events such as retirement and loss of spouse. There was also no spatial dimension to intervention provision and priority setting; by contrast, real-life strategies targeting health/healthcare inequity reduction are often geographically defined [42]. Finally, RC generated unrealistic intervention utilisation rates such as the annual client flow of around 21,000 for the multidisciplinary falls clinics. Further research should evaluate scenarios that incorporate more realistic capacity constraints.

## Conclusion

The recommendations of UK guidelines on community-based falls prevention appear cost-effective versus current practice at the local health economy level. It also appears to reduce inequitable health economic outcomes across SES quartiles. The gains in individual-level lifetime outcomes were modest, and age-based differences in health and societal benefits should motivate the coverage of a wide geriatric age range. Key methodological advances were made in conceptualising and operationalising the current model which improved its structural validity and credibility. The advances, such as the balanced incorporation of societal outcomes and the conduct of DCEA, are also relevant to further geriatric and non-geriatric public health areas. This study hence serves as an important case study in public health economic modelling.

## Supplementary Information

**Additional file 1: Appendix A.** Model conceptualisation.

**Additional file 2: Appendix B.** Model parameterisation.

**Additional file 3: Appendix C.** Model analysis results.

## Data Availability

The documents informing the model conceptualisation, the Simul8 model file, and the model outputs generated by the current study are available from the corresponding author on reasonable request. The English Longitudinal Study of Ageing (ELSA), the main source of data for model parameterisation, can be accessed from the UK Data Service: https://beta.ukdataservice.ac.uk/datacatalogue/series/series?id=200011. Additional file 2: Appendix B contains information on further data sources (all publicly available) used for parameterisation.

## Abbreviations

Aged 65+: Aged 65 and over
CASP-19: Control, autonomy, self-realisation and pleasure, 19 items
CEAC: Cost-effectiveness acceptability curve
CG161: Clinical guideline 161
CUA: Cost-utility analysis
DCEA: Distributional cost-effectiveness analysis
EDE: Equally distributed equivalent
ELSA: The English Longitudinal Study of Ageing
HAM: Home assessment and modification
ICER: Incremental cost-effectiveness ratio
INAB: Incremental no-aversion benefit
INHB: Incremental net health benefit
INMB: Incremental net monetary benefit
LTC: Long-term care
MA fall: Fall requiring medication attention
NHB: Net health benefit
NICE: The National Institute for Health and Care Excellence
OOP: Out-of-pocket
PSA: Probabilistic sensitivity analysis
QALY: Quality-adjusted life year
RC: Recommended care
RCT: Randomised controlled trial
SES: Socioeconomic status
TOC: Time opportunity cost
UC: Usual care
UI: Uncertainty interval
