# Supplementary material for: Economic model of community-based falls prevention: seeking methodological solutions in evaluating the efficiency and equity of UK guideline recommendations
Source: BMC Geriatr. 2023 Mar 30;23:187. doi: 10.1186/s12877-023-03916-z (PMC10061399; doi:10.1186/s12877-023-03916-z)
Supplement: Supplementary file 1 — Additional file 1: Appendix A. Model conceptualisation. [file 12877_2023_3916_MOESM1_ESM.docx]

# Economic model of community-based falls prevention: seeking methodological solutions in evaluating the efficiency and equity of UK guideline recommendations

**Appendix A: Model conceptualisation**

**Authors:**

Dr Joseph Kwon,^1*^ [joseph.kwon@phc.ox.ac.uk](mailto:joseph.kwon@phc.ox.ac.uk); ORCID 0000-0002-2860-7280

Dr Hazel Squires,^2^ [h.squires@sheffield.ac.uk](mailto:h.squires@sheffield.ac.uk); ORCID 0000-0001-8467-0471

Professor Tracey Young,^2^ [t.a.young@sheffield.ac.uk](mailto:t.a.young@sheffield.ac.uk); ORCID 0000-0002-0754-7223

^1^ Nuffield Department of Primary Care Health Sciences, University of Oxford, Radcliffe Primary Care Building, Radcliffe Observatory Quarter, Woodstock Road, Oxford, England, OX2 6GG

^2^ School of Health and Related Research, University of Sheffield, Regent Court (ScHARR), 30 Regent Street, Sheffield, England, S1 4DA

* Corresponding author

**Conflict of interest:** The authors declare that they have no competing interests.

**Funding:** Dr Joseph Kwon was supported by the Wellcome Trust [108903/B/15/Z] for PhD studentship.

**Acknowledgement:** We would like to thank Dr Matthew Franklin at the School of Health and Related Research, University of Sheffield, for kindly reviewing the manuscript and offering detailed feedback.

# [Contents](#_Toc114491828)

[A1 Outline 3](#_Toc114491829)

[A2 Methods for developing the falls prevention conceptual model 3](#_Toc114491830)

[A3 Understanding the local context for model development 4](#_Toc114491831)

[A3.1 Identifying relevant local stakeholders 4](#_Toc114491832)

[A3.2 Choosing the mode of stakeholder engagement 4](#_Toc114491833)

[A3.3 Establishing approaches to evidence searching 5](#_Toc114491834)

[A3.4 Time and resource availability and protocol document 6](#_Toc114491835)

[A4 What is the problem? 6](#_Toc114491836)

[A5 Why is this a problem? 7](#_Toc114491837)

[A5.1 Outcome range of falls and falls prevention 9](#_Toc114491838)

[A5.2 Heterogeneity and dynamic complexity 10](#_Toc114491839)

[A5.3 Behavioural factors and implementation challenges 10](#_Toc114491840)

[A5.4 Issues of equity 11](#_Toc114491841)

[A6 Identifying causal links in the disease process 11](#_Toc114491842)

[A6.1 Sociodemographic variables 12](#_Toc114491843)

[A6.2 Fall-related variables 13](#_Toc114491844)

[A6.3 Frailty and intrinsic variables 13](#_Toc114491845)

[A6.4 Environmental variables 14](#_Toc114491846)

[A7 Identifying relevant interventions 15](#_Toc114491847)

[A7.1 Current falls prevention in Sheffield 15](#_Toc114491848)

[A7.2 Recommended falls prevention in UK guidelines 17](#_Toc114491849)

[A7.3 Cognitively impaired persons and persons with intervention history 18](#_Toc114491850)

[A7.4 Additional falls prevention strategies 19](#_Toc114491851)

[References 21](#_Toc114491852)

# A1 Outline

The development of a health economic decision model should commence by conceptualising the decision problem and setting the model structure [1, 2]. The conceptual model differs from the final implemented model in that it is unconstrained by data availability or technical competency of the analyst. Accordingly, the conceptual model can consider the full range of relevant causal mechanisms and set a comprehensive boundary within which the final model is parameterised.

Section A2 outlines the conceptual modelling method that follows the phases and principles proposed by Squires and colleagues [1]. The subsequent sections address each of the development phases: Section A3 describes the processes of engaging local stakeholders to contextualise the decision problem. Sections A4 and A5 further clarify the nature of the decision problem. Section A6 conceptualises the major causal links in falls epidemiology. Section A7 conceptualises the current and recommended falls prevention strategies based on stakeholder inputs, guideline recommendations and wider literature, as well as alternative intervention strategies to be comparatively evaluated.

# A2 Methods for developing the falls prevention conceptual model

Squires and colleagues lay out four phases to public health economic model development [1]:

1. Aligning the framework with the decision-making process
2. Identifying relevant stakeholders
3. Understanding the problem
4. Developing and justifying the model structure

Phase (A) sets the foundation for the modelling project, selecting the modes of stakeholder engagement, evidence searching, time/resource allocation, and documentation. For Phase (B), a relevant stakeholder is “any person who impacts on or is impacted upon in the system” (p. 5) [1]. Stakeholders together help define the model scope, make value judgements, recommend assumptions on model structure and choose interventions to be evaluated. They can be divided into three types: (i) system owners – e.g., CCG and City Council commissioners; (ii) actors in the system – e.g., falls prevention clinical experts and falls modelling experts; and (iii) customers of the interventions – e.g., older persons and their informal caregivers. Outcomes of Phases (A) and (B) are reported in Section A3.

Phase (C) is divided into two parts: (I) developing a conceptual model of the problem that describes the hypothesised causal relationships based on the project scope, literature and stakeholder inputs; and (II) describing the present resource pathway in the decision-making setting. Part (I) can be formulated as a series of questions and activities:

- 1. What is the problem?
  2. Why is this a problem?
  3. Identifying causal links in the disease process
  4. Identifying relevant interventions

Sections A4 to A7 address each of these questions and activities in sequence. Part (II) of Phase (C) is addressed alongside activity (4).

The methods and results for Phase (D) are reported in Appendix B informing the ‘Model parameterisation’ section in the main manuscript. Phase (D) also involved a review of existing models in the problem area [1]. This review has been conducted and reported elsewhere [3, 4].

For all phases, Squires and colleagues highlight the following principles: (a) systems approach to modelling – e.g., identifying elements contributing to dynamic complexity; (b) documented understanding of the problem before and alongside developing and justifying the model structure; (c) strong communication with stakeholders; and (d) systematic consideration of the determinants of health including demographic, lifestyle, socioeconomic, and environmental factors [1]. In the interest of space, relevant documents for the conceptual model development have not been included in this manuscript; they are available from the authors upon reasonable request.

# A3 Understanding the local context for model development

This section addresses Phases (A) (aligning the framework with the decision-making process) and (B) (identifying relevant stakeholders). Because several key stakeholders were involved in the project from the outset (prior to the detailed framework alignment), the stakeholder identification under Phase (B) is described first (Section A3.1). The steps within Phase (A) are then described [1]: choosing the mode of stakeholder engagement (A3.2); establishing approaches to evidence searching (A3.3); and establishing resource availability and producing a protocol document (A3.4).

## A3.1 Identifying relevant local stakeholders

Table A1 lists the stakeholders who were involved in model conceptualisation. The Sheffield City Council (SCC) Public Health Principal (PHP) was involved in the project from the outset, suggesting ways in which economic modelling could assist commissioning decisions, establishing contacts with other system owners and actors, and serving as the main point of contact. Further system owners and actors were identified by snowballing from the initial group.

For customers of the intervention – i.e., older users and eligible non-users of falls prevention services – a formal qualitative research was conducted as published elsewhere [5]. Table A1 also acknowledges the contributions of independent scientific reviewers who provided written and oral feedback.

| **Table A1** Stakeholder types and members for falls prevention conceptual model development. | | |
| --- | --- | --- |
| **Stakeholder type** | **Stakeholder** | **Mode of engagement** |
| System owners | - Sheffield City Council Public Health Principal - Sheffield City Council health economist - Sheffield CCG commissioners: Chief Nurse; Commissioning director - Sheffield CCG data scientist | Face-to-face meetings and phone and email exchanges |
| Actors in the system – integrated care | - Managers of social prescribing organisations in Sheffield - Age UK Sheffield | Face-to-face meetings |
| Actors in the system – falls prevention | - Sheffield Falls Clinic falls specialist geriatrician - Sheffield Falls Clinic multidisciplinary team - PT and OT leads in Sheffield Teaching Hospitals - Dance to Health – falls prevention service provider - Age UK Sheffield – falls prevention service provider - Falls modelling expert | Face-to-face and online meetings |
| Customers of the intervention | - Older persons in Sheffield - Age UK Sheffield – representing older persons | Face-to-face focus groups, interviews and meetings |
| Independent scientific reviewers | - Two health economics modelling experts - One health services research expert | Face-to-face meetings and document feedbacks |
| **Abbreviation:** CCG: Clinical Commissioning Group; OT: occupational therapy; PT: physiotherapy | | |

Regarding the motivations for participating in the conceptualisation process, the system owners, actors and customers of falls prevention commonly sought to improve the local service quality and volume. In addition, system owners had a strategic vision for integrating complex services to achieve efficiency and quality and improving local analytic capacity.

## A3.2 Choosing the mode of stakeholder engagement

Table A1 also describes the modes of stakeholder engagement. It emerged at the early project stage that regular face-to-face stakeholder workshops would prove impractical for most stakeholders, particularly those engaged in full-time clinical practice. The mode of engagement was hence kept flexible to accommodate individual stakeholders’ schedule and commitment. This mainly involved one-to-one face-to-face or phone conversations.

## A3.3 Establishing approaches to evidence searching

The evidence searching approaches for model conceptualisation can be grouped into four: (1) stakeholder consultation; (2) systematic literature review – i.e., using explicit, systematic database search strategies; (3) general (non-systematic) literature review; and (4) primary data analysis. Table A2 shows how the approaches contributed to each conceptualisation phase or aspect.

| **Table A2** Summary of evidence searching approaches for model conceptualisation. | | |
| --- | --- | --- |
| **Conceptualisation phase** | **Evidence searching approach** | **Detail** |
| Initial scoping | Stakeholder consultation | - Falls Planning Group meeting with CCG commissioners, PHP and falls modelling expert on relevance of Public Health England falls prevention model [6] for local decision-making - Discussion with PHP on target population, type of analysis, perspective, evaluation time horizon and CCG routine data access |
|  | General literature review | - UK guidelines on community-based falls prevention: [7-10] - Cochrane systematic reviews of falls prevention RCTs: e.g., [11, 12] |
| Falls epidemiology | Stakeholder consultation | - Meeting with falls specialist geriatrician on major falls risk factors and health consequences |
|  | General literature review | - Range of falls risk factors: e.g., [9, 13, 14] - Range of health and economic consequences of falls: e.g., [15-17] - Use of routine data for falls risk analyses: e.g., [18, 19] |
|  | Primary data analysis | - Analysis of ELSA data on risk factors for MA and non-MA falls |
| Falls prevention strategy | Stakeholder consultation^1^ | - Observed multifactorial intervention at Sheffield falls clinic - Meeting with Age UK on home visit falls prevention services - Meeting with PT and OT leads of STH on current prevention pathways - Observed Dance to Health sessions and met with management |
|  | Systematic literature review | - Systematic review of community-based falls prevention RCTs (identified studies combined with those from previous systematic reviews).^2^ |
|  | General literature review | - Falls prevention guidelines:^1^ [7, 9, 10, 20-22] - Report on Sheffield Perfect Patient Pathway [23] - Previous systematic reviews of community-based falls prevention RCTs: e.g., [11, 12, 24, 25] - Literature on falls prevention facilitators and barriers: e.g., [26] - Falls prevention in broader geriatric health promotion context: e.g., [27, 28] |
|  | Primary data analysis | - Qualitative research with older persons in Sheffield on facilitators and barriers to implementing NICE falls prevention guideline.^3^ |
| Modelling features | Stakeholder consultation | - Meeting with falls modelling expert on modelling challenges - Independent scientific review on preliminary conceptual model - Meeting with SCC health economist on modelling local decision problems |
|  | Systematic literature review | - Systematic review of falls prevention economic models:^4^ how decision problem was conceptualised |
|  | General literature review | - Expert guideline on falls prevention economic evaluation [29] - Methodological challenges to public health economic modelling [30] - Problem conceptualisation for models incorporating capacity constraints: e.g., [31, 32] |
|  | Primary data analysis | - Qualitative research with older persons in Sheffield on key methodological and evaluative challenges for falls prevention economic modelling.^3^ |
| **Abbreviation:** CCG: clinical commissioning group; ELSA: English Longitudinal Study of Ageing; MA fall: fall requiring medical attention; NICE: National Institute for Health and Care Excellence; PHP: Public Health Principal; RCT: randomised controlled trial; SCC: Sheffield City Council; STH: Sheffield Teaching Hospitals  ^1^ Stakeholder consultations and UK guidelines on community-based falls prevention were used to conceptualise the ‘recommended’ falls prevention strategy. Stakeholder consultations were used to understand ‘current practice’.  ^2^ See Section B2.2 in Appendix B for methods and results.  ^3^ See publication for methods and results [5]. Qualitative research with falls prevention professionals in Sheffield was also initially planned but not conducted due to the Covid-19 pandemic.  ^4^ See publications for methods and results [3, 4]. | | |

The initial scoping of the decision problem around falls prevention involved stakeholder consultations and general literature review. The Falls Planning Group meeting was the first discussion between system owners, the falls modelling expert, and the PhD researcher on the decision problem. The general literature review covered UK guidelines on community-based falls prevention, the Cochrane systematic reviews on falls prevention RCTs, and several existing falls prevention economic models [33].

Subsequent, more detailed evidence searching partitioned the focus areas into falls epidemiology, falls prevention interventions, and modelling features. Consultations with falls prevention professionals – the falls specialist geriatrician, PT and OT leads, home visit professionals at Age UK, and Dance to Health managers and instructors – contributed to the understanding of both falls epidemiology and intervention. A systematic review of community-based falls prevention RCTs was conducted to understand the most up-to-date range of intervention evidence (see Section B2.2 in Appendix B). No systematic review was conducted for falls epidemiology, and the epidemiological evidence was based mainly on existing systematic reviews (e.g., on falls risk factors [13]). Epidemiological evidence was also sourced from a primary analysis of the English Longitudinal Survey of Ageing (ELSA) data.

Challenges to model conceptualisation and implementation were discussed from early project stages. The Falls Planning Group meeting discussed the relevance of outputs from the Public Health England model [6] for decision-making. This was supplemented by further consultations with the falls modelling expert at ScHARR and the SCC health economist and independent scientific review by another expert modeller. Because the decision problem had initially been framed as one of capacity shortage in falls prevention, the literature on capacity modelling were also consulted [31, 32]. The systematic methodological review on relevant modelling challenges was similarly consulted [30].

## A3.4 Time and resource availability and protocol document

The protocol document was produced based on initial scoping. This detailed the time and resources available for the modelling project and contained a conceptual model diagram and a logic model illustrating the preliminary understanding of the problem. It thereafter framed further discussions with stakeholders and set strategies for literature reviews and primary data analyses.

# A4 What is the problem?

Local commissioners in Sheffield perceived falls prevention as part of a broader integrated package of geriatric public health interventions, namely the Active Support & Recovery (AS&R) scheme [34] aligned with the Better Care Fund (BCF) initiative [35]. The aim of AS&R is “to improve the quality, efficiency and volume of care provided outside of hospital” (p. 2) [34]. The ‘Neighbourhood teams’ of GP practices, social care services and the voluntary sector would design services, mobilise relevant community assets, and address “all determinants of health such as biopsychosocial and environmental factors, not just healthcare” (p. 6) [34]. The ‘essential outcomes’ include: reduction in number of hospital (re-)admissions and length of stay; efficiency savings in service delivery; increase in the proportion of people receiving holistic, person-centred care in community; and reduction in the number of long-term care (LTC) admissions (p. 3) [34]. Patient-reported outcome measures such as health utilities were not mentioned, but the last two essential outcomes indicate decision-makers’ commitment to protecting the functional independence of older persons.

Evaluation of community-based falls prevention shares the core themes of AS&R, namely: reduction of public sector acute and LTC costs; provision of holistic person-centred care in out-of-hospital setting; maintaining independence of older people (e.g., preventing permanent LTC admissions); addressing wide determinants of falls risk and falls severity; and active participation of clinical and nonclinical community members in service design and implementation. The community focus excluded falls prevention initiatives in LTC and hospital inpatient settings despite these being major falls prevention arenas [36, 37]. The PHP asked for the starting age of the geriatric target population to be set as low as possible to promote primary prevention. This was eventually set as age 60 which is the age at which falls data were first collected in ELSA [38, 39]. Local commissioners oversee a geographical jurisdiction rather than a specific cohort; the target population hence includes community-dwelling cohorts who newly enter the target age range during the analysis horizon.

The default evaluation framework for the CCG and SCC is return on investment from the public sector perspective over a time horizon of five years [6, 40]. However, the PHP confirmed that the model-based evaluation need not be confined to this organisational mandate and should track wider outcomes over a longer time horizon. This is also more consistent with the objectives of AS&R to provide holistic person-centred care and maintain independence of older people. Hence, the primary evaluation framework for the model will be CUA over a 40-year horizon (i.e., near lifetime for the initial cohort aged 60+ at model baseline, though not for newly incoming cohorts) and societal perspective to track non-health outcomes and societal intervention costs incurred outside the public sector.

# A5 Why is this a problem?

The core rationale for community-based fall prevention was described in Backgrounds of the main manuscript. This section supplements this via a conceptual model in Figure A1 and arranges the concepts by the following themes: (1) outcome range of falls and falls prevention (Section A5.1); (2) heterogeneity and dynamic complexity (A5.2); (3) behavioural factors and implementation challenges (A5.3); and (4) issues of equity (A5.4).


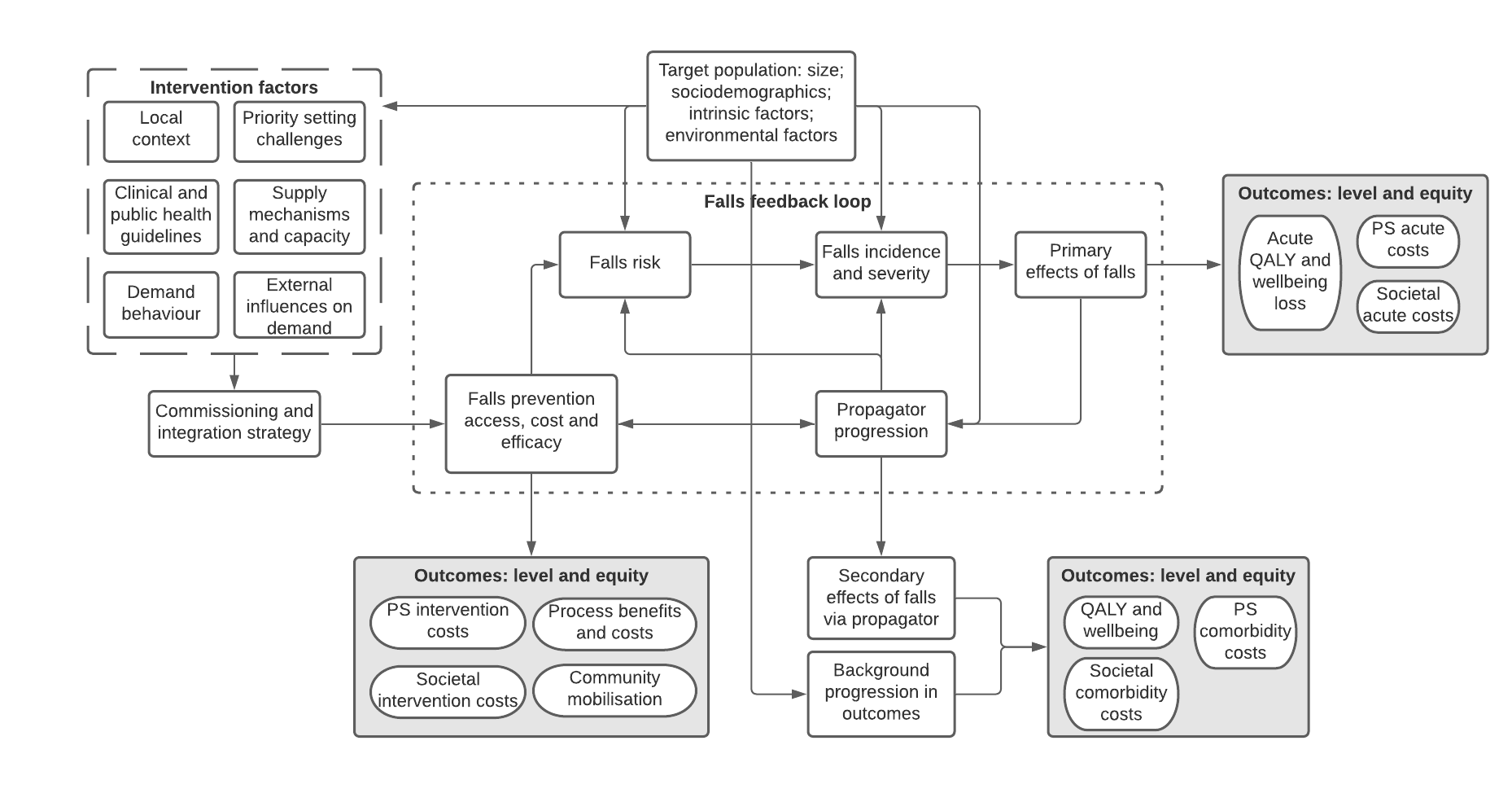


**Figure A1** Conceptual model of the decision problem for community-based falls prevention. **Abbreviation:** PS: public sector; QALY: quality-adjusted life year.

## A5.1 Outcome range of falls and falls prevention

The first key rationale for falls prevention lies in the diverse adverse impacts of falls older persons, care systems and wider society. As noted in Figure A1, falls have acute impacts on health, wellbeing (i.e., non-health aspects of quality of life not or poorly captured by health utility measures [41]), public sector care systems, and wider society. These are supplemented by the secondary effects via progression in ‘propagator’ variables; these effects determine the long-term levels of QALY, wellbeing and comorbidity care costs. A key propagator variable is frailty: a fall can cause physical impairments and functional limitations that are components of frailty [42]; frailty, in turn, is an independent risk factor for falls [43, 44]. Further propagators such as fear of falling are discussed in Section A6 below.

Particularly significant are the non-health outcomes of falls which expand the problem boundary beyond that typically overseen by the healthcare decision-makers. Prominent non-health outcomes of falls include: reduction in social wellbeing [45-47]; OOP care expenditures (around 12% of annual care costs of fallers) [48]; loss in paid and unpaid productivity [49, 50] and the related loss in older persons’ wellbeing [51]; and informal caregiver cost (around 22% of annual care costs of fallers) [52] and health-related stress [53], particularly for old/frail caregivers [54-56]. Moreover, how these outcomes are experienced at the individual level – e.g., number of persons enjoying some decent level of capability [57], fair innings [58], or financial protection [59] – may be more consistent with the principles of person-centred care under AS&R than the population-level aggregate.

Meanwhile, falls prevention also incurs costs accrued outside the healthcare system and these must be balanced against the non-health, societal outcomes of falls. These costs include: social stigma in falls prevention participation, particularly in contexts where geriatric health promotion is uncommon [60] (but participation can also bring social benefits [61]); private co-payments and costs (e.g., for transport); and time opportunity costs for participants and accompanying caregivers [62, 63]. Falls prevention may also bring benefits that chiefly accrue to the community rather than to individuals [64-66]; for example, community-wide participation can strengthen the community’s ability to organise other health promotion initiatives. The communal benefits should be weighed against the resources invested for social mobilisation, particularly those not reimbursed by the public sector (e.g., volunteer labour). These non-health outcomes and related societal intervention costs are summarised in Table A3.

| **Table A3** Non-health outcomes and societal intervention costs of falls and falls prevention. | | |
| --- | --- | --- |
| **Category** | **Non-health outcome** | **Societal intervention cost** |
| (1) Older persons’ social wellbeing | Social wellbeing affected by falls and comorbidities;^1^ intervention process benefits on wellbeing. | Intervention process costs (e.g., stigma) |
| (2) Personal finance | Out-of-pocket care expenditure due to falls and comorbidities^1^ | Private co-payment for intervention (e.g., session fee, equipment, transport) |
| (3) Productivity | Productivity loss due to falls and comorbidities^1^ | Time opportunity cost of older participants and volunteers |
| (4) Informal caregiving | Informal caregiver burden (productivity loss, health loss) due to falls and comorbidities;^1^ process benefit of informal care receipt; process benefit of joint intervention attendance for participant and caregiver | Time opportunity cost of informal caregivers attending intervention |
| (5) Common good | Community empowerment through intervention and other participations. | Financial and non-financial cost of social mobilisation |
| ^1^ These would be reduced by falls prevention intervention; the reductions would count as positive non-health benefits. | | |

In the Sheffield context, a key objective of AS&R is to prevent permanent institutionalisation that removes the older person from his/her social network. The qualitative research participants emphasised the social benefits of falls prevention, particularly group exercise, and did not report any social stigma associated with participation [5]. They also mentioned private intervention co-payments including transport costs as barriers to uptake and adherence. Other highlighted barriers included inconvenient timing and location which likely introduce time opportunity cost to participation [5]. A key aim of AS&R is to mobilise relevant community assets including the voluntary sector to implement prevention; hence, community empowerment via falls prevention is an important process outcome. Overall, the diverse adverse impacts of falls build a case for prevention, but the benefits of prevention should be weighed against the diverse range of costs involved.

## A5.2 Heterogeneity and dynamic complexity

Figure A1 notes the influence of diverse target population characteristics, including sociodemographic, intrinsic, and environmental factors, on the multifactorial falls risk profile. This introduces significant heterogeneity in falls risk and severity and endows further complexity to commissioning. The latter would need to account for variations in intervention need, access, cost and efficacy across population subgroups [67, 68]. The conceptual model also notes the influence of target population characteristics on background outcome progressions. These outcomes are not affected by the primary or secondary effects of falls but nonetheless influence the final evaluation outcomes. The underlying difference in life expectancy between socioeconomic subgroups, for example, could introduce a potentially unfair difference in the volume of QALY each subgroup can derive from falls prevention that improves the health utility level – henceforth referred as the life expectancy differential (LED) problem [69].

Dynamic complexity arises from intertemporal interactions between causal mechanisms yield non-linear outcomes and potentially higher heterogeneity between individuals and subgroups [70]. A small variation in personal or environmental determinant can yield substantial differences in long-term outcomes through feedback loops [30]. Central to the conceptual model is the cyclical ‘Falls feedback loop’ in Figure A1 that generates the secondary effects of falls via propagator progression and influences subsequent falls risk and severity and falls prevention access, cost, and efficacy.

In the Sheffield context, stakeholders suggested several factors relevant to heterogeneity and dynamic complexity. Multivariate frailty was highlighted as a better indicator of falls risk heterogeneity than age and sex by the falls modelling expert; the PT and OT leads similarly highlighted intrinsic physical capacity variables such as gait impairment that are currently used as falls risk screening tools. Physical activity level was highlighted by the council health economist as a key characteristic for planning health promotion initiatives. The qualitative data identified several vulnerable subgroups that should be prioritised by commissioning; these were delineated by comorbidity levels (i.e., frailty), cognitive status, and social isolation [5]. Moreover, both the qualitative data and the independent scientific review highlighted seasonal trends in environmental falls risk (e.g., frozen pavements).

## A5.3 Behavioural factors and implementation challenges

The qualitative data highlighted health and social motives of older individuals as key behavioural determinants of intervention uptake and adherence [5]. Communal initiatives to manage environmental risk factors were also recommended [5]. Likewise, a local pilot falls prevention scheme found that branding the exercise interventions as strength and balance improvement rather than falls prevention appealed to the health motive and reduced stigma, thereby raising uptake [23]. Accordingly, Figure A1 highlights demand behaviours and external influences on demand (e.g., communal responsibility, branding) as key intervention factors. These behavioural components introduce implementation challenges that influence the effectiveness and population reach/impact of falls prevention [71].

The qualitative research also identified diverse supply-side mechanisms that affected implementation [5]. Professional competence, shaped by psychology and organisational culture, was perceived to be a key implementation facilitator [72, 73]. But other system-side constraints were likewise perceived, such as time/capacity constraints and lack of coordination with other professionals [5]. This suggests that a coherent integration strategy is required to implement a citywide falls prevention programme [74-77]. To optimise the implementation strategy, the commissioning should distinguish between demand- and supply-side influences on the same implementation process; Table A4 lists the terms used in this study to describe the implementation processes by their demand and supply dimensions. Intervention access, for example, is shaped by demand-side uptake and supply-side adoption.

| **Table A4** Implementation processes by demand and supply dimensions. | | |
| --- | --- | --- |
| **Process** | **Demand dimension** | **Supply dimension** |
| *Access* | Uptake [78] | Adoption [79] |
| *Compliance* | Adherence [78] | Fidelity [80] |
| *Sustainability^1^* | Persistence [81] | Maintenance [79] |
| ^1^ The extent to which access and compliance are preserved over time after the initial receipt for interventions requiring ongoing receipt (e.g., exercise). | | |

## A5.4 Issues of equity

As highlighted by an international expert panel, healthcare decision-makers face several priority setting criteria beyond cost-effectiveness in the interest of fairness or equity [82]. These criteria include prioritising the care needs of socially deprived subgroups and those with more severe disease and past health loss among similar-age peers. These two vulnerable subgroups overlap in practice given the strong influence of social factors (e.g., income, housing) on health both over the earlier life course and contemporaneously in old age [27, 83]. In the Sheffield context, the lower super output area (LSOA) multiple deprivation variable [84] was perceived to be the main characteristic of equity relevance by the commissioners. The qualitative data similarly highlighted area-based social deprivation as a factor influencing intervention demand and supply; ethnic/linguistic minorities were also mentioned as being disadvantaged [5]. As for severity characteristics, the qualitative data highlighted comorbidity level (i.e., frailty), cognitive impairment and social isolation [5].

The issues of equity widen the problem boundary for falls prevention. As noted in the shaded outcome boxes in Figure A1, evaluation should now consider not only outcome levels but their distribution across population subgroups and individuals. Prioritising vulnerable subgroups likely worsens the overall cost-effectiveness of the intervention given factors such as the LED problem and the ‘double jeopardy’ (DJ) problem whereby vulnerable groups derive lower efficacy (e.g., due to comorbidity-related contraindications) and/or poorer implementation quality [85, 86]. The need to handle such equity-efficiency trade-offs introduce further complexity to the decision-making.

# A6 Identifying causal links in the disease process

This section further explores the key causal variables within the conceptual model. Generally, the variables introduce dynamic complexity and affect not only falls risk and incidence (i.e., the disease process defined narrowly) but also wider processes and outcomes such as intervention access (which in turn reduce the falls risk) and secondary effects of falls. In Table A5, they are grouped into four categories: (I) sociodemographic variables (Section A6.1); (II) fall-related variables (A6.2); (III) frailty and intrinsic capacity variables (A6.3); and (IV) environmental variables (A6.4). Table A5 summarises the links between the variables and falls prevention processes and outcomes but does not describe the inter-variable links (e.g., between falls and frailty). Figure A1 should also be referred for the links.

| **Table A5** Links between key causal variables and falls prevention processes and outcomes. | | | | | | |
| --- | --- | --- | --- | --- | --- | --- |
| **Variables** | **Processes and outcomes** | | | | | |
|  | Falls risk and severity | Falls secondary effects^1^ | Background health and care cost^2^ | Non-health outcomes^3^ | Priority setting implication | Intervention access, cost and efficacy^4^ |
| **Sociodemographic** | | | | | | |
| Age | **×** |  | **×** | **×** |  | **×** |
| Sex | **×** |  | **×** | **×** |  | **×** |
| SES | **×** |  | **×** | **×** | **×** | **×** |
| **Fall-related** | | | | | | |
| Falls history | **×** | **×** |  | **×** |  | **×** |
| Fear of falling | **×** | **×** | **×** | **×** |  | **×** |
| **Frailty and intrinsic capacity^5^** | | | | | | |
| Frailty | **×** | **×** | **×** | **×** | **×** | **×** |
| Gait and balance | **×** | **×** | **×** | **×** |  | **×** |
| Cognitive status | **×** | **×** | **×** | **×** | **×** | **×** |
| PA level | **×** | **×** | **×** | **×** |  | **×** |
| Other intrinsic | **×** | **×** |  |  |  |  |
| **Environmental** | | | | | | |
| Home space | **×** |  | **×** | **×** |  | **×** |
| Public space | **×** |  | **×** | **×** |  | **×** |
| Social community | **×** | **×** | **×** | **×** | **×** | **×** |
| **Abbreviation:** PA: physical activity; SES: socioeconomic status  ^1^ E.g., excess mortality risk and functional decline after a serious fracture.  ^2^ Health status and care costs that are not associated with the acute and secondary effects of falls.  ^3^ E.g., productivity level, out-of-pocket care expenditure, informal caregiver burden (see Section A5.1).  ^4^ Intervention access is determined by eligibility (defined by guidelines such as NICE CG161 [7]), supply, and demand. Intervention costs include societal costs and wider health, non-health, and side effects of interventions (see Section A5.1).  ^5^ This concerns physical, mental, and cognitive capacities of the individual as opposed to extrinsic factors in the individual’s social community and physical environment (p. 32-33) [27]. | | | | | | |

## A6.1 Sociodemographic variables

Age and sex are well-established independent risk factors for falls incidence, injury type, and severity [13, 87-90]. For example, older women are likelier than men to experience fractures after falling, while men are likelier to experience traumatic brain injuries [87]. Regarding background health status, there is evidence that age and sex impact geriatric health utilities even after controlling for frailty and socioeconomic status (SES) [91]. Their associations with healthcare costs is less strong, with proximity to death being a stronger predictor than age itself [92]. Age and sex affect diverse non-health outcomes, and reducing informal caregiving burden and promoting productivity among older women are public health priorities [27]. Sheffield stakeholders did not mention age or sex as characteristics with priority setting implications [5]; yet age- or sex-based rationing (e.g., if intervention is not cost-effective for the oldest age subgroup) would go against the principles of NHS and NICE [93, 94]. Falls prevention RCTs often use age and sex as inclusion/exclusion criteria [11]; hence, availability of evidence-based interventions may vary by age and sex. Intervention participation is typically higher among older women than men [11, 95]. Overall, age and sex affect almost all key processes and outcomes in Table A5 and hence are key causal variables.

There are relatively few analyses on SES as an independent falls risk factor: a systematic review of prospective falls epidemiological studies included education as the only SES-related risk factor and did not find a statistically significant association with falls risk [13]. A prospective analysis of ELSA likewise found that household wealth is not significantly associated with falls risk [89]. By contrast, primary analysis of ELSA in this study found that subjective report of financial difficulty is significantly associated with falls risk. Regarding background health status, one study found that education, but not income, had an independent impact on older persons’ health utilities after controlling for frailty [91]. Nevertheless, improving the financial circumstances of older persons is regarded as a key strategy for promoting older persons’ health and social participation [96]; hence, reducing fall-related OOP care expenditure incurred by financially deprived subgroups should be a priority [48, 82]. As noted, Sheffield CCG recommended LSOA-level multiple deprivation as the characteristic of equity relevance. During a pilot falls prevention scheme, CCG prioritised intervention access in socially deprived areas, demonstrating the area-level, SES-mediated differential in local intervention access. Overall, SES is a key causal variable with priority setting implications and impacts on multiple processes and outcomes.

## A6.2 Fall-related variables

Falls history is one of the most reliable indicators of falls risk [7, 97]. Incidence of a fall leaves the person at high risk of a recurrent fall within a year [98]. Recurrence of falls requiring medical attention (MA falls) is also frequent [99]. NICE CG161 uses recurrent, not single (unless requiring medical attention), falls history as one of the screening criteria for proactive intervention access [7]. Recurrent falls also have stronger association with physical frailty [100]; single and recurrent fallers thus likely incur heterogeneous secondary effects of their respective fall episodes. Falls of all severities, including those not incurring injuries, have secondary health effects manifesting in terms of functional decline, excess mortality and LTC admissions [42, 101-104], as well as various non-health impacts. Specific fall-related injuries inflict different acute and long-term health impacts: wrist fracture patients, for example, have recovered their pre-fracture health utility level within 12 months, while hip fracture patients have not recovered even after 48 months [105]. Finally, falls prevention RCTs often demarcate their target population by falls history types [11]; falls history thus influences the range of available interventions.

Fear of falling encompasses multiple, potentially non-overlapping concepts: physiological – e.g., change in autonomic reactivity; behavioural – e.g., reduction in walking speed to avoid falling; and cognitive – e.g., subjective assessment of one’s ability to avoid a fall while conducting daily/important tasks (i.e., self-efficacy) [106]. Fear remains a significant falls risk factor across its different conceptual forms [13, 106, 107]: it increases falls risk directly by impairing balance and/or indirectly by causing physical deconditioning via fear-related activity curtailment [106]. It is also a consequence of falling [107, 108], and likely propagates secondary effects of falls including functional impairment, all-cause mortality, cognitive decline, and reduced social wellbeing [109-112]. Several studies found that fear is more strongly associated with physical dependence than falls *per se* [15, 113]. Fear is not necessarily caused by a previous fall: according to one survey, only 37% of fear cases could be attributed to a previous fall [114]. Hence, fear could affect background health and care costs without mediating the secondary effects of falls. NICE CG161 recommends interventions that reduce cases of low self-efficacy (the cognitive form of fear) and fear of falling (the physiological-behavioural form) [7]. This is a case of falls prevention directly affecting a propagator variable: see arrow from falls prevention efficacy box to propagator progression box in Figure A1.

## A6.3 Frailty and intrinsic variables

This section discusses variables that are intrinsic to older persons beginning with frailty. There are two main frailty models in literature: phenotypic and cumulative deficit. The phenotypic model tracks the presence of a relatively small number of symptoms that indicate vulnerability in multiple organ systems; it hence generates categorical measures [115]. The cumulative deficit model tracks a group of deficits (at least 30) and calculates a frailty index between range 0-1 as a ratio between actual and potential numbers of deficits [116, 117]. Both models have independent effects on falls risk [43, 115, 118, 119] and severity [20, 120]; yet statistical comparisons suggest that the cumulative deficit model has greater predictive power [44] (confirmed by primary ELSA analysis in this study). The continuous nature of frailty index has a conceptual advantage in that even a minute change in frailty score contributes towards higher falls risk. The score change propagates the secondary effects of falls manifesting in terms of higher all-cause healthcare costs [121, 122], LTC admissions [18], excess mortality [18, 123], and non-health outcomes such as informal caregiver burden [124]. Frailty is an ideal tool for characterising the heterogeneity in background health utilities and care costs beyond sociodemographic factors [91, 121]. Frailty may serve as the health severity characteristic with priority setting implications [82]; it is moreover closely associated with social deprivation [125, 126] and hence provides some information on social inequities of health. It can enable targeting strategies as done recently in Sheffield [23]. Overall, continuous frailty index is a key causal variable with priority setting implications.

The second intrinsic variable of importance is gait and balance; their impairments are well-established falls risk factors highlighted by NICE CG161 [7, 87]. They are also outcomes of serious falls that establish a feedback loop between falls and falls risk [127, 128]. Slow walking speed is one of the key frailty phenotypes [115] and is associated with all-cause mortality [129], suggesting that it would affect the trajectories of background health and non-health outcomes. Abnormal gait and balance has a significant influence on intervention access; in NICE CG161, it is used as one of the screening criteria for proactive intervention access [7]. RCTs of multifactorial interventions likewise use it as an inclusion criterion to target high-risk groups [52, 130].

Cognitive impairment is a major risk factor for falls and related injuries [7, 131, 132]. Cognitive decline may also be a long-term, secondary effect of serious falls such as hip fracture, likely via physical deconditioning and activity restrictions that affect cognitive function [133]. Cognitive impairment affects broad health and non-health outcomes including all-cause mortality [134, 135], healthcare cost [136], informal caregiver burden [136, 137], and social wellbeing [138]. The qualitative data highlighted the difficulty faced by cognitively impaired persons in accessing appropriate interventions [5]. Thus, cognitive impairment is a major determinant of falls prevention intervention need and design such as whether and how informal caregivers are involved in delivery [139]. Moreover, intervention efficacy from falls prevention RCTs is less consistent for cognitively impaired persons than cognitively intact [140]. Overall, cognitive status encompasses multiple causal links and its impact on the intervention strategy is discussed further in Section A7.3.

Physical activity level was highlighted by council health economist as a key factor in designing and evaluating preventive interventions. High physical activity can reduce falls risk [141, 142]. Falls may also adversely affect physical activity pattern and subsequent physical deconditioning, establishing a feedback loop [102, 143]. Physical activity is a key determinant of healthy ageing beyond falls risk, reducing risks of chronic diseases and improving social wellbeing [21, 144]. Finally, higher physical activity level is a key outcome of falls prevention exercise as its wider health benefit beyond falls prevention [95]. NICE CG161 similarly recommends that falls prevention interventions promote behavioural/activity change among participants [7].

There are several further intrinsic capacity variables that have been identified as key falls risk factors in the literature: muscle weakness [145]; pain [146]; use of certain medications and polypharmacy [147, 148]; urinary incontinence [149]; visual impairment [150]; and depression [151]. Factors such as pain and depression are also consequences of falls (and closely associated with fear of falling) [106], thus propagating the secondary effects of falls. It should be noted that these variables can be incorporated as deficits in a frailty index [119]. The latter may thus serve as a parsimonious measure of the collective impact of diverse deficits to intrinsic capacity.

## A6.4 Environmental variables

Qualitative data highlighted the importance of environmental or extrinsic determinants of falls risk and health promotion [5], as did the independent scientific reviewer. Hazards at home such as poor lighting and inappropriate indoor footwear significantly increase falls risk [14, 152] and interact closely with visual impairment [150]. Beyond falls, housing quality is a key social determinant of physical health and broader wellbeing in old age [27, 153, 154]. Homeowners are likelier to implement HAM and derive consumption benefits from the home improvement [155], introducing an equity issue.

Despite the high volume of falls occurring outdoors, there are relatively few epidemiological studies on falls risk factors in the public outdoor spaces compared to those on home hazards [156]. Importantly, outdoor falls are more likely precipitated by environmental factors (e.g., uneven surfaces on sidewalks) than intrinsic capacity deficits [156]. Therefore, environmental interventions should complement those addressing intrinsic risk factors. Beyond falls, establishing safe outdoor spaces is a key strategy for creating age-friendly cities [154]. Moreover, improved local environment can significantly increase physical activity levels and hence reduce falls risk indirectly [5, 157, 158].

Finally, social isolation (e.g., living alone) is known to be associated with higher falls risk and worse health consequences after falling [159, 160]; the latter would propagate the secondary effects of falls. Availability of social support also reduces the link between fear of falling and consequent activity curtailment [161]. Beyond falls, older persons increasingly require social support to maintain functioning [27], and social isolation is associated with increased risks of several adverse events including cardiovascular disease, depression, and premature death [162]. Reducing social isolation is an important non-health outcome in itself, and promoting the social value of falls prevention is recommended by NICE CG161 as a wider intervention benefit [7]. Qualitative data identified socially isolated persons as a priority group for intervention access [5].

# A7 Identifying relevant interventions

This section explores appropriate interventions for the decision problem. Section A7.1 conceptualises the current falls prevention practice in Sheffield, seen here as representative of prevention initiatives occurring in other urban UK local health economies. Section A7.2 conceptualises the recommended falls prevention strategy based on the UK falls prevention guidelines. Section A7.3 discusses the prevention strategy for cognitively impaired persons and persons with intervention history under current and recommended practices. Section A7.4 conceptualises additional falls prevention strategies.

An important caveat is that conceptualisations of the current and recommended prevention occurred before and during the early stages of the Covid-19 pandemic. The model hence does not incorporate the pandemic’s impact on prevention features (e.g., limits to social gathering). As of writing (September 2022), all Covid-19-related restrictions have been lifted in the UK, but long-term effects on falls prevention supply and demand cannot be ruled out. Regarding the NICE guideline on falls prevention, there is no indication that the general strategy will change post-pandemic [163].

## A7.1 Current falls prevention in Sheffield

Understanding the current falls prevention practice in Sheffield mainly involved discussions with local stakeholders (commissioners, professionals, and qualitative research participants) and reference to published reports of falls prevention schemes implemented in Sheffield. Table A6 summarises the current or previously piloted falls prevention interventions in Sheffield managed by different system owners and actors. The interventions were classified by pathway: reactive, proactive, or self-referred.

| **Table A6** Current falls prevention interventions in Sheffield by system owner/actor. | | |
| --- | --- | --- |
| **System owner/actor** | **Falls prevention intervention** | **Pathway** |
| Sheffield NHS CCG and Sheffield City Council | SCC Home assessment and modification for hospitalised fallers | Reactive |
|  | SCC Fall alarms (self-financed) for hospitalised fallers | Reactive |
|  | STH rehabilitation therapy for MA fallers | Reactive |
|  | CCG Fracture Liaison Service (Tai Chi) | Reactive |
|  | CCG Integrated Community Therapy (ICT) team | Reactive; Proactive |
|  | [Pilot scheme] CCG Dance to Health group exercise (Otago and FaME) | Self-referred |
| Falls specialist geriatrician | Multidisciplinary Sheffield Falls Clinic | Reactive; Proactive |
| Evaluator of Sheffield PPP Falls Prevention | [Pilot scheme] QTUG and ICT team referral | Proactive |
| Age UK Sheffield | Independent Living Coordination programme (education, home exercise, referral to HAM) | Proactive |
| **Abbreviation:** FaME: Falls Management Exercise; MA faller: faller who received medical attention; OT: occupational therapy; PHP: Public Health Principal; PPP: Perfect Patient Pathway; PT: physiotherapy; QTUG: quantified timed up and go; SCC: Sheffield City Council; STH: Sheffield Teaching Hospitals | | |

The subsections below discuss the current interventions by pathway. The last subsection discusses the client flows under current practice and associated capacity implications.

### Current reactive pathway

In Table A6, reactive interventions comprised a high proportion (six out of nine) of falls prevention services. The proportion was even higher if temporary pilot schemes (Dance to Health and QTUG and ICT team referral) were excluded. HAM provided by SCC was part of the standard hospital discharge protocol for hospitalised fallers and constituted the main reactive component. SCC also provided a self-financed fall alarm service for discharged patients. The seven-week rehabilitation therapy provided by the Sheffield Teaching Hospitals (STH) comprised another part of the standard discharge protocol. For those who experienced a serious fracture that impaired activities of daily living, CCG provided the Fracture Liaison Service comprising twice-weekly group Tai Chi to around 20-30 clients.

The Sheffield Falls Clinic was based at the Assessment and Rehabilitation Centre (ARC) – an outpatient facility of STH – and operated by a multidisciplinary team of consultant geriatrician, physiotherapists, and occupational therapists for a single afternoon each week. Those referred received full medical, PT and OT assessments, and based on the identified falls risk factors, received tailored treatments, including medical interventions and referrals (e.g., medication change, cardiac pacing, referral to ophthalmologist), up to six weeks of one-to-one PT treatments, OT home modifications, and footwear and assistive devices. The assessment results and treatment prescriptions were sent to the GPs for monitoring. The Clinic catered to both reactive and proactive pathways. The ICT team was another multidisciplinary unit of PT and OT professionals operating mainly under the reactive pathway.

### Current proactive pathway

As noted, the Falls Clinic and the ICT team took on proactive clients. Moreover, between 2016 and 2018, a proactive falls prevention scheme was piloted in Sheffield as part of the Perfect Patient Pathway (PPP) Test Bed initiative run by STH. This initiative aimed to evaluate digital health technologies that targeted diverse population groups with complex needs including older persons at the onset of physical decline and hence eligible for falls prevention [23]. The falls prevention scheme: (i) targeted cognitively intact, ‘moderately frail’ (by electronic frailty index (eFI) [18]) older persons without falls history recorded in primary care data; (ii) screened individuals for high falls risk using the quantified timed up-and-go (QTUG) test; and (iii) referred high-risk individuals to multifactorial intervention conducted by the ICT team. The scheme involved nonclinical staff from community organisations conducting falls risk screening [23]. The scheme was subsequently discontinued after the pilot period. Therefore, the QTUG-based proactive pathway represents a potential option rather than current practice.

Age UK Sheffield provided its own proactive falls prevention through its Independent Living Coordination (ILC) programme. Clients were primarily referred to the programme via their GPs (i.e., proactive). The ILC staff conducted home visits and falls prevention services including education, home exercise and HAM referrals. This programme would likely qualify as a multifactorial intervention [12].

### Current self-referred pathway

Between 2017 and 2019, CCG piloted the Dance to Health group exercise in three locations: (1) central Sheffield; (2) Fir Vale – due to its social deprivation and high ethnic minority presence; and (3) Stocksbridge – due to its rural location and large geriatric population. The commissioning hence considered the objective of reducing social inequities in health and healthcare. The programme operated primarily within the self-referred pathway, relying on community marketing and peer recommendations rather than professional referrals. It involved weekly two-hour sessions supervised by a professional dance and postural stability instructor (PSI). The sessions incorporated components of Otago and FaME into a dance routine (with individually tailored difficulty) to improve enjoyability and long-term participation [164]. The CCG funding was discontinued after the pilot, at which point only group (1) sustained itself through self-financing of PSI fee and local church donation of venue. Therefore, self-referred prevention such as Dance to Health represents a potential option rather than current practice.

### Client flows and capacity implications

Current falls prevention in Sheffield thus implemented the NICE-recommended practice (described in Section A7.2) within the reactive and proactive pathways, particularly the multifactorial risk assessments and treatments. But it had a highly restricted intervention reach. For example, the Falls Clinic catered to around six clients per week, amounting to 300 clients per year. Likewise, the ICT team’s workflow was highly limited, with the team reporting capacity shortages when around 150 additional clients were added over 16 months during the QTUG scheme [23]. Age UK Sheffield had a limited client base, largely restricted to a relatively well-off region within Sheffield. These client flows hence represent a small proportion of the flow envisaged by the NICE guideline. For the reactive pathway alone, the guideline recommends that all MA fallers receive multifactorial intervention [7]; according to ELSA, the annual prevalence of MA fall is 6.8%, which amounts to around 8,500 MA fallers in Sheffield being eligible for the reactive pathway each year. The falls specialist geriatrician reported minimal issues with capacity under current practice; but this is likely due to a highly limited intervention eligibility rather than sufficient capacity at the NICE-envisaged level. For the self-referred pathway, the Dance to Health programme catered to around 40 regular participants during the two-year pilot and the low uptake was a major, persistent issue.

## A7.2 Recommended falls prevention in UK guidelines

Four guidelines were chiefly referred for understanding the normative falls prevention strategy in the UK community setting:

1. 2013 NICE falls prevention clinical guideline (‘NICE CG161’) [7]
2. 2019 NICE surveillance report for the update of the 2013 clinical guideline (‘NICE SR’) [20]
3. 2017 falls and fracture consensus statement by the Public Health England and the National Falls Prevention Coordination Group (‘PHE Consensus’) [10]
4. 2019 UK Chief Medical Officers’ physical activity guidelines (‘UK CMO’) [21].

In England and Wales, NICE CG161 remains normative for clinical practice; this was confirmed by commissioning and professional stakeholders in Sheffield. PHE Consensus mainly concurred with NICE CG161. UK CMO was not solely focused on falls prevention but was recommended by NICE SR as a reference point for falls prevention exercise. Figure A2 summarises the recommendations.


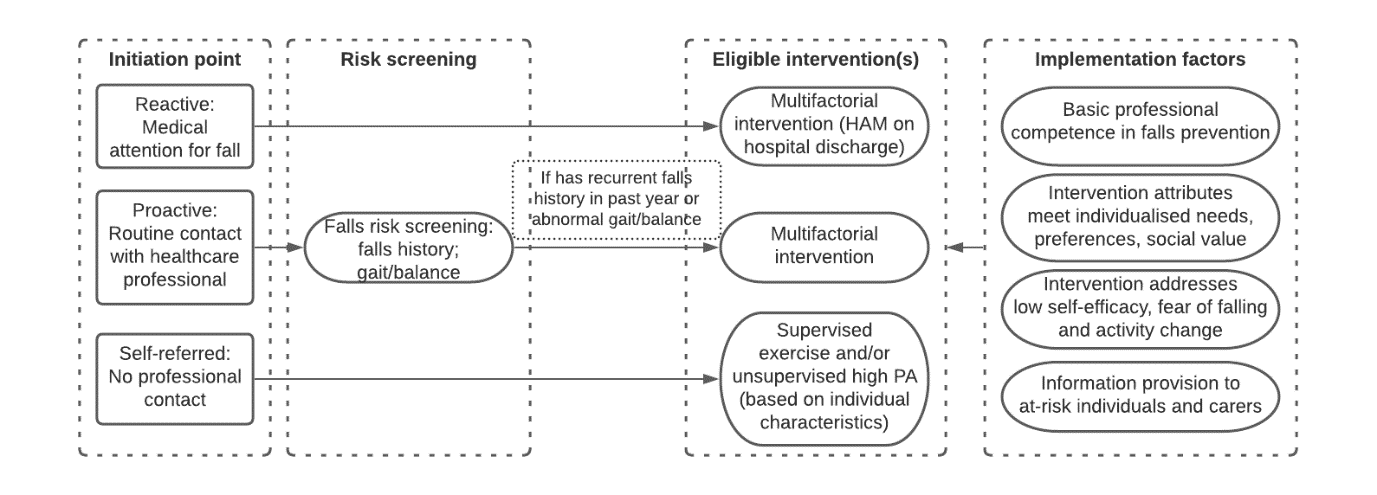


**Figure A2** Conceptual diagram of community-based falls prevention recommended by UK guidelines. **Abbreviation:** HAM: home assessment and modification; PA: physical activity.

Multifactorial intervention is central to the recommended practice for both reactive and proactive pathways. The latter targets older persons screened during routine care contact to have experienced recurrent falls in the past year and/or have abnormal gait/balance as assessed by tools such as the TUG test. Multifactorial intervention is conducted at dedicated sites such as the Falls Clinic and incorporates multidisciplinary assessment of diverse falls risk factors including falls history, gait/balance abnormality, muscle weakness, fear of falling, visual impairment, medication use, and falls hazards at home. Clients would then receive tailored sets of treatments, including exercise, HAM, medication modification, and vision improvements [7].

Recommendations for the self-referred pathway are centred around supervised exercise and unsupervised physical activity. Exercise interventions should be individually tailored, of progressive difficulty, and comprise a minimum of 50 hours of sessions for at least two hours per week [10]. UK CMO likewise recommends evidenced-based strength and balance exercise interventions in groups or at home (e.g., embedded in everyday activities) which are professionally supervised in case of frail or cognitively impaired persons; physical activity initiatives should aim to accumulate at least 150 minutes of moderate intensity exercise per week [21]. NICE SR recommends strategies that maintain this physical activity level even after professionally supervised exercise interventions end [20].

NICE CG161 also contains several recommendations regarding implementation factors [7]. On the supply-side, professionals in regular contact with older persons in the community should have basic competence in falls risk screening and referral; attributes of commissioned interventions should be adaptable to the heterogeneous needs and preferences of older persons; the social value of group-based programmes should be promoted; and interventions should have wider effects beyond falls prevention in promoting behavioural/activity change and addressing fear of falling and low self-efficacy. Information provision in appropriate languages to both older persons and their carers is recommended as the main demand-augmenting implementation strategy.

As mentioned, the current practice in Sheffield implemented the NICE-recommended practice in several areas; hence, the main point of difference between current and recommended practices is not the intervention type but the intervention reach. Attention should be paid to the capacity implications of scaling the current practice to the recommended levels. For example, as estimated during model parameterisation, fully implementing the NICE-recommended reactive and proactive pathways would require around seven full-time falls clinics; this is likely beyond the feasible capacity levels in Sheffield. Even for single-component exercise interventions, capacity may be constrained if the available venues are limited: the Dance to Health Sheffield management suggested that around 100 potential venues in the city for 20 participants each.

## A7.3 Cognitively impaired persons and persons with intervention history

The UK guidelines above contained limited recommendations regarding interventions for cognitively impaired older persons. NICE CG161 recognised cognitive impairment as a major falls risk factor and included cognitive assessment in the multifactorial risk assessment but suggested no differentiated intervention strategy for this subgroup [7]. UK CMO simply recommended professional supervision of exercise interventions for those with moderate-severe dementia [21]. By contrast, falls prevention efficacy studies draw a sharp distinction by cognitive status for recruitment. For example, 89 of 159 trials included in the 2012 Cochrane review of falls prevention RCTs excluded cognitively impaired persons [11]. An overview of systematic reviews of RCTs found greater variations in intervention efficacies for this subgroup [140]. These suggest that attention should be paid to the heterogeneous intervention attributes (e.g., caregiver accompaniment), costs, and efficacies across cognitive status.

Regarding current practice in Sheffield, the falls specialist geriatrician mentioned that access to reactive discharge interventions such as HAM and therapy rehabilitation would not make the distinction by cognitive status. Hence, the current reactive pathway is accessible to both cognitively intact and impaired persons. For the proactive pathway, the lead PT at the Falls Clinic mentioned that older persons with diagnosed cognitive impairment were referred to the specialised Memory Clinic rather than the Falls Clinic. This, together with QTUG pilot scheme’s exclusion of cognitively impaired persons, suggests that the current proactive pathway is predominantly targeted at cognitively intact persons. For the self-referred pathway, the Sheffield lead for Dance to Health mentioned that the programme would accept cognitively impaired persons while involving their caregivers for safety. Independent assessment of the Dance to Health affirmed this approach [164]. Hence, the current self-referred pathway is accessible to both cognitively intact and impaired persons.

Another distinct subgroup comprises persons who have already received a given intervention in a previous period(s) but remain eligible in the current period. Given scarce resources, an option is to restrict intervention access to this subgroup. This would affect the long-term sustainability of intervention access; the issue is particularly relevant to reactive and proactive pathways where access is largely determined by clinical protocols. There was again limited information in the UK guidelines for this subgroup. Nevertheless, the intervention features likely differ by intervention history; for example, persons who have already received a multifactorial intervention are unlikely to undergo the same multifactorial risk assessment given pre-recorded assessment results.

Regarding current practice, the falls specialist geriatrician mentioned that standard reactive treatments such as rehabilitation or HAM are unlikely to be denied based on previous receipt history. Hence, reactive interventions are likely accessible to all eligible persons each year. For the proactive pathway, the geriatrician mentioned that individuals with intervention history who remain at high falls risk would ideally be referred back to the Falls Clinic for further assessment of any significant change in the falls risk profile; but such re-referrals are infrequent in practice. For the self-referred pathway, continued participation of older persons is strongly encouraged by the Dance to Health programme [164].

## A7.4 Additional falls prevention strategies

Several additional intervention strategies can be conceptualised based on the major themes of the decision problem. First, each of the three distinct pathways could be commissioned individually at their recommended level while the other two pathways are maintained at their current levels. Alternatively, two of the three pathways could be commissioned at their recommended levels.

Second, the current and recommended practices both envisage minimal engagement of community assets. This contrasts with the focus placed on community mobilisation by AS&R as well as the pilot QTUG scheme that involved community organisations in falls risk screening. Qualitative data also highlighted the importance of community marketing in intervention access [5]. An alternative strategy would involve active involvement of community assets in various roles ranging from falls risk screening under the proactive pathway to intervention uptake promotion under the self-referred pathway.

Third, there are potential concerns around the capacity implications of implementing the recommended practice. A small increase in proactive referrals under the QTUG scheme resulted in significant waiting lists for falls prevention services [23]. Although funding would remove many of the supply-side constraints, bottlenecks may remain in several areas such as the total feasible numbers of multidisciplinary teams and group exercise venues. Alternative strategies would recognise these constraints and explore means of rationing the scarce resources, likely in the form of targeting strategies. The QTUG scheme, for example, targeted the moderately frail according to eFI scores. Another option is to target high-risk individuals according to their predicted multivariate falls risk.

Fourth, the recommended practice contains relatively little guidance around reducing environmental falls risk factors (beyond home hazards) and building health-promoting public spaces; yet both were suggested by qualitative research participants as intersectoral policies [5]. Alternative strategies could explore the impact when environmental interventions supplement current or recommended practices.

Finally, as envisaged by commissioners, falls prevention is ideally a part of a broader package of further geriatric and earlier-life public health interventions. These interventions would change the underlying epidemiological characteristics of the target geriatric population. The problem boundary should incorporate situations of such epidemiological changes and their impact on falls prevention practice.

# References

1. Squires H, Chilcott J, Akehurst R, Burr J, Kelly MP. A framework for developing the structure of public health economic models. Value in Health. 2016;19(5):588-601.

2. Tappenden P. Conceptual modelling for health economic model development. HEDS Discussion Paper 12/05. 2012.

3. Kwon J, Squires H, Franklin M, Lee Y, Young T. Economic models of community-based falls prevention: a systematic review with subsequent commissioning and methodological recommendations. BMC Health Serv Res. 2022;22. doi: 10.1186/s12913-022-07647-6.

4. Kwon J, Squires H, Franklin M, Young T. Systematic review and critical methodological appraisal of community-based falls prevention economic models. Cost Effectiveness and Resource Allocation. 2022;20. doi: <https://doi.org/10.1186/s12962-022-00367-y>.

5. Kwon J, Lee Y, Young T, Squires H, Harris J. Qualitative research to inform economic modelling: a case study in older people’s views on implementing the NICE falls prevention guideline. BMC health services research. 2021;21(1):1-19.

6. Public Health England. A Return on Investment Tool for the Assessment of Falls Prevention Programmes for Older People Living in the Community. Public Health England. 2018.

7. National Institute for Health and Care Excellence. Falls in older people: assessing risk and prevention. National Institute for Health and Care Excellence. 2013;Clinical Guideline 161(nice.org.uk/guidance/cg161).

8. National Institute for Health and Care Excellence. Falls in older people (QS86). NICE Quality Standard 86. 2015;nice.org.uk/guidance/qs86.

9. Royal College of Nursing. Clinical practice guideline for the assessment and prevention of falls in older people. Clinical Practice Guidelines. 2005;London: Royal College of Nursing.

10. Public Health England. Falls and fracture consensus statement: Supporting commissioning for prevention. London: Public Health England. 2017.

11. Gillespie LD, Robertson MC, Gillespie WJ, Sherrington C, Gates S, Clemson LM, et al. Interventions for preventing falls in older people living in the community. Cochrane database of systematic reviews. 2012;(9).

12. Hopewell S, Adedire O, Copsey BJ, Boniface GJ, Sherrington C, Clemson L, et al. Multifactorial and multiple component interventions for preventing falls in older people living in the community. Cochrane database of systematic reviews. 2018;(7).

13. Deandrea S, Lucenteforte E, Bravi F, Foschi R, La Vecchia C, Negri E. Risk factors for falls in community-dwelling older people: a systematic review and meta-analysis. Epidemiology. 2010;21(5):658-68. doi: 10.1097/EDE.0b013e3181e89905. PubMed PMID: 20585256.

14. Ambrose AF, Paul G, Hausdorff JM. Risk factors for falls among older adults: A review of the literature. Maturitas. 2013;75(1):51-61. doi: 10.1016/j.maturitas.2013.02.009. PubMed PMID: WOS:000318392500008.

15. Iglesias CP, Manca A, Torgerson DJ. The health-related quality of life and cost implications of falls in elderly women. Osteoporosis international : a journal established as result of cooperation between the European Foundation for Osteoporosis and the National Osteoporosis Foundation of the USA. 2009;20(6):869-78. doi: 10.1007/s00198-008-0753-5. PubMed PMID: 18846400.

16. Tian Y, Thompson J, Buck D, Sonola L. Exploring the system-wide costs of falls in older people in Torbay: King's Fund; 2013.

17. Davis J, Robertson M, Ashe M, Liu-Ambrose T, Khan K, Marra C. International comparison of cost of falls in older adults living in the community: a systematic review. Osteoporosis international. 2010;21(8):1295-306.

18. Clegg A, Bates C, Young J, Ryan R, Nichols L, Ann Teale E, et al. Development and validation of an electronic frailty index using routine primary care electronic health record data. Age Ageing. 2016;45(3):353-60. doi: 10.1093/ageing/afw039. PubMed PMID: 26944937; PubMed Central PMCID: PMCPMC4846793.

19. Smith MI, de Lusignan S, Mullett D, Correa A, Tickner J, Jones S. Predicting Falls and When to Intervene in Older People: A Multilevel Logistical Regression Model and Cost Analysis. PloS one. 2016;11(7):e0159365. doi: <https://dx.doi.org/10.1371/journal.pone.0159365>.

20. National Institute for Health and Care Excellence. 2019 surveillance of falls in older people: assessing risk and prevention (NICE guideline CG161). National Institute for Health and Care Excellence. 2019;Published: 23 May 2019.

21. Foster C, Reilly J, Jago R, Murphy M, Skelton D, Cooper A, et al. UK Chief Medical Officers' Physical Activity Guidelines. Department of Health and Social Care. 2019.

22. Panel on Prevention of Falls in Older Persons, American Geriatrics Society, British Geriatrics Society. Summary of the updated American Geriatrics Society/British Geriatrics Society clinical practice guideline for prevention of falls in older persons. Journal of the American Geriatrics Society. 2011;59(1):148-57.

23. Sheffield Teaching Hospitals. Perfect Patient Pathway Test Bed Overview Report. Sheffield Teaching Hospitals NHS Foundation Trust, 2019.

24. Sherrington C, Fairhall NJ, Wallbank GK, Tiedemann A, Michaleff ZA, Howard K, et al. Exercise for preventing falls in older people living in the community. Cochrane database of systematic reviews. 2019;(1).

25. Lee SH, Yu S. Effectiveness of multifactorial interventions in preventing falls among older adults in the community: a systematic review and meta-analysis. International journal of nursing studies. 2020;106:103564.

26. Child S, Goodwin V, Garside R, Jones-Hughes T, Boddy K, Stein K. Factors influencing the implementation of fall-prevention programmes: a systematic review and synthesis of qualitative studies. Implementation science. 2012;7(1):91.

27. World Health Organization. World Report on Ageing and Health: World Health Organization; 2015.

28. Department of Health. Healthy lives, healthy people: Our strategy for public health in England: The Stationery Office; 2010.

29. Davis J, Robertson MC, Comans T, Scuffham P. Guidelines for conducting and reporting economic evaluation of fall prevention strategies. Osteoporosis international. 2011;22(9):2449-59.

30. Squires H, Chilcott J, Akehurst R, Burr J, Kelly MP. A systematic literature review of the key challenges for developing the structure of public health economic models. International journal of public health. 2016;61(3):289-98.

31. Salleh S, Thokala P, Brennan A, Hughes R, Dixon S. Discrete event simulation-based resource modelling in health technology assessment. Pharmacoeconomics. 2017;35(10):989-1006.

32. Pidd M. Computer simulation in management science. Fifth edition. John Wiley & Sons. 2004.

33. Public Health England. A structured literature review to identify cost-effective interventions to prevent falls in older people living in the community. Public Health England. 2018.

34. Griffiths I. Sheffield Clinical Commissioning Group Active Support and Recovery update: governing body meeting. 2015.

35. NHS England. Better Care Fund. <https://wwwenglandnhsuk/ourwork/part-rel/transformation-fund/bcf-plan/>. 2020.

36. Cameron I, Gillespie L, Robertson C, Murray G, Hill K, Cumming R, et al. Interventions for preventing falls in older people in care facilities and hospitals. Cochrane database of systematic reviews. 2012;12:CD005465-1.

37. Nyman SR, Victor CR. Older people's recruitment, sustained participation, and adherence to falls prevention interventions in institutional settings: a supplement to the Cochrane systematic review. Age and Ageing. 2011;40(4):430-6.

38. Steptoe A, Breeze E, Banks J, Nazroo J. Cohort profile: the English longitudinal study of ageing. Int J Epidemiol. 2013;42(6):1640-8. doi: 10.1093/ije/dys168. PubMed PMID: 23143611; PubMed Central PMCID: PMCPMC3900867.

39. Marmot M, Oldfield Z, Clemens S, Blake M, Phelps A, Nazroo J, et al. English Longitudinal Study of Ageing: Waves 0-6, 1998-2013 [computer file]. Colchester: UK Data Archive [distributor]. 2014;SN: 5050.

40. National Institute for Health and Care Excellence. Supporting investment in public health: Review of methods for assessing cost effectiveness, cost impact and return on investment. London: NICE. 2011.

41. Coast J, Flynn TN, Natarajan L, Sproston K, Lewis J, Louviere JJ, et al. Valuing the ICECAP capability index for older people. Social science & medicine. 2008;67(5):874-82.

42. Sekaran NK, Choi H, Hayward RA, Langa KM. Fall-associated difficulty with activities of daily living in functionally independent individuals aged 65 to 69 in the United States: a cohort study. J Am Geriatr Soc. 2013;61(1):96-100. doi: 10.1111/jgs.12071. PubMed PMID: 23311555; PubMed Central PMCID: PMCPMC3807864.

43. Fang X, Shi J, Song X, Mitnitski A, Tang Z, Wang C, et al. Frailty in relation to the risk of falls, fractures, and mortality in older Chinese adults: Results from the Beijing longitudinal study of aging. The journal of nutrition, health & aging. 2012;16(10):903-7.

44. Li G, Thabane L, Ioannidis G, Kennedy C, Papaioannou A, Adachi JD. Comparison between frailty index of deficit accumulation and phenotypic model to predict risk of falls: data from the global longitudinal study of osteoporosis in women (GLOW) Hamilton cohort. PloS one. 2015;10(3):e0120144. Epub 2015/03/13. doi: 10.1371/journal.pone.0120144. PubMed PMID: 25764521; PubMed Central PMCID: PMCPMC4357575.

45. Yardley L, Smith H. A prospective study of the relationship between feared consequences of falling and avoidance of activity in community-living older people. The Gerontologist. 2002;42(1):17-23.

46. Scheffer AC, Schuurmans MJ, Van Dijk N, Van Der Hooft T, De Rooij SE. Fear of falling: measurement strategy, prevalence, risk factors and consequences among older persons. Age and ageing. 2008;37(1):19-24.

47. Sixsmith A, Sixsmith J. Ageing in place in the United Kingdom. Ageing International. 2008;32(3):219-35.

48. Sach TH, Logan PA, Coupland CA, Gladman JR, Sahota O, Stoner-Hobbs V, et al. Community falls prevention for people who call an emergency ambulance after a fall: an economic evaluation alongside a randomised controlled trial. Age Ageing. 2012;41(5):635-41. doi: 10.1093/ageing/afs071. PubMed PMID: 22695789; PubMed Central PMCID: PMCPMC3424053.

49. Cook J. The socio‐economic contribution of older people in the UK. Working with Older People. 2011.

50. Huter K, Kocot E, Kissimova-Skarbek K, Dubas-Jakóbczyk K, Rothgang H. Economic evaluation of health promotion for older people-methodological problems and challenges. BMC health services research. 2016;16(5):328.

51. Baker LA, Cahalin LP, Gerst K, Burr JA. Productive activities and subjective well-being among older adults: The influence of number of activities and time commitment. Social Indicators Research. 2005;73(3):431-58.

52. Jenkyn KB, Hoch JS, Speechley M. How much are we willing to pay to prevent a fall? Cost-effectiveness of a multifactorial falls prevention program for community-dwelling older adults. Canadian Journal on Aging/La Revue canadienne du vieillissement. 2012;31(2):121-37.

53. Wittenberg E, Prosser LA. Disutility of illness for caregivers and families: a systematic review of the literature. Pharmacoeconomics. 2013;31(6):489-500.

54. Koopmanschap MA, van Exel NJA, van den Berg B, Brouwer WB. An overview of methods and applications to value informal care in economic evaluations of healthcare. Pharmacoeconomics. 2008;26(4):269-80.

55. Mello JdA, Macq J, Van Durme T, Cès S, Spruytte N, Van Audenhove C, et al. The determinants of informal caregivers' burden in the care of frail older persons: a dynamic and role-related perspective. Aging & mental health. 2017;21(8):838-43.

56. Kuzuya M, Masuda Y, Hirakawa Y, Iwata M, Enoki H, Hasegawa J, et al. Falls of the elderly are associated with burden of caregivers in the community. International Journal of Geriatric Psychiatry: A journal of the psychiatry of late life and allied sciences. 2006;21(8):740-5.

57. Coast J, Smith R, Lorgelly P. Should the capability approach be applied in health economics? Health economics. 2008;17(6):667-70.

58. Williams A. Intergenerational equity: an exploration of the ‘fair innings’ argument. Health economics. 1997;6(2):117-32.

59. Verguet S, Kim JJ, Jamison DT. Extended cost-effectiveness analysis for health policy assessment: a tutorial. Pharmacoeconomics. 2016;34(9):913-23.

60. Bunn F, Dickinson A, Barnett-Page E, Mcinnes E, Horton K. A systematic review of older people's perceptions of facilitators and barriers to participation in falls-prevention interventions. Ageing & Society. 2008;28(4):449-72.

61. Hwang J, Wang L, Siever J, Medico TD, Jones CA. Loneliness and social isolation among older adults in a community exercise program: a qualitative study. Aging & mental health. 2019;23(6):736-42. Epub 2018/03/16. doi: 10.1080/13607863.2018.1450835. PubMed PMID: 29543517.

62. Krol M, Brouwer W. How to estimate productivity costs in economic evaluations. Pharmacoeconomics. 2014;32(4):335-44.

63. Nyman SR, Ingram W, Sanders J, Thomas PW, Thomas S, Vassallo M, et al. Randomised controlled trial of the effect of Tai Chi on postural balance of people with dementia. Clinical interventions in aging. 2019;14:2017.

64. Kelly MP, Powell JE, Bartle N. Health needs assessment. Oxford Textbook of Global Public Health. 2015.

65. Walker D, Aedo C. Methodological issues in assessing the cost-effectiveness of interventions to improve the health of older people. Ageing Well. 127: ROUTLEDGE in association with GSE Research; 2007. p. 127-37.

66. Shiell A, Hawe P. Health promotion community development and the tyranny of individualism. Health economics. 1996;5(3):241-7.

67. Sculpher M. Subgroups and heterogeneity in cost-effectiveness analysis. Pharmacoeconomics. 2008;26(9):799-806.

68. Earnshaw SR, Richter A, Sorensen SW, Hoerger TJ, Hicks KA, Engelgau M, et al. Optimal allocation of resources across four interventions for type 2 diabetes. Medical Decision Making. 2002;22(1_suppl):80-91.

69. Pega F, Kvizhinadze G, Blakely T, Atkinson J, Wilson N. Home safety assessment and modification to reduce injurious falls in community-dwelling older adults: cost-utility and equity analysis. Injury prevention : journal of the International Society for Child and Adolescent Injury Prevention. 2016;22(6):420-6. doi: <https://dx.doi.org/10.1136/injuryprev-2016-041999>.

70. Squires H. A methodological framework for developing the structure of Public Health economic models: University of Sheffield; 2014.

71. Pfadenhauer L, Rohwer A, Burns J, Booth A, Lysdahl KB, Hofmann B, et al. Guidance for the assessment of context and implementation in health technology assessments (HTA) and systematic reviews of complex interventions: the context and implementation of complex interventions (CICI) framework. Available from: <http://www.integrate-hta.eu/downloads/>: European Union, 2016.

72. Markle-Reid M, Dykeman C, Ploeg J, Stradiotto CK, Andrews A, Bonomo S, et al. Collaborative leadership and the implementation of community-based fall prevention initiatives: a multiple case study of public health practice within community groups. BMC health services research. 2017;17(1):141.

73. Dykeman CS, Markle-Reid MF, Boratto LJ, Bowes C, Gagné H, McGugan JL, et al. Community service provider perceptions of implementing older adult fall prevention in Ontario, Canada: a qualitative study. BMC geriatrics. 2018;18(1):34.

74. Tan AC, Clemson L, Mackenzie L, Sherrington C, Roberts C, Tiedemann A, et al. Strategies for recruitment in general practice settings: the iSOLVE fall prevention pragmatic cluster randomised controlled trial. BMC medical research methodology. 2019;19(1):236.

75. Tricco AC, Thomas SM, Veroniki AA, Hamid JS, Cogo E, Strifler L, et al. Quality improvement strategies to prevent falls in older adults: a systematic review and network meta-analysis. Age and ageing. 2019;48(3):337-46. doi: <https://dx.doi.org/10.1093/ageing/afy219>.

76. Urban K, Wright PB, Hester AL, Curran G, Rojo M, Tsai PF. Evaluation of an education strategy versus usual care to implement the STEADI algorithm in primary care clinics in an academic medical center. Clinical interventions in aging. 2020;15:1059-66. doi: <http://dx.doi.org/10.2147/CIA.S256416>.

77. Mackenzie L, Beavis A-M, Tan AC, Clemson L. Systematic review and meta-analysis of intervention studies with general practitioner involvement focused on falls prevention for community-dwelling older people. Journal of aging and health. 2020:0898264320945168.

78. Nyman SR, Ballinger C. A review to explore how allied health professionals can improve uptake of and adherence to falls prevention interventions. British Journal of Occupational Therapy. 2008;71(4):141-5.

79. Li F, Harmer P, Fitzgerald K. Implementing an Evidence-Based Fall Prevention Intervention in Community Senior Centers. Am J Public Health. 2016;106(11):2026-31. doi: 10.2105/AJPH.2016.303386. PubMed PMID: 27631751; PubMed Central PMCID: PMCPMC5055774.

80. Li F, Harmer P, Stock R, Fitzgerald K, Stevens J, Gladieux M, et al. Implementing an evidence‐based fall prevention program in an outpatient clinical setting. Journal of the American Geriatrics Society. 2013;61(12):2142-9.

81. Karlsson L, Lundkvist J, Psachoulia E, Intorcia M, Ström O. Persistence with denosumab and persistence with oral bisphosphonates for the treatment of postmenopausal osteoporosis: a retrospective, observational study, and a meta-analysis. Osteoporosis International. 2015;26(10):2401-11.

82. Norheim OF, Baltussen R, Johri M, Chisholm D, Nord E, Brock D, et al. Guidance on priority setting in health care (GPS-Health): the inclusion of equity criteria not captured by cost-effectiveness analysis. Cost Effectiveness and Resource Allocation. 2014;12(1):18.

83. Barnett K, Mercer SW, Norbury M, Watt G, Wyke S, Guthrie B. Epidemiology of multimorbidity and implications for health care, research, and medical education: a cross-sectional study. The Lancet. 2012;380(9836):37-43.

84. McLennan D, Noble S, Noble M, Plunkett E, Wright G, Gutacker N. The English indices of deprivation 2019: Technical report. 2019.

85. Matchar DB, Eom K, Duncan PW, Lee M, Sim R, Sivapragasam NR, et al. A cost-effectiveness analysis of a randomized control trial of a tailored, multifactorial program to prevent falls among the community-dwelling elderly. Archives of physical medicine and rehabilitation. 2019;100(1):1-8.

86. Asaria M, Griffin S, Cookson R. Distributional Cost-Effectiveness Analysis: A Tutorial. Medical decision making. 2016;36(1):8-19. doi: 10.1177/0272989X15583266.

87. Tinetti ME, Kumar C. The patient who falls: “It's always a trade-off”. JAMA. 2010;303(3):258-66.

88. Tinetti ME, Doucette J, Claus E, Marottoli R. Risk factors for serious injury during falls by older persons in the community. Journal of the American geriatrics society. 1995;43(11):1214-21.

89. Gale CR, Westbury LD, Cooper C, Dennison EM. Risk factors for incident falls in older men and women: the English longitudinal study of ageing. BMC Geriatrics. 2018;18(1):117. doi: 10.1186/s12877-018-0806-3.

90. Chang VC, Do MT. Risk factors for falls among seniors: implications of gender. American journal of epidemiology. 2015;181(7):521-31.

91. Kim MJ, Park S, Jung Y-i, Kim S-H, Oh I-H. Exploring health-related quality of life and frailty in older adults based on the Korean Frailty and Aging Cohort Study. Quality of Life Research. 2020:1-9.

92. Hazra NC, Rudisill C, Gulliford MC. Determinants of health care costs in the senior elderly: age, comorbidity, impairment, or proximity to death? The European Journal of Health Economics. 2018;19(6):831-42.

93. Department of Health and Social Care. The NHS Constitution for England 2021. Available from: <https://www.gov.uk/government/publications/the-nhs-constitution-for-england/the-nhs-constitution-for-england>.

94. National Institute for Health and Care Excellence. NICE Citizens Council Report on Age. UK: NICE. 2003.

95. Iliffe S, Kendrick D, Morris R, Masud T, Gage H, Skelton D, et al. Multicentre cluster randomised trial comparing a community group exercise programme and home-based exercise with usual care for people aged 65 years and over in primary care. Health technology assessment (Winchester, England). 2014;18(49):vii-105. doi: <https://dx.doi.org/10.3310/hta18490>.

96. Marmot M. The Health Gap: The Challenge of an Unequal World: Bloomsbury Paperbacks; 2015.

97. Leslie WD, Morin SN, Lix LM, Martineau P, Bryanton M, McCloskey EV, et al. Fracture prediction from self-reported falls in routine clinical practice: a registry-based cohort study. Osteoporosis international : a journal established as result of cooperation between the European Foundation for Osteoporosis and the National Osteoporosis Foundation of the USA. 2019;30(11):2195-203. Epub 2019/08/03. doi: 10.1007/s00198-019-05106-3. PubMed PMID: 31372711.

98. Tinetti ME. Preventing falls in elderly persons. New England Journal of Medicine. 2003;348(1):42-9.

99. Howland J, Shankar KN, Peterson EW, Taylor AA. Savings in acute care costs if all older adults treated for fall-related injuries completed matter of balance. Injury Epidemiology. 2015;2(1):25. doi: <http://dx.doi.org/10.1186/s40621-015-0058-z>.

100. Wu T-Y, Chie W-C, Yang R-S, Kuo K-L, Wong W-K, Liaw C-K. Risk factors for single and recurrent falls: a prospective study of falls in community dwelling seniors without cognitive impairment. Preventive medicine. 2013;57(5):511-7.

101. Cohen MA, Miller J, Shi X, Sandhu J, Lipsitz LA. Prevention Program Lowered The Risk Of Falls And Decreased Claims For Long-Term Services Among Elder Participants. Health affairs. 2015;34(6):971-7. doi: <https://dx.doi.org/10.1377/hlthaff.2014.1172>.

102. Tinetti ME, Williams CS. The effect of falls and fall injuries on functioning in community-dwelling older persons. The Journals of Gerontology Series A: Biological Sciences and Medical Sciences. 1998;53(2):M112-M9.

103. Donald IP, Bulpitt CJ. The prognosis of falls in elderly people living at home. Age and Ageing. 1999;28(2):121-5.

104. Haentjens P, Magaziner J, Colón-Emeric CS, Vanderschueren D, Milisen K, Velkeniers B, et al. Meta-analysis: excess mortality after hip fracture among older women and men. Annals of internal medicine. 2010;152(6):380-90.

105. Peasgood T, Herrmann K, Kanis JA, Brazier JE. An updated systematic review of Health State Utility Values for osteoporosis related conditions. Osteoporosis international : a journal established as result of cooperation between the European Foundation for Osteoporosis and the National Osteoporosis Foundation of the USA. 2009;20(6):853-68. doi: 10.1007/s00198-009-0844-y. PubMed PMID: 19271098.

106. Hadjistavropoulos T, Delbaere K, Fitzgerald TD. Reconceptualizing the role of fear of falling and balance confidence in fall risk. Journal of aging and Health. 2011;23(1):3-23.

107. Friedman SM, Munoz B, West SK, Rubin GS, Fried LP. Falls and fear of falling: which comes first? A longitudinal prediction model suggests strategies for primary and secondary prevention. J Am Geriatr Soc. 2002;50(8):1329-35. PubMed PMID: 12164987.

108. Chang N-T, Chi L-Y, Yang N-P, Chou P. The impact of falls and fear of falling on health-related quality of life in Taiwanese elderly. Journal of community health nursing. 2010;27(2):84-95.

109. Tinetti ME, Powell L. Fear of falling and low self-efficacy: a cause of dependence in elderly persons. Journal of gerontology. 1993.

110. Delbaere K, Close JC, Mikolaizak AS, Sachdev PS, Brodaty H, Lord SR. The falls efficacy scale international (FES-I). A comprehensive longitudinal validation study. Age and ageing. 2010;39(2):210-6.

111. Kim JH, Bae SM. Association between Fear of Falling (FOF) and all-cause mortality. Arch Gerontol Geriatr. 2020;88:104017. Epub 2020/02/12. doi: 10.1016/j.archger.2020.104017. PubMed PMID: 32044524.

112. Noh HM, Roh YK, Song HJ, Park YS. Severe Fear of Falling Is Associated With Cognitive Decline in Older Adults: A 3-Year Prospective Study. J Am Med Dir Assoc. 2019;20(12):1540-7. Epub 2019/07/29. doi: 10.1016/j.jamda.2019.06.008. PubMed PMID: 31351857.

113. Pereira C, Bravo J, Raimundo A, Tomas-Carus P, Mendes F, Baptista F. Risk for physical dependence in community-dwelling older adults: The role of fear of falling, falls and fall-related injuries. International journal of older people nursing. 2020;15(3):e12310. doi: <http://dx.doi.org/10.1111/opn.12310>.

114. Eldridge S, Spencer A, Cryer C, Parsons S, Underwood M, Feder G. Why modelling a complex intervention is an important precursor to trial design: lessons from studying an intervention to reduce falls-related injuries in older people. Journal of health services research & policy. 2005;10(3):133-42.

115. Fried LP, Tangen CM, Walston J, Newman AB, Hirsch C, Gottdiener J, et al. Frailty in older adults: evidence for a phenotype. J Gerontol A Biol Sci Med Sci. 2001;56(3):M146-56. PubMed PMID: 11253156.

116. Mitnitski AB, Mogilner AJ, Rockwood K. Accumulation of deficits as a proxy measure of aging. The Scientific World Journal. 2001;1:323-36.

117. Searle SD, Mitnitski A, Gahbauer EA, Gill TM, Rockwood K. A standard procedure for creating a frailty index. BMC geriatrics. 2008;8(1):24.

118. Kojima G. Frailty as a predictor of future falls among community-dwelling older people: a systematic review and meta-analysis. Journal of the American Medical Directors Association. 2015;16(12):1027-33.

119. Kojima G, Kendrick D, Skelton DA, Morris RW, Gawler S, Iliffe S. Frailty predicts short-term incidence of future falls among British community-dwelling older people: a prospective cohort study nested within a randomised controlled trial. BMC geriatrics. 2015;15(1):155.

120. Gratza SK, Chocano-Bedoya PO, Orav EJ, Fischbacher M, Freystätter G, Theiler R, et al. Influence of fall environment and fall direction on risk of injury among pre-frail and frail adults. Osteoporosis international : a journal established as result of cooperation between the European Foundation for Osteoporosis and the National Osteoporosis Foundation of the USA. 2019;30(11):2205-15. Epub 2019/08/05. doi: 10.1007/s00198-019-05110-7. PubMed PMID: 31377914.

121. Han L, Clegg A, Doran T, Fraser L. The impact of frailty on healthcare resource use: a longitudinal analysis using the Clinical Practice Research Datalink in England. Age and Ageing. 2019;48(5):665-71.

122. Fillion V, Sirois MJ, Gamache P, Guertin JR, Morin SN, Jean S. Frailty and health services use among Quebec seniors with non-hip fractures: a population-based study using adminsitrative databases. BMC Health Serv Res. 2019;19(1):70. Epub 2019/01/27. doi: 10.1186/s12913-019-3865-z. PubMed PMID: 30683094; PubMed Central PMCID: PMCPMC6347825.

123. Shi SM, McCarthy EP, Mitchell SL, Kim DH. Predicting Mortality and Adverse Outcomes: Comparing the Frailty Index to General Prognostic Indices. Journal of General Internal Medicine. 2020;35(5):1516-22. doi: <http://dx.doi.org/10.1007/s11606-020-05700-w>.

124. Ringer TJ, Hazzan AA, Kennedy CC, Karampatos S, Patterson C, Marr S, et al. Care recipients’ physical frailty is independently associated with subjective burden in informal caregivers in the community setting: a cross-sectional study. BMC geriatrics. 2016;16(1):1-5.

125. Clark S, Shaw C, Padayachee A, Howard S, Hay K, Frakking TT. Frailty and hospital outcomes within a low socioeconomic population. QJM. 2019;112(12):907-13. doi: <http://dx.doi.org/10.1093/qjmed/hcz203>.

126. Hoogendijk EO, Afilalo J, Ensrud KE, Kowal P, Onder G, Fried LP. Frailty: implications for clinical practice and public health. Lancet. 2019;394(10206):1365-75. Epub 2019/10/15. doi: 10.1016/s0140-6736(19)31786-6. PubMed PMID: 31609228.

127. Shumway-Cook A, Ciol MA, Gruber W, Robinson C. Incidence of and risk factors for falls following hip fracture in community-dwelling older adults. Physical Therapy. 2005;85(7):648-55.

128. Kristensen MT, Foss NB, Kehlet H. Timed “up & go” test as a predictor of falls within 6 months after hip fracture surgery. Physical therapy. 2007;87(1):24-30.

129. Studenski S, Perera S, Patel K, Rosano C, Faulkner K, Inzitari M, et al. Gait speed and survival in older adults. Jama. 2011;305(1):50-8.

130. Isaranuwatchai W, Perdrizet J, Markle-Reid M, Hoch JS. Cost-effectiveness analysis of a multifactorial fall prevention intervention in older home care clients at risk for falling. BMC geriatrics. 2017;17(1):199. doi: <https://dx.doi.org/10.1186/s12877-017-0599-9>.

131. Muir SW, Gopaul K, Montero Odasso MM. The role of cognitive impairment in fall risk among older adults: a systematic review and meta-analysis. Age and ageing. 2012;41(3):299-308.

132. Li F, Harmer P. Prevalence of Falls, Physical Performance, and Dual-Task Cost While Walking in Older Adults at High Risk of Falling with and Without Cognitive Impairment. Clinical interventions in aging. 2020;15:945-52. doi: <https://dx.doi.org/10.2147/CIA.S254764>.

133. Uzoigwe CE, O'Leary L, Nduka J, Sharma D, Melling D, Simmons D, et al. Factors associated with delirium and cognitive decline following hip fracture surgery. The bone & joint journal. 2020;102-B(12):1675-81. doi: <https://dx.doi.org/10.1302/0301-620X.102B12.BJJ-2019-1537.R3>.

134. Sachs GA, Carter R, Holtz LR, Smith F, Stump TE, Tu W, et al. Cognitive impairment: an independent predictor of excess mortality: a cohort study. Annals of internal medicine. 2011;155(5):300-8.

135. Wu C-Y, Chou Y-C, Huang N, Chou Y-J, Hu H-Y, Li C-P. Cognitive impairment assessed at annual geriatric health examinations predicts mortality among the elderly. Preventive medicine. 2014;67:28-34.

136. Zhu CW, Sano M, Ferris SH, Whitehouse PJ, Patterson MB, Aisen PS. Health‐related resource use and costs in elderly adults with and without mild cognitive impairment. Journal of the American Geriatrics Society. 2013;61(3):396-402.

137. Riffin C, Van Ness PH, Wolff JL, Fried T. Family and other unpaid caregivers and older adults with and without dementia and disability. Journal of the American Geriatrics Society. 2017;65(8):1821-8.

138. Katja P, Timo T, Taina R, Tiina-Mari L. Do mobility, cognitive functioning, and depressive symptoms mediate the association between social activity and mortality risk among older men and women? European Journal of Ageing. 2014;11(2):121-30.

139. Wheatley A, Bamford C, Shaw C, Flynn E, Smith A, Beyer F, et al. Developing an Intervention for Fall-Related Injuries in Dementia (DIFRID): an integrated, mixed-methods approach. BMC Geriatr. 2019;19(1):57. Epub 2019/03/02. doi: 10.1186/s12877-019-1066-6. PubMed PMID: 30819097; PubMed Central PMCID: PMCPMC6394022.

140. Booth V, Logan P, Harwood R, Hood V. Falls prevention interventions in older adults with cognitive impairment: a systematic review of reviews. International Journal of Therapy and Rehabilitation. 2015;22(6):289-96.

141. Thibaud M, Bloch F, Tournoux-Facon C, Brèque C, Rigaud AS, Dugué B, et al. Impact of physical activity and sedentary behaviour on fall risks in older people: a systematic review and meta-analysis of observational studies. European Review of Aging and Physical Activity. 2012;9(1):5-15.

142. Pettee Gabriel K, Griswold ME, Wang W, Conway SH, Windham BG, Palta P, et al. Physical activity trajectories and subsequent fall risk: ARIC Study. Prev Med. 2019;121:40-6. Epub 2019/02/12. doi: 10.1016/j.ypmed.2019.02.007. PubMed PMID: 30742870; PubMed Central PMCID: PMCPMC6448408.

143. Gill DP, Zou GY, Jones GR, Speechley M. Injurious falls are associated with lower household but higher recreational physical activities in community-dwelling older male veterans. Gerontology. 2008;54(2):106-15.

144. Valdés-Badilla PA, Gutiérrez-García C, Pérez-Gutiérrez M, Vargas-Vitoria R, López-Fuenzalida A. Effects of Physical Activity Governmental Programs on Health Status in Independent Older Adults: A Systematic Review. J Aging Phys Act. 2019;27(2):265-75. Epub 2018/07/11. doi: 10.1123/japa.2017-0396. PubMed PMID: 29989461.

145. Moreland JD, Richardson JA, Goldsmith CH, Clase CM. Muscle weakness and falls in older adults: a systematic review and meta‐analysis. Journal of the American Geriatrics Society. 2004;52(7):1121-9.

146. Stubbs B, Schofield P, Binnekade T, Patchay S, Sepehry A, Eggermont L. Pain is associated with recurrent falls in community-dwelling older adults: evidence from a systematic review and meta-analysis. Pain Medicine. 2014;15(7):1115-28.

147. Park H, Satoh H, Miki A, Urushihara H, Sawada Y. Medications associated with falls in older people: systematic review of publications from a recent 5-year period. European journal of clinical pharmacology. 2015;71(12):1429-40.

148. Richardson K, Bennett K, Kenny RA. Polypharmacy including falls risk-increasing medications and subsequent falls in community-dwelling middle-aged and older adults. Age and ageing. 2014;44(1):90-6.

149. Chiarelli PE, Mackenzie LA, Osmotherly PG. Urinary incontinence is associated with an increase in falls: a systematic review. Australian Journal of Physiotherapy. 2009;55(2):89-95.

150. Reed-Jones RJ, Solis GR, Lawson KA, Loya AM, Cude-Islas D, Berger CS. Vision and falls: a multidisciplinary review of the contributions of visual impairment to falls among older adults. Maturitas. 2013;75(1):22-8.

151. Kvelde T, McVeigh C, Toson B, Greenaway M, Lord SR, Delbaere K, et al. Depressive symptomatology as a risk factor for falls in older people: systematic review and meta‐analysis. Journal of the American Geriatrics Society. 2013;61(5):694-706.

152. Lim YM, Sung MH. Home environmental and health‐related factors among home fallers and recurrent fallers in community dwelling older K orean women. International journal of nursing practice. 2012;18(5):481-8.

153. Donald IP. Housing and health care for older people. Age and ageing. 2009;38(4):364-7.

154. World Health Organization. Global age-friendly cities: A guide: World Health Organization; 2007.

155. Wilson N, Kvizhinadze G, Pega F, Nair N, Blakely T. Home modification to reduce falls at a health district level: Modeling health gain, health inequalities and health costs. PloS one. 2017;12(9):e0184538.

156. Li W, Keegan TH, Sternfeld B, Sidney S, Quesenberry Jr CP, Kelsey JL. Outdoor falls among middle-aged and older adults: a neglected public health problem. American journal of public health. 2006;96(7):1192-200.

157. Day R. Local environments and older people's health: dimensions from a comparative qualitative study in Scotland. Health & place. 2008;14(2):299-312.

158. Zhou P, Grady SC, Chen G. How the built environment affects change in older people's physical activity: A mixed-methods approach using longitudinal health survey data in urban China. Social Science & Medicine. 2017;192:74-84.

159. Trevisan C, Rizzuto D, Maggi S, Sergi G, Wang HX, Fratiglioni L, et al. Impact of Social Network on the Risk and Consequences of Injurious Falls in Older Adults. J Am Geriatr Soc. 2019;67(9):1851-8. Epub 2019/06/27. doi: 10.1111/jgs.16018. PubMed PMID: 31241183.

160. Petersen N, König HH, Hajek A. The link between falls, social isolation and loneliness: A systematic review. Arch Gerontol Geriatr. 2020;88:104020. Epub 2020/02/06. doi: 10.1016/j.archger.2020.104020. PubMed PMID: 32018091.

161. Howland J, Lachman ME, Peterson EW, Cote J, Kasten L, Jette A. Covariates of fear of falling and associated activity curtailment. The Gerontologist. 1998;38(5):549-55.

162. Cotterell N, Buffel T, Phillipson C. Preventing social isolation in older people. Maturitas. 2018;113:80-4.

163. National Institute for Health and Care Excellence. Guideline scope: Falls in older people: assessing risk and prevention (update). In: Excellence NIfHaC, editor. 2022.

164. Sport Industry Research Centre. Dance to Health 'Phase 1 roll-out [test and learn]' evaluation. Sheffield Hallam University. 2019.
