# Supplementary material for: Economic model of community-based falls prevention: seeking methodological solutions in evaluating the efficiency and equity of UK guideline recommendations
Source: BMC Geriatr. 2023 Mar 30;23:187. doi: 10.1186/s12877-023-03916-z (PMC10061399; doi:10.1186/s12877-023-03916-z)
Supplement: Supplementary file 2 — Additional file 2: Appendix B. Model parameterisation. [file 12877_2023_3916_MOESM2_ESM.docx]

# Economic model of community-based falls prevention: seeking methodological solutions in evaluating the efficiency and equity of UK guideline recommendations

**Appendix B: Model parameterisation**

**Authors:**

Dr Joseph Kwon,^1*^ [joseph.kwon@phc.ox.ac.uk](mailto:joseph.kwon@phc.ox.ac.uk); ORCID 0000-0002-2860-7280

Dr Hazel Squires,^2^ [h.squires@sheffield.ac.uk](mailto:h.squires@sheffield.ac.uk); ORCID 0000-0001-8467-0471

Professor Tracey Young,^2^ [t.a.young@sheffield.ac.uk](mailto:t.a.young@sheffield.ac.uk); ORCID 0000-0002-0754-7223

^1^ Nuffield Department of Primary Care Health Sciences, University of Oxford, Radcliffe Primary Care Building, Radcliffe Observatory Quarter, Woodstock Road, Oxford, England, OX2 6GG

^2^ School of Health and Related Research, University of Sheffield, Regent Court (ScHARR), 30 Regent Street, Sheffield, England, S1 4DA

* Corresponding author

**Conflict of interest:** The authors declare that they have no competing interests.

**Funding:** Dr Joseph Kwon was supported by the Wellcome Trust [108903/B/15/Z] for PhD studentship.

**Acknowledgement:** We would like to thank Dr Matthew Franklin at the School of Health and Related Research, University of Sheffield, for kindly reviewing the manuscript and offering detailed feedback.

# [Content](#_Toc116837151)s

[B1 Outline 4](#_Toc116837152)

[B2 Key data sources 4](#_Toc116837153)

[B2.1 English Longitudinal Study of Ageing 4](#_Toc116837154)

[B2.2 Falls prevention randomised controlled trials 5](#_Toc116837155)

[B3 Model entry and baseline characteristics 7](#_Toc116837156)

[B3.1 Demographics and residence 7](#_Toc116837157)

[B3.2 Socioeconomic status 8](#_Toc116837158)

[B3.3 Baseline falls history 9](#_Toc116837159)

[B3.4 Baseline frailty 10](#_Toc116837160)

[B3.5 Covariates estimated at baseline 13](#_Toc116837161)

[B4 Outcomes estimated at baseline 17](#_Toc116837162)

[B4.1 Health utilities 17](#_Toc116837163)

[B4.2 Productivity level 19](#_Toc116837164)

[B4.3 Social wellbeing 21](#_Toc116837165)

[B4.4 Comorbidity care costs 22](#_Toc116837166)

[B5 Falls prevention strategy 28](#_Toc116837167)

[B5.1 Intervention overview 28](#_Toc116837168)

[B5.2 Intervention access conditions 32](#_Toc116837169)

[B5.3 Intervention resource use and cost 37](#_Toc116837170)

[B5.4 Intervention efficacy 46](#_Toc116837171)

[B6 Mortality risks and non-fatal falls epidemiology 48](#_Toc116837172)

[B6.1 Fatal falls and other-cause mortality 48](#_Toc116837173)

[B6.2 Non-fatal falls 50](#_Toc116837174)

[B6.3 Acute QALY loss due to falls 53](#_Toc116837175)

[B6.4 Economic consequences of falls 54](#_Toc116837176)

[B7 Dynamic transitions 56](#_Toc116837177)

[B7.1 Frailty progression 56](#_Toc116837178)

[B7.2 Long-term care admission and cost 57](#_Toc116837179)

[B7.3 Updated covariates and outcomes 59](#_Toc116837180)

[B8 Model validation 62](#_Toc116837181)

[B8.1 Face validity 62](#_Toc116837182)

[B8.2 Internal validity 62](#_Toc116837183)

[B8.3 External validity 62](#_Toc116837184)

[B9 Model analysis methods 65](#_Toc116837185)

[B9.1 Parameter distributions for probabilistic sensitivity analysis 65](#_Toc116837186)

[References 67](#_Toc116837187)

# B1 Outline

Appendix B parameterises the falls prevention economic model. This builds on the conceptual model in Appendix A and constitutes Phase (D) of the model development and validation process in the public health modelling framework [1]. A key part of Phase (D) is the systematic review and methodological appraisal of previous falls prevention models which has been published separately [2, 3].

Section B2 introduces two key data sources for model parameterisation, namely the English Longitudinal Survey of Ageing (ELSA) and a systematic review of community-based falls prevention RCTs. Sections B3 to B7 describe the parameterisation of each model component. They are arranged according to the sequence of simulation steps: see Figure 1 in the main manuscript for the model representation diagram which shows the sequence from step 1 of ‘Model entry’ to step 9 of ‘Update covariates and outcomes’. Section B8 describes the methods and results of model validation. Section B9 presents data inputs to probabilistic sensitivity analysis as part of model analysis methods.

In the interest of space, not all tables and texts from the parameterisation process are reported in this document. They are noted as ‘not reported here’ and are available from the authors upon request.

# B2 Key data sources

## B2.1 English Longitudinal Study of Ageing

ELSA was chosen as a key data source because: (a) It follows a nationally representative cohort of adults aged 50+ in England and was deemed representative of the Sheffield target population by the commissioners. (b) It collects a wide range of health, demographic, socioeconomic, lifestyle and environmental variables relevant to older populations (e.g., informal care receipt) and tracks their longitudinal trajectories via individual identifiers [4, 5]. (c) It contains falls incidence and falls prevention service use data with which falls risk equations and service use patterns can be estimated using individual-level characteristics.

To date (January 2022), nine two-year interim ELSA surveys have been conducted starting from Wave 1 in 2002 to Wave 9 in 2018. The anthropometric (e.g., weight, blood pressure) and physical capacity (e.g., walking speed) variables were collected by nurse visits on every even-number waves [6]. Though the Wave 1 cohort was recruited solely from community-dwelling persons, those admitted to residential or nursing homes were still interviewed at subsequent waves [7]. Among the nine waves, the Wave 4 data and the next follow-up Wave 5 were used for parameterisation because:

1. Wave 4 contains the most comprehensive data regarding falls and falls prevention: (i) It is the only wave with information on falls history in the previous *one year* rather than two years of survey interim. This variable is important because the falls prevention pathway emphasises falls history in the previous 12 months [8]. (ii) It is the only wave with information on fear of falling perceived while walking, a key causal variable in the conceptual model. (iii) Only Waves 2, 4 and 8 contain self-reported data on previous contact with falls prevention services: whether doctor/nurse tried to understand cause of fall; whether doctor/nurse tested balance and strength; and whether doctor/nurse recommended further risk assessments. These variables are important for estimating a plausible level of falls prevention access under UC.
2. The nurse-measured anthropometric and physical capacity variables are available only in even-numbered waves. In the absence of Wave 10 data, this ruled out the use of Wave 8 as the baseline. Wave 4 was then preferred over Wave 6 for the first reason.

Supplementary information on using ELSA for parameterisation (e.g., the original data labels and methods for handling missing observations) is available from the authors upon reasonable request.

## B2.2 Falls prevention randomised controlled trials

RCTs provide information on intervention recipient characteristics (from inclusion/exclusion criteria and sample characteristics), implementation level, resource use, and efficacy. Falls prevention interventions are characterised by substantial heterogeneity in component, mode of delivery, personnel, intensity, and duration; such heterogeneity is present across geographically defined care settings [9]. This limits the utility of secondary syntheses of RCT evidence from different jurisdictions. Therefore, this model relies on non-synthesised UK RCTs to improve generalisability to UK health economies.

A pragmatic approach was pursued in identifying the relevant RCTs, as shown in Table B1. This included a *de novo* systematic review conducted in July 2019: the search strategy and detailed inclusion/exclusion criteria are available from the authors upon request. The Cochrane review by Gillespie and colleagues remains the most comprehensive review of community-based falls prevention RCTs, covering all intervention types published before March 2012 [10]. This was updated twice: for multifactorial and multiple-component interventions published before June 2017 [11]; and for exercise interventions published before May 2018 [12]. These were supplemented by forward citation searching of the three Cochrane reviews for the period between 2019 and June 2020.

| **Table B1** Sources of community-based falls prevention randomised controlled trials. | | |
| --- | --- | --- |
| **Review** | **Search methods^1^** | **Search results** |
| Gillespie (2012): Cochrane systematic review of all community-based falls prevention [10] | *Intervention type*: all types  *Exclusion*: stroke and Parkinson’s disease rehabilitation  *Period*: database inception to Feb-Mar 2012 | # of trials: 159; of which 63 were not included in Hopewell (2018) and Sherrington (2019) |
| *De novo* systematic review conducted in July 2019 for period 2012-2018^2^ | *Intervention type*: all types  *Exclusion*: stroke and Parkinson’s disease rehabilitation  *Period*: 1^st^ Jan 2012 to 31^st^ Dec 2018 | # of trials: 61 not included in Gillespie (2012), Hopewell (2018) and Sherrington (2019) |
| Hopewell (2018): Cochrane systematic review of multifactorial and multiple-component community-based falls prevention [11] | *Intervention type*: multifactorial and multiple-component interventions  *Exclusion*: stroke and Parkinson’s disease rehabilitation  *Period*: Jan 2012 to June 2017 for studies not included in (Gillespie et al., 2012) | # of trials: 62; of which 17 were not included in Gillespie (2012) |
| Sherrington (2019) – Cochrane systematic review of community-based falls prevention exercise [12] | *Intervention type*: exercise  *Exclusion*: exercises for patients with stroke, Parkinson’s disease, multiple sclerosis, dementia, hip fracture and severe visual impairment  *Period*: Jan 2012 to May 2018 for studies not included in (Gillespie et al., 2012) | # of trials: 106; of which 54 were not included in Gillespie (2012) |
| Citation search of Cochrane systematic reviews | *Intervention type*: all types  *Exclusion*: stroke and Parkinson’s disease rehabilitation  *Period:* 1^st^ Jan 2019 to 30^th^ Jun 2020 | # of trials: 21 not included in Gillespie (2012), Hopewell (2018), Sherrington (2019) and *de novo* systematic review |
| **Total** |  | **# of trials: 312** |
| ^1^ All systematic reviews except citation search covered the same databases: Cochrane Library; Medline; Embase; CINAHL.  ^2^ The search strategy and detailed inclusion and exclusion criteria are available from the authors upon request. | | |

This process identified 312 RCTs; of these, 45 were set in the UK. Table B2 describes 11 UK-based RCTs that were aligned with current or recommended practice conceptualised in Appendix A. Five evaluated multifactorial interventions, while six evaluated exercise interventions; they are organised in Table B2 by target population characteristics (pathway, cognitive status, and falls risk). Where multiple RCTs targeted similar populations – e.g., Close (2009) and Davison (2005) targeted cognitively intact reactive patients – one of them was chosen for parameterisation rather than pooling both. Hence, for example, Close (2009) was chosen over Davison (2005) because the latter incorporated history of falls as an eligibility criterion for the reactive pathway, a criterion not mentioned in NICE CG161 [8]. See Section B5 for details on the RCTs finally used for parameterisation.

| **Table B2** UK community-based randomised controlled trials of falls prevention interventions aligned with current or recommended practice in conceptual model. | | | | |
| --- | --- | --- | --- | --- |
| **Intervention type** | **Target population** | **Reference** | **Description** | **N** |
| Multifactorial intervention (n=5) | Reactive | Close (2009) [13] | For cognitively intact persons aged 65+ admitted to A&E for fall; medical and OT assessment and treatment at geriatric day hospitals | 2 |
|  |  | Davison (2005) [14] | For cognitively intact persons aged 65+ admitted to A&E for fall *and* has history of falls; medical, PT and OT assessment and treatment at general hospitals |  |
|  | Reactive & CI-friendly | Shaw (2003) [15] | For cognitively impaired persons aged 65+ admitted to A&E for fall; medical, PT and OT assessment and intervention | 1 |
|  | Proactive | Conroy (2010) [16] | For cognitively intact persons aged 70+ screened to be high falls risk by fall history or FRAT; medical, PT and OT assessment and intervention at geriatric day hospitals | 2 |
|  |  | Spice (2009) [17] | For cognitively intact persons aged 65+ with recurrent falls history *and* no A&E admission for most recent fall; medical, PT and OT assessment and intervention at multidisciplinary secondary care clinic^1^ |  |
| Exercise (n=6) | General-risk group | Iliffe (2014) [18] | For cognitively intact and independently mobile persons aged 65+ with less than 3 falls in past year; 24-week FaME group exercise + Otago home exercise^2^ | 1 |
|  | High-risk group | Skelton (2005) [19] | For cognitively intact women aged 65+ with 3+ falls in past year; 36-week FaME group exercise + Otago home exercise | 3 |
|  |  | Stanmore (2019) [20] | For persons aged 55+ living in sheltered housing facilities; 12-week FaME/Otago-based Exergames at home |  |
|  |  | Clegg (2014) [21] | Feasibility trial (n=84); for frail persons (mean age 79) without severe dementia receiving case manager care, housebound or attending older outpatient clinics; 12-week progressive exercise for basic mobility skills |  |
|  | CI-friendly | Lamb (2018) [22] | For independently mobile persons (mean age 78) with mild or moderate dementia; 16-week aerobic and strength group exercise + continued physical activity support | 2 |
|  |  | Nyman (2020) [23] | For persons (mean age 78) with mild or moderate dementia able to participate in physical exercise and their informal caregivers; 20-week group- and home-based Tai Chi + behavioural change support |  |
| **Abbreviation:** CI-friendly: cognitively impaired-friendly; FaME: Falls Management Exercise; FRAT: Falls Risk Assessment Tool; HAM: home assessment and modification; MA fall: fall requiring medical attention; OT: occupational therapy/therapist; PT: physiotherapy/physiotherapist  ^1^ The study contained an additional intervention arm for nurse-led multifactorial risk assessment (without active treatments) in primary care setting. This intervention did not produce a statistically significant reduction in the numbers of falls and fallers relative to usual care.  ^2^ The study contained an additional intervention arm for home-based Otago exercise supervised by peer mentors. This intervention did not produce a statistically significant reduction in the numbers of falls relative to usual care**.** | | | | |

# B3 Model entry and baseline characteristics

Simulated individuals are assigned the following baseline characteristics upon model entry: demographics and residence (Section B3.1); SES (B3.2); falls history (B3.3); frailty (B3.4); and additional key covariates (B3.5).

## B3.1 Demographics and residence

The size of the target population in Sheffield during the 40-year period 2021-2060 by age (60+), sex, and residence – community-dwelling vs. institutionalised – was estimated from five datasets: (1) ONS dataset on population size by age and sex disaggregated to local authority level in mid-2019 [24]; (2) ONS lifetables for annual mortality risk by age and sex based on data in 2016-2018 period [25]; (3) ONS projections on population size by five-year age group and sex at local authority level for period 2018-2043 based on demographic, mortality and migration patterns during the 2014-2018 period [26]; (4) NHS Digital estimate of the average institutionalisation rate for the Sheffield population aged 65+ at the end of the financial year 2018-19 [27]; and (5) ELSA Wave 4 for the relative odds of being institutionalised by five-year age group and sex for the English population aged 60+.

Annual mortality risks stratified by age and sex were applied twice to the 2019 Sheffield population aged 60+ to estimate the population size by age (now 62+) and sex in 2021. The projected sizes of the population aged 60 and 61 were added to form a Sheffield cohort aged 60+ in 2021. Because the projected sizes were reported in five-year age groups, the share of those aged 60 in that age group was estimated from the ONS 2019 population data [24]. According to the NHS Digital report, at the end of the financial year 2018-19 there were 1,580 Sheffield residents aged 65+ living in nursing homes or residential care facilities supported by the local authority [27]. This constituted 1.67% of the total Sheffield population aged 65+. However, this number does not include individuals living in private LTC facilities. According to the Public Health England (PHE) model, 43.6% of new LTC admissions for hospitalised falls were privately funded [28]. If this percentage applies to LTC admissions for any cause, then the percentage of the Sheffield population aged 65+ living in LTC would be 2.96%.

In comparison, the ELSA data appear to underestimate the prevalence of institutionalisation in the older population. According to a variable that recorded where the survey interview took place, only 0.9% of Wave 4 interviews were recorded to have taken place in an institution. Nevertheless, the ELSA information on the degree of variation in institutionalisation rate by age group and sex was assumed to apply to the model population. Hence, the odds ratios of being institutionalised for each age and sex subgroup relative to males aged 65-69 were estimated from ELSA Wave 4 (not reported here). The subgroup-specific rates that produced a weighted average rate of 2.96% were then estimated. The multipliers of one minus subgroup-specific institutionalisation rates were then applied to the all-residence population to estimate the numbers of community-dwelling persons in each age and sex subgroup. The first three columns of Table B3 show the number of community-dwelling persons in Sheffield by age and sex for the initial cohort aged 60+ in 2021 (n=125,244). According to the overall sex ratio for the initial cohort across all ages, 46.5% of the individuals were assigned to be male. The age distribution for each sex was then assigned by the percentage of people of the same sex in each age (i.e., the parenthesised values in columns two and three of Table B3).

| **Table B3** Community-dwelling Sheffield population for period 2021-2060: (a) initial cohort aged 60 and over in 2021; (b) new entry cohorts aged 60 between 2022 and 2060. | | | | |
| --- | --- | --- | --- | --- |
| **(a) Initial cohort size by age and sex (% of sex total)** | | | **(b) New entry cohort size by year** | |
| **Age** | **Male** | **Female** | **Year** | **Number** |
| 60 | 3122 (5.35) | 3234 (4.84) | 2022 | 6527 |
| 61 | 3005 (5.15) | 3120 (4.66) | 2023 | 6671 |
| 62 | 2889 (4.95) | 3022 (4.52) | 2024 | 6794 |
| 63 | 2933 (5.03) | 2887 (4.32) | 2025 | 6902 |
| 64 | 2763 (4.73) | 2732 (4.08) | 2026 | 7011 |
| 65 | 2532 (4.34) | 2648 (3.96) | 2027 | 7084 |
| 66 | 2445 (4.19) | 2593 (3.88) | 2028 | 7163 |
| 67 | 2399 (4.11) | 2590 (3.87) | 2029 | 7247 |
| 68 | 2390 (4.10) | 2522 (3.77) | 2030 | 7278 |
| 69 | 2189 (3.75) | 2465 (3.69) | 2031 | 7272 |
| 70 | 2188 (3.75) | 2421 (3.62) | 2032 | 7185 |
| 71 | 2202 (3.77) | 2408 (3.60) | 2033 | 6988 |
| 72 | 2297 (3.94) | 2456 (3.67) | 2034 | 6743 |
| 73 | 2460 (4.22) | 2655 (3.97) | 2035 | 6503 |
| 74 | 2511 (4.30) | 2804 (4.19) | 2036 | 6229 |
| 75 | 1900 (3.26) | 2311 (3.46) | 2037 | 6021 |
| 76 | 1889 (3.24) | 2279 (3.41) | 2038 | 5920 |
| 77 | 1956 (3.35) | 2226 (3.33) | 2039 | 5970 |
| 78 | 1654 (2.83) | 1946 (2.91) | 2040 | 6094 |
| 79 | 1441 (2.47) | 1782 (2.66) | 2041 | 6220 |
| 80 | 1270 (2.18) | 1588 (2.37) | 2042 | 6329 |
| 81 | 1374 (2.35) | 1600 (2.39) | 2043 | 6421 |
| 82 | 1233 (2.11) | 1606 (2.40) | 2044 | 6448 |
| 83 | 1144 (1.96) | 1562 (2.34) | 2045 | 6475 |
| 84 | 1059 (1.81) | 1337 (2.00) | 2046 | 6502 |
| 85 | 907 (1.55) | 1238 (1.85) | 2047 | 6530 |
| 86 | 807 (1.38) | 1157 (1.73) | 2048 | 6557 |
| 87 | 692 (1.19) | 1009 (1.51) | 2049 | 6585 |
| 88 | 505 (0.86) | 896 (1.34) | 2050 | 6612 |
| 89 | 453 (0.78) | 781 (1.17) | 2051 | 6640 |
| 90+^1^ | 1751 (3.00) | 3008 (4.50) | 2052 | 6668 |
| **Total by sex** | 58,359 (100) | 66,885 (100) | 2053 | 6696 |
| **Total** | 125,244 (46.5% male) | | 2054 | 6724 |
|  |  |  | 2055 | 6752 |
|  |  |  | 2056 | 6780 |
|  |  |  | 2057 | 6809 |
|  |  |  | 2058 | 6837 |
|  |  |  | 2059 | 6866 |
|  |  |  | 2060 | 6895 |
|  |  |  | **Total** | 259,950 (48.6% male) |
| ^1^ No disaggregated data by integer age were available for those aged 90 and over from the Office for National Statistics data [24] or the English Longitudinal Study in Ageing. | | | | |

The sizes of incoming cohorts aged 60 were estimated using the ONS projections for each year between 2022 and 2043 [26], shown in the last two columns of Table B3. For the period after 2043, the average sex-specific annual growth rates over the period 2018-2043 (0.36% for male and 0.48% for female) were assumed to hold until 2060. Each new cohort entered the model at the start of each cycle, and 48.6% of them were assigned male according to the average sex ratio reported by ONS [26].

## B3.2 Socioeconomic status

The SES characteristic of interest in the conceptual model was the LSOA-level deprivation variable. But this variable was not accessible, and an alternative SES characteristic was sought from ELSA. The conceptual model had also noted further SES factors serving as key causal variables: education; household wealth; and subjective report of financial difficulty. For education, ELSA Wave 4 contained information on the highest qualification obtained by the respondent. Three categories were formed: (1) university degree; (2) higher education below degree or high school qualification; and (3) below high school qualification, no qualification or foreign qualification. For household wealth, the composite measure used in a previous analysis of ELSA was constructed [29]. This incorporated total value of savings, property, and business. Individuals were then divided into wealth quartiles. For self-reported financial difficulty, an ELSA variable measured the frequency of financial need as ‘Never’, ‘Rarely’, ‘Sometimes’, ‘Often’, and ‘Most of the time’.

From the three SES variables – education (3 categories), wealth (4) and self-reported financial difficulty (5) – a composite score was computed by summing the indicator levels. The score ranged between 3 and 12; higher score indicated greater deprivation. Individuals were then divided into quartiles for this composite measure. Using ELSA, proportions of social quartiles for each sex and five-year age subgroups were estimated, as shown in Table B4. The proportions were applied to individuals by age group and sex at model entry. For new cohorts aged 60, the proportions for age 60-64 were applied. Once assigned, individuals’ SES quartiles were assumed invariant over time. The discrete numbers of the categorical SES variable produced uneven quartile sizes. The varying prevalence across age in Table B4 reflects the heterogeneous mortality risks across quartiles: the third and fourth quartiles increasingly occupied smaller proportions of older subgroups due to their shorter life expectancy.

| **Table B4** Proportions of SES quartiles by five-year age group and sex in ELSA. | | | | |
| --- | --- | --- | --- | --- |
| % of age and sex subgroup | **Most privileged quartile** | **2^nd^ quartile** | **3^rd^ quartile** | **Most deprived quartile** |
| ***Male*** |  |  |  |  |
| Age 60-64 | 42.2 | 18.4 | 27.7 | 11.7 |
| Age 65-69 | 44.6 | 19.1 | 26.3 | 10.0 |
| Age 70-74 | 39.9 | 20.5 | 28.0 | 11.6 |
| Age 75-79 | 39.9 | 20.2 | 29.4 | 10.5 |
| Age 80-84 | 42.5 | 21.7 | 26.3 | 9.5 |
| Age 85-89 | 40.4 | 28.9 | 26.9 | 3.8 |
| Age 90+ | 54.9 | 20.9 | 16.5 | 7.7 |
| All age groups | 42.0 | 20.1 | 27.4 | 10.5 |
| ***Female*** |  |  |  |  |
| Age 60-64 | 37.5 | 17.6 | 30.9 | 14.0 |
| Age 65-69 | 38.1 | 17.5 | 31.6 | 12.8 |
| Age 70-74 | 32.2 | 19.1 | 33.4 | 15.3 |
| Age 75-79 | 29.8 | 22.2 | 33.5 | 14.5 |
| Age 80-84 | 31.6 | 22.5 | 36.1 | 9.8 |
| Age 85-89 | 34.5 | 22.5 | 35.2 | 7.8 |
| Age 90+ | 40.1 | 26.2 | 27.9 | 5.8 |
| All age groups | 34.7 | 19.5 | 32.6 | 13.2 |
| **Abbreviation:** ELSA: English Longitudinal Study of Ageing; SES: socioeconomic status | | | | |

## B3.3 Baseline falls history

After age, sex and SES quartile, individuals were assigned their baseline falls history. In ELSA Wave 4, participants were asked: (i) whether they had fallen down last year; (ii) number of times they had fallen down last year; and (iii) whether they were injured seriously enough from the fall to need medical treatment. From these three variables five falls history types were formed: (1) no falls history; (2) history of single fall not requiring medical attention (non-MA fall); (3) recurrent non-MA falls history; (4) single MA fall history; and (5) history of recurrent falls with at least one MA fall. Note that it was not possible to discern from ELSA whether a person experienced one or multiple MA fall(s). Table B5 shows the proportions of falls history type for men in the most privileged SES quartile by age group and for women in the most deprived SES quartile. The full set of proportions is not reported here but is available from the authors upon request.

| **Table B5** Proportions of baseline falls history types by age group, sex and SES quartile in ELSA: sample display for male in most privileged SES quartile and female in most deprived SES quartile. | | | | | |
| --- | --- | --- | --- | --- | --- |
|  | No falls history | Single non-MA fall history | Recurrent non-MA falls history | Single MA fall history | Recurrent falls with 1+ MA fall |
| ***Male – Most privileged SES quartile*** | | | | | |
| Age 60-64 | 83.5 | 9.1 | 5.5 | 1.2 | 0.7 |
| Age 65-69 | 83.2 | 6.7 | 6.4 | 2.2 | 1.5 |
| Age 70-74 | 77.7 | 10.9 | 5.5 | 3.4 | 2.5 |
| Age 75-79 | 73.3 | 10.6 | 9.3 | 4.6 | 2.2 |
| Age 80-84 | 68.4 | 13.8 | 11.3 | 3.9 | 2.6 |
| Age 85-89 | 61.9 | 11.4 | 14.3 | 3.8 | 8.6 |
| Age 90+ | 61.5 | 12.0 | 22.0 | 0.5 | 4.0 |
| ***Female – Most deprived SES quartile*** | | | | | |
| Age 60-64 | 72.0 | 11.4 | 8.7 | 3.9 | 4.0 |
| Age 65-69 | 65.5 | 12.8 | 9.9 | 5.4 | 6.4 |
| Age 70-74 | 71.3 | 11.7 | 7.8 | 5.7 | 3.5 |
| Age 75-79 | 66.0 | 11.8 | 11.7 | 4.6 | 5.9 |
| Age 80-84 | 51.4 | 11.8 | 11.8 | 14.7 | 10.3 |
| Age 85-89 | 52.9 | 11.8 | 17.6 | 5.9 | 11.8 |
| Age 90+ | 69.5 | 10.0 | 10.0 | 0.5 | 10.0 |
| **Abbreviation:** ELSA: English Longitudinal Study of Ageing; MA fall: fall requiring medical attention; SES: socioeconomic status | | | | | |

## B3.4 Baseline frailty

Multivariate frailty index was conceptualised as a key causal variable in Appendix A. Such index was constructed from ELSA, with care taken to ensure that it is broadly consistent in characteristics with indices previously used in frailty and falls prevention research.

Table B6 shows the characteristics and component items of four frailty indices used in literature: electronic frailty index (eFI) [30]; Beijing Longitudinal Study of Aging frailty index (BLSA FI) [31, 32]; Global Longitudinal Study of Osteoporosis in Women frailty index (GLOW FI) [33, 34]; and ProAct65+ trial frailty index [35]. Their component items comprised eight categories: chronic diseases; sensory/physical impairments and geriatric syndromes; cognitive impairment; subjective symptoms and health; lifestyle risk factors; activity limitation; healthcare contact; and social. The new frailty index was constructed to cover all categories. It also met the established criteria for frailty index construction (e.g., at least 30 deficit items) [36]. It also followed the stakeholder suggestion that the index contain falls risk factors highlighted by NICE CG161 including: gait deficit; balance deficit; mobility impairment; visual impairment; cognitive impairment; urinary incontinence; and environmental hazards [8]. All factors were included except environmental hazards. The final index contained 52 items as shown in the last column of Table B6. The number of deficits per individual was divided by the total possible number (52) to derive the index score.

The score ranged between 0 and 0.615 and had mean of 0.11 (SD 0.09) for men and 0.13 (SD 0.10) for women. These were slightly lower than the sex-specific mean values for eFI of 0.13 (SD 0.09) for men and 0.15 (SD 0.10) for women [30]. This is to be expected since the ELSA population is younger (aged 60+) than the eFI population (65+).

Previous studies took different approaches for establishing severity categories based on index scores. For example, eFI scores were divided into mild frailty (eFI >0.12-0.24), moderate frailty (eFI >0.24-0.36) and severe frailty (eFI >0.36) relative to fit reference category (eFI 0-0.12) at the 50^th^, 85^th^ and 97^th^ percentile eFI values, respectively. This study took this approach and established the cut-off levels for Fit, Mild, Moderate and Severe frailties at the 50^th^, 85^th^ and 97^th^ percentile values. The resulting index score ranges for each category were 0-0.10 for Fit, >0.10-0.23 for Mild, >0.23-0.37 for Moderate and >0.37 for Severe.

For parameterisation, the frailty scores were multiplied by 100 to range 0-100. A visual plot showed that the scores followed a lognormal distribution. The mean and SD for the lognormal distribution were obtained for each of the 280 subgroups delineated by age group (7 categories), sex (2), social deprivation quartile (4), and falls history (5), and similarly for the 40 subgroups within each new cohort aged 60. The frailty parameters for the initial cohort’s 280 subgroups are not reported here but are available upon request from authors. Individuals were assigned baseline frailty scores and associated frailty categories at model entry.

| **Table B6** Characteristics of multivariate frailty indices used in previous frailty and falls prevention studies and in this study. | | | | | |
| --- | --- | --- | --- | --- | --- |
|  | **eFI [30]** | **BLSA FI [31, 32]** | **GLOW FI [33, 34]** | **ProAct65+ FI^1^ [35]** | **This study** |
| Country | UK | China | Canada | England | England |
| Data source | Electronic health records | Cohort survey | Cohort survey | Cohort survey | Cohort survey |
| Total # of items | 36 | 33^2^ | 34 | 40 | 52 |
| Mean (SD) | Men: 0.13 (0.09)  Women: 0.15 (0.10) | Men: 0.11 (0.10)  Women: 0.14 (0.12) | Women only: 0.24 (0.13) | Both sex: 0.16 (0.11) | Men: 0.11 (0.09)  Women: 0.13 (0.10) |
| Severity categories | [Fit] 0-0.12 (50%)  [Mild] >0.12-0.24 (35%)  [Moderate] >0.24-0.36 (12%)  [Severe] >0.36 (3%) | [1] 0-0.03  [2] >0.03-0.10  [3] >0.10-0.20  [4] >0.20-0.50  [5] >0.50 | [Robust] 0-0.20 (43.9%)  [Prefrail] >0.20-0.35 (34.1%)  [Frail] >0.35 (22.1%) | [Non-frail] 0-<0.25 (81.5%)  [Frail] >=0.25 (18.5%) | [Fit] 0-0.10 (50%)  [Mild] >0.10-0.23 (35%)  [Moderate] >0.23-0.37 (12%)  [Severe] >0.37 (3%) |
| ***Component items*** | | | | | |
| Chronic diseases | (17) Anemia; Arthritis; AF; CBVD; CKD; Diabetes; Heart failure; Heart valve disease; Hypertension; Hypotension; IHD; Osteoporosis; PD; PVD; RD; Thyroid disease; Urinary system disease | (8) Arthritis; Cataract; CHD; Glaucoma; Hypertension; Stroke; Thyroid disease; TIA | (13) Cancer; Celiac disease; Chronic bronchitis; Crohn’s disease; Diabetes; Heart disease; High cholesterol; Hypertension; Multiple sclerosis; Osteoarthritis; PD; RA; Stroke | (15) Blood disease; Cancer; Digestive disease; Ear disease; Endocrine disease; Eye disease; Genitourinary disease; Heart disease; Infectious disease; Mental disease; MSKD; Nervous disease; RD; Skin disease; Other disease | (20) Angina; Arrhythmia; Arthritis; Asthma; Cancer; Cataract; Depression; Diabetes; DED; DKD; Glaucoma; Heart attack; Heart disease – other; Heart murmur; Hypertension; High cholesterol; Lung disease; MD; Osteoporosis; Stroke |
| Sensory/physical impairments and geriatric syndromes | (12) Hearing impairment; Visual impairment; Dizziness; Dyspnea; Falls; Foot problems; Fragility fractures; Peptic ulcer; Skin ulcer; Sleep disturbance; UI; Weight loss and anorexia | (5) Hearing problem; Use a hearing aid; Use a walking aid; Tremor; UI | (1) Unintentional weight loss | (2) Use a walking aid; Balance problems | (8) Seeing difficulties; Hearing difficulties; Slow walking speed;^3,4^ Balance problems;^4^ Weak grip strength;^3,4^ Weak leg strength;^4^ UI; Significant weight loss^3,4^ |
| Cognitive impairment | (1) Memory and cognitive problems | (1) MMSE<15 |  |  | (1) Composite measure of cognitive problems across 4 tests of memory, mental speed and numeracy |
| Subjective symptoms and health status |  | (5) Lack of energy; Felt less useful; Don’t feel a lot of fun in life; Don’t feel very happy; Feel nothing to do | (6) Feels full of life; Has a lot of energy; Feels worn out; Feels tired; Self-rated health; Self-rated pain | (6) Feeling calm; Have a lot of energy; Feeling low; Social activity interfered by physical and emotional health; Self-rated health; Normal work interfered by pain | (4) Self-reported exhaustion;^3^ Self-rated health; Self-rated pain; Self-reported long-standing illness |
| Lifestyle risk factors |  |  |  | (2) Obesity (BMI>=30); Low physical activity | (2) Low physical activity;^3^ Obesity |
| Activity limitation | (3) Any activity limitation; Housebound; Mobility and transfer problems | (14) ADL & IADL limitations | (12) ADL limitations | (14) ADL & IADL limitations | (15) ADL & IADL limitations |
| Healthcare contact | (2) Polypharmacy (5+ medications); Requirement for care |  | (2) Polypharmacy (5+ medications); Frequency of healthcare visit in past year | (1) Polypharmacy (6+ medications) | (1) Polypharmacy (5+ medications) |
| Social | (1) Social vulnerability |  |  |  | (1) Living alone |
| **Abbreviation:** ADL: activities of daily living; AF: atrial fibrillation; BLSA: Beijing Longitudinal Study of Aging; CBVD: cerebrovascular disease; CHD: coronary heart disease; CKD: chronic kidney disease; DED: diabetic eye disease; DKD: diabetic kidney disease; eFI: electronic frailty index; FI: frailty index; GLOW: Global Longitudinal Study of Osteoporosis in Women; IADL: instrumental activities of daily living; IHD: ischemic heart disease; MD: macular degeneration; MMSE: mini-mental status examination; MSKD: musculoskeletal disease; PD: Parkinson’s disease; PVD: peripheral vascular disease; RA: rheumatoid arthritis; RD: respiratory disease; SD: standard deviation; TIA: transient ischemic attack; UI: urinary incontinence  ^1^ The frailty index was constructed using data from the randomized controlled trial ProAct65+ which compared group- and home-based falls prevention exercise to usual care in London, Nottingham and Derby [18].  ^2^ The original index contained 35 items including falls and fracture [31]; the latter were taken out from index and used as outcomes in subsequent study [32].  ^3^ Components of the frailty phenotypes proposed by Fried and colleagues [37].  ^4^ These variables had more than 5% missing values which were imputed by single imputation. | | | | | |

## B3.5 Covariates estimated at baseline

Where data was available from ELSA, key causal variables identified by the conceptual model were estimated and assigned to individuals at model entry. The following variables were estimated: (a) high physical activity; (b) cognitive impairment; (c) fear of falling; and (d) abnormal gait/balance. Cross-sectional analyses were conducted using combined information from both Wave 4 and 5 cohorts (n=13,422) rather than Wave 4 alone (n=7,255) to improve the statistical power. Estimations were conducted sequentially in the order of the variables (a)-(d) given above, with the preceding variable(s) serving as an explanatory variable (depending on model fit) for the next variable estimation. It should be noted that the estimations were conducted for associative patterns rather than causal inference.

For each dependent variable, the statistical model that produced the lowest Akaike and/or Bayesian information criterion (AIC and BIC) values was chosen as the best-fit model. A separate table showing all model-fit comparisons for this section is not reported here but is available upon request. The coefficient point estimates from the best-fit models were inputted to the simulation model to generate probability for the individual having the characteristic in question. Since all four variables were binary indicators, equation (1) was used to estimate the individual-specific probability:

$$\hat{P\left( Event | X \right)}=\frac{EXP(\hat{\beta_{0}}+\hat{\beta_{1}}X_{i1}+\ldots+\hat{\beta_{k}}X_{ik})}{1+EXP(\hat{\beta_{0}}+\hat{\beta_{1}}X_{i1}+\ldots+\hat{\beta_{k}}X_{ik})} (1)$$

$X$ describes the set of *k* explanatory variables for the event in question and $x_{ij}$ describes the value the explanatory variable *j* within the set takes for individual *i*. $\hat{\beta_{1}}$ to $\hat{\beta_{k}}$ are the estimated logistic regression coefficients for the explanatory variables, and $\hat{\beta_{0}}$ is the estimated constant term. Having generated the probability, a random number was generated for each individual to determine whether he/she has the characteristic in question. The variance-covariance matrix generated by the best-fit model estimation was stored and later used in probabilistic sensitivity analysis.

### High physical activity

The conceptual model identified physical activity level as a key causal variable. Therefore, the model incorporates a binary indicator for high physical activity. The UK CMO guideline recommended 150 minutes per week of at least moderate intensity exercise of any form for adults aged 65+ [38]. According to a UK survey in 2008, 20% of men and 17% of women aged 65-74 met this guideline level [39]. ELSA Waves 4-5 contained a variable summarising the physical activity levels of individuals (Not known; Sedentary; Low; Moderate; High), but the intensity or duration was not stated. Nevertheless, 21.5% of men and 15.9% of women aged 65-74 in ELSA reported ‘High’ level of physical activity. Given the comparable prevalence rates to the survey, the ELSA information of ‘High’ physical activity was assumed to indicate meeting of CMO guideline. The overall prevalence rate of high physical activity in the ELSA cohort aged 60+ was 17.3%.

A binary variable for high vs. non-high physical activity was created and used as the dependent variable in a multivariate logistic regression to estimate the probability of an individual engaging in high physical activity given his/her characteristics. Table B7 shows the regression coefficients for the best-fit model.

| **Table B7** Logistic regression coefficients for high physical activity from ELSA Waves 4 and 5. | | |
| --- | --- | --- |
| ***Dependent variable: High physical activity (N=13,422)*** | | |
| **Explanatory variables** | **Coefficient (SE)** | **P-value** |
| Constant | -4.187 (2.475) | 0.091 |
| Age | 0.151 (0.070) | 0.031 |
| Age^2 | -0.001 (0.0004) | 0.008 |
| Female | -0.255 (0.049) | <0.001 |
| SES (ref: Most privileged quartile) |  |  |
| *2^nd^ quartile* | -0.115 (0.065) | 0.077 |
| *3^rd^ quartile* | -0.202 (0.059) | 0.001 |
| *Most deprived quartile* | -0.366 (0.091) | <0.001 |
| Frailty (0-100) | -0.100 (0.004) | <0.001 |
| **Abbreviation:** ELSA: English Longitudinal Study of Ageing; Ref: reference; SE: standard error; SES: socioeconomic status | | |

The best-fit model excluded the categorical variable for falls history as an explanatory variable. The inclusion of quadratic age term showed a non-linear association with age. Women were less likely to engage in high physical activity, and there was a statistically significant social gradient in high physical activity probability. Frailer individuals were less likely to engage in high physical activity.

### Cognitive impairment

The conceptual model identified cognitive impairment as a key causal variable. The model hence incorporated a binary indicator for cognitive impairment. ELSA appeared to under-report the prevalence of severe cognitive impairment: the combined prevalence of self-reported dementia and Alzheimer’s disease diagnosis was less than 1% of the population aged 65+ compared to estimates of around 5% in other epidemiological studies [40]. ELSA also contained no validated measure of cognitive impairment such as the mini-mental state examination. However, ELSA reported other measures of cognitive function based on tests of memory, mental speed, and numeracy [7].

From these measures, a composite measure of cognitive impairment was constructed. Individuals in the bottom quartile for scores from all of the following cognition tests – date recall, word recall (with and without delay), and animal name recall (numeracy score was only available in Wave 4 and hence not used) – were classified as cognitively impaired. One UK survey reported a prevalence rate of mild cognitive impairment and dementia of 21.8% for men aged 65-84 [41]. In comparison, the composite measure estimated the prevalence of 23.0% for men aged 65-84 in ELSA Waves 4-5. Given the comparable prevalence, the ELSA variable was used as an indicator of cognitive impairment. Table B8 shows the regression coefficients for the best-fit model.

| **Table B8** Logistic regression coefficients for cognitive impairment from ELSA Waves 4 and 5. | | |
| --- | --- | --- |
| ***Dependent variable: Cognitive impairment (N=13,422)*** | | |
| **Explanatory variables** | **Coefficient (SE)** | **P-value** |
| Constant | 0.639 (2.475) | 0.723 |
| Age | -0.099 (0.049) | 0.042 |
| Age^2 | 0.001 (0.0003) | 0.003 |
| Female | -0.340 (0.045) | <0.001 |
| SES (ref: Most privileged quartile) |  |  |
| *2^nd^ quartile* | 0.190 (0.062) | 0.002 |
| *3^rd^ quartile* | 0.168 (0.055) | 0.002 |
| *Most deprived quartile* | 0.181 (0.074) | 0.015 |
| Frailty (0-100) | 0.025 (0.002) | <0.001 |
| High physical activity | -0.280 (0.070) | <0.001 |
| **Abbreviation:** ELSA: English Longitudinal Study of Ageing; Ref: reference; SE: standard error; SES: socioeconomic status | | |

There was a statistically significant non-linear relationship between cognitive impairment and age. Women were less likely to be cognitively impaired. There was evidence of a social gradient in impairment with all SES quartiles below the most privileged showing higher rates. Frailty was associated with higher prevalence, engaging in high physical activity with lower prevalence.

### Fear of falling

The conceptual model identified fear of falling as a key causal variable. ELSA Wave 4 contained a binary variable for whether the respondent experienced fear of falling while walking, with the overall prevalence of 6.8% for the ELSA cohort aged 60+. The variable was imputed for the Wave 5 cohort from the Wave 4 data. Table B9 shows the regression coefficients for the best-fit model.

| **Table B9** Logistic regression coefficients for fear of falling from ELSA Waves 4 and 5. | | |
| --- | --- | --- |
| ***Dependent variable: Fear of falling (N=13,422)*** | | |
| **Explanatory variables** | **Coefficient (SE)** | **P-value** |
| Constant | -8.095 (0.379) | <0.001 |
| Age | 0.018 (0.005) | <0.001 |
| Female | 0.246 (0.080) | 0.002 |
| Falls history (ref: No falls history) |  |  |
| *Single non-MA fall* | 0.879 (0.113) | <0.001 |
| *Recurrent non-MA falls* | 1.315 (0.100) | <0.001 |
| *Single MA fall* | 1.335 (0.139) | <0.001 |
| *Recurrent falls with MA* | 1.330 (0.138) | <0.001 |
| Frailty (0-100) | 0.235 (0.013) | <0.001 |
| Frailty^2 | -0.003 (0.0002) | <0.001 |
| **Abbreviation:** ELSA: English Longitudinal Study of Ageing; MA fall: fall requiring medical attention; Ref: reference; SE: standard error | | |

There was a statistically significant linear relationship between age and fear of falling. Women were likelier to experience fear. There was no significant evidence of social gradient in fear prevalence. Falls history of any type was associated with increased prevalence, consistent with fear of falling being a consequence of falls. There was a non-linear relationship between frailty and fear of falling.

### Abnormal gait and balance

The conceptual model identified gait and balance status as a key causal variable. ELSA contained no validated measure for gait and balance impairment, such as TUG or Tinetti balance scale. Instead, measure of walking speed (time in seconds to walk eight feet) and self-reported level of balance difficulty were used as indicators. Following a previous practice [29], those with walking speed in the bottom sex-specific quintile and those who could not perform the walking speed test due to health reasons were deemed to have abnormal gait. The prevalence of abnormal gait for the ELSA cohort was 26.4%. For the measure of balance, those who self-reported having balance difficulties ‘Very often’, ‘Always’ or ‘Can’t walk’ were deemed to have abnormal balance. The overall prevalence of abnormal balance was 7.3%. A composite measure was constructed that gave a value of 1 if individual had abnormal gait/balance and 0 otherwise; the overall prevalence was 28.0%. Table B10 shows the regression coefficients from the best-fit model.

| **Table B10** Logistic regression coefficients for abnormal gait and/or balance from ELSA Waves 4 and 5. | | |
| --- | --- | --- |
| ***Dependent variable: Abnormal gait and/or balance (N=13,422)*** | | |
| **Explanatory variables** | **Coefficient (SE)** | **P-value** |
| Constant | 7.135 (2.217) | 0.001 |
| Age | -0.335 (0.060) | <0.001 |
| Age^2 | 0.003 (0.0004) | <0.001 |
| Female | -0.349 (0.053) | <0.001 |
| SES (ref: Most privileged quartile) |  |  |
| *2^nd^ quartile* | 0.338 (0.074) | <0.001 |
| *3^rd^ quartile* | 0.386 (0.065) | <0.001 |
| *Most deprived quartile* | 0.520 (0.083) | <0.001 |
| Falls history (ref: No falls history) |  |  |
| *Single non-MA fall* | 0.112 (0.083) | 0.176 |
| *Recurrent non-MA falls* | 0.365 (0.090) | <0.001 |
| *Single MA fall* | 0.257 (0.121) | 0.033 |
| *Recurrent falls with MA* | 0.322 (0.148) | 0.030 |
| Frailty (0-100) | 0.170 (0.004) | <0.001 |
| High physical activity | -0.299 (0.088) | 0.001 |
| Cognitive impairment | 0.265 (0.063) | <0.001 |
| Fear of falling | 0.381 (0.103) | <0.001 |
| **Abbreviation:** ELSA: English Longitudinal Study of Ageing; MA fall: fall requiring medical attention; Ref: reference; SE: standard error; SES: socioeconomic status | | |

Both age and sex were significantly associated with abnormal gait/balance. There was also a significant SES gradient to the prevalence. Falls history of any type except single non-MA fall increased the likelihood of having abnormal gait/balance, showing evidence of a feedback loop. Higher frailty and cognitive impairment were both associated with higher prevalence of abnormal gait/balance, while engaging in high physical activity was associated with lower prevalence. Finally, fear of falling was associated with abnormal gait/balance.

According to a previous model that incorporated gait/balance screening, TUG had sensitivity and specificity of 31.0% and 74.0%, respectively, for cohort aged 65-89 [42]. In comparison, the model predicted that 39.6% of those aged 65-89 who received falls risk screening and experienced any fall in the first cycle had abnormal gait/balance, while 72.0% of those who did not experience any fall did not, giving sensitivity of 39.6% and specificity of 72.0%. Hence, the ELSA variable approximates the performance of TUG, a screening tool recommended by NICE CG161 (p. 53) [8].

# B4 Outcomes estimated at baseline

Given the baseline characteristics, the following outcomes were estimated from ELSA: EQ-5D health utilities (Section B4.1); productivity level – paid employment and unpaid work (B4.2); CASP-19 social wellbeing (B4.3); and comorbidity care costs by sector – healthcare, social care, out-of-pocket care, and informal care (B4.4).

## B4.1 Health utilities

The generic health utility measure for CUA should capture the primary and secondary health effects of falls and background transition in health status (see Figure A1 in Appendix A). The ideal method is to use individual-level utility data. ELSA does not collect such data; although its parent survey, the annual cross-sectional Health Survey for England (HSE), collects EQ-5D-3L data [43, 44]. Therefore, this study estimated EQ-5D-3L values from ELSA data by using published information on EQ-5D-3L item responses and average index values for older subgroups in HSE.

Table B11 lists: (i) the five dimensions and three levels of EQ-5D-3L; (ii) the prevalence rates of item responses for each level for the subgroup aged 75+ in HSE 2008 [43]; (iii) the ELSA variables used as proxy indicators of problems in the five dimensions and three levels; and (iv) the prevalence rates of item responses for the proxy variables in ELSA. ELSA was searched for variables that not only captured similar concept as the EQ-5D-3L responses but also had similar prevalence rates to the sub-cohort aged 75+ in HSE 2008. The final set of proxy variables produced similar prevalence rates for mobility, usual activities, and anxiety and depression, but overestimated the rate of ‘Some problems’ for self-care and underestimated the rate of ‘Some’ pain. Previous studies have used this approach of identifying proxy variables and items to estimate health utility values [45, 46].

| **Table B11** Proxy variable selection in ELSA to estimate EQ-5D-3L values. | | | | |
| --- | --- | --- | --- | --- |
| **EQ-5D dimension** | **Dimension levels** | **Prevalence in HSE 2008 aged 75+^1^ (%)** | **ELSA proxy variable** | **Prevalence in ELSA Wave 4 aged 75+ (%)** |
| Mobility | No problem | 47.4 | Not (1) or (2) | 45.5 |
|  | Some problems | 52.3 | (1) Some difficulty walking ¼ mile unaided | 54.4 |
|  | Confined to bed | 0.3 | (2) Confined to bed | 0.1 |
| Self-care | No problem | 85.1 | No ADL limitation | 60.8 |
|  | Some problems | 13.6 | 1-5 ADL limitations | 37.8 |
|  | Unable to | 1.4 | 6+ ADL limitations | 1.4 |
| Usual activities | No problem | 58.8 | No IADL limitation | 58.0 |
|  | Some problems | 35.4 | 1-5 IADL limitations | 38.1 |
|  | Unable to | 5.8 | 6+ IADL limitations | 3.9 |
| Pain; discomfort | No | 38.1 | No pain most of the time | 59.6 |
|  | Some | 52.7 | Mild/moderate pain most of the time | 31.8 |
|  | Extreme | 9.2 | Severe pain most of the time | 8.6 |
| Anxiety; depression | No | 76.7 | Not (1) or (2) | 82.4 |
|  | Some | 21.8 | (1) Feels what happens in life is often beyond control | 15.6 |
|  | Extreme | 1.5 | (2) Diagnosis of psychiatric problem of anxiety | 2.0 |
| **Abbreviation:** ADL: activities of daily living; ELSA: English Longitudinal Study of Ageing; HSE: Health Survey for England; IADL: instrumental activities of daily living  ^1^ Data reported in Janssen and Szende (2014) [43]. | | | | |

Having chosen the proxy variables, the time trade-off valuation set elicited from a representative UK adult population [47] was applied to derive the preference-based EQ-5D index scores. This was the same valuation set as that applied in the HSE studies [43, 44]. Table B12 shows the means and 95% confidence intervals for the index scores reported in HSE 2008 for age groups 65-74 and 75+ [43]. There were close overlaps between the HSE 2008 means and those estimated from ELSA Wave 4 (also conducted in year 2008) for both age groups. The ELSA estimates were further compared to those reported by Ara and Brazier who pooled four HSE cross-sections between 2003 and 2006 [44]. There were reasonable overlaps between the two sets of estimates, with the ELSA means falling within the 95% confidence intervals of HSE 2003-2006 for each five-year age group.

| **Table B12** Comparison between general population EQ-5D values estimated from ELSA Wave 4 and those reported in Health Survey for England. | | | | | | | |
| --- | --- | --- | --- | --- | --- | --- | --- |
|  |  | Age 65-74 | Age 75+ |  |  |  |  |
| HSE 2008^1^ | Mean | 0.784 | 0.717 |  |  |  |  |
|  | 95% CInt | 0.772-0.796 | 0.703-0.731 |  |  |  |  |
| ELSA estimate | Mean | 0.783 | 0.718 |  |  |  |  |
|  | 95% CInt | 0.776-0.790 | 0.710-0.726 |  |  |  |  |
|  |  | Age 60-64 | Age 65-69 | Age 70-74 | Age 75-79 | Age 80-84 | Age 85+ |
| HSE 2003-2006^2^ | Mean | 0.807 | 0.804 | 0.779 | 0.753 | 0.699 | 0.650 |
|  | 95% CInt | 0.793-0.821 | 0.790-0.817 | 0.766-0.791 | 0.739-0.767 | 0.677-0.719 | 0.624-0.675 |
| ELSA estimate | Mean | 0.797 | 0.790 | 0.777 | 0.744 | 0.705 | 0.670 |
|  | 95% CInt | 0.787-0.806 | 0.780-0.800 | 0.767-0.787 | 0.732-0.756 | 0.690-0.721 | 0.646-0.695 |
| **Abbreviation:** CInt: confidence interval; ELSA: English Longitudinal Study of Ageing; HSE: Health Survey for England  ^1^ Data reported in Janssens and Szende (2014) [43]; n=14,763; UK time trade-off value set [47].  ^2^ Data reported in Ara and Brazier (2011) [44]; n=41,174; UK time trade-off value set [47]. | | | | | | | |

Having estimated the individual-level EQ-5D scores, a linear regression estimated the associations between EQ-5D scores and individuals’ characteristics. Table B13 shows the results of the linear regression. To account for ceiling effects, individuals predicted an EQ-5D score greater than 1 (3.6% of the cohort) were reassigned the score of 1. Predicted scores were assigned to simulated individuals.

| **Table B13** Linear regression coefficients for EQ-5D from ELSA Waves 4 and 5. | | |
| --- | --- | --- |
| ***Dependent variable: EQ-5D (0-1) (N=13,422)*** | | |
| **Explanatory variables** | **Coefficient (SE)** | **P-value** |
| Constant | 0.883 (0.010) | <0.001 |
| Age^2 | 0.00004 (0.000002) | <0.001 |
| Female | -0.015 (0.004) | <0.001 |
| SES (ref: Most privileged quartile) |  |  |
| *2^nd^ quartile* | -0.009 (0.005) | 0.070 |
| *3^rd^ quartile* | -0.021 (0.004) | <0.001 |
| *Most deprived quartile* | -0.053 (0.006) | <0.001 |
| Falls history (ref: No falls history) |  |  |
| *Single non-MA fall* | -0.010 (0.006) | 0.097 |
| *Recurrent non-MA falls* | -0.060 (0.006) | <0.001 |
| *Single MA fall* | -0.015 (0.009) | 0.095 |
| *Recurrent falls with MA* | -0.005 (0.011) | 0.644 |
| Frailty (0-100) | -0.021 (0.001) | <0.001 |
| Frailty^2 | 0.0001 (0.00001) | <0.001 |
| Cognitive impairment | -0.012 (0.004) | 0.008 |
| Abnormal gait/balance | -0.040 (0.005) | <0.001 |
| **Abbreviation:** ELSA: English Longitudinal Study of Ageing; MA fall: fall requiring medical attention; Ref: reference; SE: standard error; SES: socioeconomic status | | |

Only the squared age term was significantly associated with EQ-5D, and the model fit improved when the ordinary age term was removed. Women had lower EQ-5D than men. Those in the two most deprived SES quartiles had lower EQ-5D than those in the most privileged quartile. Coefficients for falls history produced counterintuitive results, with recurrent non-MA falls history being the only type to be associated with a statistically significant decrement in EQ-5D. This can be explained by the closer relationship between MA falls history and frailty, such that estimation without frailty as explanatory variables produced more intuitive results: i.e., statistically significant EQ-5D decrements for both types of MA fall history which were of larger magnitude than that of recurrent non-MA falls. Higher frailty, cognitive impairment, and abnormal gait/balance were all significantly associated with lower EQ-5D.

Note that the coefficients on the falls history variable are unlikely to capture the primary/acute effect of falls on EQ-5D. This is because the EQ-5D data estimated from ELSA could have been recorded as much as 12 months after the fall incidence. Section B6.3 separately models the acute effect of falls on QALY using other published data. Moreover, Section B7.3 estimates the longitudinal progression of EQ-5D which capture the secondary effect of falls via dynamic propagators such as frailty.

## B4.2 Productivity level

The conceptual model identified productivity level – via formal employment and voluntary work – as a key non-health outcome. Using ELSA, this section estimates the probability of individuals engaging in paid and unpaid forms of productivity.

### Paid employment

ELSA Waves 4-5 contained information on whether the respondent was in paid employment in the previous week. Overall, 17.4% of the combined cohort aged 60+ reported being in paid employment. Table B14 shows the regression coefficients from the best-fit model for being in paid employment.

| **Table B14** Logistic regression coefficients for paid employment status from ELSA Waves 4 and 5. | | |
| --- | --- | --- |
| ***Dependent variable: Paid employment status (N=13,422)*** | | |
| **Explanatory variables** | **Coefficient (SE)** | **P-value** |
| Constant | 41.116 (2.975) | <0.001 |
| Age | -1.014 (0.085) | <0.001 |
| Age^2 | 0.006 (0.001) | <0.001 |
| Female | -0.698 (0.053) | <0.001 |
| SES (ref: Most privileged quartile) |  |  |
| *2^nd^ quartile* | 0.005 (0.072) | 0.948 |
| *3^rd^ quartile* | 0.089 (0.063) | 0.160 |
| *Most deprived quartile* | -0.258 (0.095) | 0.006 |
| Frailty (0-100) | -0.034 (0.011) | 0.002 |
| Frailty^2 | -0.001 (0.0004) | 0.003 |
| High physical activity | 0.170 (0.061) | 0.006 |
| Cognitive impairment | -0.136 (0.075) | 0.070 |
| **Abbreviation:** ELSA: English Longitudinal Study of Ageing; Ref: reference; SE: standard error; SES: socioeconomic status | | |

Older individuals and women were less likely to be in paid employment. Relative to those in the most privileged SES quartile, those in the most deprived quartile were less likely to be in paid employment, while those in the 2^nd^ and 3^rd^ quartiles did not have significantly different probabilities. Higher frailty was associated with reduced probability, while engaging in high physical activity was associated with higher probability. Cognitive impairment was associated with lower probability, but this was not statistically significant at the 95% confidence level.

The human capital approach was used to value the societal contribution of older persons in paid employment. Employed persons were assumed to generate an annual value of £24,192 which is the UK national average weekly pay in May 2020 before tax and other deductions [48] multiplied by 48 working weeks. This value was assumed to be constant over the model simulation time.

### Unpaid work – volunteering and unpaid help

ELSA Waves 4-5 contained information on the frequency of ‘formal’ volunteering activities (i.e., as part of a volunteering organisation) in the past 12 months: at least once a week; less than once a week; and one-off. Similar frequency data was reported for provision of unpaid help (i.e., volunteering on a less formal basis), including informal caregiving for sick persons, childcare, and helping people with daily activities such as cooking, cleaning, and transporting. Together, they constituted unpaid work performed by older persons. It was assumed that only those who volunteered or provided unpaid help at least once a week generated consistent volume of societal contribution over a year. Therefore, a new binary variable was created to indicate weekly unpaid work, with 28.0% of the older population reporting to have done so in the previous 12 months.

Table B15 shows the logistic regression coefficients from the best-fit model estimating the prevalence of weekly unpaid work. Unlike paid employment, older individuals and women were likelier to be engaged in unpaid work. Nevertheless, there was a similar SES gradient, with the 3^rd^ and the most deprived quartiles being significantly less likely to engage than the most privileged. Increasing squared frailty was associated with lower rate of work. Engaging in high physical activity was associated with higher rate, while cognitive impairment and abnormal gait/balance were associated with lower. Those in paid employment were less likely to engage.

| **Table B15** Logistic regression coefficients for weekly unpaid work from ELSA Waves 4 and 5. | | |
| --- | --- | --- |
| ***Dependent variable: Weekly unpaid work (N=13,422)*** | | |
| **Explanatory variables** | **Coefficient (SE)** | **P-value** |
| Constant | -16.403 (1.884) | <0.001 |
| Age | 0.432 (0.052) | <0.001 |
| Age^2 | -0.003 (0.0004) | <0.001 |
| Female | 0.382 (0.041) | <0.001 |
| SES (ref: Most privileged quartile) |  |  |
| *2^nd^ quartile* | -0.007 (0.054) | 0.894 |
| *3^rd^ quartile* | -0.100 (0.048) | 0.038 |
| *Most deprived quartile* | -0.366 (0.070) | <0.001 |
| Frailty^2 | -0.0006 (0.0001) | <0.001 |
| High physical activity | 0.127 (0.052) | 0.014 |
| Cognitive impairment | -0.479 (0.056) | <0.001 |
| Abnormal gait/balance | -0.314 (0.059) | <0.001 |
| Paid employment | -0.280 (0.058) | <0.001 |
| **Abbreviation:** ELSA: English Longitudinal Study of Ageing; Ref: reference; SE: standard error; SES: socioeconomic status | | |

The opportunity cost approach was used to value each hour of unpaid work. This approach values unpaid productivity in terms of the value of the next best use of the time. Where paid work is not the next best use, which is likely for the older population, literature recommends using wage at the previous paid job (possibly adjusted for lower productivity at unpaid work) or minimum wage to value the hourly contribution [49]. Therefore, each hour of unpaid work was valued at the UK national living wage since April 2021 of £8.91 [50]. Further assumptions were needed on the average annual number of unpaid work hours. A UK survey estimated that older people aged 65+ provided on average 159.1 hours of formal and informal volunteering per year [51]. Given the overall prevalence of weekly unpaid work of 27.8% among those aged 65+ in ELSA Wave 4, if each unpaid worker provided 11 hours per week, then this would amount to an average of 159 hours per person annually (11*52*0.278). Therefore, it was assumed that those engaged in weekly unpaid work provided 572 (11*52) hours of work annually which is worth £5,097 at the national living wage.

For those providing informal care as unpaid work, the impact of caregiving on their health should be captured (see Section A5.1 in Appendix A). To identify the health impact, the best-fit linear model on EQ-5D (Table B13) was re-run with weekly informal caregiving – isolated from the weekly unpaid work variable – as a covariate. There was no statistically significant evidence of negative or positive effect of informal caregiving on EQ-5D. To verify whether this finding applies to younger informal caregivers aged less than 60, separate logistic regressions were estimated for the younger cohort with self-reported poor health status and anxiety as dependent variables (EQ-5D was not estimated for the cohort aged <60) and age, sex, and informal caregiving as explanatory variables. There was similarly no statistically significant evidence of informal caregiving affecting the likelihood of poor health status or anxiety. Therefore, health effect on informal caregivers was not incorporated in the model.

## B4.3 Social wellbeing

Section A5.1 in Appendix A discussed the importance of capturing social wellbeing as a non-health outcome. ELSA contained the control, autonomy, self-realisation and pleasure, 19 items (CASP-19) scale measuring social wellbeing on a scale between 0 and 57 [52]. Missing CASP-19 data were imputed; the scores were rescaled to range 0-1. A linear regression estimated the associations between CASP-19 scores and the individual-level characteristics. Table B16 shows the results of the linear regression. Predicted rescaled CASP-19 scores were then assigned to individuals.

| **Table B16** Linear regression coefficients for CASP-19 scores from ELSA Waves 4 and 5. | | |
| --- | --- | --- |
| ***Dependent variable: CASP-19 (0-1) (N=13,422)*** | | |
| **Explanatory variables** | **Coefficient (SE)** | **P-value** |
| Constant | 0.477 (0.091) | <0.001 |
| Age | 0.009 (0.002) | <0.001 |
| Age^2 | -0.0001 (0.00002) | <0.001 |
| Female | 0.020 (0.002) | <0.001 |
| SES (ref: Most privileged quartile) |  |  |
| *2^nd^ quartile* | -0.020 (0.003) | <0.001 |
| *3^rd^ quartile* | -0.044 (0.003) | <0.001 |
| *Most deprived quartile* | -0.081 (0.004) | <0.001 |
| Falls history (ref: No falls history) |  |  |
| *Single non-MA fall* | -0.009 (0.004) | 0.011 |
| *Recurrent non-MA falls* | -0.021 (0.004) | <0.001 |
| *Single MA fall* | -0.006 (0.005) | 0.288 |
| *Recurrent falls with MA* | -0.016 (0.007) | 0.017 |
| Frailty (0-100) | -0.008 (0.0003) | <0.001 |
| Frailty^2 | 0.00002 (0.00001) | 0.001 |
| High physical activity | 0.018 (0.002) | <0.001 |
| Abnormal gait/balance | -0.007 (0.003) | 0.022 |
| Unpaid work | 0.017 (0.002) | <0.001 |
| **Abbreviation:** CASP-19: control, autonomy, self-realisation and pleasure, 19 items; ELSA: English Longitudinal Study of Ageing; MA fall: fall requiring medical attention; Ref: reference; SE: standard error; SES: socioeconomic status | | |

There was a non-linear relationship between age and CASP-19 and between frailty and CASP-19. There was a SES gradient to the score. Those engaging in unpaid work were likelier to report higher scores. The dynamic progression of CASP-19 is estimated in Section B7.3, which allows the accumulation of CASP-adjusted life years (CALYs) using the same method as estimating QALYs with EQ-5D. Note that CALY is a metric constructed *ad hoc* and does not incorporate preference-based health utility such as EQ-5D within QALY. Results concerning CALY should therefore be interpreted with caution.

Unlike EQ-5D, no acute effects of falls on CASP-19 were incorporated, and the potential impact of falls prevention intervention (e.g., exercise enhancing self-realisation) on CASP-19 were similarly excluded. The modelled intervention impact on CASP-19 and CALY is hence conservative.

## B4.4 Comorbidity care costs

The expert guideline on falls prevention economic evaluation recommends incorporation of all-cause care consequences in base case analysis, followed by fall-related care consequences alone in sensitivity analysis [53]. Using data from literature and ELSA, all-cause care costs were estimated by sector: (i) primary and secondary healthcare; (ii) community healthcare; (iii) short-term social care; (iv) OOP care cost; and (v) informal caregiving cost. The social care costs in (iii) exclude those associated with LTC which are parameterised separately in Section B7.2.

Importantly, to avoid double-counting the care costs, those directly related to falls – parameterised in Section B6.4 – were subtracted from all-cause care costs to obtain comorbidity care costs. However, as discussed in Section B6.4, reliable data on direct fall-related care costs could be obtained only for primary and secondary healthcare costs. Therefore, the distinction between all-cause and comorbidity care costs was made only for primary and secondary healthcare costs.

### Primary and secondary healthcare costs

ELSA contains no information on individuals’ healthcare utilisation and cost. Hence, all-cause healthcare costs were obtained from literature: Han and colleagues used primary care records of 95,863 individuals aged 65-95 in 125 UK general practices to estimate the annual primary and secondary healthcare cost (comprising GP consultations and emergency and elective hospital admissions) by eFI category (Fit, Mild, Moderate and Severe) [54]. The costs were estimated using unit costs at 2013/14 prices and adjusted for age, gender, ethnicity, social deprivation, non-frailty long-term conditions, and registration drop-out. Because the current model’s frailty index used the same cut-off percentiles as the eFI and had similar cut-off values (Table B6), it was assumed that the healthcare costs estimated by Han and colleagues can be applied to modelled individuals by their frailty category.

The second column of Table B17 shows the annual all-cause healthcare cost by frailty category at 2013/14 prices as reported by Han and colleagues. The average NHS cost inflation rate for the period 2013-2019 (1.98%) obtained from the 2019 PSSRU unit costs data [55] was then applied to the 2013/14 costs to obtain the 2021/22 costs. As noted, the all-cause care costs incorporate fall-related costs which are later estimated in Section B6.4. The healthcare costs of MA falls were weighted by the prevalence of such falls by frailty category in ELSA to obtain the weighted average cost per frailty category. These were subtracted from the all-cause care costs to obtain the comorbidity care costs.

| **Table B17** Annual all-cause and comorbidity primary and secondary healthcare costs by frailty category. | | | |
| --- | --- | --- | --- |
| **Frailty category** | **Annual all-cause primary and secondary healthcare cost** | | **Annual comorbidity primary and secondary healthcare cost** |
|  | **2013/14 £^1^** | **2021/22 £^2^** | **2021/22 £^3^** |
| Fit | 1628.35 | 1904.88 | 1866.07 |
| Mild | 2189.40 | 2561.21 | 2388.48 |
| Moderate | 2836.95 | 3318.73 | 2884.86 |
| Severe | 3736.55 | 4371.10 | 3823.14 |
| ^1^ Data source: Han et al (2019) [54]  ^2^ Data source for average NHS cost inflation between 2013 and 2019 of 1.98%: Curtis and Burns (2019) [55].  ^3^ See Section B6.4 for estimation of direct healthcare costs of falls. | | | |

### Community healthcare costs

ELSA Waves 4-5 contain self-reported information on current receipt of care for the following seven basic and instrumental activities of daily living: moving around the house; washing/dressing; preparing a meal or eating; shopping and doing work around house; using phone/managing money; taking medication; and other difficulties. There is also information on the care source: (a) health visitor/district nurse – i.e., community healthcare; (b) local authority/social services – social care; (c) private paid help – OOP care; and (d) informal care. The cost associated with (a) is estimated in this subsection, and those associated with (b)-(d) in the following subsections.

According to ELSA Waves 4-5, receipt of any community healthcare service differed significantly by frailty category: among Fit or Mild individuals, the prevalence of receipt was 0.02%, compared to 1.23% among Moderate and 5.49% among Severe. Given the very low prevalence among Fit and Mild groups, it was assumed that the receipt was restricted to those with Moderate and Severe frailty. Multivariate logistic regression also found a significant association between receipt and cognitive impairment after controlling for frailty, but none for other characteristics including age, sex, SES, and falls history. Hence, the receipt was assigned to: 0.62% of cognitively intact, Moderately frail individuals; 2.57% of cognitively impaired, Moderately frail; 2.52% of cognitively intact, Severely frail; and 9.05% of cognitively impaired, Severely frail.

Once assigned, the receipt was assumed to be maintained for the duration of the annual cycle. To estimate the cost of annual receipt, the 2019 PSSRU report estimated the average cost per hour of work performed by Band 5 district and community nurses [55]: £60 at 2018/19 price, equivalent to £64.10 at 2021/22 price after applying 2018/19 NHS pay inflation of 2.24%. Recent PSSRU reports did not state the estimated duration of each nurse visit, but the 2010 PSSRU report estimated this to be 20 minutes [56]. The 2019 PSSRU report estimated that the community nurse would work 225 days per year, and the NHS job description for a district nurse stated that the nurses would make daily visits on their assigned patients [57]. This equated to 225 visits by the nurse for each patient every year, or 75 hours of work if each visit lasted 20 minutes. Applying the hourly cost, this equated to £4,809.22 per patient. It was assumed that the number of visits and the length of each visit did not vary by frailty and cognitive status and hence that the same annual cost can be applied to all recipients. This was based on the observation that the intensity of care need – measured by the number of activities of daily living (ADLs) requiring assistance – varied only modestly between frailty and cognitive status subgroups.

### Short-term social care costs

According to ELSA Waves 4-5, 2.5% of those aged 65+ received any social care from local authority or social services. This is close to the proportion reported for Sheffield in the 2018/19 Adult Social Care Activity and Finance Report which documented 2,500 new clients receiving short-term care from SCC in 2018, equivalent to 2.7% of the total Sheffield population aged 65+ in the same year (93,374) [27]. Disaggregating the prevalence rate by frailty category, 0.1% of those in Fit or Mild categories, 7.54% in Moderate and 24.26% in Severe received any social care. It was hence assumed that only the Moderately and Severely frail individuals received social care at their respective ELSA prevalence rates. The prevalence also varied significantly by cognitive status: 5.37% in Moderate frailty and cognitively intact; 12.33% in Moderate frailty and cognitively impaired; 18.91% in Severe frailty and cognitively intact; and 30.65% in Severe frailty and cognitively impaired.

According to the Age UK online care home cost calculator, the minimum hourly cost paid by SCC to home care providers was £20.75 in 2020 [58]. PSSRU 2019 reported that the average local authority-commissioned home care per patient per week was 12.8 hours. However, this figure neglects the variation in care hours/intensity by patients’ health status. As a measure of care intensity, ELSA Wave 4 contained a variable on the frequency of social care visit: every day or nearly every day; two/three times per week; and once per week or less (no corresponding variable was available for community healthcare). PSSRU 2019 described home care being received on weekdays as well as weekends. Therefore, those reporting receipt of daily or near-daily care in ELSA were assumed to receive seven visits per week; those reporting two/three visits, two visits; and those reporting one visit or less, one visit. If each visit was assumed to last 2.5 hours, this produced a weighted average of 13.0 hours per patient per week which is close to the 12.8 reported by PSSRU 2019. Hence, those receiving daily visits received 17.5 care hours per week costing £363.13 per week (at Age UK rate of £20.75), £103.75 for twice/week patients and £51.88 for once/week patients.

Given its short-term nature, social care would not be provided all-year around. According to the 2018/19 Adult Social Care Activity and Finance Report, SCC spent £5,539,000 in that year for 2,500 short-term social care clients aged 65+ [27]. This amounts to an average of £2,215.60 per client. Under the above assumptions of 2.5 hours per visit and £20.75 per care hour, the weighted average of care cost is £269.75 per week. If all clients receive the same number of weeks of care, this corresponds to 8.21 weeks of care per client. Hence, the total annual cost of social care is £2,981.26 for daily visit clients, £851.79 for twice/week clients, and £425.89 for once/week clients. The prevalence of different care intensity/visit frequency in ELSA varied by frailty category and cognitive status. From the per-recipient annual cost and the subgroup-specific prevalence of care intensity, the average cost per recipient was estimated for each frailty and cognitive status subgroup. These estimates are summarised in Table B18.

| **Table B18** Annual all-cause short-term social care costs by frailty and cognitive status. | | | | | |
| --- | --- | --- | --- | --- | --- |
| **Frailty & cognitive status** | **Prevalence of care receipt** | **Care intensity given receipt** | **Prevalence of care intensity** | **Annual cost per recipient^1^** | **Average cost per recipient** |
| Moderate – Cognitively intact | 5.37% | 7 visits/week | 65.1% | £2,981.26 | £2,149.06 |
|  |  | 2 visits/week | 14.0% | £851.79 |  |
|  |  | 1 visit/week | 20.9% | £425.89 |  |
| Moderate – Cognitively impaired | 12.33% | 7 visits/week | 65.9% | £2,981.26 | £2,192.93 |
|  |  | 2 visits/week | 19.5% | £851.79 |  |
|  |  | 1 visit/week | 14.6% | £425.89 |  |
| Severe – Cognitively intact | 18.91% | 7 visits/week | 65.0% | £2,981.26 | £2,155.02 |
|  |  | 2 visits/week | 16.0% | £851.79 |  |
|  |  | 1 visit/week | 19.0% | £425.89 |  |
| Severe – Cognitively impaired | 30.65% | 7 visits/week | 83.9% | £2,981.26 | £2,611.16 |
|  |  | 2 visits/week | 9.7% | £851.79 |  |
|  |  | 1 visit/week | 6.4% | £425.89 |  |
| ^1^ Assumed 2.5 hours per visit, 8.21 weeks of visits per year and cost of £20.75 per hour. | | | | | |

### Out-of-pocket care costs

ELSA Waves 4-5 contained information on the receipt of any privately paid help for ADL. The prevalence rates by frailty category were: 0.2% for Fit; 4.1% for Mild; 12.4% for Moderate; 19.9% for Severe; and 3.4% overall. Multivariate logistic regression showed that several covariates exerted statistically significant effects on OOP care receipt. Therefore, a multivariate risk equation for any OOP care receipt was estimated from ELSA. Table B19 shows the results from the best-fit model. Older and female individuals were likelier to require OOP care, as were frailer individuals. The SES gradient suggested that OOP care access depended on individuals’ ability to pay. High physical activity reduced the likelihood of requiring OOP care. Interestingly, those with cognitive impairment were less likely to access OOP care. This may be explained by cognitively impaired individuals receiving a greater amount of publicly financed community healthcare and social care (as well as informal care; see Table B21). That said, variables for receipts of community healthcare, social care, and informal care did not exert a statistically significant effect on OOP care receipt.

| **Table B19** Logistic regression coefficients for out-of-pocket care receipt from ELSA Waves 4 and 5. | | |
| --- | --- | --- |
| ***Dependent variable: OOP care receipt (N=13,422)*** | | |
| **Explanatory variables** | **Coefficient (SE)** | **P-value** |
| Constant | -11.184 (0.547) | <0.001 |
| Age | 0.050 (0.007) | <0.001 |
| Female | 0.703 (0.113) | <0.001 |
| SES (ref: Most privileged quartile) |  |  |
| *2^nd^ quartile* | -0.175 (0.132) | 0.185 |
| *3^rd^ quartile* | -0.499 (0.122) | <0.001 |
| *Most deprived quartile* | -1.137 (0.196) | <0.001 |
| Frailty (0-100) | 0.258 (0.019) | <0.001 |
| Frailty^2 | -0.003 (0.0003) | <0.001 |
| High physical activity | -0.640 (0.305) | 0.036 |
| Cognitive impairment | -0.523 (0.120) | <0.001 |
| **Abbreviation:** ELSA: English Longitudinal Study of Ageing; OOP: out-of-pocket; Ref: reference; SE: standard error; SES: socioeconomic status | | |

According to the Age UK online calculator, the hourly cost of privately paid home care in the Sheffield region was at least £21 in 2020 [58]. ELSA Wave 4 contained information on the frequency of OOP care visit as a measure of care intensity: every day or nearly every day; two/three times per week; and once per week or less. Therefore, similar assumptions were made as for social care to estimate the annual cost of OOP care: 2.5 hours per visit; seven visits per week for daily-visit group; two visits per week for two/three times per week; and one visit per week for once or less per week. However, unlike social care, it was assumed that OOP care was received all year around, and that the intensity of care (i.e., visits per week) varied by frailty category *and* SES quartile to account for the social gradient in access. Table B20 shows the estimated annual cost per OOP care recipient by SES quartile and frailty.

| **Table B20** Annual all-cause out-of-pocket care costs by SES quartile and frailty category. | | | | |
| --- | --- | --- | --- | --- |
| **Frailty/*SES quartile*** | ***Most privileged*** | ***2^nd^ quartile*** | ***3^rd^ quartile*** | ***Most deprived*** |
| Fit | £2,730.00 | £2,730.00 | £2,730.00 | £0 |
| Mild | £3,726.45 | £3,336.06 | £2,912.91 | £2,730.00 |
| Moderate | £7,067.97 | £5,460.00 | £5,571.93 | £6,046.95 |
| Severe | £10,537.8 | £7,275.45 | £6,491.94 | £2,730.00 |
| **Abbreviation:** SES: socioeconomic status  **Note:** The distribution of visit/week varied by frailty category and social quartile according to English Longitudinal Study of Ageing Wave 4 data. Assumed 2.5 hours per visit, 52 weeks of visits per year and cost of £21 per hour. | | | | |

### Informal caregiving costs

ELSA Waves 4-5 contained information on the receipt of informal help for ADL. The prevalence by frailty category were: 7.2% for Fit; 30.3% for Mild; 66.4% for Moderate; 86.0% for Severe; and 25.3% overall. Multivariate logistic regression showed that several covariates exerted statistically significant effects on receipt. Therefore, a multivariate risk equation for any informal care receipt was estimated from ELSA. Table B21 shows the results from the best-fit model. Older and frailer individuals, as well as women and the cognitively impaired, were likelier to receive informal care, as were those with fear of falling and abnormal gait/balance. Those with high physical activity were less likely to require informal care. There was significant evidence that those receiving community healthcare (but not other care types) were likelier to receive informal care.

| **Table B21** Logistic regression coefficients for informal care receipt from ELSA Waves 4 and 5. | | |
| --- | --- | --- |
| ***Dependent variable: Informal care receipt (N=13,422)*** | | |
| **Explanatory variables** | **Coefficient (SE)** | **P-value** |
| Constant | -3.299 (0.243) | <0.001 |
| Age | -0.015 (0.003) | <0.001 |
| Female | 0.388 (0.050) | <0.001 |
| Frailty (0-100) | 0.205 (0.009) | <0.001 |
| Frailty^2 | -0.002 (0.0002) | <0.001 |
| High physical activity | -0.432 (0.087) | <0.001 |
| Cognitive impairment | 0.401 (0.058) | <0.001 |
| Fear of falling | 0.405 (0.086) | <0.001 |
| Abnormal gait/balance | 0.330 (0.060) | <0.001 |
| Community healthcare | 1.572 (0.642) | 0.014 |
| **Abbreviation:** ELSA: English Longitudinal Study of Ageing; Ref: reference; SE: standard error | | |

Unlike social care and OOP care receipts, there was no variable in ELSA for weekly frequency of informal care receipt which could be used as a measure of care intensity. Hence, requiring informal care for two or more ADLs was used as an alternative measure of intensity. Among informal care recipients, 53.4% required care for a single need; the rest required it for multiple needs. Multivariate logistic regression showed that several covariates exerted statistically significant effects on the probability of having multiple vs. single needs given informal care receipt. Therefore, a multivariate risk equation for having multiple care needs given receipt was estimated from ELSA. Table B22 shows the results from the best-fit model. Older individuals and women were less likely to have multiple needs, and there was a significant SES gradient. Those engaging in high physical activity were less likely to have multiple needs, as well as those accessing OOP care. Frailer individuals and those with cognitive impairment and abnormal gait/balance were likelier to have multiple needs.

| **Table B22** Logistic regression coefficients for multiple informal care needs given informal care receipt from ELSA Waves 4 and 5. | | |
| --- | --- | --- |
| ***Dependent variable: Multiple informal care needs given informal care receipt (N=3,401)*** | | |
| **Explanatory variables** | **Coefficient (SE)** | **P-value** |
| Constant | -1.428 (0.432) | 0.001 |
| Age | -0.021 (0.005) | <0.001 |
| Female | -0.422 (0.085) | <0.001 |
| SES deprivation (ref: Most privileged quartile) |  |  |
| *2^nd^ quartile* | -0.319 (0.120) | 0.008 |
| *3^rd^ quartile* | -0.357 (0.103) | 0.001 |
| *Most deprived quartile* | -0.494 (0.128) | <0.001 |
| Frailty (0-100) | 0.201 (0.017) | <0.001 |
| Frailty^2 | -0.002 (0.0003) | <0.001 |
| High physical activity | -0.436 (0.208) | 0.036 |
| Cognitive impairment | 0.476 (0.092) | <0.001 |
| Abnormal gait/balance | 0.405 (0.100) | <0.001 |
| OOP care receipt | -0.578 (0.150) | <0.001 |
| **Abbreviation:** ELSA: English Longitudinal Study of Ageing; OOP: out-of-pocket; Ref: reference; SE: standard error; SES: socioeconomic status | | |

Having divided the informal care recipients into single vs. multiple need subgroups, further assumptions were required on the number of hours of informal care received per week and year to estimate the annual cost of informal care. First, it was assumed that the association between the number of care needs and the number of weekly visits for *social* care can be generalised to that for informal care. Social care was chosen as reference because its access does not depend on ability to pay (unlike OOP care). In Table B23, the first three columns show the distribution of *social* care receipt frequency by the number of social care needs (single vs. multiple). Assuming that these apply to informal care and that each visit lasted 2.5 hours, the average numbers of care hours per week for those with single (10.1 hours) or multiple (15.2 hours) care needs were estimated. Proxy goods method was used to value the informal care hours [49]: it was assumed that in the absence of informal care, individuals would purchase OOP care as a direct substitute at £21 per hour. Assuming that informal care would be received all-year around, the annual cost of informal care per needs category was estimated.

| **Table B23** Annual all-cause informal care cost by single vs. multiple care needs. | | | | |
| --- | --- | --- | --- | --- |
| **Number of care needs** | **Care receipt frequency^1^** | **Prevalence^1^** | **Average care hours per week^2^** | **Estimated annual informal care cost^3^** |
| Single | 7 days/week | 47.6% | 10.1 | £11,089.26 |
|  | 2 days/week | 20.6% |  |  |
|  | 1 day/week | 31.8% |  |  |
| Multiple | 7 days/week | 83.5% | 15.2 | £16,601.13 |
|  | 2 days/week | 7.1% |  |  |
|  | 1 day/week | 9.4% |  |  |
| ^1^ Data taken from weekly social care receipt frequency. It is assumed that the frequency distribution by number of care needs for social care is generalisable to informal care.  ^2^ Assumed 2.5 hours per visit.  ^3^ Estimated using proxy goods method: cost of £21 per care hour; 52 weeks of care per year. | | | | |

Finally, whether any receipt of informal care confers a measurable health benefit to the patient was explored. Tong, for example, incorporated an EQ-5D increment of 0.051 to dementia patients living with their caregivers, after adjusting for their MMSE score, behavioural score and residence [59]. Using a similar multiple linear regression technique as Tong, the best-fit linear model for EQ-5D (Table B13) was re-estimated for a subset of ELSA individuals receiving any form of care with informal care receipt as an additional covariate. There was no statistically significant evidence of an EQ-5D increment associated with informal care receipt relative to other forms of care. Hence, potential health benefit of informal care was not incorporated in the model. This, however, neglects potential non-health process benefits associated with informal care receipt that are not captured by EQ-5D.

# B5 Falls prevention strategy

As part of the conceptual model, Section A7 in Appendix A had discussed the current intervention practice in Sheffield and the recommended practices by the UK guidelines. The model represents these practices as usual care (UC) and recommended care (RC) scenarios, respectively. Section B5.1 provides an overview of interventions by scenario. Subsequent sections parameterise the following intervention features: access conditions (Section B5.2); resource use and cost (B5.3); and efficacy (B5.4).

## B5.1 Intervention overview

The model incorporates three intervention pathways operating in tandem (i.e., non-mutually exclusive from the decision-maker’s perspective): reactive, proactive, and self-referred. These pathways are mutually exclusive for individuals in any given annual cycle, though the same individual could enter multiple pathways over the simulation as his/her characteristics and falls risk change. Table B24 describes the current and recommended practices alongside their respective model scenarios. The final column lists the falls prevention RCTs from which relevant intervention evidence (e.g., efficacy, uptake rate) are obtained for each intervention.

| **Table B24** Falls prevention intervention features by intervention pathway and configuration. | | | | | |
| --- | --- | --- | --- | --- | --- |
| **Pathway** | **Configuration** | **Target population** | **Intervention type** | **Access condition** | **RCT evidence** |
| Reactive | Current practice | MA fall patients | - Mixed^1^ |  |  |
|  |  | Cognitively impaired | - Mixed^1^ |  |  |
|  |  | With intervention history | - Mixed^1^ |  |  |
|  | *Model usual care (UC) scenario* | MA fall patients | - Home assessment and modification (HAM) | Limited access^2^ | [60] |
|  |  | Cognitively impaired | - HAM | Limited access^2^ | [60] |
|  |  | With intervention history | - HAM | Limited access^2^ | [60] |
|  | Recommended practice | MA fall patients | - Multifactorial intervention |  |  |
|  |  | Cognitively impaired | - No specific recommendation |  |  |
|  |  | With intervention history | - No specific recommendation |  |  |
|  | *Model recommended care (RC) scenario* | MA fall patients | - Multifactorial intervention | Access pending demand^2^ | [13] |
|  |  | Cognitively impaired | - Multifactorial intervention | Access pending demand^2^ | [15] |
|  |  | With intervention history | - Multifactorial intervention | Access pending demand^2^ | [13]; [15] |
| Proactive | Current practice | General older population in community | - Falls risk screening in community; referral to multifactorial intervention by ICT team and Falls Clinic for high falls risk |  |  |
|  |  | Cognitively impaired | - No intervention: referral to Memory Clinic |  |  |
|  |  | With intervention history | - No repeated intervention: monitoring in primary care |  |  |
|  | *Model UC scenario* | Persons not receiving reactive intervention | - Falls risk screening at GP routine contact; referral to multifactorial intervention if high falls risk | Limited access^3^ | [17] |
|  |  | Cognitively impaired | - No intervention |  |  |
|  |  | With intervention history | - No intervention |  |  |
|  | Recommended practice | General older population in community | - Falls risk screening in community; referral to multifactorial intervention if high falls risk |  |  |
|  |  | Cognitively impaired | - No specific recommendation |  |  |
|  |  | With intervention history | - No specific recommendation |  |  |
|  | *Model RC scenario* | Persons not receiving reactive intervention | - Falls risk screening at GP routine contact; referral to multifactorial intervention if high falls risk | Access pending demand^3^ | [17] |
|  |  | Cognitively impaired | - CI-specific group- and home-based Tai Chi together with informal caregiver if high falls risk | Access pending demand^3^ | [23] |
|  |  | With intervention history | - Multifactorial intervention with reduced components, up to 3 re-receipts of falls clinic for cognitively intact persons^4^ - CI-specific Tai Chi | Access pending demand^3^ | [17]; [23] |
| Self-referred | Current practice | General older population in community | - Dance to Health strength and balance group exercise – individually tailored, supervised, progressive difficulty, 2 hours/week, ongoing duration |  |  |
|  |  | Cognitively impaired | - Dance to Health group exercise |  |  |
|  |  | With intervention history | - Encourage sustained participation |  |  |
|  | *Model UC scenario* | Persons not receiving reactive or proactive intervention | - Strength and balance group exercise^5^ - Increased likelihood of achieving physical activity target for group exercise participants | Limited access^6^ | [18]; [19] |
|  |  | Cognitively impaired | - Balance group exercise (efficacy from Tai Chi) | Limited access^6^ | [23] |
|  |  | With intervention history | - Sustained participation pending access | Limited access^6^ | [18]; [19]; [23] |
|  | Recommended practice | General older population in community | - Strength and balance exercise – individually tailored, progressive difficulty, 50+ hours (2+ hours/week) - Achieve recommended 150 (75) minutes/week of moderate (vigorous) intensity physical activity |  |  |
|  |  | Cognitively impaired | - Exercise should have supervision and appropriate intensity |  |  |
|  |  | With intervention history | - Encourage sustained participation |  |  |
|  | *Model RC scenario* | Persons not receiving reactive or proactive intervention | - Strength and balance group exercise^5^ - Increased likelihood of achieving physical activity target for group exercise participants | Access pending demand^6^ | [18]; [19] |
|  |  | Cognitively impaired | - Balance group exercise (efficacy from Tai Chi) | Access pending demand^6^ | [23] |
|  |  | With intervention history | - Sustained participation pending demand | Access pending demand^6^ | [18]; [19]; [23] |
| **Abbreviation:** CCG: Clinical Commissioning Group; FLS: Fracture Liaison Service; HAM: home assessment and modification; ICT: Integrated Community Therapy; MA fall: fall requiring medical attention; PT: physiotherapy; RC: recommended care; SCC: Sheffield City Council; STH: Sheffield Teaching Hospitals; UC: usual care.  ^1^ HAM for hospitalised fallers by SCC; multifactorial intervention by ICT team and Sheffield Falls Clinic; Tai Chi and PT by CCG FLS and STH; fall alarm service.  ^2^ See Table B25 for access conditions and rates.  ^3^ See Table B26 for access conditions and rates.  ^4^ The cap on the number of receipts (four in total; three re-receipts) was necessary to keep the annual flow of clients constant at around 21,000; see Section B5.3 for discussion. The cap does not affect cognitively impaired persons who are not referred to the falls clinic.  ^5^ Individually tailored, supervised, progressive difficulty, 50+ hours (2+ hours/week)  ^6^ See Table B28 for access conditions and rates. | | | | | |

### Reactive pathway

The current reactive pathway in Sheffield makes use of several interventions commissioned and delivered by different system actors, including home assessment and modification (HAM) provided by SCC and multifactorial intervention by diverse multidisciplinary teams (see Table A6 in Appendix A). But the client flows for multifactorial intervention appear to be low. Hence, the UC scenario makes a simplifying assumption that the reactive pathway relies on HAM provided by SCC. Eligibility does not depend on cognitive status or intervention history. For the recommended practice, NICE CG161 recommends that all MA fallers receive a multifactorial intervention without specific recommendation for cognitively impaired and intervention history subgroups [8]. Therefore, the RC scenario provides multifactorial intervention to all MA fallers regardless of cognitive status and intervention history.

The level of access to reactive HAM under current practice is limited, with hospitalised fallers being prioritised by SCC. This limited access pattern is parameterised for UC using ELSA data in Section B5.2. For RC, the model characterises full supply-side coverage of CG161-recommended level of intervention provision; but the access rate would still depend on uptake (i.e., ‘pending demand’ in Table B24) taken from the relevant RCTs; these are parameterised in Section B5.2.

The intervention study evidence for the reactive pathway was drawn from one Australia-based and two UK-based RCTs: (i) a reactive HAM evaluated against usual care in Australia for hip fracture patients aged 55+ with diverse cognitive status [60]; (ii) a reactive multifactorial intervention against usual care for cognitively intact persons aged 65+ admitted to A&E for a fall [13]; and (iii) a reactive multifactorial intervention against usual care for cognitively impaired persons aged 65+ admitted to A&E for a fall [15]. The Australian RCT was used in (i) because no UK-based RCT was identified.

### Proactive pathway

For the proactive pathway, the current practice in Sheffield relies on falls risk screening at routine contact with care professionals in community followed by referral of high-risk individuals to multifactorial intervention delivered by multidisciplinary teams. The current access rate for the proactive intervention is low, with the Falls Clinic managing around 300 clients per year (including reactive pathway clients). Accordingly, UC incorporates limited access to falls risk screening at GP contact and limited referral of high-risk individuals to multifactorial intervention; see Section B5.2 for parameterisation using ELSA. By contrast, RC incorporates 100% falls risk screening at GP contact and 100% referral of high-risk individuals, resulting in access to all eligible persons pending demand.

Under current practice, cognitively impaired persons are not generally referred to the proactive intervention (see Section A7.3 in Appendix A). Therefore, UC incorporates no intervention for the cognitively impaired subgroup. NICE CG161 makes no specific recommendation for the cognitively impaired. But it would be reasonable to assume that cognitively impaired persons are offered a proactive multifactorial intervention under RC, just as they are offered a reactive one. Unlike the reactive pathway, no RCT (UK or non-UK) was identified that evaluated a proactive multifactorial intervention targeting the cognitively impaired. Therefore, on discussion with the falls modelling expert, it was decided that high-risk cognitively impaired persons are referred to a tailored Tai Chi evaluated in a UK setting [23].

Also under the current practice, those who have already received the proactive multifactorial intervention are not generally referred again to the multidisciplinary team but monitored in primary care (Section A7.3). Hence, UC incorporates no intervention for those with proactive intervention history. NICE CG161 makes no specific recommendation for this subgroup. However, as discussed in Section A7.3, the falls specialist geriatrician confirmed that those who remain at high falls risk would ideally be referred again for further assessments. Hence, RC refers high-risk individuals with intervention history to appropriate proactive interventions. But the number of re-receipts of proactive multifactorial intervention for cognitively intact was limited to three to maintain the annual flow of clients to a level managed by seven falls clinics; see Section B5.3 for further discussion.

The intervention study evidence for the proactive pathway was drawn from two UK-based RCTs: (i) a proactive multifactorial intervention evaluated against usual care for cognitively intact persons aged 65+ (recruited at GP practices) with recurrent falls in past year and no A&E presentation for the latest fall [17]; and (ii) the aforementioned 20-week group- and home-based Tai Chi intervention for dementia patients accompanied by their caregivers [23].

### Self-referred pathway

For the self-referred pathway, the sole intervention under current practice in Sheffield is the Dance to Health exercise intervention which meets the recommendations on the requisite features of falls prevention exercise: i.e., individually tailored, supervised, progressive difficulty, and 50+ hours (at least 2 hours/week) [61]. But the current access rate is minimal, with around 20 regular self-financing participants. Therefore, UC incorporates highly limited access to self-referred group exercise. RC incorporates full coverage of self-referred group exercise intervention pending demand. Section B5.2 describes the parameterisation using ELSA.

Under current practice, cognitively impaired persons are still eligible to participate in group exercise [62]. Hence, UC does not exclude this subgroup from intervention receipt. Moreover, those with intervention history are actively encouraged to sustain participation in the exercise programme. Therefore, UC also does not exclude this subgroup from intervention receipt. The UK guidelines similarly recommend supervised, tailored exercise interventions for cognitively impaired persons and sustained participation for all with intervention history [38, 61]. Therefore, RC incorporates full coverage for cognitively impaired persons and those with intervention history pending demand.

The intervention study evidence for the proactive pathway was drawn from three UK-based RCTs: (i) a 24-week group- and home-based exercise intervention evaluated against usual care for cognitively intact persons aged 65+ (recruited via postal invites) who have experienced less than three falls in the past year [18]; (ii) a 36-week group- and home-based exercise for cognitively intact women aged 65+ with three or more falls in past year [19]; and (iii) the aforementioned 20-week group- and home-based Tai Chi intervention for cognitively impaired [23].

## B5.2 Intervention access conditions

This section parameterises the intervention access rates for UC and RC scenarios. Initial and long-term access rates are determined by eligibility and implementation levels (i.e., supply and demand levels).

### Reactive pathway

In practice, reactive interventions would be accessed by fallers immediately after being discharged from the fall-related medical attention. This process is simulated in the model by making reactive intervention accessible to eligible individuals at the very start of the given cycle (i.e., before routine GP contact). Those who access the intervention would have a reduced risk of falling in the given cycle. A limitation of this approach is that the MA fall in the previous cycle (for which the intervention is given) has already influenced the dynamic trajectories between the previous and given cycles (see Section B7.1) and the intervention is unable to modify this influence; this underestimates the reactive intervention benefits.

Table B25 summarises the eligibility and access rates for the reactive pathway under UC and RC. Under UC, only the hospitalised MA fall patients are eligible for HAM. This means that only a proportion of MA fallers receive HAM, though access would not depend on cognitive status and intervention history.

| **Table B25** Eligibility and access rates for the reactive falls prevention pathway under usual care and recommended care scenarios. | | |
| --- | --- | --- |
|  | **Usual care** | **Recommended care** |
| **Pathway component: Reactive HAM or multifactorial intervention** | | |
| Eligibility | HAM for severe MA fall patients regardless of cognitive status or intervention history | Multifactorial intervention for all MA fall patients regardless of fall severity, cognitive status, and intervention history |
| Access rate | Limited access rate by frailty category according to ELSA: 28.7% overall; 9.6% if Fit; 23.5% if Mild; 51.7% if Moderate; and 72.7% if Severe | All eligible persons pending demand: 53.8% uptake rate for cognitively intact [13]; 49.6% for cognitively impaired [15] |
| **Abbreviation:** ELSA: English Longitudinal Study of Ageing; HAM: home assessment and modification; MA fall: fall requiring medical attention | | |

To estimate the limited access under UC, ELSA data was used. ELSA Wave 4 contained variables for whether the individual received a balance and gait test from a doctor/nurse, whether further fall risk tests were recommended, and whether falls risk factors were discussed in the past year. It also contained variables for whether the individual received HAM and physiotherapy. From these, it was possible to construct a variable indicating receipt of falls risk screening/assessment by a medical professional in the past year plus HAM and/or physiotherapy in the same period. Assuming that the latter treatments are related to the falls risk assessment, the variable served as a proxy measure of annual access to falls risk assessment followed by treatment(s). Among those who had experienced at least one MA fall in the past year, 28.7% had received such assessment and treatment.^[[1]](#footnote-1)^ Under a further assumption that the assessment and treatment took place in response to the MA fall, this percentage measured the annual access to reactive falls prevention. According to the national HES data incorporated in the PHE model, 28% of MA falls resulted in hospital inpatient stay [28]. This is close to the 28.7% estimate. It is plausible that the ELSA variable approximates the access to standard discharge package for hospitalised fallers corresponding to the current practice in Sheffield. According to this variable, 1.9% of the population aged 60+ accessed the reactive intervention in one year, corresponding to around 2,300 individuals if applied to the Sheffield population.

A multivariate logistic regression was estimated to assess whether this access rate was significantly associated with individual-level characteristics. The result showed no significant association except with frailty. The lack of association with cognitive impairment was consistent with the reactive intervention being accessed regardless of cognitive status. Therefore, the model incorporated the access rate that varied by frailty category: 9.6% if Fit; 23.5% Mild; 51.7% Moderate; and 72.7% Severe.

Under RC, eligibility is expanded to all MA fall patients regardless of cognitive status and intervention history. The intervention supply would expand without barrier to accommodate all eligible persons, but the final access rate would depend on demand. The uptake rates from relevant UK-based RCTs were used as measures of demand: 53.8% for cognitively intact persons [13]; and 49.6% for cognitively impaired [15]. There was no data on the individual-level variation in the uptake rates.

### Proactive pathway

Table B26 summarises the eligibility and access conditions under UC and RC for three components of the proactive pathway: (1) routine GP contact; (2) falls risk screening at GP contact; and (3) access to appropriate falls prevention intervention for high-risk, eligible individuals.

| **Table B26** Eligibility and access rates for the proactive falls prevention pathway under usual care and recommended care scenarios. | | |
| --- | --- | --- |
|  | **Usual care** | **Recommended care** |
| **Pathway component: Routine GP contact** | | |
| Eligibility | All persons not receiving reactive intervention that year | All persons not receiving reactive intervention that year |
| Access rate | Limited access rate according to ELSA: 81.3% overall; varying by individual-level characteristics (see Table B27) | Limited access rate according to ELSA: 81.3% overall; varying by individual-level characteristics (see Table B27) |
| **Pathway component: Fall risk screening at routine GP contact** | | |
| Eligibility | All persons at routine contact | All persons at routine contact |
| Access rate | Limited access rate by frailty category according to ELSA: 15.7% overall among those with recurrent non-MA or MA falls history; 12.0% if Fit; 15.1% if Mild; 19.7% if Moderate; 21.9% if Severe | 100% access |
| **Pathway component: Proactive falls prevention intervention** | | |
| Eligibility | Cognitively intact persons without intervention history; prioritisation by unknown criteria in ELSA (see Access rate) | Individuals at high risk by NICE criteria^1^ and varying by cognitive status:  (i) Multifactorial intervention for cognitively intact persons, up to three re-receipts  (ii) Tai Chi for cognitively impaired with or without intervention history |
| Access rate | Limited access rate by frailty category according to ELSA: 33.5% overall among cognitively intact persons who received falls risk screening; 10.9% if Fit; 24.7% if Mild; 53.6% if Moderate; 100% if Severe | All eligible persons pending demand: 82.4% uptake rate for (i) [17]; 44.5% for (ii) [23] |
| **Abbreviation:** ELSA: English Longitudinal Study of Ageing; NICE: National Institute for Health and Clinical Excellence  ^1^ Recurrent non-MA falls or MA fall history and/or abnormal gait/balance | | |

Under both UC and RC, the proactive pathway is initiated through routine GP contact at which older persons are screened for falls risk. ELSA contained a variable for whether the individual received a blood pressure check at the GP in the past 12 months. This was used as an indicator of at least one GP visit in a year. According to this measure, 83.8% of the ELSA Wave 4 community-dwelling cohort aged 65+ had visited their GP at least once in the past year, which is slightly below the 87.4% estimated from another survey of 1,685 adults aged 65+ in Northwest England [63]. Logistic regression indicated that the probability of GP visit was significantly associated with several covariates. Table B27 shows the coefficient estimates from the best-fit model. The sample was restricted to those who did not receive a reactive falls prevention intervention that year and hence qualified for the proactive pathway.

| **Table B27** Logistic regression coefficients for GP contact from ELSA Waves 4 and 5. | | |
| --- | --- | --- |
| ***Dependent variable: routine GP contact (N=13,280)^1^*** | | |
| **Explanatory variables** | **Coefficient (SE)** | **P-value** |
| Constant | -17.444 (1.960) | <0.001 |
| Age | 0.495 (0.054) | <0.001 |
| Age^2 | -0.003 (0.0004) | <0.001 |
| Female | -0.212 (0.049) | <0.001 |
| SES (ref: Most privileged quartile) |  |  |
| *2^nd^ quartile* | 0.132 (0.066) | 0.045 |
| *3^rd^ quartile* | 0.118 (0.059) | 0.045 |
| *Most deprived quartile* | 0.223 (0.088) | 0.012 |
| Falls history (ref: No falls history) |  |  |
| *Single non-MA fall* | 0.172 (0.082) | 0.036 |
| *Recurrent non-MA falls* | 0.450 (0.108) | <0.001 |
| *Single MA fall* | 0.701 (0.161) | <0.001 |
| *Recurrent falls with MA* | 0.930 (0.242) | <0.001 |
| Frailty (0-100) | 0.175 (0.008) | <0.001 |
| Frailty^2 | -0.002 (0.0002) | <0.001 |
| Cognitive impairment | -0.944 (0.058) | <0.001 |
| Fear of falling | 0.339 (0.141) | 0.016 |
| Abnormal gait/balance | -0.455 (0.076) | <0.001 |
| Community healthcare | -0.933 (0.341) | 0.006 |
| Social care | -1.023 (0.179) | <0.001 |
| OOP care | -0.300 (0.156) | 0.055 |
| Informal care | -0.345 (0.072) | <0.001 |
| **Abbreviation:** ELSA: English Longitudinal Study of Ageing; MA fall: fall requiring medical attention; OOP: out-of-pocket; Ref: reference; SE: standard error; SES: socioeconomic status  ^1^ Sample was restricted to those who did not receive reactive falls prevention intervention that year. | | |

Older individuals were likelier to visit the GP, and women less so. There was a SES gradient in GP contact in favour of more deprived quartiles. Falls of all types were predictors of GP contact, suggesting that GPs are well-positioned to identify high-risk individuals with fall history. But those with abnormal gait/balance were less likely to visit their GPs which reduces the probability of GPs identifying high-risk individuals. Higher frailty score was associated with increased probability. Cognitively impaired persons were less likely to visit, while those with fear of falling were likelier. There were also significant associations between receipt of other forms of care and the GP visit probability.

For falls risk screening at GP contact, all persons in the target population (aged 60+) were assumed to be eligible. This deviates from the NICE recommendation that screening be targeted at those aged 65+ [8], but was consistent with the stakeholder preference for early prevention. Cognitively impaired persons and those with intervention history still received falls risk screening under UC even though they would not be referred to the multifactorial intervention. This was consistent with the falls specialist geriatrician’s opinion that falls risk among these subgroups should still be monitored in primary care even if they are referred to different services. The key difference between UC and RC was the level of screening access. To parameterise the screening access under UC, ELSA data was used. As for the reactive pathway, a composite variable was created from ELSA to measure whether the individual had received fall risk screening in the past year. According to this variable, 31.4% of the ELSA cohort aged 60+ who had a history of recurrent non-MA falls or MA fall(s) (but did not receive the reactive intervention) received falls risk screening.^[[2]](#footnote-2)^ A multivariate regression showed no significant association between access rate and individual characteristics except for frailty. Therefore, the modelled rate varied by frailty (given recurrent non-MA or MA falls history): 12.0% if Fit; 15.1% Mild; 19.7% Moderate; and 21.9% Severe. The rate was 100% for RC.

The referral to falls prevention intervention after the screening differed significantly between UC and RC. Under UC, only cognitively intact persons without intervention history were eligible for multifactorial intervention. ELSA contained variables that indicated the receipt of physiotherapy and/or HAM. These were used to create a composite variable which showed that 33.5% of cognitively intact individuals who received falls risk screening subsequently received any falls prevention treatment. The recipients comprised 1.0% of the ELSA cohort aged 65+, which, if applied to the Sheffield population, would constitute around 1,000 clients per year. This is consistent with the currently low access rate in Sheffield. A multivariate regression showed no significant association between access rate and individual characteristics except for frailty. Therefore, the modelled access rate varied by frailty category: 10.9% if Fit; 24.7% Mild; 53.6% Moderate; and 100% Severe. Those with proactive intervention history were excluded from referral from the second model cycle.

Under RC, all individuals deemed to be at high falls risk were eligible for proactive intervention according to their cognitive status. The NICE criteria were used to determine high falls risk: i.e., recurrent non-MA falls history or MA fall history without reactive intervention access and/or abnormal gait/balance [8]. The intervention supply would expand to accommodate all referred persons, but the final access would depend on demand estimated from uptake rates in UK-based RCTs: 82.4% for cognitively intact persons [17]; and 44.5% for cognitively impaired [23]. The uptake rates were assumed not to vary by other characteristics such as frailty.

### Self-referred pathway

Table B28 summarises the eligibility and access conditions for the self-referred falls prevention pathway under UC and RC.

| **Table B28** Eligibility and access rates for the self-referred falls prevention pathway under usual care and recommended care scenarios. | | |
| --- | --- | --- |
|  | **Usual care** | **Recommended care** |
| **Pathway component: Self-referred exercise intervention** | | |
| Eligibility | All persons not receiving reactive or proactive intervention that year | All persons not receiving reactive or proactive intervention that year |
| Access rate | Limited access rate:  0.1% among most privileged SES quartile | All eligible persons pending demand: uptake rate according to ELSA; 9.5% overall; varying by individual-level characteristics (see Table B29) |
| **Abbreviation:** ELSA: English Longitudinal Study of Ageing; SES: socioeconomic status | | |

Under both scenarios, all persons not receiving reactive or proactive intervention that year are eligible for the intervention. For the final access rate, UC should portray the very low uptake of the Dance to Health programme (20-30 regular attendees) under current practice. According to qualitative research, current attendance was concentrated in a well-off neighbourhood with participants who can self-finance the intervention [64]. Therefore, the model assumed that 0.1% of individuals in the most privileged SES quartile (around 30 individuals) would enrol in self-referred exercise.

For the access rate under RC, ELSA was used to parameterise the uptake rate. ELSA contained variables which together indicated whether an individual is currently participating in an exercise or PT session. It was unclear whether these sessions were evidence-based falls prevention exercises or ‘conventional’ exercises for general physical difficulties. The overall uptake rate of 9.5% among those aged 60+ was substantially higher than that known for Dance to Health in Sheffield. Therefore, it was assumed that: (i) the ELSA variable captured the uptake rate for conventional exercise; and (ii) the uptake rate represented a *latent* demand for falls prevention exercise. Assumption (i) means that the uptake of *falls prevention* exercise remains low in UC. Assumption (ii) holds that if falls prevention exercise is commissioned and marketed on a wide scale, those who would take up conventional exercise would take up falls prevention exercise as a substitute or complement. This is reasonable given that falls prevention exercises typically aim to resemble conventional leisure programmes to promote uptake [62]. Indeed, the qualitative research participants did not draw a clear distinction between conventional and falls prevention exercises and cited previous/concurrent engagement in other exercises as a facilitator for falls prevention exercise uptake [64].

Another issue is that the ELSA variable may better reflect current *supply* rather than demand conditions for exercise. In other words, the access rate would be higher than 9.5% under RC with unlimited supply. It is also unclear whether the variable better reflects recommendations (or direct referrals) made by care professionals than ‘pure’ self-referrals. But given the frequent contact between older persons and those in supportive roles, external facilitation is a constant feature of geriatric health promotion. Nevertheless, the ELSA uptake rate was similar to that observed in a UK trial of falls prevention exercise that mainly relied on community marketing for recruitment [18]: among those aged 65+ with less than three falls in the past year, the ELSA uptake rate was 8.8% compared to 6.1% in the trial.

A multivariate logistic regression was estimated to assess the variation in exercise uptake. Table B29 shows the results. Older individuals and women were likelier to take up exercise. There was a SES gradient to uptake with the bottom two SES quartiles less likely to take up the intervention than the top two. Falls history of any type was associated with higher uptake. Interestingly, higher frailty and high physical activity were both associated with greater likelihood. Cognitively impaired persons were less likely to take up exercise, though association was not statistically significant at 95% confidence level. Those with abnormal gait/balance were less likely to take up, suggesting that these high-risk individuals may be disadvantaged under the self-referred pathway (if they fail to enter the proactive pathway). Finally, all forms of care contact in the community setting were associated with greater likelihood of exercise uptake, suggesting that supportive environment facilitated health promotion.

| **Table B29** Logistic regression coefficients for self-referred exercise uptake from ELSA Waves 4 and 5. | | |
| --- | --- | --- |
| ***Dependent variable: exercise uptake (N=13,173)^1^*** | | |
| **Explanatory variables** | **Coefficient (SE)** | **P-value** |
| Constant | -11.206 (2.665) | <0.001 |
| Age | 0.209 (0.074) | 0.005 |
| Age^2 | -0.002 (0.0005) | 0.001 |
| Female | 0.781 (0.067) | <0.001 |
| SES (ref: Most privileged quartile) |  |  |
| *2^nd^ quartile* | 0.038 (0.082) | 0.645 |
| *3^rd^ quartile* | -0.276 (0.076) | <0.001 |
| *Most deprived quartile* | -0.409 (0.108) | <0.001 |
| Falls history (ref: No falls history) |  |  |
| *Single non-MA fall* | 0.269 (0.093) | 0.004 |
| *Recurrent non-MA falls* | 0.246 (0.105) | 0.019 |
| *Single MA fall* | 0.434 (0.136) | 0.001 |
| *Recurrent falls with MA* | 0.549 (0.158) | 0.001 |
| Frailty (0-100) | 0.081 (0.010) | <0.001 |
| Frailty^2 | -0.001 (0.0002) | <0.001 |
| High physical activity | 0.763 (0.080) | <0.001 |
| Cognitive impairment | -0.151 (0.084) | 0.072 |
| Abnormal gait/balance | -0.187 (0.088) | 0.035 |
| Community healthcare | 0.743 (0.371) | 0.045 |
| Social care | 0.485 (0.194) | 0.012 |
| OOP care | 0.609 (0.134) | <0.001 |
| Informal care | 0.412 (0.077) | <0.001 |
| GP routine contact | 0.213 (0.093) | 0.022 |
| **Abbreviation:** ELSA: English Longitudinal Study of Ageing; MA fall: fall requiring medical attention; OOP: out-of-pocket; Ref: reference; SE: standard error; SES: socioeconomic status  ^1^ Sample was restricted to those who did not receive reactive or proactive falls prevention intervention that year. | | |

## B5.3 Intervention resource use and cost

Falls prevention interventions under UC and RC can be divided into two groups: multifactorial interventions for reactive and proactive pathways; and single-component interventions including HAM for the reactive pathway and exercise for proactive and self-referred pathways. The two groups have marked differences in resource and cost structures. Multifactorial interventions typically require in-house facilities and equipment with major fixed cost components [65], although several component services may be outsourced to other clinical facilities [66]. The NHS professionals in the multidisciplinary team likely receive salaries that do not vary directly with the client numbers [55]. By contrast, single-component interventions, particularly exercise, may not operate at a single dedicated venue and their professionals are likely paid at piece rates per session/hour [28]. The model should assign similar type and volume of resources as RCTs to derive corresponding efficacies.

### Multifactorial interventions – resource use

Table B30 summarises the intervention resource use detailed in each of the three UK-based RCTs that evaluated multifactorial interventions: Close (1999) [13] and Shaw (2003) [15] under the reactive pathway; and Spice (2009) [17] under the proactive. The resource uses are catalogued by three components: participant screening and recruitment; falls risk assessment; and therapeutic treatment. Strictly research-related resource uses such as phone calls to collect falls diaries are excluded.

| **Table B30** Resource use in UK-based randomised controlled trials of multifactorial interventions. | | | |
| --- | --- | --- | --- |
| **RCT reference** | **Intervention type** | **Component** | **Resource use** |
| Close (1999) [13] | Reactive multifactorial intervention for cognitively intact persons at MA fall presentation | Participant screening & recruitment^1^ | (1) Eligibility screening by researcher using A&E records  (2) Invitation by letter with information sheet and telephone call to answer questions |
|  |  | Falls risk assessment | Assessments conducted by one physician at a day hospital  (A) Medical and cardiovascular assessments: balance; vision; medication; affect; cognition; postural hypotension; cardiac arrhythmias; carotid sinus hypersensitivity  (B) OT assessment at home: function; environmental hazards; psychological status after fall |
|  |  | Therapeutic treatment | (I) In-house treatments: 92.1% received HAM and falls prevention education by OT  (II) Outsourced treatments: 44.1% to specialist services in outpatient setting; 25.0% to multidisciplinary falls clinic in day hospital; 21.7% referred to GPs mainly for medication modification; 17.8% to optician/ophthalmologist; 15.8% received no referrals; major home modifications referred to social services |
| Shaw (2003) [15] | Reactive multifactorial intervention for cognitively impaired persons at MA fall presentation | Participant screening & recruitment^2^ | (1) 52-week eligibility screening at A&E  (2) Participant consent sought from patient, immediate caregiver and next of kin |
|  |  | Falls risk assessment | Assessments conducted at a secondary care clinic  (A) Medical and cardiovascular assessments: medication; vision; depression; epilepsy; cerebrovascular; orthostatic hypotension; carotid sinus hypersensitivity; syncope  (B) PT assessment: gait; balance; feet; footwear; walking aid  (C) OT assessment: environmental hazards |
|  |  | Therapeutic treatment | (I) In-house treatments: 96.2% prescribed balance treatment and 90.0% gait treatment contained in 3-month PT-supervised home exercise, continued by caregiver; 80.8% prescribed HAM; 70.8% prescribed medication modification; 35.4% prescribed treatment for orthostatic hypotension; 10.8% prescribed treatment for carotid sinus hypersensitivity  (II) Outsourced treatments: 28.5% had feet or footwear problems addressed by chiropody; 17.7% had vision problems addressed by optician or ophthalmologist; 6.9% had depression addressed by psychogeriatric assessment |
| Spice (2009) [17] | Proactive multifactorial intervention for cognitively intact persons at high falls risk | Participant screening & recruitment^3^ | (1) Consent by 19 GP practices in same PCT area  (2) GP nurse conducted baseline risk screening: AMT; Barthel index; TUG test; medical history; falls history; osteoporosis risk factors  (3) Information leaflet given to older persons for participation consent |
|  |  | Falls risk assessment | Assessments conducted at a one-stop multidisciplinary clinic  (A) Medical and cardiovascular assessments: medication; vision; alcohol; neurological; musculoskeletal; postural hypotension; other cardiovascular  (B) PT assessment: mobility  (C) OT assessment: environmental hazards; feet/footwear |
|  |  | Therapeutic treatment | (I) In-house treatments: 53.6% received any PT treatment; 51.8% received any medication modification; 33.8% received any OT treatment; 3.6% received any nursing treatment |
| **Abbreviation:** AMT: Abbreviated Mental Test; HAM: home assessment and modification; MA fall: fall requiring medical attention; OT: occupational therapy/therapist; PCT: primary care trust; PT: physiotherapy/therapist; RCT: randomised controlled trial; TUG: Timed Up and Go test  ^1^ Uptake rate was 53.8%.  ^2^ Uptake rate was 46.9%.  ^3^ Uptake rate was 82.4%. | | | |

All three RCTs allocated resources to participant screening and recruitment. The reactive interventions used A&E admission records for eligibility screening, while the proactive intervention relied on screening by GP nurses. MA fallers did not automatically participate in the reactive interventions and resources were needed for their recruitment. Falls risk assessments comprised three main types: (A) medical and cardiovascular assessments; (B) PT assessments; and (C) OT assessments. The range of risk factors covered was broadly similar for the three RCTs, but the method of delivery differed. Close (1999) referred only 25% of intervention participants to a full multidisciplinary assessment at falls clinic, while the rest received a bi-disciplinary assessment by physician and OT. By contrast, Shaw (2003) and Spice (2009) conducted all assessments at multidisciplinary clinics.

The therapeutic treatments based on identified risk factors were either delivered in-house or outsourced to external providers. The proportion delivered in-house differed between RCTs. In Close (1999), only HAM and falls prevention education were delivered in-house by the OT. By contrast, Spice (2009) mentioned no external referrals, suggesting that most treatments were delivered in-house at the multidisciplinary clinic. Shaw (2003) mentioned external referrals for several treatments including chiropody, vision correction, and geriatric psychiatry.

The RCTs provided little details on the timeframe and professional workload associated with intervention delivery. Close (1999) mentioned that a single physician carried out the medical assessments for all 184 intervention participants completed within three weeks of A&E discharge. The OT assessment and treatments were delivered within a single home visit per patient by the OT. The study did not report what assessments and treatments were prescribed for the 25% of intervention participants who were referred to the multidisciplinary falls clinic. Shaw (2003) did not report how many and which professionals were involved in the delivery of multidisciplinary assessment and treatments. It nonetheless reported that all assessments and treatments were completed by the three-month follow-up. The home-based exercise was the longest component, delivered by PT for three months and ideally sustained thereafter by participants’ caregivers. Spice (2009) provided no detail on the workflow except the proportions of participants receiving different treatment components.

It was overall clear that full implementation of multifactorial interventions would rely extensively on referrals to multidisciplinary falls clinics. The falls specialist geriatrician at the Sheffield Falls Clinic confirmed that his team had the expertise, equipment, and links to other specialist services to deliver all assessment and treatment components recommended by NICE CG161. It is hence plausible that a group of multidisciplinary falls clinics – each clinic equipped to operate like the current Sheffield Falls Clinic (but on an expanded scale) – could cater to both reactive and proactive referrals.

### Multifactorial interventions – cost

Table B31 shows the estimated annual cost of delivering the multifactorial interventions. The costing is divided into two parts: (1) referral to multidisciplinary falls clinic; and (2) bi-disciplinary assessment and treatment for 75% of cognitively intact MA fallers under the reactive pathway [13].

| **Table B31** Resource use and cost of multifactorial interventions. | | | |
| --- | --- | --- | --- |
| **Component** | **Resource use** | **Mean cost^1^** | **Reference** |
| **(1) Referral to multidisciplinary falls clinic (21,000 clients; 3,000 per clinic per year)** | | | |
| Risk screening and access | Reactive clients – A&E record screening for eligibility and invitation by letter/phone^2^ | £16.37 per client | [66] |
|  | Proactive clients – TUG test set-up and staff time and training^3^ | £10.83 per client | [42] |
|  | Bi-disciplinary assessment for 25% of cognitively intact reactive clients (n=903) | £121.46 per client | [66] |
| Falls clinic operation | 1 falls specialist geriatrician (medical consultant) – salary & oncosts | £121,393 annual fixed | [55] |
|  | 2 registrars – salary & oncosts | £112,644 annual fixed | [55] |
|  | 2 PTs (band 6 & 7) – salary & oncosts | £94,906 annual fixed | [55] |
|  | 2 OTs (band 6 & 7) – salary & oncosts | £94,906 annual fixed | [55] |
|  | 2 PT assistants (band 4) – salary & oncosts | £56,919 annual fixed | [55] |
|  | 2 OT assistants (band 4) – salary & oncosts | £56,919 annual fixed | [55] |
|  | 3 nurses (band 4) – salary & oncosts | £75,834 annual fixed | [55] |
|  | 1 falls prevention facilitator – salary & oncosts^4^ | £33,123 annual fixed | [55]; [65] |
|  | 2 administrative staffs – salary & oncosts^5^ | £58,754 annual fixed | [55] |
|  | Capital overheads – land and office^6^ | £9,256 annual fixed | [55] |
|  | Non-staff overheads – travel/transport, telephone, education and training, office supplies, clinical/general services, utilities^7^ | £400,898 annual fixed | [55] |
|  | ***Total cost per falls clinic*** | **£1,115,552 annual fixed** |  |
|  | ***Total cost for 7 falls clinics*** | **£7,808,864 annual fixed** |  |
| Outsourced services and equipment^8^ | 2 specialist outpatient visits for 30% of clients | £32.89 expected^9^ per client | [66] |
|  | Optician and/or ophthalmologist referral for 17.7% of clients | £51.33 expected per client | [66] |
|  | 1 podiatry visit for 17% of clients | £5.77 expected per client | [66] |
|  | Geriatric psychiatry for 6.9% of cognitively impaired clients | £0.16 expected per client | [66]; [15] |
|  | Walking aid for 25% of clients | £21.29 expected per client | [66] |
|  | Hip protectors for 17% of clients | £11.81 expected per client | [66] |
|  | New footwear for 19% of clients | £10.79 expected per client | [66] |
|  | HAM major modifications (social care) | £94.97 expected per client | [28] |
|  | Handouts and amenities | £22.71 per client | [65] |
|  | ***Total cost per client*** | **£251.73** |  |
| Non-public sector cost per participant | Travel cost | £62.45 per year | [65] |
|  | HAM major modifications (self-funded) | £94.97 per receipt | [28] |
|  | Time opportunity cost – if employed^10^ | £348.80 per year |  |
|  | Time opportunity cost – if engaged in regular unpaid work^10^ | £87.20 per year |  |
|  | Time opportunity cost – informal caregiver for CI client^11^ | £348.80 per year |  |
| **(2) Bi-disciplinary assessment and treatment [13] (2,700 clients per year)** | | | |
| Risk screening and access | Reactive pathway screening and invitation^2^ | £16.37 per client | [66] |
| Bi-disciplinary assessment | Physician assessment | £121.46 per client | [66] |
|  | OT assessment for 92.1% of clients | £269.63 expected per client | [66]; [13] |
| Outsourced services and equipment^8^ | Specialist outpatient visit for 44.1% of clients | £45.90 expected per client | [66]; [13] |
|  | 2 GP visits for medication change for 21.7% of clients | £16.63 expected per client | [66]; [13] |
|  | Optician and/or ophthalmologist referral for 17.8% of clients | £51.62 expected per client | [66]; [13] |
|  | HAM major modifications (social care) for 92.1% of clients | £87.47 expected per client | [28]; [13] |
| Non public sector cost per participant | Travel cost | £62.45 per year | [65] |
|  | HAM major modifications (self-funded) for 92.1% of clients | £87.47 expected per client | [28]; [13] |
|  | Time opportunity cost – if employed^12^ | £69.76 per year |  |
|  | Time opportunity cost – if engaged in regular unpaid work^12^ | £17.44 per year |  |
| **Abbreviation:** CI: cognitively impaired; HAM: home assessment and modification; MA fall: fall requiring medical attention; OT: occupational therapy/therapist; PT: physiotherapy/therapist; TUG: timed up and go  ^1^ All costs are expressed in 2021/22 £. Earlier estimates were inflated at the annual rate of 1.98% which is the average NHS cost inflation between 2013 and 2019 [55]. Costs in Australian dollar were converted to £ at rate of £0.55/AUS$1.  ^2^ This incorporated cost of 30 minutes of hospital staff time, 28% premium as cost of recruitment (i.e., letters and phone calls), and 50% premium as office overheads. The costs were converted from 2008/09 Australian dollar to 2021/22 £.  ^3^ This included cost of set-up (i.e., office overheads) at £24 per GP practice which amounted to £0.02 per person when spread across all recommended scenario recipients. The 28% premium for recruitment was applied as in reactive pathway (see note 2) to obtain the final per-participant cost of £10.83.  ^4^ Salary/oncosts of social work assistant from PSSRU depository were used [55].  ^5^ Salary/oncosts of administrative staff for medical consultant were used [55].  ^6^ Assumed to be four times the capital overheads for Dementia Memory Clinic operating 40 hours per week for 50.4 weeks per year and catering to 708 dementia patients as costed in PSSRU depository. The overheads were annuitized over 60 years at a discount of 3.5%, declining to 3% after 30 years [55].  ^7^ Assumed to be two times the non-staff overheads for Dementia Memory Clinic [55].  ^8^ Assumed that those with intervention history incurred 20% of the costs of outsourced services.  ^9^ Expected cost given the probability of receiving the given service.  ^10^ Assumed that average time committed per client is 40 hours: 2 hours for falls risk assessment at falls clinic; 4 hours for in-house medical treatments at falls clinic; 30 hours for in-house exercise treatments; 2 hours for HAM; 2 hours per outsourced services amounting to 2 hours on average per client (2 outpatient visits for 30%, optician/ophthalmologist visit for 17.7%, podiatry visit for 17%). Those in paid employment assumed to incur an hourly opportunity cost equal to the national living wage (£8.72). Those engaged in unpaid work assumed to incur an hourly opportunity cost equal to quarter of the national living wage (£2.18) due to shorter weekly working hours (11 vs. 40 hours for employed).  ^11^ Assumed that cognitively impaired clients are accompanied by their informal caregivers for all 40 hours of intervention, incurring an hourly opportunity cost equal to the national living wage.  ^12^ Assumed that average time committed per client is 8 hours: 2 hours for physician assessment; 4 hours for OT HAM and falls prevention education; and 2 hours per outsourced services. The hours were valued in the same way as in note 10. | | | |

Given the high fixed costs of falls clinics, it is important to estimate the total number of clinics required. For the reactive pathway under RC, all MA fallers would access the multifactorial intervention pending demand. According to ELSA Wave 4, 6.8% of the population aged 60+ experienced at least one MA fall in the past year and were eligible for the reactive intervention. This equates to 8,542 individuals out of 125,244 aged 60+ in Sheffield. Of these, 78.4% (n=6,695) were cognitively intact and the other 21.6% (n=1,847) impaired. According to the RCTs, the uptake rate for the reactive intervention was 53.8% for cognitively intact [13] and 49.6% for impaired [15]. Hence, 3,602 cognitively intact and 916 impaired persons would access the intervention. However, given that only 25% of cognitively intact MA fallers are referred to the clinic [13], the final client number for the clinic would be around 900, while the other 2,700 would receive bi-disciplinary intervention.

For the proactive pathway under RC, all cognitively intact individuals screened to be at high falls risk at GP contact are eligible for multifactorial intervention. Among those who did not experience an MA fall, 29.0% or 36,333 individuals in Sheffield had abnormal gait/balance or experienced recurrent non-MA falls in the past year and hence were at high falls risk. Of these, 71.9% (n=26,131) were cognitively intact and hence eligible for the multifactorial intervention, while the other 28.1% (n=10,202) were impaired. But not all eligible persons would have a GP contact in that year for the fall risk screening. The average annual probability of having a GP contact was 89.0% for the cognitively intact at high falls risk, meaning that 23,257 would be referred. For those referred, the uptake rate was 82.4% according to the RCT [17], meaning that the final number accessing the intervention would be 19,163.

Therefore, across both reactive and proactive pathways under RC, the total number accessing the falls clinic would be 20,979. Annual falls clinic capacity should thus be planned for 21,000 clients of whom 91.3% (n=19,173) are cognitively intact proactive clients, 4.4% (n=924) cognitively impaired reactive clients, and 4.3% (n=903) cognitively intact reactive clients. A further 2,700 clients would receive the bi-disciplinary intervention. The Sheffield Falls Clinic under current practice ran for a single afternoon per week and catered to around 300 clients per year. If the Clinic operates full-time (i.e., 10 morning and afternoon sessions), it would have an annual capacity of 3,000 clients. At this workflow, seven clinics would be needed to manage 21,000 clients per year. That said, initial model simulation showed that the clinic client flow averaged around 25,000 over 40 years due to the unlimited re-receipt of intervention by those with proactive intervention history. Capping the number of proactive intervention re-receipts to three returned the annual flow to around 21,000.

Resource uses and costs for referrals to multidisciplinary falls clinics were divided into four components: (i) risk screening and access; (ii) falls clinic operation; (iii) outsourced services and equipment; and (iv) non-public sector costs. The falls clinic operation was assumed to mainly incur fixed annual costs in the form of salaries and overheads, while other components were costed at per-client rates. This is a simplification since many of the latter components (e.g., specialist outpatient services) also operate in facilities incurring fixed costs. However, unlike the falls clinic, these facilities are not dedicated to falls prevention and hence the per-client rates were deemed more appropriate.

Two previous falls prevention models informed the per-client cost of risk screening, recruitment and physical assessment for the bi-disciplinary intervention [42, 66]. No cost was assigned to GP contact since it would be included in the annual comorbidity care costs for primary and secondary care. More details on the costing of the falls clinic operation is not reported here and is available upon request. In brief, the annual salary and overhead cost of operating a full-time clinic (40 hours per week) was estimated based on stakeholder consultations and literature. The PSSRU unit cost depository [55] served as an important data source. Treatments outsourced to external providers were costed at per-client rates. The proportion of multifactorial intervention clients requiring each outsourced service and cost per service were obtained from a previous model [66] to estimate the expected cost per service.

Based on consultation with the falls modelling expert, clients with previous receipt history were assumed to incur only 20% of the cost of outsourced services to reflect the lower number of newly identified falls risk factors at the repeat visit. Attending the falls clinic also incurred costs outside the public sector. The annual travel cost was sourced from a previous model [65]. Time opportunity costs were estimated for participants who are employed or engaged in unpaid work, and for informal caregivers accompanying cognitively impaired participants.

The bi-disciplinary intervention was costed at per-client rates. The RCT for the bi-disciplinary intervention reported the proportions of clients receiving different outsourced services [13]. These proportions were combined with unit costs from the previous model [66] to estimate the expected costs.

### Single-component interventions – resource use

Table B32 summarises the intervention resource use in RCTs for: reactive HAM for hip fracture patients [60]; and three exercise interventions for different subgroups [18, 19, 23].

| **Table B32** Resource use in randomised controlled trials of single-component interventions. | | | |
| --- | --- | --- | --- |
| **RCT reference** | **Intervention type** | **Category** | **Resources** |
| Lockwood (2019) [60] | HAM for hip fracture patients discharged from hospital (reactive pathway under usual care only) | Participant screening & recruitment | (1) Eligibility screening method unclear  (2) Hip fracture patients approached after surgery; consent given by family member if have moderate/severe cognitive impairment |
|  |  | Therapeutic treatment | (I) 1-hour OT home visit with patient (and family member if cognitively impaired) 1-5 days before discharge^1^ – assessed home safety, mobility, self-care and function, followed by education, advice and recommendation on home modifications, equipment and community services^2^ |
| Nyman (2020) [23] | Group- and home-based Tai Chi for dementia patients (proactive and self-referred pathways) | Participant screening & recruitment | (1) Potential participants identified from: NHS research/clinic databases; memory services; local charities; self-referrals  (2) Eligibility confirmed and consent obtained from participant and caregiver at home visit by researcher  (3) Reimbursed travel cost for intervention |
|  |  | Therapeutic treatment | (I) Group Tai Chi: 90-minute (45-minute exercise and 45-minute informal discussion) weekly Tai Chi class (10 patient-caregiver dyads) for 20 weeks – delivered by qualified lead/assistant Tai Chi instructor; individually-tailored and progressing in difficulty  (II) Home-based Tai Chi: 20-minute daily Tai Chi for 20 weeks – instructed by Tai Chi instructor and supervised by caregiver  (III) Behavioural change techniques: e.g., solutions to personal barriers, self-monitoring, peer support  (Note) Programme designed to accumulate 50 hours of exercise over 20 weeks; the mean number of hours spent in exercise was 25 |
| Iliffe (2014) [18] | Group-based FaME and home-based Otago exercises for cognitively intact persons at general falls risk (self-referred pathway) | Participant screening & recruitment | (1) GP practices with suitable venues for exercise classes created a list of potentially eligible older persons; random number generator selected 600 persons per GP practice; invitation letters were sent |
|  |  | Therapeutic treatment | (I) FaME: 1-hour weekly group class (up to 15 participants per class) for 24 weeks – delivered by PSI at local venues; individually-tailored and progressing in difficulty  (II) Otago home exercise: 30-minute exercise 2 times per week for 24 weeks using instruction booklets  (III) Walking: 30 minutes at moderate pace 2+ times per week for 24 weeks  (Note) 72 hours of exercise including walking over 24 weeks; 17% completed 75% of FaME and Otago exercise hours |
| Skelton (2005) [19] | Group-based FaME and home-based Otago exercises for cognitively intact women at high falls risk (self-referred pathway) | Participant screening & recruitment | (1) Direct community marketing: posters at NHS clinics and voluntary organizations; advertisement in local and national newspapers and local radio station  (2) Eligibility screening: initial telephone call to people who responded to marketing; postal questionnaire sent for further screening |
|  |  | Therapeutic treatment | (I) FaME: 1-hour weekly group class for 36 weeks – delivered by qualified geriatric exercise instructors with additional FaME training at local venues; individually tailored and progressing in difficulty  (II) Otago home exercise: 30-minute exercise 2 times per week for 36 weeks using instruction booklets  (Note) 72 hours of exercise over 24 weeks; adherence not reported but 68% continued exercising at trial end |
| **Abbreviation:** FaME: Falls Management Exercise; HAM: home assessment and modification; OT: occupational therapy/therapist; PSI: postural stability instructor  ^1^ Both intervention and control group participants received multidisciplinary inpatient rehabilitation and (depending on functional level) community rehabilitation after discharge. The OT who conducted the 1-hour home visit was also involved with the inpatient rehabilitation for the same patient.  ^2^ The study did not mention major home modifications being made at the single home visit or being financed by the evaluated intervention, suggesting that external social services were referred for major modifications with their costs being borne by the social care sector or by participants themselves. | | | |

Lockwood (2019) for reactive HAM did not clearly describe the eligibility screening method, perhaps because hip fracture patients were readily identifiable; but implementing the same intervention on all hospitalised MA fallers will require screening resources. The intervention consisted of an OT home visit 1-5 days before discharge to make safety assessment and relevant recommendations.

Nyman (2020) evaluated a 20-week group- and home-based Tai Chi intervention that was tailored to dementia patients. The participants were screened and recruited in a largely proactive manner, with dementia patients being identified from their routine contacts with clinical and nonclinical services, although self-referrals were also accepted. Those whose dementia was too severe to be able to give informed consent (assisted by the caregiver) were excluded from participation. Due to the initially low uptake, the intervention travel costs were reimbursed. There were three therapeutic components: group-based Tai Chi delivered by qualified Tai Chi instructor; home-based Tai Chi instructed by the instructor but supervised by the caregiver; and behavioural change techniques delivered by the instructor. The aim was to accumulate 50 hours of exercise per participant over 20 weeks. But dose adherence was low: the average hours of exercise was 25, with only three of 42 participants (7.1%) reaching 50 hours.

Iliffe (2014) evaluated a 24-week multi-component exercise intervention that targeted a relatively low falls risk (less than three falls in past year) cognitively intact older population. The recruitment was conducted via invitation letters; the access mode was hence self-referral. There were three therapeutic components: group-based FaME delivered by postural stability instructors (PSIs); self-implemented Otago home exercise; and encouragement of walking. The total number of exercise hours was 72 hours over 24 weeks. But adherence was low, with 17% completing 75% of FaME and Otago doses.

Skelton (2005) evaluated a 36-week multi-component exercise intervention that targeted a high falls risk (three or more falls in past year) cognitively intact older women. The trial recruitment relied on direct marketing – posters and newspaper and radio advertisements – consistent with the self-referred pathway. The therapeutic components were like those in Iliffe (2014), except that encouragement of walking was excluded. This meant that even though the overall number of exercise hours (72) was the same – spread across 36 weeks in Skelton (2005) vs. 24 weeks in Iliffe (2014) – the intervention in Skelton (2005) was more intensive than that in Iliffe (2014), composed entirely of FaME and Otago. The adherence rates were not reported.

### Single-component interventions – cost

Table B33 shows the resource use and cost parameters for single-component interventions.

| **Table B33** Resource use and cost of single-component interventions. | | | |
| --- | --- | --- | --- |
| **Component** | **Resource use** | **Mean cost^1^** | **Reference** |
| **(1) Reactive HAM under usual care** | | | |
| Risk screening and access | Hospital record screening for eligibility and invitation before discharge | £16.37 per client | [66] |
| Therapeutic component | OT (band 7) home visit and assessment (2 hours including travel) | £128.23 per client | [55] |
|  | HAM major modifications (social care) | £94.97 per client | [28] |
|  | Walking aid for 25% of clients | £21.29 expected^2^ per client | [66] |
|  | Hip protectors for 17% of clients | £11.81 expected per client | [66] |
|  | New footwear for 19% of clients | £10.79 expected per client | [66] |
|  | 2 referrals to community OT service for 20% of clients | £19.97 expected per client | [55] |
| Non-public sector cost per client | HAM major modifications (self-funded) | £94.97 | [28] |
|  | Time opportunity cost – informal caregiver for CI patient^3^ | £17.44 per year |  |
| Public sector cost | **Average cost per client** | **£303.43 per year** |  |
|  | **Average cost per client with intervention history^4^** | **£176.37 per year** |  |
| **(2) 20-week Tai Chi for cognitively impaired persons and their caregivers** | | | |
| Risk screening and access | Screening at GP contact (proactive only) | £10.83 per client | [42] |
|  | Travel cost (proactive only) | £62.45 per client | [65] |
|  | Ongoing administration^5^ | £50 per client | PCS |
|  | Direct marketing (self-referred only) | £25.31 per client | [66] |
| Therapeutic component | Staff time | £367.06 per client | [23] |
|  | Staff training | £8.25 per client | [28] |
|  | Staff travel | £15.01 per client | [28] |
|  | Equipment | £5 per client | [28] |
|  | Venue hire | £30 per client | [28] |
| Non public sector cost per client | Travel cost (self-referred only) | £62.45 | [65] |
|  | Time opportunity cost – if employed^6^ | £218 per year |  |
|  | Time opportunity cost – if engaged in regular unpaid work^6^ | £54.5 per year |  |
|  | Time opportunity cost – informal caregiver^7^ | £218 per year |  |
| Public sector cost | **Average cost per proactive client** | **£537.77 per year** |  |
|  | **Average cost per self-referred client^8^** | **£500.63 per year** |  |
| **(3) 24-week self-referred FaME and Otago for general-risk cognitively intact persons** | | | |
| Access | Direct marketing | £10.03 per client | [67] |
| Therapeutic component | Staff time | £126 per client | PCS |
|  | Staff training | £4.60 per client | [28] |
|  | Staff travel | £21.11 per client | [28] |
|  | Equipment | £20.02 per client | [28] |
|  | Venue hire | £18 per client | [28] |
| Non public sector cost per client | Travel cost | £62.45 | [65] |
|  | Time opportunity cost – if employed^9^ | £340.60 per year | [18] |
|  | Time opportunity cost – if engaged in regular unpaid work^9^ | £85.15 per year | [18] |
| Public sector cost | **Average cost per client** | **£199.76 per year** |  |
| **(4) 36-week self-referred FaME and Otago for high-risk cognitively intact persons** | | | |
| Access | Direct marketing | £10.03 per client | [67] |
| Therapeutic component | Staff time | £189 per client | PCS |
|  | Staff training | £4.60 per client | [28] |
|  | Staff travel | £31.66 per client | [28] |
|  | Equipment | £20.02 per client | [28] |
|  | Venue hire | £27 per client | [28] |
| Non public sector cost per client | Travel cost^10^ | £93.68 | [65] |
|  | Time opportunity cost – if employed^9^ | £340.60 per year | [18] |
|  | Time opportunity cost – if engaged in regular unpaid work^9^ | £85.15 per year | [18] |
| Public sector cost | **Average cost per client** | **£272.28 per year** |  |
| **Abbreviation:** FaME: Falls Management Exercise; HAM: home assessment and modification; OT: occupational therapy/therapist; PCS: personal communication with stakeholder  ^1^ All costs are expressed in 2021/22 £. Earlier estimates were inflated at the annual rate of 1.98% which is the average NHS cost inflation between 2013 and 2019 [55]. Costs in Australian dollar were converted to £ at £0.55/AUS$1.  ^2^ Expected cost given the probability of receiving the given service.  ^3^ Assumed that cognitively impaired patients are accompanied by informal caregivers for 2 hours of OT home visit, incurring an hourly opportunity cost equal to the national living wage (£8.72).  ^4^ Assumed that those with intervention history incurred 20% of the costs of HAM major modifications, equipment and community OT services.  ^5^ The manager for Dance to Health Sheffield informed that each weekly session for 20 participants incurred an administrative cost of £25, which equates to £500 for a 20-week programme. This translates to £50 per client if each Tai Chi class is attended by 10 participant-caregiver dyads as mentioned in Nyman (2020).  ^6^ Assumed that average time committed per participant is 25 hours according to the average exercise time in RCT [23]. Those in paid employment assumed to incur an hourly opportunity cost equal to the national living wage. Those engaged in unpaid work assumed to incur an hourly opportunity cost equal to quarter of the national living wage.  ^7^ Assumed that cognitively impaired clients are accompanied by their informal caregivers for all 25 hours of intervention, incurring an hourly opportunity cost equal to the national living wage.  ^8^ Under usual care, the cost of self-referred intervention is entirely self-paid.  ^9^ According to Iliffe (2014) [18], 17% of general-risk persons receiving the 24-week intervention completed 75% of prescribed time for FaME and Otago exercises. Assuming the other 83% completed 50% of prescribed time and that the pattern was the same for 24-hour walking, then the average time committed was 39.06 hours. If the same pattern was applied to the 36-week intervention evaluated by Skelton (2005) [19] which contained 72 hours of FaME and Otago, the average time committed was again 39.06 hours. Time opportunity costs were assigned as in note 6.  ^10^ Travel cost was assumed to be proportional to the programme duration. Hence, the 36-week programme for high-risk group incurred 1.5 times the travel cost of the 24-week programme for general-risk. | | | |

For reactive HAM under UC, the cost of screening was taken from a previous model [66]. In the intervention RCT, each OT home visit lasted one hour [60]. But the OT involvement during the inpatient stay would have reduced the required length of home visit. The RCT also excluded travel time which was costed separately for HAM in previous models [28, 66]. A simplifying assumption was made that each OT home visit lasted two hours including travel time. The hourly cost for OT band 7 was obtained from PSSRU [55]. The costs of major home modifications, sourced from a previous model [28], were split equally between social care sector and participants themselves. The home visit also resulted in equipment recommendations and community service referrals. Further details on costing the equipment and services are not reported here and are available upon request. Patients with history of reactive HAM incurred 20% of the equipment/service costs. Lockwood (2019) described cognitively impaired patients being accompanied by their caregivers at the OT home visit. Hence, HAM incurred the opportunity cost of two hours of caregivers’ time. It was assumed that hospitalised MA fallers engaged in minimal paid/unpaid work and hence incurred no time opportunity cost.

For the three exercise interventions, the dosage costed for the year was that evaluated in their respective RCTs. The Tai Chi operated under both proactive and self-referred pathways. The former would incur the cost of falls risk screening taken from a previous model [42]. Nyman (2020) also mentioned that participants’ travel costs were reimbursed to increase uptake. Hence, participant travel costs, taken from a previous model [65], accrued to the public sector under the proactive pathway but not under the self-referred pathway. The administration cost was included at per-client rate. The cost of direct marketing under the self-referred pathway was taken from a previous model [66]. The marketing cost for FaME and Otago exercises targeting cognitively intact persons was assumed to be lower than that for cognitively impaired Tai Chi participants and taken from a previous economic evaluation [67]. The cost categories for the therapeutic components of all three exercises were taken from the PHE model [28]: staff time; staff training; staff travel; equipment; and venue hire. Further details on costing the therapeutic components are available upon request. The costs were assumed not to vary by intervention history. Societal costs included travel cost (except for proactive Tai Chi) which was assumed proportional to the programme duration. Time opportunity costs for those in paid/unpaid work and for accompanying caregivers were assumed proportional to the number of hours committed.

## B5.4 Intervention efficacy

The RCT efficacy evidence should match the model specification in terms of: (i) the main fall event of interest – e.g., any fall, recurrent fall, MA fall; (ii) the incidence metric – relative risk (RR) for falls risk reduction and rate ratio (RaR) for falls rate reduction [68]; and (iii) efficacy duration – i.e., no longer than the RCT duration, unless implementation is sustained [53]. Table B34 summarises the efficacy data extracted from seven RCTs and one Cochrane meta-analysis.

| **Table B34** Falls prevention intervention efficacy parameters for base case analysis (usual care vs. recommended care scenarios). | | | | | |
| --- | --- | --- | --- | --- | --- |
| **Target population** | **Intervention** | **RCT source** | **Follow-up duration** | **Efficacy type** | **RR (95% CInt)** |
| ***Reactive pathway*** | | | | | |
| MA fall patients (UC) | HAM | Lockwood (2009) [60] | 6 months | RR – any faller | 0.828 (0.472-1.452) |
|  |  |  |  | RR – recurrent faller given any fall^1^ | 1.383 (0.788-2.427) |
| Cognitively intact MA fall patients (RC) | Multifactorial intervention | Close (1999) [13] | 12 months | RR – any faller | 0.615 (0.485-0.793) |
|  |  |  |  | RR – recurrent faller given any fall^2^ | 0.719 (0.486-1.063) |
|  |  |  |  | RR – MA faller given any fall | 0.942 (0.428-2.071) |
| Cognitively impaired MA fall patients (RC) | Multifactorial intervention | Shaw (2003) [15] | 12 months | RR – any faller | 0.925 (0.811-1.054) |
|  |  |  |  | RR – recurrent faller given any fall^1^ | 1.183 (1.037-1.349) |
| ***Proactive pathway*** | | | | | |
| Cognitively intact high-risk persons | Multifactorial intervention | Spice (2009) [17] | 12 months | RR – any faller | 0.874 (0.778-0.982) |
|  |  |  |  | RR – MA faller given any fall^3^ | 0.854 (0.441-1.652) |
| Cognitively impaired high-risk persons (RC) | Tai Chi | Nyman (2020) [23] | 6 months | RR – any faller | 1.024 (0.608-1.723) |
|  |  |  |  | RR – recurrent faller given any fall^1^ | 0.379 (0.225-0.638) |
| ***Self-referred pathway*** | | | | | |
| Cognitively intact general-risk persons | Exercise | Sherrington (2019) [12] | 3-25 months | RR – any faller | 0.850 (0.810-0.890) |
|  |  | Iliffe (2014) [18] | 12 months | RR – recurrent faller given any fall^4^ | 0.964 (0.921-1.009) |
|  |  |  |  | RR – engaging in high physical activity^5^ | 2.308 (1.156-4.909) |
| Cognitively intact high-risk persons | Exercise | Skelton (2005) [19] | 50 weeks | RR – any faller | 0.733 (0.567-0.946) |
|  |  |  |  | RR – recurrent faller given any fall^1^ | 0.863 (0.669-1.113) |
|  |  |  |  | RR – MA faller given any fall^6^ | 0.429 (0.190-0.968) |
| Cognitively impaired persons | Tai Chi | Nyman (2020) [23] |  | *See proactive pathway above* |  |
| **Abbreviation:** CInt: confidence interval; HAM: home assessment and modification; MA fall: fall requiring medical attention; OR: odds ratio; RaR: rate ratio; RC: recommended care; RCT: randomized controlled trial; RR: relative risk; UC: usual care.  ^1^ Calculated from the RR, RaR and SE for log(RR any faller) reported by the RCT and recurrent fall pattern value (A) of 0.903 from a UK RCT [16]; see Equation (2) for formula. The 95% CInt for RR^RF^ was obtained by assuming that the SE for log(RR any faller) equals SE for log(RR^RF^).  ^2^ Proxied by RR of experiencing 3+ falls given any fall reported by the same RCT.  ^3^ Proxied by RR of being hospitalized faller given any fall reported by the same RCT and assumed to apply to both single and recurrent fallers.  ^4^ Calculated from RaR reported by the RCT, RR and SE for log(RR any faller) from Cochrane meta-analysis [12] and A of 0.903. The 95% CInt for RR^RF^ was obtained by assuming that SE for log(RR any faller) equals SE for log(RR^RF^).  ^5^ Calculated from OR and baseline risk of 38% of control group being highly physically active from the RCT.  ^6^ Proxied by RR of being injurious faller given any fall reported by the RCT; applied to both single and recurrent fallers. | | | | | |

Several RCTs reported both RR and RaR for the intervention relative to comparator. Nyman (2020), for example, reported RR of 1.024 (95% confidence interval (CInt): 0.608-1.723) and RaR of 0.350 (95% CInt: 0.150-0.810) [23]. Hence, the intervention significantly reduces the total number of falls but not the number of fallers. This implies that the number of falls *per faller* is significantly lower, which in turn means that the number of recurrent fallers has declined among all fallers. Using either one of RR or RaR alone would neglect the information content of the other. Therefore, where the RCT reported both RaR and RR, the following equation (2) was used to estimate RR of being a recurrent faller given any fall, or RR^RF^, from the information in the two metrics:

$$RaR=RR\times{RR}^{RF}\times A$$

$$and A=\frac{1+Ratio'\cdot{RF\#}^{'}}{1+Ratio\cdot RF\#}\times\frac{Ratio}{Ratio'}\times\frac{APY}{APY'} (2)$$

where $A$ is a composite term expressing the pattern of falls among recurrent fallers (henceforth, ‘recurrent fall pattern’). $Ratio'$ and $Ratio$ are ratios between the numbers of recurrent fallers and single fallers in the intervention group and control group, respectively. ${RF\#}^{'}$ and $RF\#$ are the average numbers of falls experienced by recurrent fallers in the intervention group and control group, respectively. ${APY}^{'}$ and $APY$ are the average length of follow-up duration expressed in person-years for the intervention group and control group, respectively. The derivation of equation (2) is reported elsewhere and is available upon request. Only one UK RCT (not used for parameterisation) reported sufficient data to estimate the recurrent fall pattern of value 0.903 [16]. This estimate was assumed to hold for other RCTs when equation (2) was applied. In the example of Nyman (2020) given above, the RR of being a recurrent faller given any fall (*RR^RF^*) would be 0.350/(1.024*0.903) = 0.379. The intervention’s high efficacy in reducing the number of falls per faller thus translates to around 62% reduction in the risk of being a recurrent faller given any fall.

Where sufficient data were reported, RR of being an *MA faller* given any fall was computed. Technically, the model could have distinguished between RR of being an MA faller given a single fall and RR given recurrent falls, but no RCT provided sufficient detail for this distinction. The RR was hence applied to both single and recurrent fallers. No RCT reported RR of experiencing a fatal fall. The reliance on reported data meant that the evidence availability varied across RCTs: Lockwood (2009), for example, lacked the data for RR of being an MA faller given any fall, while Spice (2009) lacked it for RR^RF^. Yet the absence of reported evidence does not imply the absence of effect, and hence the absence is a model limitation. Iliffe (2014) provided the sole wider health effect of falls prevention, namely the higher likelihood of engaging in high physical activity. The OR was converted into RR and applied to the individual-level probability of engaging in high physical activity in the next cycle (see Section B7.3). Iliffe (2014) nevertheless lacked the data on RR of experiencing any fall; this was thus obtained from the Cochrane meta-analysis on community-based falls prevention exercise [12].

Five RCTs had follow-up durations of 12 months, while two had durations of six months. For the latter two, the model assumed that the six-month efficacies were durable for the year in which the interventions are accessed. This is a limitation since there is no evidence that the efficacies are durable in the second half of the year. A further caveat is the model’s lack of consideration of per-protocol efficacy evidence, incorporating intention-to-treat (ITT) efficacy data only. The per-protocol evidence would have captured heterogeneity in the intervention benefit access by adherence level. But only Nyman (2020) conducted per-protocol analysis and did not report its outcome.

# B6 Mortality risks and non-fatal falls epidemiology

After individuals have received their appropriate falls prevention interventions (if any), the model proceeds to generate the risk and incidence of mortality (Section B6.1), followed by the risk and incidence of falls of various types (B6.2). Intervention efficacies are applied to the intervention recipients after estimating their falls risks and before their falls incidence (if any).

## B6.1 Fatal falls and other-cause mortality

According to the 2019 Global Burden of Disease (GBD) study, falls constituted 0.76% of all deaths in men aged 50-69 in Sheffield; 0.45% in women aged 50-69; 1.09% in men aged 70+; and 0.96% in women aged 70+ [69]. From this information, estimates of all-cause mortality rates can be partitioned into fall-related and other-cause mortality rates. The costs of health and social care by cause of death in the final year of life were assigned to deceased individuals.

### Risk of fatal falls

The ONS Life Tables provided the annual all-cause mortality risks stratified by age and sex based on death incidence for the period 2016-2018 [25]. The Life Tables covered both community-dwelling and institutionalised populations who have significantly different mortality rates. A prospective cohort study of primary care records of 9,772 care home residents and 354,306 community-dwelling persons in England and Wales aged 65-104 reported one-year mortality rates for the two populations by age group [70]. From this data, the relative risks of death in an institutional setting compared to community were computed by age group: 10.67 for age 65-74; 5.51 for age 75-84; and 3.07 for age 85+. Using the relative mortality risks and the ELSA-estimated proportions of the total population institutionalised by age group and sex (Section B3.1), annual mortality rates were estimated for the community-dwelling and institutionalised populations. It was assumed that the relative risk for age 65-74 applied also to age 60-64. The weighted average of the two rates equalled the stratified rates in the ONS Life Tables.

The age- and sex-stratified annual mortality rates for the community-dwelling population were further stratified by frailty category. The UK study of eFI reported the hazard ratios of annual mortality (adjusted for age and sex) by frailty category: 1.92 (95% CInt: 1.81-2.04) for Mild relative to Fit; 3.10 (95% CInt: 2.91-3.31) for Moderate relative to Fit; and 4.52 (95% CInt: 4.16-4.91) for Severe relative to Fit [30]. Using the hazard ratios and the ELSA-estimated proportions of community-dwelling by frailty category in each age and sex subgroup, the frailty-stratified mortality rates were estimated for the community-dwelling population by age and sex. Having estimated the stratified mortality rates, the percentages reported by GBD 2019 were applied to estimate the rates of fatal falls. This assumed that: (i) GBD percentages for age group 50-69 applied equally to the age group 60-69; and (ii) GBD percentages did not vary by frailty. Table B35 shows the annual fatal fall rates stratified by age, sex, and frailty category incorporated in the model.

| **Table B35** Annual fatal fall rate (range 0-1) by age group, sex and frailty category. | | | |
| --- | --- | --- | --- |
| **Sex** | **Frailty** | **Age 60-69** | **Age 70+** |
| Male | Fit | 0.000063 | 0.000354 |
|  | Mild | 0.000121 | 0.000680 |
|  | Moderate | 0.000196 | 0.001097 |
|  | Severe | 0.000285 | 0.001600 |
| Female | Fit | 0.000024 | 0.000198 |
|  | Mild | 0.000046 | 0.000381 |
|  | Moderate | 0.000074 | 0.000615 |
|  | Severe | 0.000108 | 0.000896 |

The weighted average of the estimated fatal fall rates for the whole community-dwelling population aged 60+ was 0.00029. The PHE model incorporated an estimate from HES that 2.39% of hospitalised falls were fatal [28]; it also estimated that 5.6% of falls required hospitalisation. This meant that 0.13% of all falls were fatal. If it is assumed that 30% of the population aged 60+ experience a fall each year (the PHE model assumed that 34% of the population aged 65+ fell at least once per year and that the average falls per person was 1.06), then the fatal fall rate for this population would be 0.00039. Hence, this model underestimates the rate of fatal falls relative to the PHE model, though both rates are likely too low to substantially affect the evaluation results.

### Risk of other-cause mortality

The factor of one minus GBD (age group- and sex-specific) percentages were applied to the all-cause mortality rates to obtain the other-cause rates by age, sex, and frailty category. These rates are displayed in Figure B1 (the precise rates are not reported here and are available upon request).

**Figure B1** Annual other-cause mortality rate (range 0-1) by age, sex and frailty category.

### Costs in final year of life

Table B36 shows the costs in the final year of life by care sector and primary cause of death. The data source was the 2019 PSSRU unit cost depository which reported the final-year costs for falls patients and all patients [55]. Assuming that falls accounted for 0.82% of all deaths as estimated from the GBD data [69], weighted fall-related costs were subtracted from the all-cause costs to obtain the other-cause costs. They were assigned to the deceased individuals as they exited the model.

| **Table B36** Costs in final year of life by care sector and primary cause of death. | | | |
| --- | --- | --- | --- |
| **Cause of death** | **Healthcare^1,2^** | **Social care** | **Total public sector** |
| Falls | £10,492.48 | £6,507.61 | £17,000.09 |
| Other causes^3^ | £7,666.08 | £4,226.05 | £11,892.13 |
| **Source:** 2019 PSSRU unit cost depository [55].  ^1^ All costs are expressed in 2021/22 £. Healthcare costs were inflated at 1.98% per annum according to average NHS cost inflation rate over the period 2013-19; social care costs were inflated at 2.13% per annum according to average PSS pay & price inflation rate over the same period [55].  ^2^ Secondary care only: inpatient emergency and non-emergency; outpatient; A&E  ^3^ Assumed that falls accounted for 0.029% of all deaths; weighted fall-related costs were then subtracted from all-cause costs. | | | |

## B6.2 Non-fatal falls

The risks of non-fatal falls were estimated prospectively using the ELSA data on fall incidence and type during the two-year interim between Waves 4-5, then annualised. There are five fall-related outcomes of interest which match the five types of fall history: (1) no fall; (2) single non-MA fall; (3) recurrent non-MA falls; (4) single MA fall; and (5) recurrent falls with at least one MA fall. To estimate the risk of each of these outcomes, four separate multivariate logistic regressions are estimated for: (a) risk of being any faller; (b) risk of being a recurrent faller given any fall; (c) risk of being an MA faller given single fall; and (d) risk of being an MA faller given recurrent fall. These are sufficient to generate the five fall incidence types above: e.g., yes for (a), no for (b) and yes for (c) generate type (4) single MA fall. Nevertheless, the issue of recall bias in the use of ELSA falls data is first discussed.

### Recall bias in the ELSA falls data

A key methodological issue in prospective analyses of falls incidence between ELSA Waves 4-5 is the quality of the falls data collected at Wave 5. Whilst the Wave 4 survey asked the respondent ‘Whether fallen down in the last year (for any reason)?’, the Wave 5 survey asked ‘[Since we last talked to you on [date of last interview], have/Have] you fallen down [BLANK/in the last two years] for any reason?’. Despite the different recall periods, the two-year prevalence rates of falls of different types reported in Wave 5 were comparable to the annual rates reported in Wave 4. These are shown in Table B37.

| **Table B37** Comparison of falls risk between: (1) ELSA falls incidence between Waves 4-5; (2) ELSA falls prevalence/history in Wave 4; and (3) estimates in literature. | | | |
| --- | --- | --- | --- |
| **Fall type: target population** | **(1) Two-year [annualised] risk from ELSA Waves 4-5** | **(2) Annual risk from ELSA Wave 4** | **(3) Annual risk in literature [reference]** |
| Any fall: aged 65+ | 29.7% [16.2%] | 27.0% | NICE: 30% [8] |
| Any fall: aged 80+ | 39.1% [22.0%] | 35.5% | NICE: 50% [8] |
| Recurrent fall given any fall: aged 60+ | 43.2% [24.6%] | 43.9% |  |
| Any fall: women aged 65+ with 3+ falls in past year | 67.6% [43.1%] |  | RCT: 88.9% [19]^1^ |
| Any fall: mean age 78 with MA fall history | 47.8% [27.8%] |  | RCT: 52.0% [13]^1,2^; 68.5% [14]^1,2^ |
| **Abbreviation:** ELSA: English Longitudinal Study of Ageing; MA fall: fall requiring medical attention; NICE: National Institute for Health and Care Excellence; RCT: randomised controlled trial  ^1^ One-year falls risk is taken from the control group that received no falls prevention intervention.  ^2^ Presented to A&E for an MA fall at trial baseline. | | | |

According to Wave 5 data, 29.7% of the cohort aged 65+ (at Wave 4) and followed up in Wave 5 reported the experience of at least one fall. This two-year risk was only marginally greater than the prevalence of 27.0% for falls experienced one year prior to Wave 4. The 29.7% two-year risk was equivalent to an annualised risk of 16.2%. This is substantially lower than the Wave 4 annual prevalence and the 30% annual risk cited by the NICE guideline as generalisable to the UK population aged 65+ [8]. A noticeable pattern is that the original two-year risks from Waves 4-5 are comparable to the annual prevalence from Wave 4. The last column also gives the annual risks reported for the control groups of three UK-based RCTs that used high-frequency prospective falls recording methods as recommended for falls epidemiological research [10, 71]. A noticeable pattern is that the two-year risks from ELSA Waves 4-5 are all lower than the *annual* RCT risks.

A plausible explanation for the observed patterns is recall bias in ELSA falls measurement. It may be that despite the specific wording of the fall-related survey questions in Wave 5, the older respondents are recalling the falls that occurred over the past 12 months or so rather than 24 months, which explains the close correspondence to annual risks from Wave 4. Such recall bias is a prominent methodological issue in falls research [53, 71]. A systematic review of studies on fall-related recall bias found that the sensitivity of retrospective recording at quarterly or longer intervals can be as low as 31% relative to prospective weekly recording [72]. In the UK setting, a recent RCT found that the four-month recall method reported on average 32% fewer falls than prospective monthly recording [73]. Given the above features of ELSA falls data and the likelihood of recall bias, it was deemed reasonable to treat the Waves 4-5 data as *annual* rather than two-year risks. This meant *not* annualising the Waves 4-5 risks and assigning them to the modelled individuals. Even so, a key caveat is that two years, rather than one, have elapsed since Wave 4 which would overestimate the risk transition over time.

### Risk of any fall

As mentioned, 27.8% of the ELSA Wave 4 cohort reported the incidence of at least one fall of any type at Wave 5 follow-up. Table B38 shows the results of the logistic regression for incidence of any fall. A separate table showing all model-fit comparisons (using AIC/BIC) for falls risk estimations is not reported here but is available upon request. Older individuals and women were likelier to experience any fall. Any falls history prior to Wave 4 was associated with increased risk. Recurrent non-MA falls history had a higher coefficient than other fall types. Fear of falling was associated with higher risk.

| **Table B38** Logistic regression coefficients for any fall incidence between ELSA Waves 4 and 5. | | |
| --- | --- | --- |
| ***Dependent variable: Incidence of any fall (N=6,205)^1^*** | | |
| **Explanatory variables** | **Coefficient (SE)** | **P-value** |
| Constant | -2.709 (0.297) | <0.001 |
| Age | 0.009 (0.004) | 0.028 |
| Female | 0.187 (0.061) | 0.002 |
| Falls history (ref: No falls history) |  |  |
| *Single non-MA fall* | 0.845 (0.090) | <0.001 |
| *Recurrent non-MA falls* | 1.654 (0.102) | <0.001 |
| *Single MA fall* | 0.657 (0.141) | <0.001 |
| *Recurrent falls with MA* | 0.974 (0.166) | <0.001 |
| Frailty (0-100) | 0.049 (0.010) | <0.001 |
| Frailty^2 | -0.0007 (0.0002) | 0.002 |
| Fear of falling | 0.279 (0.125) | 0.026 |
| Abnormal gait/balance | -0.148 (0.084) | 0.079 |
| **Abbreviation:** ELSA: English Longitudinal Study of Ageing; MA fall: fall requiring medical attention; Ref: reference; SE: standard error  ^1^ Sample restricted to those interviewed in both ELSA Waves 4 and 5. | | |

### Risk of recurrent fall given any fall

Of those who experienced any fall, 43.2% experienced recurrent falls. Table B39 shows the results of logistic regression for incidence of recurrent falls given any fall. Interestingly, older fallers were less likely to be recurrent fallers. Falls history was associated with recurrent falls risk, though the coefficient for single MA fall was not significantly different from zero. Higher frailty score was associated with greater risk at a decreasing rate. Abnormal gait/balance was associated with greater risk.

| **Table B39** Logistic regression coefficients for incidence of recurrent falls given any fall between ELSA Waves 4 and 5. | | |
| --- | --- | --- |
| ***Dependent variable: Incidence of recurrent falls given any fall (N=1,731)^1^*** | | |
| **Explanatory variables** | **Coefficient (SE)** | **P-value** |
| Constant | -0.692 (0.494) | 0.161 |
| Age | -0.015 (0.007) | 0.037 |
| Falls history (ref: No falls history) |  |  |
| *Single non-MA fall* | 0.322 (0.151) | 0.033 |
| *Recurrent non-MA falls* | 1.678 (0.151) | <0.001 |
| *Single MA fall* | 0.089 (0.238) | 0.708 |
| *Recurrent falls with MA* | 1.314 (0.262) | <0.001 |
| Frailty (0-100) | 0.089 (0.017) | <0.001 |
| Frailty^2 | -0.001 (0.0004) | 0.001 |
| Abnormal gait/balance | 0.319 (0.143) | 0.026 |
| **Abbreviation:** ELSA: English Longitudinal Study of Ageing; MA fall: fall requiring medical attention; Ref: reference; SE: standard error  ^1^ Sample restricted to those interviewed in both ELSA Waves 4 and 5 who experienced any fall. | | |

### Risk of MA fall given single or recurrent fall

Single and recurrent fallers likely face differing risks of experiencing at least one MA fall. Therefore, estimation of the risk of being an MA faller given any fall was conducted separately for single and recurrent fallers. Table B40 shows the regression coefficients for single fallers, while Table B41 shows those for recurrent fallers. The best-fit model for single fallers contained only age, sex, and high physical activity as covariates. Older individuals and women were likelier to experience a single MA fall. High physical activity was associated with *greater* risk, though this was not statistically significant at 95% confidence level. It may be that high-intensity physical activity, despite its health benefits, leaves older persons more susceptible to an injurious fall.

| **Table B40** Logistic regression coefficients for incidence of MA fall given single fall between ELSA Waves 4 and 5. | | |
| --- | --- | --- |
| ***Dependent variable: Incidence of MA fall given single fall (N=984)^1^*** | | |
| **Explanatory variables** | **Coefficient (SE)** | **P-value** |
| Constant | -4.636 (0.736) | <0.001 |
| Age | 0.043 (0.009) | <0.001 |
| Female | 0.340 (0.149) | 0.023 |
| High physical activity | 0.366 (0.188) | 0.052 |
| **Abbreviation:** ELSA: English Longitudinal Study of Ageing; MA fall: fall requiring medical attention; Ref: reference; SE: standard error  ^1^ Sample restricted to those interviewed in both ELSA Waves 4 and 5 who experienced a single fall. | | |

Among recurrent fallers, women were likelier to experience at least one MA fall, while the coefficient on age was not significantly different from zero at 95% confidence level. In contrast to single fallers, histories of single MA fall and recurrent falls with MA fall were associated with greater risk of MA fall. Higher frailty was also associated with greater risk at a decreasing rate.

| **Table B41** Logistic regression coefficients for incidence of MA fall given recurrent falls between ELSA Waves 4 and 5. | | |
| --- | --- | --- |
| ***Dependent variable: Incidence of MA fall given recurrent falls (N=747)^1^*** | | |
| **Explanatory variables** | **Coefficient (SE)** | **P-value** |
| Constant | -4.159 (0.811) | <0.001 |
| Age | 0.020 (0.010) | 0.063 |
| Female | 0.426 (0.180) | 0.018 |
| Falls history (ref: No falls history) |  |  |
| *Single non-MA fall* | 0.152 (0.268) | 0.570 |
| *Recurrent non-MA falls* | 0.118 (0.207) | 0.570 |
| *Single MA fall* | 0.983 (0.383) | 0.010 |
| *Recurrent falls with MA* | 0.912 (0.310) | 0.003 |
| Frailty (0-100) | 0.091 (0.028) | 0.001 |
| Frailty^2 | -0.002 (0.0006) | 0.002 |
| **Abbreviation:** ELSA: English Longitudinal Study of Ageing; MA fall: fall requiring medical attention; Ref: reference; SE: standard error  ^1^ Sample restricted to those interviewed in both ELSA Waves 4 and 5 who experienced recurrent falls. | | |

Unlike single fallers who experience an MA fall, recurrent fallers can experience multiple MA falls. However, ELSA does not report the number of MA falls. Hence, the proportion of MA fallers who experienced recurrent MA falls was obtained from literature. The falls prevention model Howland (2015) targeted persons aged 65+ in the US who were admitted to ED for MA fall [74]. The model incorporated a parameter from a primary survey that 18% of MA fallers admitted to ED are readmitted for the same cause within one year. Between ELSA Waves 4-5, 7.7% of the cohort experienced at least one MA fall. Of these, 57.7% were single MA fallers, while the other 42.3% were recurrent fallers with one or more MA falls. If 18% of all MA fallers experienced recurrent MA falls – and these individuals are necessarily found among recurrent fallers with one or more MA falls – then calculation shows that 42.6% of those in this group would have experienced recurrent MA falls. This parameter was incorporated without any adjustment since there was no information on its variation (e.g., by frailty).

In Section B5.2 where access to reactive intervention was parameterised, it was noted that the access rate under UC from ELSA Wave 4 likely represented the proportion of MA fallers who are hospitalised. Hence, this proportion stratified by frailty category – 9.6% for Fit; 23.5% for Mild; 51.7% for Moderate; and 72.7% for Severe – was incorporated in the model to estimate the proportion of (single or recurrent) MA fallers who were hospitalised. For recurrent MA fallers, this proportion was applied again to determine the nature of the second MA fall. Hence, the nature of the first MA fall was assumed not to influence the risk of the second MA fall requiring hospitalisation.

## B6.3 Acute QALY loss due to falls

It was discussed in Section B4.1 that the EQ-5D decrements associated with falls history are unlikely to capture the acute impact of falls. Therefore, this section incorporates the latter separately. Table B42 shows the point estimates for acute QALY loss obtained from literature.

| **Table B42** Acute QALY loss associated with fall types. | | |
| --- | --- | --- |
| **Fall type** | **QALY loss** | **Source reference** |
| Single non-MA fall | 0.012 | Albert (2016) [75] |
| Recurrent non-MA falls | 0.031 | Albert (2016) [75] |
| Non-hospitalized MA fall | 0.040 | Peasgood (2009) [76] |
| Hospitalized MA fall | 0.239 | Peasgood (2009) [76] |
| **Abbreviation:** MA fall: fall requiring medical attention; QALY: quality-adjusted life year | | |

Albert (2016) developed a decision tree model using individual-level data, including EQ-5D values, collected at a quasi-experimental evaluation of multifactorial intervention in the US [75]. The EQ-5D values for control and intervention groups were categorised by the number of falls experienced (none, single or multiple) and all-cause healthcare utilisation frequency over the 12-month follow-up. Control group participants who experienced a single fall and had no healthcare utilisation had an average EQ-5D value 0.024 less than control group participants who experienced no fall or healthcare utilisation. Hence, single non-MA fallers were assumed to experience a non-permanent EQ-5D decrement of 0.024 for the year in which the fall occurs, which is equivalent to a QALY loss of 0.012 if the fall is experienced mid-year. The QALY loss for recurrent non-MA falls was similarly estimated.

As noted, 28.7% of MA falls required hospitalisation. McLean (2015) reported the distribution of fall-related injuries by secondary care utilisation type (requiring vs. not requiring hospital inpatient stay) from a falls prevention RCT [67]. Of all injuries reported (hip fracture, wrist fracture, shoulder fracture, other fracture, head injury, cuts/bruises, other), wrist fracture had the highest likelihood of requiring secondary care but not hospitalisation (87.5%). Hence, it was assumed that the QALY impact incurred by wrist fracture is representative of that incurred by injuries requiring non-hospitalised secondary care. The QALY loss in the first year of wrist fracture was estimated by Peasgood and colleagues who conducted a systematic review and meta-analysis of studies measuring the health utility impact of fractures [76]; the authors estimated a QALY loss of 0.040 over 12 months. For hospitalised MA falls, McLean (2015) reported that hip fracture had the highest likelihood of requiring hospitalisation (100%) relative to other injuries [67]. Hence, the QALY impact of hip fracture was assumed to be representative of injuries requiring hospitalisation; Peasgood and colleagues estimated QALY loss of 0.239 in the first year of hip fracture [76]. For recurrent MA fallers, a simple assumption was made that the relevant QALY losses would be added: e.g., QALY loss of 0.279 was assigned to a person whose first MA fall did not require hospitalisation but the second one did.

## B6.4 Economic consequences of falls

Economic consequences directly attributable to falls can be categorised by sector and care type: healthcare – A&E, hospitalisation, ambulatory/community, and rehabilitation; social care – short-term and long-term; and societal – OOP care, informal caregiver burden, and productivity loss. These direct costs complement the comorbidity care costs parameterised in Section B4.4. Costs of LTC are parameterised separately in Section B7.2 as part of dynamic transition.

### Public sector costs

Table B43 summarises the direct healthcare costs of MA falls in the year of incidence. Data were obtained from two UK-based models, Franklin (2019) [42] and PHE model [28], that incorporated nationally representative cost estimates. For those who experienced recurrent MA falls, the resource consequences were assumed not to overlap, and their costs were added. Healthcare costs incurred after the year of incidence were incorporated as comorbidity care costs associated with higher frailty.

| **Table B43** Direct healthcare costs of falls in the year of incidence. | | | |
| --- | --- | --- | --- |
| **Fall type** | **Resource consequence** | **Cost^1^** | **Source reference** |
| MA fall – not hospitalised | 999 call | £7.77 | [42] |
|  | Ambulance | £265.96 | [42] |
|  | A&E only | £157.48 | [42] |
|  | Rehabilitation^2^ | £47.57 | [42] |
|  | **Total** | **£478.78** |  |
| MA fall – hospitalised | 999 call | £7.77 | [42] |
|  | Ambulance | £265.96 | [42] |
|  | A&E attendance before hospitalisation | £101.56 | [28] |
|  | Hospitalisation^3^ | £9,292.13 | [28] |
|  | Rehabilitation^2^ | £81.20 | [28] |
|  | Geriatric ward^4^ | £956.36 | [28] |
|  | **Total** | **£10,704.98** |  |
| **Abbreviation:** ELSA: English Longitudinal Study of Ageing; MA fall: fall requiring medical attention; SE: standard error  ^1^ All mean unit costs obtained from previous models were inflated to 2021/22 prices using the average annual inflation rate between 2013-19 (1.98%) for healthcare services as reported in PSSRU unit cost depository [55].  ^2^ Rehabilitation costs comprised of costs of GP and outpatient visits weighted by age group-specific probabilities of their need [42]. The probabilities were higher for hospitalised falls – hence the higher average. This was further weighted by proportion of target population in each age group from ELSA to obtain the final weighted average.  ^3^ Hospitalisation costs were originally disaggregated by two types of fall-related injuries that caused hospitalisation: hip fracture vs. other injuries [28]. The weighted average was obtained from the information that 31% of hospitalised falls were caused by hip fracture [28].  ^4^ Only 5.8% of hospitalised falls required long-stay geriatric ward which cost £14,659 per person in 2015/16 price [28]. In the absence of individual-level data on the risk of geriatric ward admission, its expected cost was applied to all hospitalised falls. | | | |

There were some differences in how the two models had parameterised the healthcare costs. First, Franklin (2019) included rehabilitation costs (GP and outpatient visits) for all MA falls, while the PHE model included them for hip fracture only. This model chose the first approach since the PSSRU unit cost repository showed significant care costs – mean of £1,128 excluding inpatient costs – after hospital discharge for any cause [55]. Secondly, the hospitalisation cost was substantially higher in the PHE model: mean of £9,292.13 as in Table B43 compared to £3,935.15 in Franklin (2019) (in 2021/22 price). The disparity can be attributed to the differing sources of data for the cost estimates. Franklin (2019) used the weighted average cost of orthopaedic related items recorded in NHS reference costs, while the PHE model drew on cost-of-illness studies that focused specifically on falls and hip fractures [77, 78]. This model chose the second approach because the estimates from the fall-specific cost-of-illness studies are likely more accurate than the average costs for orthopaedic conditions. Finally, the PHE model included the cost of admission to long-stay geriatric wards which was not considered in Franklin (2019). This model followed the PHE model since the ward admission was a separate resource consequence to ambulatory rehabilitation. Having estimated the healthcare costs, ELSA Wave 4 data was used to estimate the annual prevalence of MA falls by frailty category. The expected cost of MA falls by frailty category was then estimated and subtracted from the all-cause primary and secondary care costs to obtain the comorbidity care costs by frailty category in Section B4.4 (Table B17).

The direct healthcare costs estimated here did not include those of district nursing which were included as all-cause community healthcare cost in Section B4.4. The systematic review preceding parameterisation [2, 79] identified three UK-based evaluations that included district nursing as a resource consequence of falls [80-82]. Of these, two targeted specific populations of women requiring cataract surgeries [80, 81], while the third, Sach (2012) [82], targeted older persons requiring an ambulance following a fall. To estimate the district nursing cost *directly* attributable to falls from Sach (2012), the difference in the district nursing cost between intervention and control groups was examined since the intervention group experienced significantly lower number of falls. The intervention group used on average 0.36 less hours of district nursing during the 12-month study at £9.25 cost reduction per person. This amount was considerably smaller than the annual cost of district nursing estimated in Section B4.4 (£4,809.22), and the 95% confidence interval around the mean cost reduction was very wide (-£352.73 to £307.23). Hence, falls were assumed to incur community healthcare cost only via frailty progression, and no costs were subtracted from the all-cause care cost in Section B4.4.

### Societal costs

Sach (2012) also reported costs of short-term social care (home care worker, day centre visits, meals on wheels, and special equipment), OOP care (meals on wheels and home care workers), and informal care (time opportunity cost) [82]. Interestingly, the intervention group incurred on average £381.58 *more* per person for short-term social care, £132.66 *more* for OOP care and £1,303.71 *more* for informal care relative to the control group. A potential explanation is that the multifactorial intervention evaluated by the trial identified previously unrecognised care needs and induced greater care provision. In the absence of evidence, these costs were not directly attributed to falls in the model; and hence no costs were subtracted from the all-cause estimates in Section B4.4.

# B7 Dynamic transitions

Following the incidence of some type of non-fatal fall(s) (if any), individuals’ characteristics are updated for the next cycle. First, their frailty scores are updated (Section B7.1) according to the fall incidence type and current cycle characteristics. Second, the risk and incidence of LTC admission are estimated (B7.2). If the current cycle is the final one, the model concludes at this point, and all individuals exit the model with or without the LTC admission. For non-admitted individuals in non-final cycles, their characteristics are updated for the next cycle (B7.3).

## B7.1 Frailty progression

Between ELSA Waves 4-5, the frailty score (range 0-100) increased on average by 2.60 points. If the rate of change is assumed to be constant over the two-year interim, this is equivalent to an annual change of 1.30 points. Table B44 shows the coefficient estimates from the best-fit multivariate regression for (two-year) change in frailty. A separate table showing all model-fit comparisons (using AIC/BIC) for dynamic transition estimations is not reported here but is available upon request.

| **Table B44** Linear regression coefficients for two-year change in frailty (0-100) between ELSA Waves 4 and 5. | | |
| --- | --- | --- |
| ***Dependent variable: Change in frailty (0-100) (N=6,205)*** | | |
| **Explanatory variables** | **Coefficient (SE)** | **P-value** |
| Constant | -5.460 (0.696) | <0.001 |
| Age W4 | 0.134 (0.010) | <0.001 |
| SES (ref: Most privileged quartile) |  |  |
| *2^nd^ quartile* | 0.089 (0.215) | 0.680 |
| *3^rd^ quartile* | 0.011 (0.184) | 0.951 |
| *Most deprived quartile* | 0.701 (0.219) | 0.001 |
| Falls incidence W5 (ref: No fall incidence) |  |  |
| *Single non-MA fall* | 0.684 (0.227) | 0.003 |
| *Recurrent non-MA falls* | 2.329 (0.261) | <0.001 |
| *Single MA fall* | 1.648 (0.350) | <0.001 |
| *Recurrent falls with MA* | 3.870 (0.412) | <0.001 |
| Frailty W4 (0-100) | -0.198 (0.010) | <0.001 |
| High physical activity W4 | -0.730 (0.192) | <0.001 |
| Cognitive impairment W4 | 0.620 (0.187) | 0.001 |
| Social care receipt W4 | 2.643 (0.589) | <0.001 |
| Informal care receipt W4 | 1.612 (0.202) | <0.001 |
| **Abbreviation:** ELSA: English Longitudinal Study of Ageing; MA fall: fall requiring medical attention; Ref: reference; SE: standard error; SES: socioeconomic status; W4: ELSA Wave 4; W5: ELSA Wave 5 | | |

Older individuals were likelier to experience a higher increase in frailty. Those in the most deprived SES quartile were likelier to experience a higher increase relative to those in the most privileged quartile. Falls incidence of any type between Waves 4-5 had a significant impact on the frailty progression; these associations capture the secondary effects of falls. Frailer individuals at Wave 4 were less likely to experience a faster rate of frailty increase. High physical activity was associated with lower rate of frailty increase, capturing the wider health benefits of exercise that habituates physical activity [18]. Cognitive impairment was associated with higher rate. Interestingly, social and informal care receipts were associated with higher rate of increase. This likely indicates the vulnerability of care recipients that is not wholly captured by frailty.

The estimated change in frailty was halved to obtain the annualised rate. It was then used as an explanatory variable in other longitudinal estimations below. The annualised change was also added to individuals’ pre-change frailty score to obtain the score used in the next cycle.

## B7.2 Long-term care admission and cost

This section estimates the likelihood of surviving individuals entering LTC. Due to data limitations, the distinction is not made between fall-related and other-cause LTC admissions; ELSA did not specify the cause of LTC admission. Table B45 shows the coefficient estimates from the best-fit logistic regression for the risk of LTC admission between ELSA Waves 4-5. Older individuals were likelier to enter LTC. Falls incidence between Waves 4-5 was in fact excluded from the best-fit model, indicating a weak direct association between falls and LTC admission. Change in frailty was associated with admission risk; falls thus raise the admission risk indirectly via frailty progression. The cognitively impaired were likelier to require LTC. Interestingly, receipts of various forms of care in the community were associated with increased risk. Care receipts are indicators of broader vulnerability that eventually requires permanent institutionalisation.

| **Table B45** Logistic regression coefficients for long-term care admission between ELSA Waves 4 and 5. | | |
| --- | --- | --- |
| ***Dependent variable: New LTC admission between ELSA Wave 4-5 (N=6,205)*** | | |
| **Explanatory variables** | **Coefficient (SE)** | **P-value** |
| Constant | -15.281 (1.919) | <0.001 |
| Age W4 | 0.107 (0.025) | <0.001 |
| Change in frailty^1^ | 0.061 (0.020) | 0.003 |
| Cognitive impairment W4 | 1.736 (0.413) | <0.001 |
| Community healthcare receipt W4 | 2.376 (0.822) | 0.004 |
| Social care receipt W4 | 1.821 (0.408) | <0.001 |
| OOP care receipt W4 | 1.000 (0.454) | 0.028 |
| Informal care receipt W4 | 1.302 (0.446) | 0.003 |
| **Abbreviation:** ELSA: English Longitudinal Study of Ageing; LTC: long-term care; MA fall: fall requiring medical attention; OOP: out-of-pocket; Ref: reference; SE: standard error; W4: ELSA Wave 4; W5: ELSA Wave 5.  ^1^ Covariate included in logistic regression is two-year change in frailty measured in ELSA. In model simulation, annualised change is used to predict dependent variable. | | |

In applying the estimated admission risk, the estimates were not halved for annualisation in the same way that change in frailty estimates were halved. Instead, estimated coefficients were applied to a one-year increase in age and annualised change in frailty to obtain a ‘quasi-annualised’ risk of LTC admission. According to ELSA, 0.74% of the Wave 4 cohort aged 65+ were newly admitted to LTC in the two years between Waves 4-5. This is equivalent to an annualised risk of 0.37%, while the quasi-annualised risk would lie somewhere between. There are reasons to believe that the latter is a better estimate of the true LTC admission risk than the two-year risk or its annualised version. First, given the high mortality rate in LTC, the true two-year (annual) risk is likely higher than 0.74% (0.37%) since there would have been LTC admissions between Waves 4-5 that are unobservable due to participant deaths prior to Wave 5 follow-up. Secondly, it was noted in Section B3.1 that ELSA underestimates the prevalence of institutionalisation. Thirdly, according to NHS Digital, there were 365 new requests for Council-supported LTC admission in 2018/19 in Sheffield for persons aged 65+ (sheet T11) [27]; this constitutes 0.4% of the population aged 65+. As also noted in Section B3.1, publicly funded LTC admissions account for 56.4% of all LTC admissions if the public-private ratio observed for fall-related LTC admissions hold for other causes [28]. If so, new LTC admissions would account for around 0.6% of the population aged 65+: i.e., close to the quasi-annualised risk within the range 0.37-0.74%.

In costing the LTC admission, the PHE model divided the admissions into three categories [28]:

1. 43.6% of LTC admissions are into private care homes and require no full-time nursing care funded by the NHS. All residential costs are self-funded since persons in this category do not meet the condition for local authority financial support: savings/capital of £23,250 or less [58].
2. 40.8% of LTC admissions are into nursing homes with full-time nursing care funded by the NHS. Residential costs are shared by private savings and local authority. The extent of local authority funding is determined by individual means. In England, individuals with savings below £14,250 would qualify for full funding. Those with savings between £14,250 and £23,250 would quality for partial funding [58]. The PHE model used an estimate from literature that the average local authority share of the residential cost is two-thirds while the rest is borne by private expenditure [28].
3. 15.6% of LTC admissions are into residential facilities and require no full-time nursing care. Residential costs are shared by private savings and local authority as for category (2). The PHE model again assumed that the public sector incurs two-thirds of the residential cost [28].

In this model, category (1) admissions are assumed to occur only among those in the most and the second most privileged SES quartiles. According to ELSA, 52.5% of all new LTC admissions occurred among individuals in these quartiles which is a higher number of admissions than category (1) admissions alone (43.6% of all new admissions). Hence, category (2) admissions were assumed to make up the shortfall. If so, category (2) admissions account for 17.0% of all LTC admissions in the upper-half quartiles. Hence, in Table B46, 17.0% of LTC residents in the 1^st^ and 2^nd^ SES quartiles require NHS-funded nursing care under category (2) admission. For the other 83.0%, all residential care costs are self-funded and no nursing care costs are incurred.

| **Table B46** Cost and health outcomes in long-term care admission. | | | |
| --- | --- | --- | --- |
| **Social quartile** | **Publicly funded care component** | **Expected public sector (self-funded) cost^1^** | **Remaining QALY^2^** |
| Most privileged | 17.0% need NHS nursing care; 0% of residential care cost | £2,809 (£81,138) | 0.958 |
| 2^nd^ quartile | 17.0% need NHS nursing care; 0% of residential care cost | £2,809 (£81,138) | 0.958 |
| 3^rd^ quartile | 67.2% need NHS nursing care; 50% of residential care cost | £51,673 (£40,569) | 0.958 |
| Most deprived | 67.2% need NHS nursing care; 100% of residential care cost | £92,242 (£0) | 0.958 |
| **Abbreviation:** ELSA: English Longitudinal Study of Ageing; LTC: long-term care; PW: per week; QALY: quality-adjusted life year  ^1^ As in the Public Health England model, the aggregate care cost in LTC is estimated under the assumption that the average life expectancy in LTC is 2.5 years or 130 weeks [28]. The unit costs of nursing and residential care are also taken from Public Health England model: £127.11 per week for nursing care and £624.14 per week for residential care. The 2015/16 prices was updated to 2021/22 prices using the annual NHS pay and price inflation rate of 1.98% for nursing care and social care inflation rate of 2.13% for residential care [55].  ^2^ The aggregate remaining QALY in LTC is estimated under the assumption that the average life expectancy in LTC is 2.5 years. The life-years are weighted by factor 0.383 which is the median EQ-5D of institutionalised respondents in ELSA Wave 4 (see Section B4.1 for EQ-5D estimation). | | | |

According to the PHE model, the cost of nursing care was £127.11 per week (in 2021/22 price), while the average length of stay at LTC (of all categories) was 2.5 years or 130 weeks. Hence, the expected total nursing care cost for 1^st^ and 2^nd^ social quartile individuals was £2,809 per person. For this subgroup, the total self-funded residential cost is £81,138 per person. ELSA data was used to obtain the median EQ-5D estimate of 0.383 among institutionalised persons. It was assumed that this EQ-5D value applied to all individuals regardless of SES and LTC category. Hence, given the average life expectancy of 2.5 years at LTC entry, the expected remaining QALY is 0.958 for all individuals.

For individuals in the 3^rd^ and 4^th^ (‘bottom-half’) SES quartiles, their LTC admissions are assumed to be of category (2) or (3), with varying level of public support for residential care cost. As mentioned, the PHE model used the empirical finding that around two-thirds of the costs incurred by category (2) and (3) admissions are borne by local authorities. According to ELSA, of all new LTC admissions that occurred for individuals in the bottom-half quartiles, those in the 3^rd^ SES quartile accounted for around 70%, while those in the 4^th^ quartile accounted for the other 30%. If it is assumed that the local authorities on average supported 50% of the residential costs for those in the 3^rd^ quartile and 100% for those in the 4^th^ quartile, then the average level of public funding support is 65%, i.e., close to two-thirds. Based on the ELSA estimate that individuals in the bottom-half quartiles accounted for 47.5% of all new LTC admissions and on the assumption this subgroup accounted for all category (3) admissions and the remaining category (2) admissions not accounted by individuals in the upper-half quartiles, it can be calculated that 67.2% of admissions in the bottom-half quartiles are category (2), while the other 32.8% are category (3). Under these assumptions, the expected cost of LTC stay borne by the public sector is £51,673 for those in the 3^rd^ quartile and £92,242 for the 4^th^ quartile. Those in the 3^rd^ social quartile would self-fund 50% of their residential costs amounting to £40,569.

## B7.3 Updated covariates and outcomes

The model incorporates several covariates that change over time. Age and falls history change in certain patterns: age increases by one year, while falls incidence for the cycle becomes falls history for the next. Frailty progression has already been estimated above. Other covariates and outcomes which have significant ‘memories’ (i.e., where its current value strongly influences the value taken in the next cycle) should similarly be estimated prospectively using the longitudinal information contained in ELSA. For illustration, this section reports the results of prospective estimations for: (i) high physical activity; (ii) self-referred exercise demand (under RC); and (iii) change in EQ-5D. Estimations for the following covariates are reported elsewhere and are available upon request: (1) cognitive impairment; (2) abnormal gait and balance; (3) GP routine contact; (4) paid employment; (5) unpaid work status; (6) change in CASP-19; (7) out-of-pocket care receipt; and (8) informal care receipt.

It would have been feasible to include Wave 5 variables already predicted by the model as explanatory variables in subsequent equations for other Wave 5 variables. However, this would create a strong linear association between Wave 4 and Wave 5 values of the same covariate (i.e., those with significant ‘memories’) included as explanatory variables. The resulting multicollinearity may inflate the standard errors of the affected coefficients. Therefore, explanatory variables were restricted to Wave 4 values (except for Waves 4-5 fall incidence and frailty change). The two-year interim between Waves 4-5 may overestimate the dynamic change occurring for the one-year model cycle. That said, assigning the estimated coefficients to modelled individuals with *annualised* frailty change (a key explanatory variable for all prospective estimations) reduced the extent of overestimation.

### High physical activity

In ELSA, the proportion of the population engaged in high physical activity fell slightly from 17.3% in Wave 4 to 16.8% in Wave 5. Table B47 shows the coefficient estimates from the best-fit logistic regression for high physical activity in Wave 5.

| **Table B47** Logistic regression coefficients for high physical activity in ELSA Wave 5. | | |
| --- | --- | --- |
| ***Dependent variable: High physical activity in Wave 5 (N=6,205)*** | | |
| **Explanatory variables** | **Coefficient (SE)** | **P-value** |
| Constant | 2.123 (0.453) | <0.001 |
| Age W4 | -0.047 (0.007) | <0.001 |
| Female | -0.226 (0.079) | 0.004 |
| SES (ref: Most privileged quartile) |  |  |
| *2^nd^ quartile* | -0.065 (0.110) | 0.558 |
| *3^rd^ quartile* | -0.188 (0.096) | 0.051 |
| *Most deprived quartile* | -0.455 (0.130) | <0.001 |
| Frailty W4 (0-100) | -0.051 (0.008) | <0.001 |
| Change in frailty^1^ | -0.070 (0.009) | <0.001 |
| High physical activity W4 | 2.029 (0.082) | <0.001 |
| Informal care receipt W4 | -0.444 (0.142) | 0.002 |
| **Abbreviation:** ELSA: English Longitudinal Study of Ageing; Ref: reference; SE: standard error; SES: socioeconomic status; W4: ELSA Wave 4; W5: ELSA Wave 5  ^1^ Covariate included in logistic regression is two-year change in frailty measured in ELSA. In model simulation, the annualised change in frailty is used instead to predict dependent variable. | | |

Older age was associated with reduced probability of engaging in high physical activity. Women were less likely to engage in high physical activity, as were those in the most deprived SES quartile. Both Wave 4 frailty score and the change in frailty were associated with the probability. As expected, those who engaged in high physical activity in Wave 4 were likelier to be engaged in Wave 5. Informal care recipients were in vulnerable states that reduced their chance of engaging in high physical activity.

### Self-referred exercise demand

Demand for self-referred exercise increased from 8.2% in Wave 4 to 10.6% in Wave 5. This may be attributed to the higher proportion of women in Wave 5 (since the cohort is two years older) who are likelier to attend exercise sessions. Table B48 shows the coefficient estimates from the best-fit logistic regression for self-referred exercise demand.

| **Table B48** Logistic regression coefficients for self-referred exercise demand in ELSA Wave 5. | | |
| --- | --- | --- |
| ***Dependent variable: Self-referred exercise demand in Wave 5 (N=6,094)*** | | |
| **Explanatory variables** | **Coefficient (SE)** | **P-value** |
| Constant | -3.211 (0.464) | <0.001 |
| Age W4 | -0.017 (0.006) | 0.010 |
| Female | 0.627 (0.096) | <0.001 |
| Falls incidence W5 (ref: No fall incidence) |  |  |
| *Single non-MA fall* | 0.184 (0.137) | 0.179 |
| *Recurrent non-MA falls* | 0.264 (0.148) | 0.075 |
| *Single MA fall* | 0.371 (0.191) | 0.052 |
| *Recurrent falls with MA* | 0.490 (0.212) | 0.021 |
| Frailty W4 (0-100) | 0.069 (0.015) | <0.001 |
| Frailty^2 W4 | -0.0008 (0.0003) | 0.013 |
| Change in frailty^1^ | 0.040 (0.007) | <0.001 |
| High physical activity W4 | 0.590 (0.115) | <0.001 |
| Abnormal gait/balance W4 | -0.253 (0.129) | 0.050 |
| OOP care receipt W4 | 0.480 (0.208) | 0.021 |
| Exercise receipt W4 | 1.812 (0.107) | <0.001 |
| **Abbreviation:** ELSA: English Longitudinal Study of Ageing; MA fall: fall requiring medical attention; OOP: out-of-pocket; Ref: reference; SE: standard error; W4: ELSA Wave 4; W5: ELSA Wave 5  ^1^ Covariate included in logistic regression is two-year change in frailty measured in ELSA. In model simulation, the annualised change in frailty is used instead to predict dependent variable. | | |

Older age was associated with lower exercise demand. Women were likelier to demand exercise. Falls incidence types appeared to induce demand, though only the coefficient for recurrent falls with one or more MA fall was statistically significant at 95% confidence level. There was a significant nonlinear association between baseline frailty and demand, while change in frailty was also significantly associated. As in cross-sectional analysis, those already engaged in high physical activity were likelier to demand exercise sessions. Those with abnormal gait/balance were less likely to demand exercise. Those receiving OOP care were likelier to demand exercise, which may suggest that more socially privileged individuals demand self-referred exercise. Finally, those who received it in Wave 4 likelier to receive it again in Wave 5.

### Change in EQ-5D

The average change in EQ-5D between ELSA Waves 4-5 was -0.014 (95% CInt: -0.020 to -0.008). Table B49 shows the coefficient estimates from the best-fit linear regression for EQ-5D change.

| **Table B49** Linear regression coefficients for change in EQ-5D between ELSA Waves 4 and 5. | | |
| --- | --- | --- |
| ***Dependent variable: Change in EQ-5D (N=6,205)*** | | |
| **Explanatory variables** | **Coefficient (SE)** | **P-value** |
| Constant | 0.500 (0.025) | <0.001 |
| Age W4 | 0.002 (0.0003) | <0.001 |
| Female | -0.019 (0.005) | <0.001 |
| SES (ref: Most privileged quartile) |  |  |
| *2^nd^ quartile* | -0.019 (0.007) | 0.008 |
| *3^rd^ quartile* | -0.009 (0.006) | 0.162 |
| *Most deprived quartile* | -0.023 (0.007) | 0.002 |
| Falls incidence W5 (ref: No fall incidence) |  |  |
| *Single non-MA fall* | -0.013 (0.008) | 0.081 |
| *Recurrent non-MA falls* | -0.040 (0.009) | <0.001 |
| *Single MA fall* | -0.022 (0.012) | 0.056 |
| *Recurrent falls with MA* | -0.0001 (0.014) | 0.943 |
| Frailty W4 (0-100) | -0.010 (0.0004) | <0.001 |
| Change in frailty^1^ | -0.014 (0.0004) | <0.001 |
| Abnormal gait/balance W4 | -0.017 (0.007) | 0.016 |
| EQ-5D W4 | -0.739 (0.034) | <0.001 |
| EQ-5D^2 W4 | 0.136 (0.029) | <0.001 |
| **Abbreviation:** ELSA: English Longitudinal Study of Ageing; MA fall: fall requiring medical attention; Ref: reference; SE: standard error; SES: socioeconomic status; W4: ELSA Wave 4; W5: ELSA Wave 5  ^1^ Covariate included in logistic regression is two-year change in frailty measured in ELSA. In model simulation, the annualized change in frailty is used instead to predict dependent variable. | | |

As found for the cross-sectional analysis of EQ-5D level in Table B13, older age was in fact significantly associated with a *higher* change in EQ-5D. Women were likelier to experience a lower EQ-5D increase than men. Persons in the second most privileged quartile and the most deprived quartile were likelier to experience lower EQ-5D change than the most privileged. Only the incidence of recurrent non-MA falls was significantly associated with a negative EQ-5D change due to the closer association between more serious fall types and frailty progression. Both baseline frailty and rate of frailty change were associated with EQ-5D change. Abnormal gait/balance had a significant effect on EQ-5D. There was a non-linear relationship between baseline EQ-5D and EQ-5D change.

The estimated EQ-5D changes were not halved in the same way that the estimate change in frailty was halved for annualisation. Instead, estimated coefficients were applied to a one-year increase in age and annualised change in frailty to obtain a ‘quasi-annualised’ EQ-5D change. This assumes that the magnitudes of association between other covariates and EQ-5D change remain the same whether the period is annual or two-year. The change estimates were then added to individuals’ EQ-5D values to update them for the next cycle.

# B8 Model validation

According to the 2012 International Society for Pharmacoeconomics and Outcomes Research (ISPOR) guideline on model validation [83], recommended validation practices include the assessments of face, internal, external, and cross validities. This section explores three validities of the parameterised falls prevention model: face (Section B8.1); internal (B8.2); and external (B8.3). Cross validity is assessed in Discussion of the main manuscript where results are compared to those of previous models.

## B8.1 Face validity

Face validity assessment involves expert assessment of model structure/assumptions, data sources, problem formulation (e.g., model interventions and outcomes), and results [83]. The validator should be independent from the model development and decision-making. In this study, these model aspects were presented to three independent health economic expert modellers. The first author gave an hour-long presentation on the evaluation framework, model overview, key parameterisation methods, and preliminary results. Model parameterisation methods were subsequently reviewed by the expert modellers and both oral and written feedback was received.

The two most substantive issues raised by modellers were: (i) whether annually incoming cohorts are necessary; and (ii) whether more locally sourced and individual-level data are available for all-cause healthcare costs. Written responses were given to the issues before the face validity was confirmed. First, incoming cohorts are necessary to accurately characterise the resource and cost implications for decision-makers overseeing a geographically defined jurisdiction (rather than a specific cohort), which is the case for all local health economies. Second, it was acknowledged that there may be nontrivial variation in parameters between national and local settings. But the first author explained how the use of ELSA had been discussed and approved by stakeholders.

## B8.2 Internal validity

Internal validity assessment concerns the accuracy of coding and its consistency with the model’s specifications [83]. For transparency, this study kept the complete documentation of the Simul8 Visual Logic and features alongside written explanations of their purpose (not reported here but available upon request). The assessments were independently conducted by another researcher proficient in Simul8 coding. First, each line of model code was verified. Second, coding outputs generated onto an Excel spreadsheet were verified: e.g., prevalence rates of baseline covariates including cognitive impairment should match the rates reported in ELSA. Third, simulation stop commands were placed at activity entry points to ensure that only eligible simulated entities entered. Finally, several scenario analyses verified whether model outcomes varied in a credible fashion in response to parameter changes: e.g., impact of increasing the baseline prevalence of cognitive impairment on other covariates, comorbidity care costs, EQ-5D, productivity, and GP contact. The model’s internal validity was then confirmed.

## B8.3 External validity

External validity assessment involves the comparison of simulated model outcomes to real-world data [83]. Four outcomes were used for validation: (1) annual falls incidence after 20 model cycles; (2) annual all-cause healthcare cost after 20 model cycles; (3) total fall-related healthcare cost; and (4) life expectancy at age 65. The outcomes were chosen because they are central to the final evaluation outcomes and because comparable external data exist (e.g., UK-based RCTs for falls incidence). For (1)-(2), 20 model cycles were incorporated to allow for long-term dynamic progressions of variables within the model; in the case of less credible dynamic trajectories, the deviations between model and external data would increase with the number of cycles. Twenty rather than 40 cycles also created a balanced mix between initial and incoming cohorts.

Concerning (1), the second column of Table B50 describes the characteristics (falls history, cognitive status, and mean age) of control groups of several UK-based RCTs. Comparable model subgroups in the 20^th^ cycle of UC were then identified. The model closely tracked the RCT incidence rates or underpredicted them which would generate a conservative cost-effectiveness estimate of RC versus UC.

| **Table B50** Comparison of falls incidence rates between model simulation and UK falls prevention randomised controlled trial data. | | | | | |
| --- | --- | --- | --- | --- | --- |
| **#** | **RCT control group characteristics** | **Reference** | **Model subgroup under usual care** | **Falls incidence** | |
|  |  |  |  | **RCT^1^** | **Model** |
| 1 | MA falls history; cognitively intact; mean age 78 | [13] | Cohort 2 in 20^th^ model cycle with MA falls history; cognitively intact; mean age 78 | 52.0% | 55.1% |
| 2 | Recurrent non-MA or MA falls history; cognitively intact; mean age 73 | [19] | Cohort 7 in 20^th^ model cycle with recurrent non-MA or MA falls history; cognitively intact; mean age 73 | 85.1% | 53.4% |
| 3 | Non-MA falls history; cognitively intact; mean age 83 | [17] | Cohort 1 in 20^th^ model cycle with non-MA falls history; cognitively intact; mean age 83 | 82.4% | 67.1% |
| 4 | All falls histories; cognitively intact; mean age 78 | [16] | Cohort 2 in 20^th^ model cycle; cognitively intact; mean age 78 | 52.9% | 40.8% |
| 5 | No recurrent non-MA or MA falls history; cognitively intact; mean age 73 | [18] | Cohort 7 in 20^th^ model cycle without recurrent non-MA or MA falls history; cognitively intact; mean age 73 | 31.2% | 34.2% |
| 6 | MA falls history; cognitively impaired; mean age 84 | [15] | Cohort 1 in 20^th^ model cycle with MA falls history; cognitively impaired; mean age 85 | 79.9% | 60.5% |
| 7 | All falls histories; cognitively impaired; mean age 78 | [23] | Cohort 2 in 20^th^ model cycle; cognitively impaired; mean age 78 | 47.0% | 42.0% |
| **Abbreviation:** MA fall: fall requiring medical attention; RCT: randomised controlled trial  ^1^ Falls data for the control group were recorded prospectively at high frequency (e.g., weekly). | | | | | |

Concerning (2), Hazra and colleagues reported the average annual all-cause cost of primary and secondary healthcare in the UK for persons aged 80+ [84]. Inflated to 2021/22 prices, the costs were £4,115 per person for ages 80-84, £4,609 for age 85-89 and £4,475 for ages 90+ years. The corresponding model predictions for the 20^th^ cycle (fall-related and comorbidity primary and secondary healthcare cost without discounting) were £4,137, £4,390 and £4,977. Hence, the model underpredicted the healthcare costs for those aged 85-89 and overpredicted them by around 10% for those aged 90+. The total annual healthcare cost (for primary, secondary, community and NHS-funded nursing home care) for the model population aged 60+ in the 20^th^ cycle was around £431 million, which is around half of the £800 million annual budget of Sheffield CCG. This figure, whilst plausible, is hard to verify since the CCG spending decisions are not delineated by age groups.

Concerning (3), the PHE model had used HES data to estimate the primary and secondary healthcare costs of falls which were deemed representative of UK local health economies by the model’s Steering Group [28]. For Sheffield, the PHE model predicted a two-year fall-related primary and secondary healthcare cost of £7.9 million for 6,300 persons aged 65+ under usual care. The latter were a subgroup (20%) of fallers who would take up the prescribed exercise if offered; the fallers in turn comprised 34% of the Sheffield population aged 65+. Halving the £7.9 million to obtain the one-year cost and using the ELSA prevalence of falls among those aged 60-64, the estimated annual cost for Sheffield population aged 60+ was around £23.1 million. In comparison, the current model predicted total primary and secondary healthcare cost of £20.3 million in the first model cycle when the model population size is similar to that in the PHE model. Over the 40-year horizon under UC, the total discounted fall-related healthcare cost amounted to £658.5 million out of £10,058 million all-cause public sector cost, or 6.5%. It is difficult to verify this proportion, but it can be noted that according to GBD 2019, falls contributed 6.23% of total years lived with disability in England in 2019 for those aged 70+ [69]. If healthcare costs are proportional to the disability burden, the proportion of 6.5% is a high yet plausible figure.

Concerning (4), the average life expectancy for those aged 65 at model baseline was 16.8 years for men and 18.7 for women (including the average 2.5 years spent in LTC for those admitted). By comparison, the ONS estimates using 2017-19 data were 18.8 years for men and 21.1 for women [85]. Hence, the model underpredicts the life expectancy by around two years. This could be partly attributed to the use of ELSA Wave 4 (year 2008) data; between 2008 and 2019 the life expectancy from age 65 improved by around one year for men and women [85]. Nevertheless, the discrepancy motivates the analysis of an alternative scenario involving mortality risks to assess their impact on outcomes.

# B9 Model analysis methods

## B9.1 Parameter distributions for probabilistic sensitivity analysis

For the multivariate linear and logistic regressions, estimates were randomly sampled from the joint Normal distribution that had the point estimates reported in their respective tables as mean values and the variance-covariance matrices as measures of variability (not reported here, available upon request).

For the remaining parameters, Table B51 shows their distributional assumptions. Parameters constrained to be positive were assigned Gamma distributions, while those constrained to lie between zero and one were assigned Beta distributions [86]. Intervention efficacy RRs were assigned lognormal distributions [87]. The standard error (SE) expressed the extent of sampling-related uncertainty around a point estimate. Hence, the relative magnitude of SE as a proportion of the mean/point estimate was obtained from previously reviewed models [2]. For example, Sach (2007) reported the standard deviation of the annual cost of district nursing (i.e., community healthcare), from which the SE could be derived [80]; this SE was 8.2% of the mean cost of district nursing. The SE/mean proportion of 8.2% corresponded to the shape parameter *k* of value 148.7 for the Gamma distribution. As far as possible, such literature-based information was used to estimate the shape parameters of Gamma and Beta distributions. SEs for efficacy RRs were obtained directly from RCTs. In several cases, the best available information were assumptions made by previous models: e.g., Franklin (2019) assumed that SE for Gamma-distributed LTC cost was 10% of the mean [42].

| **Table B51** Parameter distributions used for probabilistic sensitivity analysis. | | | | |
| --- | --- | --- | --- | --- |
| **#** | **Parameter** | **Mean** | **Distribution** | **Reference** |
| ***Values and costs*** | | | | |
| 1 | Monetary value of being in paid employment | £24,192 | Gamma (*k* =26.6; ϑ =Mean/*k*)^1,2^ | [80] |
| 2 | Monetary value of engaging in unpaid work | £5,097 | Gamma (*k* =26.6; ϑ =Mean/*k*)^2^ | [80] |
| 3 | Comorbidity primary and secondary healthcare costs by frailty category | Table B17 | Gamma (*k* =82.6; ϑ =Mean/*k*) | [80] |
| 4 | Community healthcare cost | £4,809.22 | Gamma (*k* =148.7; ϑ =Mean/*k*) | [80] |
| 5 | Short-term social care cost by frailty category and cognitive status | Table B18 | Gamma (*k* =9.6; ϑ =Mean/*k*) | [80] |
| 6 | OOP care cost by frailty and SES categories | Table B20 | Gamma (*k* =26.6; ϑ =Mean/*k*) | [80] |
| 7 | Informal care cost by single vs. multiple needs | Table B23 | Gamma (*k* =26.6; ϑ =Mean/*k*) | [80] |
| 8 | Public sector cost in final year of life by cause | Table B36 | Gamma (*k* =49.6; ϑ =Mean/*k*) | [42] |
| 9 | Acute QALY loss by fall type | Table B42 | Beta (α for SE around 17% of mean; β derived)^3,4^ | [88] |
| 10 | Healthcare cost of MA falls by type | Table B43 | Gamma (*k* =25; ϑ =Mean/*k*) | [89] |
| 11 | Long-term care cost by sector and SES | Table B46 | Public sector: Gamma (*k* =100; ϑ =Mean/*k*)  Societal: Gamma (*k* =26.6; ϑ =Mean/*k*) | [42, 80] |
| 12 | Long-term care remaining QALY | 0.958 | Gamma (*k* =50.3; ϑ =Mean/*k*) | [42] |
| ***Probabilities*** | | | | |
| 1 | Probability of accessing community healthcare by frailty and cognitive status | 0.62% - 9.05% (Section B4.4) | Beta (α for SE around 10% of mean; β derived)^3^ | Assumption |
| 2 | Probability of accessing short-term social care by frailty and cognitive status | 5.37% - 30.65% (Section B4.4) | Beta (α for SE around 10% of mean; β derived) | Assumption |
| 3 | Risk of recurrent MA fall | 42.6% | Beta (α =1.8 for SE around 11% of mean; β derived) | [42] |
| 4 | Probability of MA fall requiring hospitalisation by frailty category | 9.6% - 72.7% (Section B6.2) | Beta (α for SE around 16% of mean; β derived) | [67] |
| ***Intervention access, cost and efficacy*** | | | | |
| 1 | Probability of accessing reactive intervention under UC by frailty category | 9.58% - 72.73% (Table B25) | Beta (α for SE around 16% of mean; β derived)^3^ | [67] |
| 2 | Demand rate for reactive intervention under RC by cognitive status | 53.8%, 49.6% (Table B25) | Beta (α for SE around 10% of mean; β derived) | [90] |
| 3 | Proportion accessing bi-disciplinary (rather than multidisciplinary) reactive intervention | 75% | Beta (α =1.2 for SE around 10% of mean; β derived) | Assumption |
| 4 | Probability of accessing falls risk screening under UC by frailty category | 23.9% - 43.9% (Table B26) | Beta (α for SE around 10% of mean; β derived) | Assumption |
| 5 | Probability of accessing proactive intervention under UC by frailty category | 10.9% - 100% (Table B26) | Beta (α for SE around 10% of mean; β derived) | Assumption |
| 6 | Demand rate for proactive intervention under RC by cognitive status | 82.4%, 44.5% (Table B26) | Beta (α for SE around 10% of mean; β derived) | [90] |
| 7 | Per-participant (variable) costs of multifactorial interventions | Table B31 | Public sector: Gamma (*k* =100; ϑ =Mean/*k*)  Societal: Gamma (*k* =25; ϑ =Mean/*k*) | [42, 91] |
| 8 | Fixed cost of operating falls clinic | Table B31 | Gamma (*k* =100; ϑ =Mean/*k*) | [42, 91] |
| 9 | Per-participant (variable) costs of self-referred interventions | Table B33 | Public sector: Gamma (*k* =100; ϑ =Mean/*k*)  Societal: Gamma (*k* =25; ϑ =Mean/*k*) | [42, 91] |
| 10 | Intervention efficacies | Table B34 | Lognormal (SE as reported by RCT or derived from 95% CInt) | Table B34 |
| **Abbreviation:** CInt: confidence interval; MA fall: fall requiring medical attention; OOP: out-of-pocket; QALY: quality-adjusted life year; RC: recommended care; RCT: randomised controlled trial; SE: standard error; SES: socioeconomic status; UC: usual care  ^1^ *k* is the shape parameter, and its smaller value means greater skew for the Gamma distribution; ϑ is the scale parameter.  ^2^ No distributional information was found for productivity values. Since Sach (2007)’s informal caregiving cost was estimated using human capital approach [80], its distributional information was assumed to apply to productivity.  ^3^ Both α and β are positive shape parameters; $\beta= {(\alpha-\alpha*Mean)}/{Mean}$.  ^4^ According to Lee (2013) [88], the SE of utility decrement for MA fall was 17.1% of the mean value. Hence, α was set (and β subsequently derived) such that this SE to mean percentage is realised for all fall types. | | | | |

# References

1. Squires H, Chilcott J, Akehurst R, Burr J, Kelly MP. A framework for developing the structure of public health economic models. Value in Health. 2016;19(5):588-601.

2. Kwon J, Squires H, Franklin M, Lee Y, Young T. Economic models of community-based falls prevention: a systematic review with subsequent commissioning and methodological recommendations. BMC Health Serv Res. 2022;22. doi: 10.1186/s12913-022-07647-6.

3. Kwon J, Squires H, Franklin M, Young T. Systematic review and critical methodological appraisal of community-based falls prevention economic models. Cost Effectiveness and Resource Allocation. 2022;20. doi: <https://doi.org/10.1186/s12962-022-00367-y>.

4. Steptoe A, Breeze E, Banks J, Nazroo J. Cohort profile: the English longitudinal study of ageing. Int J Epidemiol. 2013;42(6):1640-8. doi: 10.1093/ije/dys168. PubMed PMID: 23143611; PubMed Central PMCID: PMCPMC3900867.

5. Marmot M, Oldfield Z, Clemens S, Blake M, Phelps A, Nazroo J, et al. English Longitudinal Study of Ageing: Waves 0-6, 1998-2013 [computer file]. Colchester: UK Data Archive [distributor]. 2014;SN: 5050.

6. English Longitudinal Study of Ageing. ELSA Dataset Waves 0 to 9 2019.

7. NatCen Social Research. English Longitudinal Study of Ageing (ELSA) Wave One to Wave Five: User Guide to the datasets. 2012.

8. National Institute for Health and Care Excellence. Falls in older people: assessing risk and prevention. National Institute for Health and Care Excellence. 2013;Clinical Guideline 161(nice.org.uk/guidance/cg161).

9. Ippoliti R, Allievi I, Falavigna G, Giuliano P, Montani F, Obbia P, et al. The sustainability of a community nurses programme aimed at supporting active ageing in mountain areas. The International Journal of Health Planning & Management. 2018;33(4):e1100-e11. doi: <http://dx.doi.org/10.1002/hpm.2591>. PubMed PMID: 2151287056.

10. Gillespie LD, Robertson MC, Gillespie WJ, Sherrington C, Gates S, Clemson LM, et al. Interventions for preventing falls in older people living in the community. Cochrane database of systematic reviews. 2012;(9).

11. Hopewell S, Adedire O, Copsey BJ, Boniface GJ, Sherrington C, Clemson L, et al. Multifactorial and multiple component interventions for preventing falls in older people living in the community. Cochrane database of systematic reviews. 2018;(7).

12. Sherrington C, Fairhall NJ, Wallbank GK, Tiedemann A, Michaleff ZA, Howard K, et al. Exercise for preventing falls in older people living in the community. Cochrane database of systematic reviews. 2019;(1).

13. Close J, Ellis M, Hooper R, Glucksman E, Jackson S, Swift C. Prevention of falls in the elderly trial (PROFET): a randomised controlled trial. Lancet. 1999;353(9147):93-7. doi: 10.1016/S0140-6736(98)06119-4. PubMed PMID: 10023893.

14. Davison J, Bond J, Dawson P, Steen IN, Kenny RA. Patients with recurrent falls attending Accident & Emergency benefit from multifactorial intervention—a randomised controlled trial. Age and ageing. 2005;34(2):162-8.

15. Shaw FE, Bond J, Richardson DA, Dawson P, Steen IN, McKeith IG, et al. Multifactorial intervention after a fall in older people with cognitive impairment and dementia presenting to the accident and emergency department: randomised controlled trial. BMJ: British medical journal. 2003;326(7380):73.

16. Conroy S, Kendrick D, Harwood R, Gladman J, Coupland C, Sach T, et al. A multicentre randomised controlled trial of day hospital-based falls prevention programme for a screened population of community-dwelling older people at high risk of falls. Age and Ageing. 2010;39(6):704-10.

17. Spice CL, Morotti W, George S, Dent TH, Rose J, Harris S, et al. The Winchester falls project: a randomised controlled trial of secondary prevention of falls in older people. Age and ageing. 2009;38(1):33-40.

18. Iliffe S, Kendrick D, Morris R, Masud T, Gage H, Skelton D, et al. Multicentre cluster randomised trial comparing a community group exercise programme and home-based exercise with usual care for people aged 65 years and over in primary care. Health technology assessment (Winchester, England). 2014;18(49):vii-105. doi: <https://dx.doi.org/10.3310/hta18490>.

19. Skelton D, Dinan S, Campbell M, Rutherford O. Tailored group exercise (Falls Management Exercise—FaME) reduces falls in community-dwelling older frequent fallers (an RCT). Age and ageing. 2005;34(6):636-9.

20. Stanmore EK, Mavroeidi A, de Jong LD, Skelton DA, Sutton CJ, Benedetto V, et al. The effectiveness and cost-effectiveness of strength and balance Exergames to reduce falls risk for people aged 55 years and older in UK assisted living facilities: a multi-centre, cluster randomised controlled trial. BMC medicine. 2019;17(1):1-14.

21. Clegg A, Barber S, Young J, Iliffe S, Forster A. The Home-based Older People's Exercise (HOPE) trial: a pilot randomised controlled trial of a home-based exercise intervention for older people with frailty. Age and Ageing. 2014;43(5):687-95.

22. Lamb SE, Mistry D, Alleyne S, Atherton N, Brown D, Copsey B, et al. Aerobic and strength training exercise programme for cognitive impairment in people with mild to moderate dementia: the DAPA RCT. Health technology assessment. 2018;22(28):1-202. doi: <https://dx.doi.org/10.3310/hta22280>.

23. Nyman SR, Ingram W, Sanders J, Thomas PW, Thomas S, Vassallo M, et al. Randomised controlled trial of the effect of Tai Chi on postural balance of people with dementia. Clinical interventions in aging. 2019;14:2017.

24. Office for National Statistics. Estimates of the population for the UK, England and Wales, Scotland and Northern Ireland (Release date: 24 June 2020). 2020.

25. Office for National Statistics. National Life Tables, United Kingdom, 1980-1982 to 2016-2018. 2019.

26. Office for National Statistics. 2018-based subnational principal population projections for local authorities and higher administrative areas in England. 2020.

27. NHS Digital. Adult Social Care Activity and Finance Report, England - 2018-19. 2019.

28. Public Health England. A Return on Investment Tool for the Assessment of Falls Prevention Programmes for Older People Living in the Community. Public Health England. 2018.

29. Gale CR, Westbury LD, Cooper C, Dennison EM. Risk factors for incident falls in older men and women: the English longitudinal study of ageing. BMC Geriatrics. 2018;18(1):117. doi: 10.1186/s12877-018-0806-3.

30. Clegg A, Bates C, Young J, Ryan R, Nichols L, Ann Teale E, et al. Development and validation of an electronic frailty index using routine primary care electronic health record data. Age Ageing. 2016;45(3):353-60. doi: 10.1093/ageing/afw039. PubMed PMID: 26944937; PubMed Central PMCID: PMCPMC4846793.

31. Shi J, Song X, Yu P, Tang Z, Mitnitski A, Fang X, et al. Analysis of frailty and survival from late middle age in the Beijing Longitudinal Study of Aging. BMC geriatrics. 2011;11(1):17.

32. Fang X, Shi J, Song X, Mitnitski A, Tang Z, Wang C, et al. Frailty in relation to the risk of falls, fractures, and mortality in older Chinese adults: Results from the Beijing longitudinal study of aging. The journal of nutrition, health & aging. 2012;16(10):903-7.

33. Li G, Ioannidis G, Pickard L, Kennedy C, Papaioannou A, Thabane L, et al. Frailty index of deficit accumulation and falls: data from the Global Longitudinal Study of Osteoporosis in Women (GLOW) Hamilton cohort. BMC musculoskeletal disorders. 2014;15:185. Epub 2014/06/03. doi: 10.1186/1471-2474-15-185. PubMed PMID: 24885323; PubMed Central PMCID: PMCPMC4046442.

34. Li G, Thabane L, Ioannidis G, Kennedy C, Papaioannou A, Adachi JD. Comparison between frailty index of deficit accumulation and phenotypic model to predict risk of falls: data from the global longitudinal study of osteoporosis in women (GLOW) Hamilton cohort. PloS one. 2015;10(3):e0120144. Epub 2015/03/13. doi: 10.1371/journal.pone.0120144. PubMed PMID: 25764521; PubMed Central PMCID: PMCPMC4357575.

35. Kojima G, Kendrick D, Skelton DA, Morris RW, Gawler S, Iliffe S. Frailty predicts short-term incidence of future falls among British community-dwelling older people: a prospective cohort study nested within a randomised controlled trial. BMC geriatrics. 2015;15(1):155.

36. Searle SD, Mitnitski A, Gahbauer EA, Gill TM, Rockwood K. A standard procedure for creating a frailty index. BMC geriatrics. 2008;8(1):24.

37. Fried LP, Tangen CM, Walston J, Newman AB, Hirsch C, Gottdiener J, et al. Frailty in older adults: evidence for a phenotype. J Gerontol A Biol Sci Med Sci. 2001;56(3):M146-56. PubMed PMID: 11253156.

38. Foster C, Reilly J, Jago R, Murphy M, Skelton D, Cooper A, et al. UK Chief Medical Officers' Physical Activity Guidelines. Department of Health and Social Care. 2019.

39. French DP, Olander EK, Chisholm A, Mc Sharry J. Which behaviour change techniques are most effective at increasing older adults’ self-efficacy and physical activity behaviour? A systematic review. Annals of Behavioral Medicine. 2014;48(2):225-34.

40. Bunn F, Goodman C, Burn A-M. Multimorbidity and frailty in people with dementia. Nursing Standard. 2015.

41. Fish M, Bayer A, Gallacher J, Bell T, Pickering J, Pedro S, et al. Prevalence and pattern of cognitive impairment in a community cohort of men in South Wales: methodology and findings from the Caerphilly Prospective Study. Neuroepidemiology. 2008;30(1):25-33.

42. Franklin M, Hunter RM. A modelling-based economic evaluation of primary-care-based fall-risk screening followed by fall-prevention intervention: a cohort-based Markov model stratified by older age groups. Age and ageing. 2019.

43. Janssen B, Szende A. Population norms for the EQ-5D. Self-reported population health: an international perspective based on EQ-5D: Springer; 2014. p. 19-30.

44. Ara R, Brazier JE. Using health state utility values from the general population to approximate baselines in decision analytic models when condition-specific data are not available. Value in Health. 2011;14(4):539-45.

45. Beusterien KM, Yeung J-E, Pang F, Brazier J. Development of the multi-attribute adolescent health utility measure (AHUM). Health and Quality of life outcomes. 2012;10(1):1-9.

46. Xiong X, Dalziel K, Huang L, Mulhern B, Carvalho N. PIH33 How Do Different Health Conditions IMPACT Dimensions of Pediatric Preference-Based Health-Related Quality of Life Measures? Value in Health. 2021;24:S104.

47. Kind P, Hardman G, Macran S. UK population norms for EQ-5D. 1999.

48. Office for National Statistics. Average weekly earnings in Great Britain: July 2020 2020. Available from: <https://www.ons.gov.uk/employmentandlabourmarket/peopleinwork/employmentandemployeetypes/bulletins/averageweeklyearningsingreatbritain/july2020>.

49. Krol M, Brouwer W. How to estimate productivity costs in economic evaluations. Pharmacoeconomics. 2014;32(4):335-44.

50. Low Pay Commission. Press release: National Living Wage extended to younger workers 2021. Available from: <https://www.gov.uk/government/news/national-living-wage-extended-to-younger-workers>.

51. Cook J. The socio‐economic contribution of older people in the UK. Working with Older People. 2011.

52. Rafnsson SB, Shankar A, Steptoe A. Longitudinal influences of social network characteristics on subjective well-being of older adults: findings from the ELSA study. Journal of Aging and Health. 2015;27(5):919-34.

53. Davis J, Robertson MC, Comans T, Scuffham P. Guidelines for conducting and reporting economic evaluation of fall prevention strategies. Osteoporosis international. 2011;22(9):2449-59.

54. Han L, Clegg A, Doran T, Fraser L. The impact of frailty on healthcare resource use: a longitudinal analysis using the Clinical Practice Research Datalink in England. Age and Ageing. 2019;48(5):665-71.

55. Curtis LA, Burns A. Unit costs of health and social care 2019. PSSRU K, UK, editor2019.

56. Curtis L. Unit costs of health and social care 2010. Canterbury: PSSRU, University of Kent. 2010.

57. National Health Service. Health Careers: District Nurse 2020. Available from: <https://www.healthcareers.nhs.uk/explore-roles/nursing/roles-nursing/district-nurse>.

58. Age UK. How much does care cost? 2020. Available from: <https://www.ageuk.org.uk/information-advice/care/paying-for-care/local-care-costs/>.

59. Tong T. Broadening the perspective of economic evaluation in health care - A case study in dementia care in the UK: University of Sheffield; 2017.

60. Lockwood KJ, Harding KE, Boyd JN, Taylor NF. Predischarge home visits after hip fracture: a randomized controlled trial. Clinical rehabilitation. 2019;33(4):681-92.

61. Public Health England. Falls and fracture consensus statement: Supporting commissioning for prevention. London: Public Health England. 2017.

62. Sport Industry Research Centre. Dance to Health 'Phase 1 roll-out [test and learn]' evaluation. Sheffield Hallam University. 2019.

63. Giebel C, Rodgers S, Barr B, Collins B, Akpan A, Shenton J, et al. Does Social Support Affect Older Adults’ General Practitioner Attendance Rates? Findings from the North West Coast Household Health Survey. Clinical Gerontologist. 2020:1-11.

64. Kwon J, Lee Y, Young T, Squires H, Harris J. Qualitative research to inform economic modelling: a case study in older people’s views on implementing the NICE falls prevention guideline. BMC health services research. 2021;21(1):1-19.

65. Comans T, Brauer S, Haines T. A break-even analysis of a community rehabilitation falls prevention service. Australian and New Zealand journal of public health. 2009;33(3):240-5. doi: <https://dx.doi.org/10.1111/j.1753-6405.2009.00382.x>.

66. Day L, Hoareau E, Finch C, Harrison JE, Segal L, Bolton TG, et al. Modelling the impact, cost and benefits of falls prevention measures to support policy-makers and program planners. 2009.

67. McLean K, Day L, Dalton A. Economic evaluation of a group-based exercise program for falls prevention among the older community-dwelling population. BMC Geriatr. 2015;15:33. Epub 2015/04/17. doi: 10.1186/s12877-015-0028-x. PubMed PMID: 25879871; PubMed Central PMCID: PMCPMC4404560.

68. Robertson MC, Campbell AJ, Herbison P. Statistical analysis of efficacy in falls prevention trials. The Journals of Gerontology Series A: Biological Sciences and Medical Sciences. 2005;60(4):530-4.

69. Institute for Health Metrics and Evaluation. GBD Compare Data Visualization Seattle, WA: IHME, University of Washington2020 [Accessed 2020.11.16]. Available from <http://vizhub.healthdata.org/gbd-compare>].

70. Shah SM, Carey IM, Harris T, DeWilde S, Cook DG. Mortality in older care home residents in England and Wales. Age and ageing. 2013;42(2):209-15.

71. Lamb SE, Jørstad‐Stein EC, Hauer K, Becker C, Europe PoFN, Group OC. Development of a common outcome data set for fall injury prevention trials: the Prevention of Falls Network Europe consensus. Journal of the American Geriatrics Society. 2005;53(9):1618-22.

72. Ganz DA, Higashi T, Rubenstein LZ. Monitoring falls in cohort studies of community‐dwelling older people: effect of the recall interval. Journal of the American Geriatrics Society. 2005;53(12):2190-4.

73. Griffin J, Lall R, Bruce J, Withers E, Finnegan S, Lamb SE, et al. Comparison of alternative falls data collection methods in the Prevention of Falls Injury Trial (PreFIT). Journal of clinical epidemiology. 2019;106:32-40.

74. Howland J, Shankar KN, Peterson EW, Taylor AA. Savings in acute care costs if all older adults treated for fall-related injuries completed matter of balance. Injury Epidemiology. 2015;2(1):25. doi: <http://dx.doi.org/10.1186/s40621-015-0058-z>.

75. Albert SM, Raviotta J, Lin CJ, Edelstein O, Smith KJ. Cost-effectiveness of a statewide falls prevention program in Pennsylvania: Healthy Steps for Older Adults. The American journal of managed care. 2016;22(10):638-44.

76. Peasgood T, Herrmann K, Kanis JA, Brazier JE. An updated systematic review of Health State Utility Values for osteoporosis related conditions. Osteoporosis international : a journal established as result of cooperation between the European Foundation for Osteoporosis and the National Osteoporosis Foundation of the USA. 2009;20(6):853-68. doi: 10.1007/s00198-009-0844-y. PubMed PMID: 19271098.

77. Craig J, Murray A, Mitchell S, Clark S, Saunders L, Burleigh L. The high cost to health and social care of managing falls in older adults living in the community in Scotland. Scottish medical journal. 2013;58(4):198-203.

78. Leal J, Gray A, Prieto-Alhambra D, Arden NK, Cooper C, Javaid M, et al. Impact of hip fracture on hospital care costs: a population-based study. Osteoporosis International. 2016;27(2):549-58.

79. Kwon J, Lee Y, Squires H, Franklin M, Young T. Economic evaluation of community-based falls prevention interventions for older populations: a systematic methodological overview of systematic reviews. BMC Health Serv Res. 2022;22. doi: <https://doi.org/10.1186/s12913-022-07764-2>.

80. Sach TH, Foss AJ, Gregson RM, Zaman A, Osborn F, Masud T, et al. Falls and health status in elderly women following first eye cataract surgery: an economic evaluation conducted alongside a randomised controlled trial. Br J Ophthalmol. 2007;91(12):1675-9. doi: 10.1136/bjo.2007.118687. PubMed PMID: 17585002; PubMed Central PMCID: PMCPMC2095519.

81. Sach T, Foss A, Gregson R, Zaman A, Osborn F, Masud T, et al. Second-eye cataract surgery in elderly women: a cost-utility analysis conducted alongside a randomized controlled trial. Eye. 2010;24(2):276.

82. Sach TH, Logan PA, Coupland CA, Gladman JR, Sahota O, Stoner-Hobbs V, et al. Community falls prevention for people who call an emergency ambulance after a fall: an economic evaluation alongside a randomised controlled trial. Age Ageing. 2012;41(5):635-41. doi: 10.1093/ageing/afs071. PubMed PMID: 22695789; PubMed Central PMCID: PMCPMC3424053.

83. Eddy DM, Hollingworth W, Caro JJ, Tsevat J, McDonald KM, Wong JB. Model transparency and validation: a report of the ISPOR-SMDM Modeling Good Research Practices Task Force–7. Medical Decision Making. 2012;32(5):733-43.

84. Hazra NC, Rudisill C, Gulliford MC. Determinants of health care costs in the senior elderly: age, comorbidity, impairment, or proximity to death? The European Journal of Health Economics. 2018;19(6):831-42.

85. Office for National Statistics. National life tables - life expectancy in the UK: 2017 to 2019 2020 [updated 24 September 2020]. Available from: <https://www.ons.gov.uk/peoplepopulationandcommunity/birthsdeathsandmarriages/lifeexpectancies/bulletins/nationallifetablesunitedkingdom/2017to2019>.

86. Briggs AH, Goeree R, Blackhouse G, O’Brien BJ. Probabilistic analysis of cost-effectiveness models: choosing between treatment strategies for gastroesophageal reflux disease. Medical decision making. 2002;22(4):290-308.

87. Briggs AH, Weinstein MC, Fenwick EA, Karnon J, Sculpher MJ, Paltiel AD. Model parameter estimation and uncertainty analysis: a report of the ISPOR-SMDM Modeling Good Research Practices Task Force Working Group–6. Medical decision making. 2012;32(5):722-32.

88. Lee RH, Weber T, Colon-Emeric C. Comparison of cost-effectiveness of vitamin D screening with that of universal supplementation in preventing falls in community-dwelling older adults. Journal of the American Geriatrics Society. 2013;61(5):707-14. doi: <https://dx.doi.org/10.1111/jgs.12213>.

89. Hirst A, Knight C, Hirst M, Dunlop W, Akehurst R. Tramadol and the risk of fracture in an elderly female population: a cost utility assessment with comparison to transdermal buprenorphine. The European journal of health economics : HEPAC : health economics in prevention and care. 2016;17(2):217-27. doi: <https://dx.doi.org/10.1007/s10198-015-0673-1>.

90. Deverall E, Kvizhinadze G, Pega F, Blakely T, Wilson N. Exercise programmes to prevent falls among older adults: modelling health gain, cost-utility and equity impacts. Injury prevention : journal of the International Society for Child and Adolescent Injury Prevention. 2018. doi: <https://dx.doi.org/10.1136/injuryprev-2016-042309>.

91. Boyd M, Kvizhinadze G, Kho A, Wilson G, Wilson N. Cataract surgery for falls prevention and improving vision: modelling the health gain, health system costs and cost-effectiveness in a high-income country. Injury prevention. 2020;26(4):302-9.

1. Access to the falls risk assessment and treatment was not restricted to those with MA fall history: 1.58% of those without MA fall history accessed it. However, this access was restricted to those with history of recurrent non-MA falls, with 17.63% of individuals in this group accessing it. [↑](#footnote-ref-1)
2. None of those who had no falls history or history of single non-MA fall received falls risk screening, suggesting that falls history served as a preliminary criterion for screening receipt under UC. [↑](#footnote-ref-2)
