# Supplementary material for: Economic model of community-based falls prevention: seeking methodological solutions in evaluating the efficiency and equity of UK guideline recommendations
Source: BMC Geriatr. 2023 Mar 30;23:187. doi: 10.1186/s12877-023-03916-z (PMC10061399; doi:10.1186/s12877-023-03916-z)
Supplement: Supplementary file 3 — Additional file 3: Appendix C. Model analysis results. [file 12877_2023_3916_MOESM3_ESM.docx]

# Economic model of community-based falls prevention: seeking methodological solutions in evaluating the efficiency and equity of UK guideline recommendations

**Appendix C: Model analysis results**

**Authors:**

Dr Joseph Kwon,^1*^ [joseph.kwon@phc.ox.ac.uk](mailto:joseph.kwon@phc.ox.ac.uk); ORCID 0000-0002-2860-7280

Dr Hazel Squires,^2^ [h.squires@sheffield.ac.uk](mailto:h.squires@sheffield.ac.uk); ORCID 0000-0001-8467-0471

Professor Tracey Young,^2^ [t.a.young@sheffield.ac.uk](mailto:t.a.young@sheffield.ac.uk); ORCID 0000-0002-0754-7223

^1^ Nuffield Department of Primary Care Health Sciences, University of Oxford, Radcliffe Primary Care Building, Radcliffe Observatory Quarter, Woodstock Road, Oxford, England, OX2 6GG

^2^ School of Health and Related Research, University of Sheffield, Regent Court (ScHARR), 30 Regent Street, Sheffield, England, S1 4DA

* Corresponding author

**Conflict of interest:** The authors declare that they have no competing interests.

**Funding:** Dr Joseph Kwon was supported by the Wellcome Trust [108903/B/15/Z] for PhD studentship.

**Acknowledgement:** We would like to thank Dr Matthew Franklin at the School of Health and Related Research, University of Sheffield, for kindly reviewing the manuscript and offering detailed feedback.

# Contents

[Societal cost-utility analysis 3](#_Toc117887069)

[Subgroup analysis 5](#_Toc117887070)

[Distributional cost-effectiveness analysis 14](#_Toc117887071)

[Scenario analysis 16](#_Toc117887072)

# Societal cost-utility analysis

**Figure C1** Cost-effectiveness acceptability curve for societal cost-utility analysis over 40-year horizon. **Abbreviation:** QALY: quality-adjusted life year; RC: recommended care; UC: usual care.

| **Table C1** Characteristics of intervention users by pathway in 40-year societal cost-utility analysis. | | | | |
| --- | --- | --- | --- | --- |
|  | **Person-years (%) of intervention use by pathway^1^** | | | **Total** |
|  | **Reactive** | **Proactive** | **Self-referred** |  |
| **Usual care** | | | | |
| Total | 96,588 | 60,697 | 1,885 | 159,169 |
| Annual average | 2,414.7 | 1,517.4 | 47.1 | 3,979.2 |
| Age group at use |  |  |  |  |
| *Aged 60-64* | 7,819 (8.1) | 7,019 (11.6) | 426 (22.6) | 15,264 (9.6) |
| *Aged 65-69* | 9,675 (10.0) | 10,949 (18.0) | 407 (21.6) | 21,030 (13.2) |
| *Aged 70-74* | 14,036 (14.5) | 12,451 (20.5) | 366 (19.4) | 26,853 (16.9) |
| *Aged 75-79* | 20,357 (21.1) | 13,039 (21.5) | 303 (16.1) | 33,699 (21.2) |
| *Aged 80-84* | 22,462 (23.3) | 10,638 (17.5) | 223 (11.8) | 33,324 (20.9) |
| *Aged 85-89* | 15,252 (15.8) | 5,107 (8.4) | 116 (6.2) | 20,476 (12.9) |
| *Aged 90+* | 6,986 (7.2) | 1,493 (2.5) | 45 (2.4) | 8,524 (5.4) |
| Sex |  |  |  |  |
| *Male* | 29,393 (30.4) | 24,320 (40.1) | 893 (47.4) | 54,606 (34.3) |
| *Female* | 67,194 (69.6) | 36,377 (59.9) | 992 (52.6) | 104,564 (65.7) |
| Socioeconomic status |  |  |  |  |
| *1^st^ quartile* | 30,549 (31.6) | 18,946 (31.2) | 1,885 (100.0) | 51,380 (32.3) |
| *2^nd^ quartile* | 16,774 (17.4) | 10,421 (17.2) | 0 (0.0) | 27,195 (17.1) |
| *3^rd^ quartile* | 30,900 (32.0) | 19,101 (31.5) | 0 (0.0) | 50,001 (31.4) |
| *4^th^ quartile* | 18,365 (19.0) | 12,229 (20.1) | 0 (0.0) | 30,594 (19.2) |
| Frailty category at use |  |  |  |  |
| *Fit* | 2,243 (2.3) | 1,650 (2.7) | 561 (29.7) | 4,454 (2.8) |
| *Mild* | 24,618 (25.5) | 25,084 (41.3) | 930 (49.3) | 50,632 (31.8) |
| *Moderate* | 61,655 (63.8) | 31,353 (51.7) | 377 (20.0) | 93,386 (58.7) |
| *Severe* | 8,071 (8.4) | 2,610 (4.3) | 17 (0.9) | 10,697 (6.7) |
| Physical activity status at use |  |  |  |  |
| *High* | 4,852 (5.0) | 1,133 (1.9) | 303 (16.1) | 6,287 (3.9) |
| *Low* | 91,736 (95.0) | 59,564 (98.1) | 1,583 (83.9) | 152,883 (96.1) |
| Cognitive status at use |  |  |  |  |
| *Intact* | 62,536 (64.7) | 60,697 (100.0) | 1,469 (77.9) | 124,702 (78.3) |
| *Impaired* | 34,052 (35.3) | 0 (0.0) | 416 (22.1) | 34,468 (21.7) |
| Fear of falling at use |  |  |  |  |
| *No fear* | 59,754 (61.9) | 45,926 (75.7) | 1,699 (90.1) | 107,378 (67.5) |
| *Fear* | 36,834 (38.1) | 14,771 (24.3) | 186 (9.9) | 51,791 (32.5) |
| Gait/balance at use |  |  |  |  |
| *Normal gait/balance* | 23,598 (24.4) | 23,016 (37.9) | 1,237 (65.6) | 47,851 (30.1) |
| *Abnormal gait/balance* | 72,990 (75.6) | 37,681 (62.1) | 648 (34.4) | 111,319 (69.9) |
| Falls history at use |  |  |  |  |
| *No falls history* | 0 (0.0) | 19,311 (31.8) | 1,302 (69.1) | 20,613 (13.0) |
| *Single non-MA fall* | 0 (0.0) | 5,390 (8.9) | 267 (14.2) | 5,657 (3.6) |
| *Recurrent non-MA falls* | 0 (0.0) | 30,644 (50.5) | 262 (13.9) | 30,907 (19.4) |
| *Single MA fall* | 35,739 (37.0) | 2,155 (3.6) | 27 (1.4) | 37,920 (23.8) |
| *Recurrent falls with 1+ MA fall* | 60,849 (63.0) | 3,197 (5.3) | 26 (1.4) | 64,072 (40.3) |
| **Recommended care** | | | | |
| Total | 121,581 | 1,077,117 | 511,726 | 1,710,424 |
| Annual average | 3,039.5 | 26,927.9 | 12,793.1 | 42,760.6 |
| Age group at use |  |  |  |  |
| *Aged 60-64* | 18,068 (14.9) | 167,038 (15.5) | 113,031 (22.1) | 298,137 (17.4) |
| *Aged 65-69* | 17,331 (14.3) | 188,159 (17.5) | 117,420 (22.9) | 322,910 (18.9) |
| *Aged 70-74* | 20,991 (17.3) | 224,932 (20.9) | 100,461 (19.6) | 346,384 (20.3) |
| *Aged 75-79* | 23,444 (19.3) | 226,545 (21.0) | 78,856 (15.4) | 328,845 (19.2) |
| *Aged 80-84* | 21,580 (17.7) | 163,176 (15.1) | 57,022 (11.1) | 241,777 (14.1) |
| *Aged 85-89* | 14,028 (11.5) | 80,897 (7.5) | 32,091 (6.3) | 127,015 (7.4) |
| *Aged 90+* | 6,139 (5.0) | 26,370 (2.4) | 12,846 (2.5) | 45,355 (2.7) |
| Sex |  |  |  |  |
| *Male* | 40,457 (33.3) | 494,711 (45.9) | 148,975 (29.1) | 684,143 (40.0) |
| *Female* | 81,124 (66.7) | 582,406 (54.1) | 362,750 (70.9) | 1,026,280 (60.0) |
| Socioeconomic status |  |  |  |  |
| *1^st^ quartile* | 40,806 (33.6) | 350,401 (32.5) | 179,930 (35.2) | 571,137 (33.4) |
| *2^nd^ quartile* | 21,603 (17.8) | 194,002 (18.0) | 89,779 (17.5) | 305,385 (17.9) |
| *3^rd^ quartile* | 38,845 (32.0) | 335,836 (31.2) | 158,871 (31.0) | 533,552 (31.2) |
| *4^th^ quartile* | 20,327 (16.7) | 196,877 (18.3) | 83,146 (16.2) | 300,350 (17.6) |
| Frailty category at use |  |  |  |  |
| *Fit* | 12,483 (10.3) | 81,556 (7.6) | 110,419 (21.6) | 204,458 (12.0) |
| *Mild* | 51,071 (42.0) | 620,465 (57.6) | 252,444 (49.3) | 923,979 (54.0) |
| *Moderate* | 53,567 (44.1) | 353,347 (32.8) | 140,039 (27.4) | 546,953 (32.0) |
| *Severe* | 4,460 (3.7) | 21,748 (2.0) | 8,825 (1.7) | 35,033 (2.0) |
| Physical activity status at use |  |  |  |  |
| *High* | 10,956 (9.0) | 81,015 (7.5) | 96,804 (18.9) | 188,775 (11.0) |
| *Low* | 110,625 (91.0) | 996,102 (92.5) | 414,922 (81.1) | 1,521,648 (89.0) |
| Cognitive status at use |  |  |  |  |
| *Intact* | 86,628 (71.3) | 788,485 (73.2) | 395,520 (77.3) | 1,270,632 (74.3) |
| *Impaired* | 34,953 (28.7) | 288,632 (26.8) | 116,206 (22.7) | 439,791 (25.7) |
| Fear of falling at use |  |  |  |  |
| *No fear* | 85,981 (70.7) | 908,353 (84.3) | 450,569 (88.0) | 1,444,903 (84.5) |
| *Fear* | 35,600 (29.3) | 168,764 (15.7) | 61,157 (12.0) | 265,521 (15.5) |
| Gait/balance at use |  |  |  |  |
| *Normal gait/balance* | 48,504 (39.9) | 217,454 (20.2) | 350,695 (68.5) | 616,653 (36.1) |
| *Abnormal gait/balance* | 73,077 (60.1) | 859,663 (79.8) | 161,031 (31.5) | 1,093,771 (63.9) |
| Falls history at use |  |  |  |  |
| *No falls history* | 0 (0.0) | 518,972 (48.2) | 357,812 (69.9) | 876,784 (51.3) |
| *Single non-MA fall* | 0 (0.0) | 141,193 (13.1) | 92,858 (18.1) | 234,051 (13.7) |
| *Recurrent non-MA falls* | 0 (0.0) | 360,886 (33.5) | 51,525 (10.1) | 412,411 (24.1) |
| *Single MA fall* | 62,975 (51.8) | 30,413 (2.8) | 4,591 (0.9) | 97,979 (5.7) |
| *Recurrent falls with 1+ MA fall* | 58,606 (48.2) | 25,654 (2.4) | 4,939 (1.0) | 89,199 (5.2) |
| **Abbreviation:** MA fall: fall requiring medical attention  ^1^ All outcomes were averaged across 20 model trial runs with different random number seeds. | | | | |

# Subgroup analysis

| **Table C2** Outcomes by cohort for 40-year societal cost-utility analysis. | | | |
| --- | --- | --- | --- |
|  | **Usual care** | **Recommended care** | **Incremental** |
| **Initial cohort aged 60+ at baseline (n=125,244)** | | | |
| Public sector costs^1^ |  |  |  |
| *(1) All-cause public sector costs^2^* | £4,881,117,084 | £4,821,364,087 | -£59,752,997 |
| *(2) Fall-related healthcare costs* | £351,014,119 | £296,762,792 | -£54,251,328 |
| *Public sector intervention costs^3^* | £15,844,344 | £185,765,944 | £169,921,600 |
| QALY | 864,289 | 873,590 | 9,301 |
| Societal outcomes |  |  |  |
| *Net productivity^4^* |  |  | £9,387,197 |
| *Net private expenditure^5^* |  |  | £10,053,761 |
| *Net informal caregiving cost^6^* |  |  | -£11,519,567 |
| Societal gain, QALY equivalent |  |  | 181 QALYs |
| **Societal ICER using (1)^7^** |  |  | **£11,619 per QALY gained** |
| **Societal ICER using (2)** |  |  | **£12,199 per QALY gained** |
| **Societal INMB using (1)^8^** |  |  | **£174,278,899** |
| **Societal INMB using (2)** |  |  | **£168,777,229** |
| **New entry cohorts aged 60 at model entry (n=259,948)** | | | |
| Public sector costs |  |  |  |
| *(1) All-cause public sector costs* | £5,172,976,103 | £5,105,506,496 | -£67,469,608 |
| *(2) Fall-related healthcare costs* | £307,409,758 | £256,277,725 | -£51,132,033 |
| *Public sector intervention costs* | £19,537,764 | £246,611,759 | £227,073,995 |
| QALY | 1,222,636 | 1,232,905 | 10,269 |
| Societal outcomes |  |  |  |
| *Net productivity* |  |  | £2,282,879 |
| *Net private expenditure* |  |  | £13,668,965 |
| *Net informal caregiving cost* |  |  | -£83,305,784 |
| Societal gain, QALY equivalent |  |  | 1,199 QALYs |
| **Societal ICER using (1)^9^** |  |  | **£13,918 per QALY gained** |
| **Societal ICER using (2)** |  |  | **£15,342 per QALY gained** |
| **Societal INMB using (1)** |  |  | **£184,429,962** |
| **Societal INMB using (2)** |  |  | **£168,092,387** |
| **Abbreviation:** ICER: incremental cost-effectiveness ratio; INMB: incremental net monetary benefit; LTC: long-term care; OOP: out-of-pocket; QALY: quality-adjusted life year.  ^1^ All outcomes were averaged across 20 model trial runs with different random number seeds.  ^2^ Includes costs of fall-related primary and secondary healthcare, comorbidity primary and secondary healthcare, cost of dying, community healthcare, short-term social care, all-cause long-term care.  ^3^ Fixed intervention cost is divided across subgroup by the number of falls clinic users in each subgroup.  ^4^ Includes values of paid and unpaid employment minus intervention time opportunity costs.  ^5^ Includes OOP care expenditure and privately incurred LTC cost minus intervention private co-payments.  ^6^ Includes informal caregiver burden/cost minus intervention caregiver time opportunity costs.  ^7^ The public sector ICERs are £11,845 per QALY gained using (1) and £12,437 using (2).  ^8^ INMBs are estimated using the cost-effectiveness threshold of £30,000 per QALY gained.  ^9^ The public sector ICERs are £15,542 per QALY gained using (1) and £17,133 using (2). | | | |

| **Table C3** Outcomes by age group at model entry for 40-year societal cost-utility analysis. | | | |
| --- | --- | --- | --- |
|  | **Usual care** | **Recommended care** | **Incremental** |
| **Aged 60-64 at model entry among initial cohort (n=29,077)** | | | |
| Public sector costs^1^ |  |  |  |
| *(1) All-cause public sector costs^2^* | £1,295,563,238 | £1,280,659,209 | -£14,904,028 |
| *(2) Fall-related healthcare costs* | £84,148,287 | £70,843,148 | -£13,305,139 |
| *Public sector intervention costs^3^* | £4,292,997 | £50,473,135 | £46,180,138 |
| QALY | 283,967 | 287,051 | 3,084 |
| Societal outcomes |  |  |  |
| *Net productivity^4^* |  |  | £8,379,759 |
| *Net private expenditure^5^* |  |  | £1,480,789 |
| *Net informal caregiving cost^6^* |  |  | -£14,637,398 |
| Societal gain, QALY equivalent |  |  | 359 QALYs |
| **Societal ICER using (1)^7^** |  |  | **£9,085 per QALY gained** |
| **Societal ICER using (2)** |  |  | **£9,550 per QALY gained** |
| **Societal INMB using (1)^8^** |  |  | **£72,000,074** |
| **Societal INMB using (2)** |  |  | **£70,401,185** |
| **Aged 65-69 at model entry (n=25,093)** | | | |
| Public sector costs |  |  |  |
| *(1) All-cause public sector costs* | £1,068,761,942 | £1,055,067,977 | -£13,693,965 |
| *(2) Fall-related healthcare costs* | £75,952,881 | £63,618,898 | -£12,333,983 |
| *Public sector intervention costs* | £3,594,409 | £42,346,982 | £38,752,573 |
| QALY | 211,020 | 213,419 | 2,398 |
| Societal outcomes |  |  |  |
| *Net productivity* |  |  | £756,511 |
| *Net private expenditure* |  |  | £887,608 |
| *Net informal caregiving cost* |  |  | -£4,096,261 |
| Societal gain, QALY equivalent |  |  | 66 QALYs |
| **Societal ICER using (1)^9^** |  |  | **£10,168 per QALY gained** |
| **Societal ICER using (2)** |  |  | **£10,719 per QALY gained** |
| **Societal INMB using (1)** |  |  | **£48,877,475** |
| **Societal INMB using (2)** |  |  | **£47,517,492** |
| **Aged 70-74 at model entry (n=25,289)** | | | |
| Public sector costs |  |  |  |
| *(1) All-cause public sector costs* | £1,004,599,256 | £994,070,842 | -£10,528,414 |
| *(2) Fall-related healthcare costs* | £74,591,609 | £63,415,440 | -£11,176,169 |
| *Public sector intervention costs* | £3,271,570 | £39,366,924 | £36,095,354 |
| QALY | 173,643 | 175,535 | 1,892 |
| Societal outcomes |  |  |  |
| *Net productivity* |  |  | £922,329 |
| *Net private expenditure* |  |  | £3,151,993 |
| *Net informal caregiving cost* |  |  | £3,077,672 |
| Societal gain, QALY equivalent |  |  | -88 QALYs |
| **Societal ICER using (1)^10^** |  |  | **£14,174 per QALY gained** |
| **Societal ICER using (2)** |  |  | **£13,815 per QALY gained** |
| **Societal INMB using (1)** |  |  | **£28,546,893** |
| **Societal INMB using (2)** |  |  | **£29,194,647** |
| **Aged 75-79 at model entry (n=18,775)** | | | |
| Public sector costs |  |  |  |
| *(1) All-cause public sector costs* | £688,777,958 | £677,915,271 | -£10,862,687 |
| *(2) Fall-related healthcare costs* | £53,395,279 | £44,769,997 | -£8,625,283 |
| *Public sector intervention costs* | £2,229,433 | £26,171,839 | £23,942,407 |
| QALY | 101,028 | 101,931 | 903 |
| Societal outcomes |  |  |  |
| *Net productivity* |  |  | -£617,665 |
| *Net private expenditure* |  |  | £552,947 |
| *Net informal caregiving cost* |  |  | -£603,118 |
| Societal gain, QALY equivalent |  |  | -9 QALYs |
| **Societal ICER using (1)^11^** |  |  | **£14,634 per QALY gained** |
| **Societal ICER using (2)** |  |  | **£17,137 per QALY gained** |
| **Societal INMB using (1)** |  |  | **£13,734,033** |
| **Societal INMB using (2)** |  |  | **£11,496,629** |
| **Aged 80-84 at model entry (n=14,457)** | | | |
| Public sector costs |  |  |  |
| *(1) All-cause public sector costs* | £467,008,915 | £461,024,033 | -£5,984,883 |
| *(2) Fall-related healthcare costs* | £36,351,496 | £30,876,666 | -£5,474,830 |
| *Public sector intervention costs* | £1,456,738 | £16,734,881 | £15,278,143 |
| QALY | 58,592 | 59,262 | 670 |
| Societal outcomes |  |  |  |
| *Net productivity* |  |  | £49,777 |
| *Net private expenditure* |  |  | £1,646,049 |
| *Net informal caregiving cost* |  |  | £2,893,593 |
| Societal gain, QALY equivalent |  |  | -75 QALYs |
| **Societal ICER using (1)^12^** |  |  | **£15,605 per QALY gained** |
| **Societal ICER using (2)** |  |  | **£16,462 per QALY gained** |
| **Societal INMB using (1)** |  |  | **£8,572,306** |
| **Societal INMB using (2)** |  |  | **£8,062,254** |
| **Aged 85-89 at model entry (n=8,232)** | | | |
| Public sector costs |  |  |  |
| *(1) All-cause public sector costs* | £238,515,929 | £236,140,760 | -£2,375,169 |
| *(2) Fall-related healthcare costs* | £17,908,811 | £15,561,214 | -£2,347,598 |
| *Public sector intervention costs* | £686,490 | £7,478,164 | £6,791,674 |
| QALY | 25,821 | 26,092 | 271 |
| Societal outcomes |  |  |  |
| *Net productivity* |  |  | -£66,100 |
| *Net private expenditure* |  |  | £1,209,084 |
| *Net informal caregiving cost* |  |  | £1,694,790 |
| Societal gain, QALY equivalent |  |  | -50 QALYs |
| **Societal ICER using (1)^13^** |  |  | **£19,926 per QALY gained** |
| **Societal ICER using (2)** |  |  | **£20,050 per QALY gained** |
| **Societal INMB using (1)** |  |  | **£2,233,008** |
| **Societal INMB using (2)** |  |  | **£2,205,437** |
| **Aged 90+ at model entry (n=4,567)** | | | |
| Public sector costs |  |  |  |
| *(1) All-cause public sector costs* | £117,889,846 | £116,485,995 | -£1,403,850 |
| *(2) Fall-related healthcare costs* | £8,665,755 | £7,677,430 | -£988,326 |
| *Public sector intervention costs* | £312,707 | £3,194,018 | £2,881,312 |
| QALY | 10,218 | 10,299 | 81 |
| Societal outcomes |  |  |  |
| *Net productivity* |  |  | -£37,413 |
| *Net private expenditure* |  |  | £1,125,290 |
| *Net informal caregiving cost* |  |  | £151,155 |
| Societal gain, QALY equivalent |  |  | -22 QALYs |
| **Societal ICER using (1)^14^** |  |  | **£24,726 per QALY gained** |
| **Societal ICER using (2)** |  |  | **£31,681 per QALY gained** |
| **Societal INMB using (1)** |  |  | **£315,110** |
| **Societal INMB using (2)** |  |  | **-£100,414** |
| **Abbreviation:** ICER: incremental cost-effectiveness ratio; INMB: incremental net monetary benefit; LTC: long-term care; OOP: out-of-pocket; QALY: quality-adjusted life year.  ^1^ All outcomes were averaged across 20 model trial runs with different random number seeds.  ^2^ Includes costs of fall-related primary and secondary healthcare, comorbidity primary and secondary healthcare, cost of dying, community healthcare, short-term social care, all-cause long-term care.  ^3^ Fixed intervention cost is divided across subgroup by the number of falls clinic users in each subgroup.  ^4^ Includes values of paid and unpaid employment minus intervention time opportunity costs.  ^5^ Includes OOP care expenditure and privately incurred LTC cost minus intervention private co-payments.  ^6^ Includes informal caregiver burden/cost minus intervention caregiver time opportunity costs.  ^7^ The public sector ICERs are £10,143 per QALY gained using (1) and £10,661 using (2).  ^8^ INMBs are estimated using the cost-effectiveness threshold of £30,000 per QALY gained.  ^9^ The public sector ICERs are £10,448 per QALY gained using (1) and £11,015 using (2).  ^10^ The public sector ICERs are £13,511 per QALY gained using (1) and £13,169 using (2).  ^11^ The public sector ICERs are £14,481 per QALY gained using (1) and £16,958 using (2).  ^12^ The public sector ICERs are £13,863 per QALY gained using (1) and £14,624 using (2).  ^13^ The public sector ICERs are £16,288 per QALY gained using (1) and £16,390 using (2).  ^14^ The public sector ICERs are £18,095 per QALY gained using (1) and £23,184 using (2). | | | |

| **Table C4** Outcomes by sex for 40-year societal cost-utility analysis. | | | |
| --- | --- | --- | --- |
|  | **Usual care** | **Recommended care** | **Incremental** |
| **Male (n=183,881)** | | | |
| Public sector costs^1^ |  |  |  |
| *(1) All-cause public sector costs^2^* | £4,418,365,849 | £4,389,336,842 | -£29,029,007 |
| *(2) Fall-related healthcare costs* | £195,909,838 | £170,535,909 | -£25,373,929 |
| *Public sector intervention costs^3^* | £12,962,962 | £181,930,407 | £168,967,446 |
| QALY | 1,009,664 | 1,016,957 | 7,293 |
| Societal outcomes |  |  |  |
| *Net productivity^4^* |  |  | £4,398,468 |
| *Net private expenditure^5^* |  |  | £19,427,455 |
| *Net informal caregiving cost^6^* |  |  | -£27,870,521 |
| Societal gain, QALY equivalent |  |  | 214 QALYs |
| **Societal ICER using (1)^7^** |  |  | **£18,641 per QALY gained** |
| **Societal ICER using (2)** |  |  | **£19,128 per QALY gained** |
| **Societal INMB using (1)^8^** |  |  | **£85,273,829** |
| **Societal INMB using (2)** |  |  | **£81,618,750** |
| **Female (n=201,311)** | | | |
| Public sector costs |  |  |  |
| *(1) All-cause public sector costs* | £5,635,727,338 | £5,537,533,741 | -£98,193,598 |
| *(2) Fall-related healthcare costs* | £462,514,040 | £382,504,608 | -£80,009,432 |
| *Public sector intervention costs* | £22,419,146 | £250,447,296 | £228,028,150 |
| QALY | 1,077,261 | 1,089,538 | 12,277 |
| Societal outcomes |  |  |  |
| *Net productivity* |  |  | £7,271,608 |
| *Net private expenditure* |  |  | £4,295,271 |
| *Net informal caregiving cost* |  |  | -£66,954,830 |
| Societal gain, QALY equivalent |  |  | 1,166 QALYs |
| **Societal ICER using (1)^9^** |  |  | **£9,659 per QALY gained** |
| **Societal ICER using (2)** |  |  | **£11,011 per QALY gained** |
| **Societal INMB using (1)** |  |  | **£273,439,532** |
| **Societal INMB using (2)** |  |  | **£255,255,366** |
| **Abbreviation:** ICER: incremental cost-effectiveness ratio; INMB: incremental net monetary benefit; LTC: long-term care; OOP: out-of-pocket; QALY: quality-adjusted life year.  ^1^ All outcomes were averaged across 20 model trial runs with different random number seeds.  ^2^ Includes costs of fall-related primary and secondary healthcare, comorbidity primary and secondary healthcare, cost of dying, community healthcare, short-term social care, all-cause long-term care.  ^3^ Fixed intervention cost is divided across subgroup by the number of falls clinic users in each subgroup.  ^4^ Includes values of paid and unpaid employment minus intervention time opportunity costs.  ^5^ Includes OOP care expenditure and privately incurred LTC cost minus intervention private co-payments.  ^6^ Includes informal caregiver burden/cost minus intervention caregiver time opportunity costs.  ^7^ The public sector ICERs are £19,188 per QALY gained using (1) and £19,689 using (2).  ^8^ INMBs are estimated using the cost-effectiveness threshold of £30,000 per QALY gained.  ^9^ The public sector ICERs are £10,575 per QALY gained using (1) and £12,057 using (2). | | | |

| **Table C5** Outcomes by initial frailty category for 40-year societal cost-utility analysis. | | | |
| --- | --- | --- | --- |
|  | **Usual care** | **Recommended care** | **Incremental** |
| **Fit category at model entry (n=244,231)** | | | |
| Public sector costs^1^ |  |  |  |
| *(1) All-cause public sector costs^2^* | £5,826,094,424 | £5,767,683,441 | -£58,410,983 |
| *(2) Fall-related healthcare costs* | £324,111,696 | £275,254,176 | -£48,857,520 |
| *Public sector intervention costs^3^* | £17,933,939 | £248,444,499 | £237,076,461 |
| QALY | 1,494,891 | 1,505,833 | 10,942 |
| Societal outcomes |  |  |  |
| *Net productivity^4^* |  |  | £10,227,163 |
| *Net private expenditure^5^* |  |  | £11,862,880 |
| *Net informal caregiving cost^6^* |  |  | -£65,531,860 |
| Societal gain, QALY equivalent |  |  | 1,065 QALYs |
| **Societal ICER using (1)^7^** |  |  | **£14,880 per QALY gained** |
| **Societal ICER using (2)** |  |  | **£15,676 per QALY gained** |
| **Societal INMB using (1)^8^** |  |  | **£181,542,593** |
| **Societal INMB using (2)** |  |  | **£171,989,130** |
| **Mild frailty category at model entry (n=105,693)** | | | |
| Public sector costs |  |  |  |
| *(1) All-cause public sector costs* | £3,042,658,172 | £2,996,592,951 | -£46,065,221 |
| *(2) Fall-related healthcare costs* | £229,182,020 | £190,639,806 | -£38,542,215 |
| *Public sector intervention costs* | £11,767,896 | £135,248,016 | £123,480,120 |
| QALY | 498,178 | 504,682 | 6,504 |
| Societal outcomes |  |  |  |
| *Net productivity* |  |  | £680,906 |
| *Net private expenditure* |  |  | £8,062,598 |
| *Net informal caregiving cost* |  |  | -£29,102,192 |
| Societal gain, QALY equivalent |  |  | 362 QALYs |
| **Societal ICER using (1)^9^** |  |  | **£11,275 per QALY gained** |
| **Societal ICER using (2)** |  |  | **£12,371 per QALY gained** |
| **Societal INMB using (1)** |  |  | **£128,562,351** |
| **Societal INMB using (2)** |  |  | **£121,039,345** |
| **Moderate frailty category at model entry (n=28,528)** | | | |
| Public sector costs |  |  |  |
| *(1) All-cause public sector costs* | £933,836,914 | £914,376,961 | -£19,459,953 |
| *(2) Fall-related healthcare costs* | £89,271,727 | £73,172,956 | -£16,098,771 |
| *Public sector intervention costs* | £4,581,233 | £40,433,974 | £35,852,741 |
| QALY | 83,519 | 85,336 | 1,817 |
| Societal outcomes |  |  |  |
| *Net productivity* |  |  | £594,864 |
| *Net private expenditure* |  |  | £2,616,730 |
| *Net informal caregiving cost* |  |  | -£2,095,303 |
| Societal gain, QALY equivalent |  |  | 1 QALY |
| **Societal ICER using (1)^10^** |  |  | **£9,017 per QALY gained** |
| **Societal ICER using (2)** |  |  | **£10,866 per QALY gained** |
| **Societal INMB using (1)** |  |  | **£38,147,931** |
| **Societal INMB using (2)** |  |  | **£34,786,749** |
| **Severe frailty category at model entry (n=6,740)** | | | |
| Public sector costs |  |  |  |
| *(1) All-cause public sector costs* | £251,503,678 | £248,217,230 | -£3,286,448 |
| *(2) Fall-related healthcare costs* | £15,858,434 | £13,973,579 | -£1,884,855 |
| *Public sector intervention costs* | £1,099,040 | £8,251,215 | £7,152,175 |
| QALY | 10,336 | 10,643 | 307 |
| Societal outcomes |  |  |  |
| *Net productivity* |  |  | £167,143 |
| *Net private expenditure* |  |  | £1,180,518 |
| *Net informal caregiving cost* |  |  | £1,904,005 |
| Societal gain, QALY equivalent |  |  | -49 QALYs |
| **Societal ICER using (1)^11^** |  |  | **£14,959 per QALY gained** |
| **Societal ICER using (2)** |  |  | **£20,382 per QALY gained** |
| **Societal INMB using (1)** |  |  | **£3,887,083** |
| **Societal INMB using (2)** |  |  | **£2,485,490** |
| **Abbreviation:** ICER: incremental cost-effectiveness ratio; INMB: incremental net monetary benefit; LTC: long-term care; OOP: out-of-pocket; QALY: quality-adjusted life year.  ^1^ All outcomes were averaged across 20 model trial runs with different random number seeds.  ^2^ Includes costs of fall-related primary and secondary healthcare, comorbidity primary and secondary healthcare, cost of dying, community healthcare, short-term social care, all-cause long-term care.  ^3^ Fixed intervention cost is divided across subgroup by the number of falls clinic users in each subgroup.  ^4^ Includes values of paid and unpaid employment minus intervention time opportunity costs.  ^5^ Includes OOP care expenditure and privately incurred LTC cost minus intervention private co-payments.  ^6^ Includes informal caregiver burden/cost minus intervention caregiver time opportunity costs.  ^7^ The public sector ICERs are £16,328 per QALY gained using (1) and £17,202 using (2).  ^8^ INMBs are estimated using the cost-effectiveness threshold of £30,000 per QALY gained.  ^9^ The public sector ICERs are £11,903 per QALY gained using (1) and £13,060 using (2).  ^10^ The public sector ICERs are £9,023 per QALY gained using (1) and £10,873 using (2).  ^11^ The public sector ICERs are £12,590 per QALY gained using (1) and £17,155 using (2). | | | |

| **Table C6** Outcomes by initial physical activity status for 40-year societal cost-utility analysis. | | | |
| --- | --- | --- | --- |
|  | **Usual care** | **Recommended care** | **Incremental** |
| **High physical activity at model entry (n=82,460)** | | | |
| Public sector costs^1^ |  |  |  |
| *(1) All-cause public sector costs^2^* | £1,940,938,986 | £1,921,824,646 | -£19,114,340 |
| *(2) Fall-related healthcare costs* | £108,715,022 | £92,345,927 | -£16,369,095 |
| *Public sector intervention costs^3^* | £6,122,662 | £84,726,644 | £78,603,982 |
| QALY | 493,144 | 497,474 | 4,330 |
| Societal outcomes |  |  |  |
| *Net productivity^4^* |  |  | £6,381,636 |
| *Net private expenditure^5^* |  |  | £4,116,903 |
| *Net informal caregiving cost^6^* |  |  | -£21,892,919 |
| Societal gain, QALY equivalent |  |  | 403 QALYs |
| **Societal ICER using (1)^7^** |  |  | **£12,570 per QALY gained** |
| **Societal ICER using (2)** |  |  | **£13,150 per QALY gained** |
| **Societal INMB using (1)^8^** |  |  | **£82,490,684** |
| **Societal INMB using (2)** |  |  | **£79,745,439** |
| **Not high physical activity at model entry (n=302,732)** | | | |
| Public sector costs |  |  |  |
| *(1) All-cause public sector costs* | £8,113,154,201 | £8,005,045,936 | -£108,108,265 |
| *(2) Fall-related healthcare costs* | £549,708,856 | £460,694,591 | -£89,014,265 |
| *Public sector intervention costs* | £29,259,446 | £347,651,060 | £318,391,613 |
| QALY | 1,593,781 | 1,609,020 | 15,240 |
| Societal outcomes |  |  |  |
| *Net productivity* |  |  | £5,288,440 |
| *Net private expenditure* |  |  | £19,605,822 |
| *Net informal caregiving cost* |  |  | -£72,932,432 |
| Societal gain, QALY equivalent |  |  | 977 QALYs |
| **Societal ICER using (1)^9^** |  |  | **£12,967 per QALY gained** |
| **Societal ICER using (2)** |  |  | **£14,145 per QALY gained** |
| **Societal INMB using (1)** |  |  | **£276,212,177** |
| **Societal INMB using (2)** |  |  | **£257,118,177** |
| **Abbreviation:** ICER: incremental cost-effectiveness ratio; INMB: incremental net monetary benefit; LTC: long-term care; OOP: out-of-pocket; QALY: quality-adjusted life year.  ^1^ All outcomes were averaged across 20 model trial runs with different random number seeds.  ^2^ Includes costs of fall-related primary and secondary healthcare, comorbidity primary and secondary healthcare, cost of dying, community healthcare, short-term social care, all-cause long-term care.  ^3^ Fixed intervention cost is divided across subgroup by the number of falls clinic users in each subgroup.  ^4^ Includes values of paid and unpaid employment minus intervention time opportunity costs.  ^5^ Includes OOP care expenditure and privately incurred LTC cost minus intervention private co-payments.  ^6^ Includes informal caregiver burden/cost minus intervention caregiver time opportunity costs.  ^7^ The public sector ICERs are £13,739 per QALY gained using (1) and £14,373 using (2).  ^8^ INMBs are estimated using the cost-effectiveness threshold of £30,000 per QALY gained.  ^9^ The public sector ICERs are £13,798 per QALY gained using (1) and £15,051 using (2). | | | |

| **Table C7** Outcomes by initial cognitive status for 40-year societal cost-utility analysis. | | | |
| --- | --- | --- | --- |
|  | **Usual care** | **Recommended care** | **Incremental** |
| **Cognitively intact at model entry (n=325,083)** | | | |
| Public sector costs^1^ |  |  |  |
| *(1) All-cause public sector costs^2^* | £8,390,605,434 | £8,282,250,108 | -£108,355,326 |
| *(2) Fall-related healthcare costs* | £547,613,591 | £458,259,582 | -£89,354,009 |
| *Public sector intervention costs^3^* | £29,735,544 | £365,276,480 | £335,540,936 |
| QALY | 1,794,561 | 1,811,256 | 16,695 |
| Societal outcomes |  |  |  |
| *Net productivity^4^* |  |  | £10,366,724 |
| *Net private expenditure^5^* |  |  | £17,969,354 |
| *Net informal caregiving cost^6^* |  |  | -£90,183,718 |
| Societal gain, QALY equivalent |  |  | 1,376 QALYs |
| **Societal ICER using (1)^7^** |  |  | **£12,571 per QALY gained** |
| **Societal ICER using (2)** |  |  | **£13,623 per QALY gained** |
| **Societal INMB using (1)^8^** |  |  | **£314,963,934** |
| **Societal INMB using (2)** |  |  | **£295,962,617** |
| **Cognitively impaired at model entry (n=60,109)** | | | |
| Public sector costs |  |  |  |
| *(1) All-cause public sector costs* | £1,663,487,754 | £1,644,620,475 | -£18,867,279 |
| *(2) Fall-related healthcare costs* | £110,810,286 | £94,780,935 | -£16,029,351 |
| *Public sector intervention costs* | £5,646,564 | £67,101,224 | £61,454,660 |
| QALY | 292,364 | 295,238 | 2,874 |
| Societal outcomes |  |  |  |
| *Net productivity* |  |  | £1,303,352 |
| *Net private expenditure* |  |  | £5,753,371 |
| *Net informal caregiving cost* |  |  | -£4,641,633 |
| Societal gain, QALY equivalent |  |  | 3 QALYs |
| **Societal ICER using (1)^9^** |  |  | **£14,800 per QALY gained** |
| **Societal ICER using (2)** |  |  | **£15,786 per QALY gained** |
| **Societal INMB using (1)** |  |  | **£43,738,927** |
| **Societal INMB using (2)** |  |  | **£40,900,998** |
| **Abbreviation:** ICER: incremental cost-effectiveness ratio; INMB: incremental net monetary benefit; LTC: long-term care; OOP: out-of-pocket; QALY: quality-adjusted life year.  ^1^ All outcomes were averaged across 20 model trial runs with different random number seeds.  ^2^ Includes costs of fall-related primary and secondary healthcare, comorbidity primary and secondary healthcare, cost of dying, community healthcare, short-term social care, all-cause long-term care.  ^3^ Fixed intervention cost is divided across subgroup by the number of falls clinic users in each subgroup.  ^4^ Includes values of paid and unpaid employment minus intervention time opportunity costs.  ^5^ Includes OOP care expenditure and privately incurred LTC cost minus intervention private co-payments.  ^6^ Includes informal caregiver burden/cost minus intervention caregiver time opportunity costs.  ^7^ The public sector ICERs are £13,608 per QALY gained using (1) and £14,746 using (2).  ^8^ INMBs are estimated using the cost-effectiveness threshold of £30,000 per QALY gained.  ^9^ The public sector ICERs are £14,816 per QALY gained using (1) and £15,804 using (2). | | | |

| **Table C8** Outcomes by initial fear of falling status for 40-year societal cost-utility analysis. | | | |
| --- | --- | --- | --- |
|  | **Usual care** | **Recommended care** | **Incremental** |
| **No fear of falling at model entry (n=367,640)** | | | |
| Public sector costs^1^ |  |  |  |
| *(1) All-cause public sector costs^2^* | £9,494,922,194 | £9,377,123,970 | -£117,798,224 |
| *(2) Fall-related healthcare costs* | £609,664,870 | £512,519,766 | -£97,145,104 |
| *Public sector intervention costs^3^* | £32,654,964 | £408,474,667 | £375,819,703 |
| QALY | 2,025,483 | 2,043,838 | 18,355 |
| Societal outcomes |  |  |  |
| *Net productivity^4^* |  |  | £10,358,495 |
| *Net private expenditure^5^* |  |  | £22,177,045 |
| *Net informal caregiving cost^6^* |  |  | -£96,532,831 |
| Societal gain, QALY equivalent |  |  | 1,412 QALYs |
| **Societal ICER using (1)^7^** |  |  | **£13,053 per QALY gained** |
| **Societal ICER using (2)** |  |  | **£14,098 per QALY gained** |
| **Societal INMB using (1)^8^** |  |  | **£334,988,662** |
| **Societal INMB using (2)** |  |  | **£314,335,541** |
| **Fear of falling at model entry (n=17,552)** | | | |
| Public sector costs |  |  |  |
| *(1) All-cause public sector costs* | £559,170,994 | £549,746,613 | -£9,424,381 |
| *(2) Fall-related healthcare costs* | £48,759,007 | £40,520,751 | -£8,238,256 |
| *Public sector intervention costs* | £2,727,144 | £23,903,037 | £21,175,893 |
| QALY | 61,442 | 62,657 | 1,215 |
| Societal outcomes |  |  |  |
| *Net productivity* |  |  | £1,311,581 |
| *Net private expenditure* |  |  | £1,545,680 |
| *Net informal caregiving cost* |  |  | £1,707,479 |
| Societal gain, QALY equivalent |  |  | -32 QALYs |
| **Societal ICER using (1)^9^** |  |  | **£9,940 per QALY gained** |
| **Societal ICER using (2)** |  |  | **£10,943 per QALY gained** |
| **Societal INMB using (1)** |  |  | **£23,717,199** |
| **Societal INMB using (2)** |  |  | **£22,531,074** |
| **Abbreviation:** ICER: incremental cost-effectiveness ratio; INMB: incremental net monetary benefit; LTC: long-term care; OOP: out-of-pocket; QALY: quality-adjusted life year.  ^1^ All outcomes were averaged across 20 model trial runs with different random number seeds.  ^2^ Includes costs of fall-related primary and secondary healthcare, comorbidity primary and secondary healthcare, cost of dying, community healthcare, short-term social care, all-cause long-term care.  ^3^ Fixed intervention cost is divided across subgroup by the number of falls clinic users in each subgroup.  ^4^ Includes values of paid and unpaid employment minus intervention time opportunity costs.  ^5^ Includes OOP care expenditure and privately incurred LTC cost minus intervention private co-payments.  ^6^ Includes informal caregiver burden/cost minus intervention caregiver time opportunity costs.  ^7^ The public sector ICERs are £14,057 per QALY gained using (1) and £15,182 using (2).  ^8^ INMBs are estimated using the cost-effectiveness threshold of £30,000 per QALY gained.  ^9^ The public sector ICERs are £9,675 per QALY gained using (1) and £10,651 using (2). | | | |

| **Table C9** Outcomes by initial gait/balance status for 40-year societal cost-utility analysis. | | | |
| --- | --- | --- | --- |
|  | **Usual care** | **Recommended care** | **Incremental** |
| **No abnormal gait/balance at model entry (n=306,863)** | | | |
| Public sector costs^1^ |  |  |  |
| *(1) All-cause public sector costs^2^* | £7,693,078,618 | £7,605,671,025 | -£87,407,593 |
| *(2) Fall-related healthcare costs* | £473,701,595 | £399,299,108 | -£74,402,487 |
| *Public sector intervention costs^3^* | £25,592,957 | £329,422,972 | £303,830,015 |
| QALY | 1,782,506 | 1,797,759 | 15,253 |
| Societal outcomes |  |  |  |
| *Net productivity^4^* |  |  | £14,478,556 |
| *Net private expenditure^5^* |  |  | £15,082,111 |
| *Net informal caregiving cost^6^* |  |  | -£81,002,509 |
| Societal gain, QALY equivalent |  |  | 1,340 QALYs |
| **Societal ICER using (1)^7^** |  |  | **£13,043 per QALY gained** |
| **Societal ICER using (2)** |  |  | **£13,827 per QALY gained** |
| **Societal INMB using (1)^8^** |  |  | **£281,376,055** |
| **Societal INMB using (2)** |  |  | **£268,370,950** |
| **Abnormal gait/balance at model entry (n=78,329)** | | | |
| Public sector costs |  |  |  |
| *(1) All-cause public sector costs* | £2,361,014,570 | £2,321,199,558 | -£39,815,012 |
| *(2) Fall-related healthcare costs* | £184,722,282 | £153,741,409 | -£30,980,873 |
| *Public sector intervention costs* | £9,789,151 | £102,954,731 | £93,165,580 |
| QALY | 304,419 | 308,735 | 4,316 |
| Societal outcomes |  |  |  |
| *Net productivity* |  |  | -£2,808,480 |
| *Net private expenditure* |  |  | £8,640,614 |
| *Net informal caregiving cost* |  |  | -£13,822,841 |
| Societal gain, QALY equivalent |  |  | 40 QALYs |
| **Societal ICER using (1)^9^** |  |  | **£12,248 per QALY gained** |
| **Societal ICER using (2)** |  |  | **£14,276 per QALY gained** |
| **Societal INMB using (1)** |  |  | **£77,325,305** |
| **Societal INMB using (2)** |  |  | **£68,491,166** |
| **Abbreviation:** ICER: incremental cost-effectiveness ratio; INMB: incremental net monetary benefit; LTC: long-term care; OOP: out-of-pocket; QALY: quality-adjusted life year.  ^1^ All outcomes were averaged across 20 model trial runs with different random number seeds.  ^2^ Includes costs of fall-related primary and secondary healthcare, comorbidity primary and secondary healthcare, cost of dying, community healthcare, short-term social care, all-cause long-term care.  ^3^ Fixed intervention cost is divided across subgroup by the number of falls clinic users in each subgroup.  ^4^ Includes values of paid and unpaid employment minus intervention time opportunity costs.  ^5^ Includes OOP care expenditure and privately incurred LTC cost minus intervention private co-payments.  ^6^ Includes informal caregiver burden/cost minus intervention caregiver time opportunity costs.  ^7^ The public sector ICERs are £14,189 per QALY gained using (1) and £15,041 using (2).  ^8^ INMBs are estimated using the cost-effectiveness threshold of £30,000 per QALY gained.  ^9^ The public sector ICERs are £12,360 per QALY gained using (1) and £14,407 using (2). | | | |

| **Table C10** Outcomes by initial falls history type for 40-year societal cost-utility analysis. | | | |
| --- | --- | --- | --- |
|  | **Usual care** | **Recommended care** | **Incremental** |
| **No falls history (n=292,074)** | | | |
| Public sector costs^1^ |  |  |  |
| *(1) All-cause public sector costs^2^* | £7,446,456,913 | £7,362,570,982 | -£83,885,931 |
| *(2) Fall-related healthcare costs* | £460,117,890 | £388,777,031 | -£71,340,859 |
| *Public sector intervention costs^3^* | £23,388,688 | £313,012,980 | £289,624,292 |
| QALY | 1,633,681 | 1,647,721 | 14,039 |
| Societal outcomes |  |  |  |
| *Net productivity^4^* |  |  | £11,442,078 |
| *Net private expenditure^5^* |  |  | £17,130,464 |
| *Net informal caregiving cost^6^* |  |  | -£66,113,947 |
| Societal gain, QALY equivalent |  |  | 1,007 QALYs |
| **Societal ICER using (1)^7^** |  |  | **£13,674 per QALY gained** |
| **Societal ICER using (2)** |  |  | **£14,507 per QALY gained** |
| **Societal INMB using (1)^8^** |  |  | **£245,653,420** |
| **Societal INMB using (2)** |  |  | **£233,108,348** |
| **History of single non-MA fall (n=38,847)** | | | |
| Public sector costs |  |  |  |
| *(1) All-cause public sector costs* | £1,044,148,137 | £1,029,836,174 | -£14,311,963 |
| *(2) Fall-related healthcare costs* | £71,992,985 | £60,394,783 | -£11,598,202 |
| *Public sector intervention costs* | £3,552,756 | £44,696,822 | £41,144,066 |
| QALY | 207,793 | 209,751 | 1,958 |
| Societal outcomes |  |  |  |
| *Net productivity* |  |  | -£2,005,028 |
| *Net private expenditure* |  |  | £3,181,559 |
| *Net informal caregiving cost* |  |  | -£9,140,507 |
| Societal gain, QALY equivalent |  |  | 66 QALYs |
| **Societal ICER using (1)^9^** |  |  | **£13,255 per QALY gained** |
| **Societal ICER using (2)** |  |  | **£14,596 per QALY gained** |
| **Societal INMB using (1)** |  |  | **£33,895,358** |
| **Societal INMB using (2)** |  |  | **£31,181,596** |
| **History of recurrent non-MA falls (n=32,301)** | | | |
| Public sector costs |  |  |  |
| *(1) All-cause public sector costs* | £921,287,577 | £904,729,125 | -£16,558,452 |
| *(2) Fall-related healthcare costs* | £74,657,208 | £61,453,608 | -£13,203,600 |
| *Public sector intervention costs* | £4,568,548 | £43,359,843 | £38,791,295 |
| QALY | 141,123 | 143,307 | 2,184 |
| Societal outcomes |  |  |  |
| *Net productivity* |  |  | £104,467 |
| *Net private expenditure* |  |  | £1,517,201 |
| *Net informal caregiving cost* |  |  | -£10,857,070 |
| Societal gain, QALY equivalent |  |  | 157 QALYs |
| **Societal ICER using (1)^10^** |  |  | **£9,495 per QALY gained** |
| **Societal ICER using (2)** |  |  | **£10,928 per QALY gained** |
| **Societal INMB using (1)** |  |  | **£48,012,325** |
| **Societal INMB using (2)** |  |  | **£44,657,473** |
| **History of single MA fall (n=13,217)** | | | |
| Public sector costs |  |  |  |
| *(1) All-cause public sector costs* | £372,715,925 | £366,336,806 | -£6,379,118 |
| *(2) Fall-related healthcare costs* | £28,038,580 | £23,143,605 | -£4,894,976 |
| *Public sector intervention costs* | £2,090,692 | £18,453,256 | £16,362,565 |
| QALY | 67,466 | 68,173 | 707 |
| Societal outcomes |  |  |  |
| *Net productivity* |  |  | £1,258,716 |
| *Net private expenditure* |  |  | £1,106,253 |
| *Net informal caregiving cost* |  |  | -£5,812,477 |
| Societal gain, QALY equivalent |  |  | 99 QALYs |
| **Societal ICER using (1)^11^** |  |  | **£12,375 per QALY gained** |
| **Societal ICER using (2)** |  |  | **£14,214 per QALY gained** |
| **Societal INMB using (1)** |  |  | **£14,219,523** |
| **Societal INMB using (2)** |  |  | **£12,735,381** |
| **History of recurrent falls with one or more MA fall (n=8,753)** | | | |
| Public sector costs |  |  |  |
| *(1) All-cause public sector costs* | £269,484,636 | £263,397,496 | -£6,087,140 |
| *(2) Fall-related healthcare costs* | £23,617,214 | £19,271,491 | -£4,345,724 |
| *Public sector intervention costs* | £1,781,424 | £12,854,802 | £11,073,378 |
| QALY | 36,862 | 37,542 | 681 |
| Societal outcomes |  |  |  |
| *Net productivity* |  |  | £869,842 |
| *Net private expenditure* |  |  | £787,249 |
| *Net informal caregiving cost* |  |  | -£2,901,350 |
| Societal gain, QALY equivalent |  |  | 50 QALYs |
| **Societal ICER using (1)^12^** |  |  | **£6,827 per QALY gained** |
| **Societal ICER using (2)** |  |  | **£9,212 per QALY gained** |
| **Societal INMB using (1)** |  |  | **£16,923,734** |
| **Societal INMB using (2)** |  |  | **£15,182,317** |
| **Abbreviation:** ICER: incremental cost-effectiveness ratio; INMB: incremental net monetary benefit; LTC: long-term care; OOP: out-of-pocket; QALY: quality-adjusted life year.  ^1^ All outcomes were averaged across 20 model trial runs with different random number seeds.  ^2^ Includes costs of fall-related primary and secondary healthcare, comorbidity primary and secondary healthcare, cost of dying, community healthcare, short-term social care, all-cause long-term care.  ^3^ Fixed intervention cost is divided across subgroup by the number of falls clinic users in each subgroup.  ^4^ Includes values of paid and unpaid employment minus intervention time opportunity costs.  ^5^ Includes OOP care expenditure and privately incurred LTC cost minus intervention private co-payments.  ^6^ Includes informal caregiver burden/cost minus intervention caregiver time opportunity costs.  ^7^ The public sector ICERs are £14,654 per QALY gained using (1) and £15,548 using (2).  ^8^ INMBs are estimated using the cost-effectiveness threshold of £30,000 per QALY gained.  ^9^ The public sector ICERs are £13,701 per QALY gained using (1) and £15,087 using (2).  ^10^ The public sector ICERs are £10,179 per QALY gained using (1) and £11,715 using (2).  ^11^ The public sector ICERs are £14,114 per QALY gained using (1) and £16,212 using (2).  ^12^ The public sector ICERs are £7,326 per QALY gained using (1) and £9,885 using (2). | | | |

# Distributional cost-effectiveness analysis

| **Table C11** Outcomes by socioeconomic status quartile for 40-year societal cost-utility analysis. | | | |
| --- | --- | --- | --- |
|  | **Usual care** | **Recommended care** | **Incremental** |
| **1^st^ socioeconomic status quartile (n=132,844)** | | | |
| Public sector costs^1^ |  |  |  |
| *(1) All-cause public sector costs^2^* | £3,202,571,699 | £3,166,850,629 | -£35,721,070 |
| *(2) Fall-related healthcare costs* | £210,350,293 | £177,216,531 | -£33,133,761 |
| *Public sector intervention costs^3^* | £11,181,285 | £144,517,691 | £133,280,563 |
| QALY | 796,280 | 803,121 | 6,841 |
| Societal outcomes |  |  |  |
| *Net productivity^4^* |  |  | £8,010,569 |
| *Net private expenditure^5^* |  |  | -£3,236,404 |
| *Net informal caregiving cost^6^* |  |  | -£34,898,688 |
| Societal gain, QALY equivalent |  |  | 769 QALYs |
| **Societal ICER using (1)^7^** |  |  | **£12,819 per QALY gained** |
| **Societal ICER using (2)** |  |  | **£13,159 per QALY gained** |
| **Societal INMB using (1)^8^** |  |  | **£130,756,961** |
| **Societal INMB using (2)** |  |  | **£128,169,652** |
| **2^nd^ socioeconomic status quartile (n=71,780)** | | | |
| Public sector costs |  |  |  |
| *(1) All-cause public sector costs* | £1,725,402,199 | £1,705,189,086 | -£20,213,112 |
| *(2) Fall-related healthcare costs* | £117,132,131 | £98,833,621 | -£18,298,510 |
| *Public sector intervention costs* | £6,213,655 | £78,542,655 | £72,329,000 |
| QALY | 391,621 | 395,068 | 3,447 |
| Societal outcomes |  |  |  |
| *Net productivity* |  |  | £443,341 |
| *Net private expenditure* |  |  | £5,810,088 |
| *Net informal caregiving cost* |  |  | -£14,950,779 |
| Societal gain, QALY equivalent |  |  | 160 QALYs |
| **Societal ICER using (1)^9^** |  |  | **£14,450 per QALY gained** |
| **Societal ICER using (2)** |  |  | **£14,981 per QALY gained** |
| **Societal INMB using (1)** |  |  | **£52,115,888** |
| **Societal INMB using (2)** |  |  | **£54,030,490** |
| **3^rd^ socioeconomic status quartile (n=118,935)** | | | |
| Public sector costs |  |  |  |
| *(1) All-cause public sector costs* | £3,276,107,364 | £3,232,673,931 | -£43,433,433 |
| *(2) Fall-related healthcare costs* | £210,656,929 | £176,496,459 | -£34,160,470 |
| *Public sector intervention costs* | £11,226,941 | £134,960,545 | £123,733,604 |
| QALY | 631,512 | 637,465 | 5,953 |
| Societal outcomes |  |  |  |
| *Net productivity* |  |  | £3,816,622 |
| *Net private expenditure* |  |  | £11,077,206 |
| *Net informal caregiving cost* |  |  | -£32,391,864 |
| Societal gain, QALY equivalent |  |  | 419 QALYs |
| **Societal ICER using (1)^10^** |  |  | **£12,602 per QALY gained** |
| **Societal ICER using (2)** |  |  | **£14,057 per QALY gained** |
| **Societal INMB using (1)** |  |  | **£110,863,064** |
| **Societal INMB using (2)** |  |  | **£101,590,100** |
| **4^th^ socioeconomic status quartile (n=61,633)** | | | |
| Public sector costs |  |  |  |
| *(1) All-cause public sector costs* | £1,850,011,924 | £1,822,156,935 | -£27,854,989 |
| *(2) Fall-related healthcare costs* | £120,284,524 | £100,493,905 | -£19,790,619 |
| *Public sector intervention costs* | £6,764,885 | £74,417,313 | £67,652,428 |
| QALY | 267,512 | 270,840 | 3,328 |
| Societal outcomes |  |  |  |
| *Net productivity* |  |  | -£600,455 |
| *Net private expenditure* |  |  | £10,071,835 |
| *Net informal caregiving cost* |  |  | -£12,584,021 |
| Societal gain, QALY equivalent |  |  | 32 QALYs |
| **Societal ICER using (1)^11^** |  |  | **£11,844 per QALY gained** |
| **Societal ICER using (2)** |  |  | **£14,244 per QALY gained** |
| **Societal INMB using (1)** |  |  | **£61,008,301** |
| **Societal INMB using (2)** |  |  | **£52,943,931** |
| **Abbreviation:** ICER: incremental cost-effectiveness ratio; INMB: incremental net monetary benefit; LTC: long-term care; OOP: out-of-pocket; QALY: quality-adjusted life year.  ^1^ All outcomes were averaged across 20 model trial runs with different random number seeds.  ^2^ Includes costs of fall-related primary and secondary healthcare, comorbidity primary and secondary healthcare, cost of dying, community healthcare, short-term social care, all-cause long-term care.  ^3^ Fixed intervention cost is divided across subgroup by the number of falls clinic users in each subgroup.  ^4^ Includes values of paid and unpaid employment minus intervention time opportunity costs.  ^5^ Includes OOP care expenditure and privately incurred LTC cost minus intervention private co-payments.  ^6^ Includes informal caregiver burden/cost minus intervention caregiver time opportunity costs.  ^7^ The public sector ICERs are £14,260 per QALY gained using (1) and £14,638 using (2).  ^8^ INMBs are estimated using the cost-effectiveness threshold of £30,000 per QALY gained.  ^9^ The public sector ICERs are £15,120 per QALY gained using (1) and £15,676 using (2).  ^10^ The public sector ICERs are £13,488 per QALY gained using (1) and £15,046 using (2).  ^11^ The public sector ICERs are £11,957 per QALY gained using (1) and £14,380 using (2). | | | |

**Figure C2** Societal EDE INHB for SES-delineated equity analysis across parameter spectrum of: (a) relative inequality aversion; and (b) absolute inequality aversion. **Abbreviation:** EDE: equally distributed equivalent; INHB: incremental net health benefit; RC: recommended care; SES: socioeconomic status; UC: usual care.

# Scenario analysis

| **Table C12** Scenarios of 5-, 10-, 15-, 20- and 30-year time horizons for societal cost-utility analysis. | | | |
| --- | --- | --- | --- |
|  | **Usual care** | **Recommended care** | **Incremental** |
| **5-year time horizon (n=152,138)** | | | |
| Public sector costs^1^ |  |  |  |
| *(1) All-cause public sector costs^2^* | £1,849,611,631 | £1,829,897,263 | -£19,714,368 |
| *(2) Fall-related healthcare costs* | £101,303,106 | £85,069,262 | -£16,233,844 |
| *Public sector intervention costs* | £6,516,944 | £89,739,906 | £83,222,962 |
| QALY | 448,251 | 449,478 | 1,227 |
| Societal outcomes |  |  |  |
| *Net productivity^3^* |  |  | -£4,356,278 |
| *Net private expenditure^4^* |  |  | £14,145,932 |
| *Net informal caregiving cost^5^* |  |  | -£836,358 |
| Societal gain, QALY equivalent |  |  | -294 QALYs |
| **Societal ICER using (1)^6^** |  |  | **£68,077 per QALY gained** |
| **Societal ICER using (2)** |  |  | **£71,808 per QALY gained** |
| **Societal INMB using (1)^7^** |  |  | **-£35,521,833** |
| **Societal INMB using (2)** |  |  | **-£39,002,357** |
| **10-year time horizon (n=187,921)** | | | |
| Public sector costs |  |  |  |
| *(1) All-cause public sector costs* | £3,599,096,165 | £3,555,157,110 | -£43,939,055 |
| *(2) Fall-related healthcare costs* | £209,309,323 | £175,101,250 | -£34,208,073 |
| *Public sector intervention costs* | £12,612,505 | £166,007,887 | £153,395,381 |
| QALY | 830,217 | 834,105 | 3,888 |
| Societal outcomes |  |  |  |
| *Net productivity* |  |  | -£1,328,093 |
| *Net private expenditure* |  |  | £18,442,428 |
| *Net informal caregiving cost* |  |  | -£18,774,415 |
| Societal gain, QALY equivalent |  |  | -17 QALYs |
| **Societal ICER using (1)^8^** |  |  | **£28,272 per QALY gained** |
| **Societal ICER using (2)** |  |  | **£30,785 per QALY gained** |
| **Societal INMB using (1)** |  |  | **£6,691,588** |
| **Societal INMB using (2)** |  |  | **-£3,039,393** |
| **15-year time horizon (n=222,612)** | | | |
| Public sector costs |  |  |  |
| *(1) All-cause public sector costs* | £5,163,749,028 | £5,095,770,591 | -£67,978,437 |
| *(2) Fall-related healthcare costs* | £311,397,486 | £261,631,065 | -£49,766,421 |
| *Public sector intervention costs* | £17,997,433 | £231,483,620 | £213,486,188 |
| QALY | 1,151,454 | 1,158,140 | 6,686 |
| Societal outcomes |  |  |  |
| *Net productivity* |  |  | £11,981,817 |
| *Net private expenditure* |  |  | £20,511,050 |
| *Net informal caregiving cost* |  |  | -£49,053,402 |
| Societal gain, QALY equivalent |  |  | 675 QALYs |
| **Societal ICER using (1)^9^** |  |  | **£19,765 per QALY gained** |
| **Societal ICER using (2)** |  |  | **£22,239 per QALY gained** |
| **Societal INMB using (1)** |  |  | **£75,347,594** |
| **Societal INMB using (2)** |  |  | **£57,135,578** |
| **20-year time horizon (n=252,846)** | | | |
| Public sector costs |  |  |  |
| *(1) All-cause public sector costs* | £6,500,266,055 | £6,416,275,041 | -£83,991,014 |
| *(2) Fall-related healthcare costs* | £406,217,223 | £340,013,243 | -£66,203,980 |
| *Public sector intervention costs* | £22,924,065 | £286,750,828 | £263,826,763 |
| QALY | 1,415,416 | 1,425,836 | 10,421 |
| Societal outcomes |  |  |  |
| *Net productivity* |  |  | £5,841,364 |
| *Net private expenditure* |  |  | £20,743,528 |
| *Net informal caregiving cost* |  |  | -£56,348,033 |
| Societal gain, QALY equivalent |  |  | 691 QALYs |
| **Societal ICER using (1)^10^** |  |  | **£16,185 per QALY gained** |
| **Societal ICER using (2)** |  |  | **£17,786 per QALY gained** |
| **Societal INMB using (1)** |  |  | **£153,505,433** |
| **Societal INMB using (2)** |  |  | **£135,718,399** |
| **30-year time horizon (n=317,525)** | | | |
| Public sector costs |  |  |  |
| *(1) All-cause public sector costs* | £8,581,201,839 | £8,471,616,325 | -£109,585,515 |
| *(2) Fall-related healthcare costs* | £554,779,116 | £465,164,198 | -£89,614,919 |
| *Public sector intervention costs* | £39,519,947 | £372,515,702 | £332,995,755 |
| QALY | 1,810,486 | 1,826,345 | 15,858 |
| Societal outcomes |  |  |  |
| *Net productivity* |  |  | £10,648,465 |
| *Net private expenditure* |  |  | £22,740,785 |
| *Net informal caregiving cost* |  |  | -£82,650,953 |
| Societal gain, QALY equivalent |  |  | 1,176 QALYs |
| **Societal ICER using (1)^11^** |  |  | **£13,115 per QALY gained** |
| **Societal ICER using (2)** |  |  | **£14,288 per QALY gained** |
| **Societal INMB using (1)** |  |  | **£287,619,018** |
| **Societal INMB using (2)** |  |  | **£267,648,422** |
| **Abbreviation:** ICER: incremental cost-effectiveness ratio; INMB: incremental net monetary benefit; LTC: long-term care; OOP: out-of-pocket; QALY: quality-adjusted life year.  ^1^ All outcomes were averaged across 20 model trial runs with different random number seeds.  ^2^ Includes costs of fall-related primary and secondary healthcare, comorbidity primary and secondary healthcare, cost of dying, community healthcare, short-term social care, all-cause long-term care.  ^3^ Includes values of paid and unpaid employment minus intervention time opportunity costs.  ^4^ Includes OOP care expenditure and privately incurred LTC cost minus intervention private co-payments.  ^5^ Includes informal caregiver burden/cost minus intervention caregiver time opportunity costs.  ^6^ The public sector ICERs are £51,746 per QALY gained using (1) and £54,581 using (2).  ^7^ INMBs are estimated using the cost-effectiveness threshold of £30,000 per QALY gained.  ^8^ The public sector ICERs are £28,151 per QALY gained using (1) and £30,654 using (2).  ^9^ The public sector ICERs are £20,558 per QALY gained using (1) and £22,515 using (2).  ^10^ The public sector ICERs are £17,258 per QALY gained using (1) and £18,965 using (2).  ^11^ The public sector ICERs are £14,088 per QALY gained using (1) and £15,347 using (2). | | | |

| **Table C13** Scenarios of discount rate variations for 40-year societal cost-utility analysis. | | | |
| --- | --- | --- | --- |
| N=385,192 | **Usual care** | **Recommended care** | **Incremental** |
| **0% discount rates for health and cost outcomes** | | | |
| Public sector costs^1^ |  |  |  |
| *(1) All-cause public sector costs^2^* | £19,043,000,718 | £18,804,158,243 | -£238,842,476 |
| *(2) Fall-related healthcare costs* | £1,283,630,231 | £1,080,452,217 | -£203,178,014 |
| *Public sector intervention costs* | £66,955,387 | £803,372,331 | £736,416,944 |
| QALY | 3,826,084 | 3,867,600 | 41,517 |
| Societal outcomes |  |  |  |
| *Net productivity^3^* |  |  | £28,132,717 |
| *Net private expenditure^4^* |  |  | £33,066,768 |
| *Net informal caregiving cost^5^* |  |  | -£191,878,650 |
| Societal gain, QALY equivalent |  |  | 3,116 QALYs |
| **Societal ICER using (1)^6^** |  |  | **£11,148 per QALY gained** |
| **Societal ICER using (2)** |  |  | **£11,947 per QALY gained** |
| **Societal INMB using (1)^7^** |  |  | **£841,403,618** |
| **Societal INMB using (2)** |  |  | **£805,739,157** |
| **6% discount rates for health and cost outcomes** | | | |
| Public sector costs |  |  |  |
| *(1) All-cause public sector costs* | £6,923,588,664 | £6,835,978,010 | -£87,610,654 |
| *(2) Fall-related healthcare costs* | £442,811,032 | £371,541,329 | -£71,269,704 |
| *Public sector intervention costs* | £24,335,837 | £302,415,159 | £278,079,322 |
| QALY | 1,472,263 | 1,484,448 | 12,185 |
| Societal outcomes |  |  |  |
| *Net productivity* |  |  | £6,211,613 |
| *Net private expenditure* |  |  | £20,261,231 |
| *Net informal caregiving cost* |  |  | -£60,587,635 |
| Societal gain, QALY equivalent |  |  | 776 QALYs |
| **Societal ICER using (1)^8^** |  |  | **£14,696 per QALY gained** |
| **Societal ICER using (2)** |  |  | **£15,956 per QALY gained** |
| **Societal INMB using (1)** |  |  | **£198,357,077** |
| **Societal INMB using (2)** |  |  | **£182,016,126** |
| **Abbreviation:** ICER: incremental cost-effectiveness ratio; INMB: incremental net monetary benefit; LTC: long-term care; OOP: out-of-pocket; QALY: quality-adjusted life year.  ^1^ All outcomes were averaged across 20 model trial runs with different random number seeds.  ^2^ Includes costs of fall-related primary and secondary healthcare, comorbidity primary and secondary healthcare, cost of dying, community healthcare, short-term social care, all-cause long-term care.  ^3^ Includes values of paid and unpaid employment minus intervention time opportunity costs.  ^4^ Includes OOP care expenditure and privately incurred LTC cost minus intervention private co-payments.  ^5^ Includes informal caregiver burden/cost minus intervention caregiver time opportunity costs.  ^6^ The public sector ICERs are £11,985 per QALY gained using (1) and £12,844 using (2).  ^7^ INMBs are estimated using the cost-effectiveness threshold of £30,000 per QALY gained.  ^8^ The public sector ICERs are £15,631 per QALY gained using (1) and £16,972 using (2). | | | |

| **Table C14** Scenario of no falls-frailty feedback loop for 40-year societal cost-utility analysis. | | | |
| --- | --- | --- | --- |
| N=385,192 | **Usual care** | **Recommended care** | **Incremental** |
| Public sector costs^1^ |  |  |  |
| *(1) All-cause public sector costs^2^* | £10,059,133,198 | £9,955,607,722 | -£103,525,475 |
| *(2) Fall-related healthcare costs* | £653,232,465 | £552,870,256 | -£100,362,210 |
| *Public sector intervention costs* | £34,295,528 | £437,094,863 | £402,799,335 |
| QALY | 2,076,511 | 2,083,406 | 6,895 |
| Societal outcomes |  |  |  |
| *Net productivity^3^* |  |  | -£25,795,971 |
| *Net private expenditure^4^* |  |  | £58,520,172 |
| *Net informal caregiving cost^5^* |  |  | £10,880,063 |
| Societal gain, QALY equivalent |  |  | -1,587 QALYs |
| **Societal ICER using (1)^6^** |  |  | **£56,372 per QALY gained** |
| **Societal ICER using (2)** |  |  | **£56,968 per QALY gained** |
| **Societal INMB using (1)^7^** |  |  | **-£140,007,651** |
| **Societal INMB using (2)** |  |  | **-£143,170,917** |
| **Abbreviation:** ICER: incremental cost-effectiveness ratio; INMB: incremental net monetary benefit; LTC: long-term care; OOP: out-of-pocket; QALY: quality-adjusted life year.  ^1^ All outcomes were averaged across 20 model trial runs with different random number seeds.  ^2^ Includes costs of fall-related primary and secondary healthcare, comorbidity primary and secondary healthcare, cost of dying, community healthcare, short-term social care, all-cause long-term care.  ^3^ Includes values of paid and unpaid employment minus intervention time opportunity costs.  ^4^ Includes OOP care expenditure and privately incurred LTC cost minus intervention private co-payments.  ^5^ Includes informal caregiver burden/cost minus intervention caregiver time opportunity costs.  ^6^ The public sector ICERs are £43,401 per QALY gained using (1) and £43,860 using (2).  ^7^ INMBs are estimated using the cost-effectiveness threshold of £30,000 per QALY gained. | | | |

| **Table C15** Scenarios of 20% reduction in initial frailty and annual rate of frailty progression for 40-year societal cost-utility analysis. | | | |
| --- | --- | --- | --- |
| N=385,192 | **Usual care** | **Recommended care** | **Incremental** |
| **Reduction in initial frailty** | | | |
| Public sector costs^1^ |  |  |  |
| *(1) All-cause public sector costs^2^* | £9,871,409,213 | £9,755,434,731 | -£115,974,482 |
| *(2) Fall-related healthcare costs* | £617,333,093 | £520,669,631 | -£96,663,462 |
| *Public sector intervention costs* | £33,114,114 | £419,420,015 | £386,305,901 |
| QALY | 2,191,155 | 2,209,507 | 18,351 |
| Societal outcomes |  |  |  |
| *Net productivity^3^* |  |  | £5,473,826 |
| *Net private expenditure^4^* |  |  | £19,791,980 |
| *Net informal caregiving cost^5^* |  |  | -£92,339,765 |
| Societal gain, QALY equivalent |  |  | 1,300 QALYs |
| **Societal ICER using (1)^6^** |  |  | **£13,756 per QALY gained** |
| **Societal ICER using (2)** |  |  | **£14,739 per QALY gained** |
| **Societal INMB using (1)^7^** |  |  | **£319,216,233** |
| **Societal INMB using (2)** |  |  | **£299,905,213** |
| **Reduction in rate of frailty progression** | | | |
| Public sector costs |  |  |  |
| *(1) All-cause public sector costs* | £9,919,041,861 | £9,803,335,668 | -£115,706,193 |
| *(2) Fall-related healthcare costs* | £620,064,905 | £523,208,292 | -£96,856,613 |
| *Public sector intervention costs* | £33,261,392 | £422,592,169 | £389,330,777 |
| QALY | 2,173,119 | 2,190,004 | 16,885 |
| Societal outcomes |  |  |  |
| *Net productivity* |  |  | £1,189,219 |
| *Net private expenditure* |  |  | £24,605,080 |
| *Net informal caregiving cost* |  |  | -£82,188,685 |
| Societal gain, QALY equivalent |  |  | 980 QALYs |
| **Societal ICER using (1)^8^** |  |  | **£15,317 per QALY gained** |
| **Societal ICER using (2)** |  |  | **£16,372 per QALY gained** |
| **Societal INMB using (1)** |  |  | **£262,299,190** |
| **Societal INMB using (2)** |  |  | **£243,449,610** |
| **Abbreviation:** ICER: incremental cost-effectiveness ratio; INMB: incremental net monetary benefit; LTC: long-term care; OOP: out-of-pocket; QALY: quality-adjusted life year.  ^1^ All outcomes were averaged across 20 model trial runs with different random number seeds.  ^2^ Includes costs of fall-related primary and secondary healthcare, comorbidity primary and secondary healthcare, cost of dying, community healthcare, short-term social care, all-cause long-term care.  ^3^ Includes values of paid and unpaid employment minus intervention time opportunity costs.  ^4^ Includes OOP care expenditure and privately incurred LTC cost minus intervention private co-payments.  ^5^ Includes informal caregiver burden/cost minus intervention caregiver time opportunity costs.  ^6^ The public sector ICERs are £14,731 per QALY gained using (1) and £15,783 using (2).  ^7^ INMBs are estimated using the cost-effectiveness threshold of £30,000 per QALY gained.  ^8^ The public sector ICERs are £16,206 per QALY gained using (1) and £17,322 using (2). | | | |

| **Table C16** Scenario of higher life expectancy for 40-year societal cost-utility analysis. | | | |
| --- | --- | --- | --- |
| N=385,192 | **Usual care** | **Recommended care** | **Incremental** |
| Public sector costs^1^ |  |  |  |
| *(1) All-cause public sector costs^2^* | £10,495,349,038 | £10,358,144,772 | -£137,204,266 |
| *(2) Fall-related healthcare costs* | £719,869,949 | £608,513,741 | -£111,356,208 |
| *Public sector intervention costs* | £37,746,129 | £452,822,016 | £415,075,888 |
| QALY | 2,157,713 | 2,178,420 | 20,707 |
| Societal outcomes |  |  |  |
| *Net productivity^3^* |  |  | £1,162,561 |
| *Net private expenditure^4^* |  |  | £20,987,322 |
| *Net informal caregiving cost^5^* |  |  | -£92,844,303 |
| Societal gain, QALY equivalent |  |  | 1,217 QALYs |
| **Societal ICER using (1)^6^** |  |  | **£12,674 per QALY gained** |
| **Societal ICER using (2)** |  |  | **£13,853 per QALY gained** |
| **Societal INMB using (1)^7^** |  |  | **£379,843,298** |
| **Societal INMB using (2)** |  |  | **£353,995,239** |
| **Abbreviation:** ICER: incremental cost-effectiveness ratio; INMB: incremental net monetary benefit; LTC: long-term care; OOP: out-of-pocket; QALY: quality-adjusted life year.  ^1^ All outcomes were averaged across 20 model trial runs with different random number seeds.  ^2^ Includes costs of fall-related primary and secondary healthcare, comorbidity primary and secondary healthcare, cost of dying, community healthcare, short-term social care, all-cause long-term care.  ^3^ Includes values of paid and unpaid employment minus intervention time opportunity costs.  ^4^ Includes OOP care expenditure and privately incurred LTC cost minus intervention private co-payments.  ^5^ Includes informal caregiver burden/cost minus intervention caregiver time opportunity costs.  ^6^ The public sector ICERs are £13,419 per QALY gained using (1) and £14,668 using (2).  ^7^ INMBs are estimated using the cost-effectiveness threshold of £30,000 per QALY gained. | | | |

| **Table C17** Scenario of 20% reduction in other-cause mortality risk gap between frailty categories for 40-year societal cost-utility analysis. | | | |
| --- | --- | --- | --- |
| N=385,192 | **Usual care** | **Recommended care** | **Incremental** |
| Public sector costs^1^ |  |  |  |
| *(1) All-cause public sector costs^2^* | £10,123,840,949 | £9,996,651,591 | -£127,189,358 |
| *(2) Fall-related healthcare costs* | £666,782,115 | £561,693,429 | -£105,088,686 |
| *Public sector intervention costs* | £35,806,603 | £436,267,582 | £400,460,978 |
| QALY | 2,099,165 | 2,118,457 | 19,292 |
| Societal outcomes |  |  |  |
| *Net productivity^3^* |  |  | £2,450,997 |
| *Net private expenditure^4^* |  |  | £21,263,281 |
| *Net informal caregiving cost^5^* |  |  | -£90,651,111 |
| Societal gain, QALY equivalent |  |  | 1,197 QALYs |
| **Societal ICER using (1)^6^** |  |  | **£13,337 per QALY gained** |
| **Societal ICER using (2)** |  |  | **£14,416 per QALY gained** |
| **Societal INMB using (1)^7^** |  |  | **£341,412,108** |
| **Societal INMB using (2)** |  |  | **£319,311,437** |
| **Abbreviation:** ICER: incremental cost-effectiveness ratio; INMB: incremental net monetary benefit; LTC: long-term care; OOP: out-of-pocket; QALY: quality-adjusted life year.  ^1^ All outcomes were averaged across 20 model trial runs with different random number seeds.  ^2^ Includes costs of fall-related primary and secondary healthcare, comorbidity primary and secondary healthcare, cost of dying, community healthcare, short-term social care, all-cause long-term care.  ^3^ Includes values of paid and unpaid employment minus intervention time opportunity costs.  ^4^ Includes OOP care expenditure and privately incurred LTC cost minus intervention private co-payments.  ^5^ Includes informal caregiver burden/cost minus intervention caregiver time opportunity costs.  ^6^ The public sector ICERs are £14,165 per QALY gained using (1) and £15,310 using (2).  ^7^ INMBs are estimated using the cost-effectiveness threshold of £30,000 per QALY gained. | | | |
